# Leveraging detection uncertainty to estimate *Renibacterium salmoninarum* infection status among multiple tissues and assays

**DOI:** 10.1371/journal.pone.0323010

**Published:** 2025-05-08

**Authors:** Tawni B. R. Firestone, Eric R. Fetherman, Kathryn P. Huyvaert, John D. Drennan, Rebecca E. Brock, Brooke Yeatts, Dana L. Winkelman

**Affiliations:** 1 Colorado Parks and Wildlife, Aquatic Research Section, Fort Collins, Colorado, United States of America; 2 Fish, Wildlife, and Conservation Biology, Colorado State University, Fort Collins, Colorado, United States of America; 3 Colorado Parks and Wildlife, Aquatic Animal Health Laboratory, Brush, Colorado, United States of America; 4 Colorado Cooperative Fish and Wildlife Research Unit, Colorado State University, Fort Collins, Colorado, United States of America; 5 U.S. Geological Survey, Colorado Cooperative Fish and Wildlife Research Unit, Fort Collins, Colorado, United States of America; Benha University, EGYPT

## Abstract

Effective disease surveillance relies on accurate pathogen testing and robust prevalence estimates. Diagnostic specificity (DSp), the probability that an uninfected animal tests negative, is high when false positives are low. Diagnostic sensitivity (DSe) is the probability an infected animal tests positive; higher DSe means fewer false negatives. However, sensitivity and false negatives are harder to estimate without a “gold standard”, an assay that can detect between 90 − 100% of true positive infections. Occupancy estimation of infection prevalence offers one solution by allowing for imperfect detection of the pathogen. Testing potentially infected tissues multiple times allows for the use of a Bayesian multistate occupancy model to estimate the probability of pathogen infection in tissues $\left(\Psi_{k}\right)$ and detection probabilities (p) for different assays. Using $\Psi_{k}$ and p from the posterior distribution, the conditional probability of detecting the pathogen can be modeled, allowing for the calculation of DSe. *Renibacterium salmoninarum* is a bacterial pathogen causing bacterial kidney disease among salmonid species and was the model pathogen we used to train our model. The current testing standard for salmonids combines initial screening for antibodies using direct fluorescent antibody test (DFAT) with polymerase chain reaction (PCR) confirmation to detect *R. salmoninarum*. However, detection of *R. salmoninarum* still varies between species, tissues, and assays. Here, a multi-state occupancy model was used to estimate detection probability among individual and dual kidney/liver infections with DFAT and qPCR in fish with an unknown infection status. Both assays produced false negatives, but qPCR had fewer than DFAT and a higher DSe. Infection state was often misclassified, but multiple surveys per individual or combining tissues for testing improved DSe for both assays.

## Introduction

Emerging, infectious fish pathogens pose a serious threat for wild fisheries [1] aquaculture [2], and fish biodiversity [3]. Climate change will likely influence the distribution and severity of these pathogens [4,5]. Successful intervention and mitigation of disease outbreaks depend on effective surveillance and early detection of infections [6–8]. Accurate diagnostic testing and robust statistical estimation of pathogen prevalence are critical for effective surveillance and understanding of disease risk [6] in both aquaculture and wild fish populations.

Molecular diagnostic tools are at the forefront of pathogen detection techniques given how quickly results can be obtained, the ability to evaluate a broad range of pathogens, and ability to detect low pathogen levels [8–10]. However, the analytical specificity, sensitivity, and detection limits are often not assessed or are confused with one another [11,12]. Precise terminology is critical for meaningful inferences. For the purposes of our study, we define diagnostic specificity (DSp) as the conditional probability that an uninfected animal will test negative, with higher DSp indicating fewer false positives (i.e., identifying an individual as positive when they are not infected; [11,13,14]). We define diagnostic sensitivity (DSe) as the conditional probability that an infected animal will test positive, with higher DSe meaning fewer false negatives (i.e., identifying an individual as negative for a pathogen when in fact they are infected [11,13,14]). Finally, the detection limit of a particular diagnostic tool or assay is the minimum detectable pathogen level within a sample. Detection limit is frequently referred to as sensitivity or analytic sensitivity and is often confused with the DSe as defined above [11,14]. Correctly defining DSe is important because there is a tradeoff between DSe and DSp. Tests with high DSp, like polymerase chain reaction (PCR) based tests, often have lower DSe [10], meaning they avoid false positives but may result in more false negatives.

Modern molecular assays can identify pathogens with high specificity by targeting precise genetic regions unique to each pathogen, virtually eliminating false positives [15]. These assays may also detect extremely low levels of pathogens in a sample due to their low detection limits [16]. However, accurately assessing sensitivity and the potential for false negatives is more challenging without an independent “gold standard” test to confirm results [13,14]. Because the true infection status of fish or tissue is often unknown, defining DSe and DSp is often not feasible. Numerous approaches like Bayesian latent class analysis can model DSe and DSp without requiring known infection status, enabling estimation in wild populations [14]. However, this approach has limitations when a pathogen infects multiple organs simultaneously, and one wants to understand which tissues are infected at a given time. One possible approach is to model the detectability of an assay by using Bayesian multistate occupancy models, where detection is classified as either detected (1) or not detected (0) to estimate an infected and non-infected status of the host [17]. In occupancy models, detection probability refers to the likelihood of detecting a pathogen from multiple surveys of an infected host, without knowing the true infection status. By surveying multiple tissues that could be infected individually or simultaneously, we can estimate the probability that specific tissue(s) are infected within an individual fish. We can also estimate the detection probability across multiple assays and derive estimates for DSe and DSp. DSe is analogous to the conditional probability of detection, derived from the estimate of the modeled detection probability [18].

The ultimate consequence of false negatives is an underestimate of pathogen prevalence. To minimize this risk, disease detection assays should aim for high DSe, even if it increases false positives and overestimates prevalence. Overestimation is less harmful than underestimation for management decisions, like stocking fish, which rely on prevalence data. To overcome the lack of a gold standard test and the possibility of a false negative result, we applied a Bayesian multistate occupancy approach to estimate the sensitivity of a diagnostic test among multiple tissues and assays for a bacterial pathogen of fish because it does not require knowledge of the true infection status [14].

### *Renibacterium salmoninarum*: A case study

**Fig 1:**
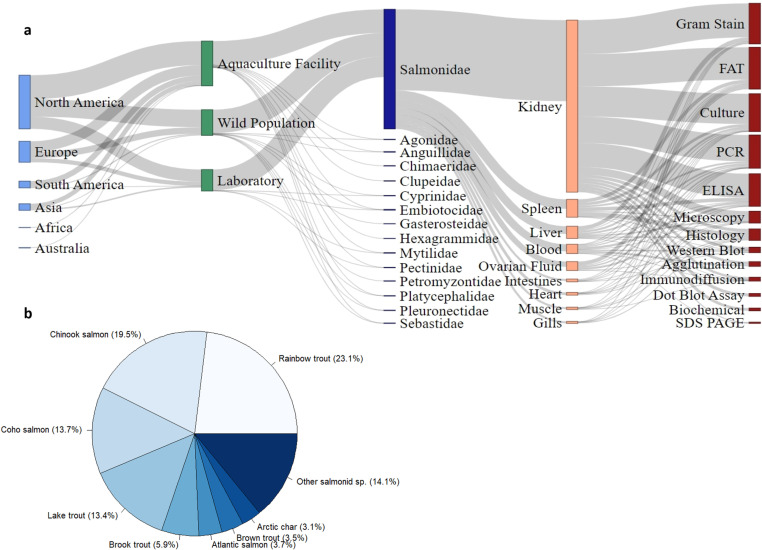
Sankey diagram and species distribution of *Renibacterium salmoninarum* infections in salmonids. a) Each node in the diagram, rectangles and text, represents one set of the data we collected from the literature (i.e., location, source, species, tissue, and assay). The connections represent the relationship between two nodes and are proportional to number of reports for each of the data nodes. b) Pie chart showing the relative distribution of salmonid species that have had a *Renibacterium salmoninarum* infection documented in the literature. All citations used to produce the Sankey diagram and pie chart are included in S1 Appendix.

**Fig 2:**
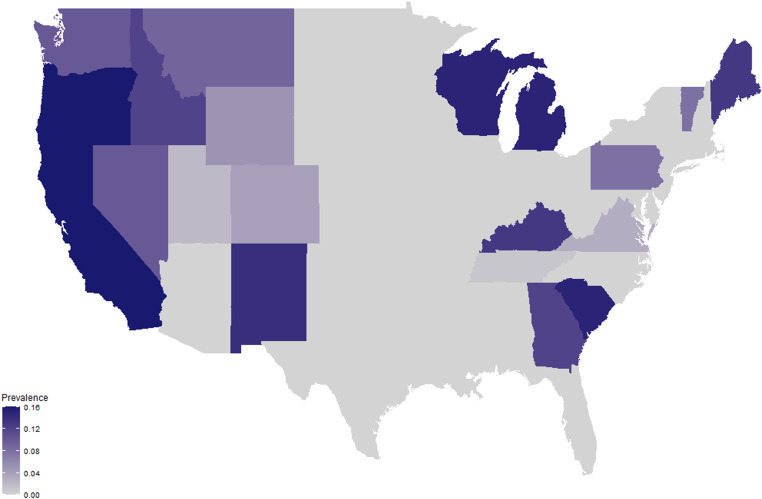
Geographic distribution of *Renibacterium salmoninarum* detections in inland United States. Detections of *Renibacterium salmoninarum* with PCR in the inland U.S. states. Darker colors represent higher detections of the bacteria among individual fish. Data collected from the U.S. Fish and Wildlife Service, wild fish survey in February 2019.

Bacterial kidney disease (BKD) is a globally distributed fish disease caused by the bacterium *Renibacterium salmoninarum*. While most research has focused on understanding and controlling BKD in aquaculture facilities in Europe and North America (Fig 1a), most studies have examined the disease in wild salmonid populations, particularly in the Pacific Northwest of the United States (Fig 2). Salmonids account for most documented cases of BKD, reflecting their economic importance, but the pathogen has also been detected in non-salmonid species (Fig 1a). The focus of research on farmed salmonids may not reflect the overall ecological impact of BKD.

Kidney tissue is the primary tissue that gets tested for *R. salmoninarum*, but other tissues like spleen, liver, blood, and ovarian fluid have also been used (Fig 1a). While isolation and culture of *R. salmoninarum* on selective kidney disease medium followed by a serological test was previously used as the definitive diagnostic method [19,20], molecular techniques are now more common for assessment because they are faster and less labor-intensive (Fig 1a) [20–22]. Furthermore, bacterial culture of *R. salmoninarum* can be less sensitive than molecular techniques, especially when bacterial loads are low or when the bacteria is unevenly distributed across tissues [15,23].

Due to the need for rapid assessment of infection status during salmonid disease outbreaks, detection methods typically utilize antibody-based tests like direct fluorescent antibody tests (DFAT), membrane-filtration FAT (mFAT), and enzyme-linked immunosorbent assays (ELISA) or molecular tests such as polymerase chain reaction assays (PCR; Fig 1a) [12,22,24]. Current guidelines for quick, initial detection of *R. salmoninarum* involve screening kidney tissues or ovarian fluid using DFAT, mFAT, and ELISA [24,25], but these tests may not be sensitive enough for detections of subclinical infections. Presumptive positives from these tests are confirmed with a more sensitive PCR assay including quantitative (qPCR), nested (nPCR), or single-round PCR [12,22,26,27]. Current methods for detecting *R. salmoninarum* vary in DSe and DSp depending on fish species [22,28–30]. In fact, most assays have not consistently identified infections across methods, and presumptive negatives obtained from initial screening tests are generally not confirmed using molecular assays, leading to false negative results [31]. Therefore, understanding the efficacy and tradeoffs of screening particular tissues and with different assays to detect *R. salmoninarum* across susceptible fish species is critical.

Bacterial kidney disease primarily infects kidney tissue, but the bacteria may also spread without obvious symptoms to other organs, like the liver and spleen, as well as heart, brain, and mucus during different times of infection (Fig 1a; [32]). For example, one study of Chinook salmon (*Oncorhynchus tshawytscha*, Salmonidae) found lesions on liver tissue but no signs of infection in the kidneys [32]. Moreover, the distribution of *R. salmoninarum* can be uneven, resulting in variable detections depending on the sample location and bacterial load [33]. Additionally, infections may progress through different tissues at different times, also contributing to variable detection results [34,35].

Cutthroat trout (*O. clarkii*, Salmonidae), a species of significant conservation concern in the western U.S., are particularly susceptible to *R. salmoninarum* [27,36–39]. These fish are routinely raised in hatcheries for stocking and reestablishing wild populations. Given their susceptibility to *R. salmoninarum* infections, regular fish health inspections are conducted at hatcheries to detect the bacterium before stocking. Recent and continued detections of *R. salmoninarum* in Colorado hatcheries further underscore the need for continuous monitoring [27]. Specifically, in one Colorado hatchery, cutthroat trout were moved onto the facility after presumptively testing negative for *R. salmoninarum*. However, during subsequent routine annual health inspections, these fish were found to be infected with *R. salmoninarum* [27]. Although initial testing was negative, the detection of *R. salmoninarum* at later stages highlighted a need for improvement in diagnostic methods. Furthermore, during a recent wild fish survey conducted within Colorado waters, 19 cutthroat trout populations were tested, with 100% of those populations showing evidence of an *R. salmoninarum* infection in at least one fish [38].

The growing number of *R. salmoninarum* detections in both hatchery and wild populations prompted a reassessment of testing protocols to ensure the most effective methods were being used to detect *R. salmoninarum* in cutthroat trout. In response to this, we collaborated with a hatchery experiencing an outbreak of *R. salmoninarum* among a population of adult cutthroat trout. This provided a unique opportunity to conduct comprehensive testing, during which we were able to euthanize and test all fish to ensure thorough analysis of infection status, and assess the efficacy of assays for detecting the bacterium across a wide range of infection states and tissue types.

Our study aligns with the current standards for fish health testing, which follow the American Fisheries Society (AFS) guidelines [24]. These guidelines recommend the use of DFAT for screening fish and PCR analysis for confirming infections. Our literature review further justifies our approach by indicating PCR and DFAT as the predominant rapid detection methods for testing tissues for *R. salmoninarum*. By compiling relevant studies (Fig 1; S1 Appendix), we were able to select appropriate tissues and assays for our own testing. We sampled adult spawning cutthroat trout from Colorado Parks and Wildlife’s (CPW) Poudre Rearing Unit. At the time, the overall population was known to be positive for *R. salmoninarum*, although the infection status of individual fish was unknown. Our two main objectives were to: 1) compare the probability of *R. salmoninarum* infection across tissues to determine which provides the highest detection probability, if present, and 2) assess the diagnostic sensitivity of DFAT and qPCR to determine the best assay for diagnosing *R. salmoninarum* infections in hatchery fish populations. Further, we developed a Bayesian multi-state occupancy model to help explain the complexities associated with detection of *R. salmoninarum*. Our approach allowed us to estimate detection probabilities for both liver and kidney tissues and DFAT and qPCR assays, as well as calculate conditional probabilities to inform estimates of DSe.

## Materials and methods

### Literature review data collection

The detection of *R. salmoninarum* in fish populations has been a challenging endeavor for researchers worldwide due to the pathogen’s complex biological characteristics and elusive nature within fish tissues. Past studies of this bacterium have utilized a diverse array of assays and sampling methods across various geographic regions and fish species. However, this wide variation in techniques has led to inconsistent and often conflicting results regarding the true prevalence, distribution, and detection of *R. salmoninarum*. To address this critical knowledge gap and bring clarity, we compiled detection data from available studies conducted globally into a detailed Sankey flow diagram and pie chart.

We identified studies published in peer reviewed journals from Web of Science and Google Scholar using combinations of search terms associated with bacterial kidney disease, Dee disease (common name for BKD between 1930–1969), *Corynebacterium* spp. (scientific name between 1930–1980), and *R. salmoninarum* research from 1900–2021. We also identified studies by searching the literature cited sections of papers obtained during the initial search. The literature search occurred in August 2021. Due to the goals of our study, not all published studies were included within our dataset. We only included studies that described detection of *R. salmoninarum* from an aquatic species that was collected from the field, hatchery, or aquaculture facility, or experimentally injected in the laboratory (Fig 1). We excluded any publication that described the cellular capabilities of individual *R. salmoninarum* isolates and if the bacteria were never used in a fish inoculation experiment. We extracted data manually from the text, tables, figures, and supplementary information including geographic location of the infected fish, source of the infected fish (hatchery, field, or laboratory), year of detection, tissue tested, and assay used for a detection. We only included a report of one positive detection from each location in the event that detection of *R. salmoninarum* in more than one fish of the same species was indicated from the same water body or the same hatchery.

Our graphical approach using a Sankey flow diagram (Fig 1a) highlights the complex web of relationships between the geographic regions, fish species, infection type (i.e., laboratory, field, and hatchery), the detection assays implemented, and the specific tissues assayed. For example, the diagram reveals that fluorescent antibody tests (FAT), culture, PCR, and ELISA are most commonly performed using kidney tissues, but are used less often with other tissues. Furthermore, the accompanying pie chart (Fig 1b) offers a succinct summary of *R. salmoninarum* detections in salmonid species across the literature given the assortment of assays used. Together these two figures provide an insightful visualization of the challenges and variability inherent in *R. salmoninarum* surveillance. Our synthesis of these data allowed us to justify our own methodology by selecting appropriate tissues and assays based on the compiled studies. The literature supporting the Sankey diagram can be found in S1 Appendix.

### Distribution of *R. salmoninarum* in the U.S.

The Sankey Diagram shows that most *R. salmoninarum* detections occurred in North America, mostly in the U.S., but the literature review data only report those detections that are published. To understand the distribution of detections across the U.S., we gathered data from the U.S. Fish and Wildlife Service that regularly analyzes pathogen detections from a National Wild Fish Survey. This survey includes both hatchery and wild populations tested during fish health inspections, and provides a more comprehensive list of detections than what is currently published in the literature. Thus, data on salmonid fish infected with *R. salmoninarum* in each U.S. state were compiled from the U.S. Fish and Wildlife Service National Wild Fish Survey database from 1960–2015 [40]. Only polymerase chain reaction (PCR) assay detections were included, as this was the most common assay used in the surveys (Fig 2). Once data were collected from the publically accessible website [40], we used RStudio to create a base map of the inland United States. The base map was loaded from the library ggmap using the function map_data(). The U.S. Fish and Wildlife Service National Wild Fish Survey publically accessible data were uploaded to RStudio to create a gradient-filled depiction of PCR prevalence across the inland US using the libraries ggplot2 and ggthemes and the function geom_map().

### Tissue collection

This animal study protocol was approved by the Institutional Animal Care and Use Committee Review Board of Colorado State University through written consent (protocol number 721).

We sampled 781 adult cutthroat trout at the CPW Poudre Rearing Unit during the 2019 spawning season to test for the presence of *R. salmoninarum*. The Sankey analysis showed that spleen, liver, and kidney were most often sampled (Fig 1a) so we collected the spleen, liver, and kidney from the cutthroat trout. All samples were collected from individual three-year-old female (n = 389) and two-year-old male (n = 392) brood fish. All fish were weighed (g), measured (mm), and euthanized using tricaine methanesulfonate (MS-222; Syndel, Ferndale, WA, USA). We removed whole spleen, liver, and kidney tissues through an abdominal incision and placed each tissue into individual sterile whirl-pak-bags. All tissue samples were labelled so they could be associated with individual fish. Samples were placed on dry ice until arrival at the laboratory (6 hours after collection) and held at -20°C until processed.

### Laboratory analysis

All samples were analyzed for the presence of *R. salmoninarum* using two different assays, DFAT and qPCR. Samples were prepared for DFAT by making two tissue imprints per tissue with sterile cotton swabs on individual wells located on a 12-well slide (6 individual fish tissue per slide). A separate control slide with a smear of known *R. salmoninarum* ATCC 33209 culture [24] was also prepared with each set of slides to ensure that the reagents were working properly. We used a *Fluorescein isothiocyanate*-conjugate (FITC), affinity purified polyclonal antibody to *R. salmoninarum* derived from goat (KPL; Milford, MA, USA) to stain the slides, followed by an eriochrome black T counterstain (Sigma-Aldrich®; [25]). We examined all slides under 500x magnification using a FITC fluorescent lamp with a wavelength of 400 nm. Samples exhibiting visible fluorescent cells were further examined at 1000x magnification and were confirmed as *R. salmoninarum* by cell morphology and cell size. Any cells that fluoresced in a sample smear where diplobacilli bacterial cells were present and measured approximately 1.0 X 0.5 µm, were considered positive [24]. *Renibacterium salmoninarum* was detected in individual tissue samples by noting the presence (1) or absence (0) of cell fluorescence.

Replicate individual tissues were used to screen for *R. salmoninarum* with qPCR. For DNA extraction, all samples were thawed and homogenized. Individual spleen, liver, and kidney samples were homogenized manually with a sterile rolling pin while remaining in the whirl-pak-bags. Two replicates of approximately 0.25 g of tissue were prepared for DNA extractions. DNA extractions were completed using a Qiagen DNeasy Blood and Tissue Kit (Qiagen, Germantown, MD, USA). We increased the AE buffer in all DNA extraction protocols to 400 μL to properly rinse filters of all DNA [22].

We used an ABI StepOnePlus System (Applied Biosystems, Foster City, CA, USA) for all qPCR assays to detect the 69-base pair DNA segment of the *msa* gene of *R. salmoninarum* [12]. Each sample resulted in a final volume of 5 μL of extracted DNA template with TaqMan Gene Expression Master Mix (ThermoFisher, Waltham, MA, USA) using predetermined primer sets RS 1238 Forward, 5’-GTGACCAACACCCAGATATCCA-3’, RS 1307 Reverse, 5’-TCGCCAGACCACCATTTACC-3’, and MGB probe 1262, 5’-CACCAGATGGAGCAAC-3’ for each PCR reaction [12]. DNase free water was used as a no template control for each plate.

Known positive controls, obtained from adult rainbow trout (*O. mykiss*, Salmonidae) at the CPW Bellvue Fish Research Hatchery, were used with each qPCR run and known negative controls obtained from rainbow trout were used with every 10^th^ qPCR run. The isolate for the positive controls was identified through 16S rRNA gene sequencing (Genewiz, Azenta Life Sciences) and was confirmed as *R. salmoninarum* by blasting the product. The bacteria were stored as a stock culture in phosphate buffered solution (PBS) at −80°C until use. Positive controls were used to produce a standard curve for the absolute quantification of *R. salmoninarum* in each sample. We rehydrated the stock culture to inoculate kidney disease medium broth (KDM) and inoculated the bacteria five times in new media over a total of 45 days to ensure the culture was pure. Stocks with 1 mL of the cultured bacteria were prepared with an optical density value of 0.181 at a wavelength of 420 nm. Eight, ten-fold serial dilutions were prepared from the stock to quantify the number of bacteria in each dilution. Bacterial cells were centrifuged at 5000 x *g* for 20 min at 4°C. The pellet was re-suspended in 1X PBS-peptone. We followed the protocols for membrane-filtration fluorescent antibody test (MFAT) to quantify the number of cells present in each dilution [41,42]. Syringes containing serial dilutions were fit with Whatman® pop-top filter holders, 13 mm-0.2 μm polycarbonate filters, and 13 mm-5.0 μm nylon membrane filters, and samples were forced through the filters. Filters were rinsed with PBS plus Triton-X and incubated with 100 μL of *Fluorescein*-labeled, affinity purified polyclonal antibody to *R. salmoninarum* for one hour; rinsed again and counterstained with eriochrome black T; and rinsed a final time with 1X PBS. Following rinsing, the polycarbonate filter was placed onto a glass microscope slide to air dry, and a glass coverslip was mounted with DABCO-glycerol medium. Filters on the slides were examined at 1000x magnification to quantify the number of bacterial cells present in each dilution, when there was an observable amount to count. We were able to quantify cells from the MFAT standards from the 10^-6^ dilution. Ten replicate 10^-6^ dilutions were used to calculate the mean number of bacterial cells (bacterial cells mL^-1^) within the dilution. The final five-point curve was generated by plotting five-$\log_{10}$ bacterial concentrations from 58 ten-fold serial dilutions against the quantification cycle (Cq) output values from qPCR to determine slope, Cq cut-off value, and amplification efficiency. Detections of *R. salmoninarum* in individual tissue samples were recorded by noting whether DNA was detected (1) or not (0).

### Statistical analysis

To determine if more detections occur in the two most infected tissues, kidney and liver, due to a higher number of bacteria in one tissue over the other, differences between the number of bacteria present in the tissues were determined by performing a Welch’s two sample t-test in program R (version 4.1.0) with a significance (α) set at 0.05.

### Occupancy model

Multistate occupancy modeling was used to estimate the probability of *R. salmoninarum* infection in tissues and detection probabilities among assays. We used this approach because of imperfect detection of *R. salmoninarum* with DFAT and qPCR and wanted to account for the possibility of false negatives (i.e., not detecting the pathogen when it is actually present). We focused on kidney and liver tissue for the modeling because kidney is the standard tissue recommended by AFS-Fish Health Blue Book for *R. salmoninarum* evaluation, and the raw data indicated that *R. salmoninarum* was detected more often in the liver than the kidney tissue. The sampling unit was an individual fish, and the kidney and liver tissues were considered “locations” within each fish [43]. The replicate testing on each individual tissue represented two surveys for each assay (DFAT or qPCR). We assumed that there were no false positives with either assay based on the previously determined high diagnostic specificity (DSp) of both assays (DFAT: 0.85; qPCR: 1.0; [22]).

The data consisted of detection histories from both assays. The detection histories were produced using the results of the two surveys (j) for each tissue type, kidney $\left(K_{j=1};K_{j=2}\right)$ or liver $\left(L_{j=1};L_{j=2}\right)$, resulting in 16 possible detection histories (d; Table 1). For instance, the following detection history indicates that an individual fish tested negative on both surveys in each tissue (with two observations each for kidney and liver)

**Table 1:** Detection history and probability of *Renibacterium salmoninarum* infections across tissues and infection states. Possible detection history for each state of *Renibacterium salmoninarum* infection consisting of detection (1) or lack thereof (0) from two surveys (S1 and S2) conducted on each tissue (kidney and liver). Probability of infection (Ψ) for each assay (DFAT and qPCR^*^) is listed for each possible state of infection.

Tissue						
Kidney		Liver				
S1	S2	S1	S2	Infection State	Ψ (DFAT)	Ψ (qPCR)
0	0	0	0	State 1 {K − L−}	0.83	0.21
0	1	0	0	State 2 {K + L−}	<0.01	0.10
1	0	0	0	State 2 {K + L−}		
1	1	0	0	State 2 {K + L−}		
0	0	1	0	State 3 {K − L+}	0.10	0.43
0	0	0	1	State 3 {K − L+}		
0	0	1	1	State 3 {K − L+}		
1	0	1	0	State 4 {K + L+}	0.06	0.26
1	0	0	1	State 4 {K + L+}		
0	1	0	1	State 4 {K + L+}		
0	1	1	0	State 4 {K + L+}		
1	1	1	1	State 4 {K + L+}		
1	1	1	0	State 4 {K + L+}		
1	1	0	1	State 4 {K + L+}		
1	0	1	1	State 4 {K + L+}		
0	1	1	1	State 4 {K + L+}		

* DFAT, direct fluorescent antibody test; qPCR, quantitative polymerase chain reaction.


$$d=\left[K_{1}K_{2}L_{1}L_{2}\right]=[0000]$$ (1)

The sixteen possible detection histories resulted in four possible states of infection for each assay; state 1: {K−L−}, state 2: {K+L−}, state 3: {K−L+}, and state 4: {K+L+} where += detection in the tissue on either survey (Table 1). The states of detection are defined as follows. When testing the kidney and liver tissues, *R. salmoninarum* may be present in the tissue (true positive = 1), not present (true negative = 0), or present but not detected (false negative = 0). Due to the possibility of false negative results, there may be multiple possible true states of infection for any detection history with a zero, ultimately affecting the classification of the observed infection state. In our detection history example above, the individual would be classified in observed state 1{K−L−}, which could correctly represent a true state of infection of {K−L−} if both tissues were truly negative. However, that individual could also be positive in one or both kidney surveys (a false negative for kidney) and/or in one or both liver surveys (a false negative for liver) and the true infection state could be {K+L−},{K−L+},or{K+L+}. The model is static with no transition states of infection because fish were euthanized, making multiple measurements over time impossible. Therefore, we model the probability that the observed state represents the true state of infection.

Prior to running the multistate occupancy models, we used a multinomial logistic regression to evaluate the significance of fish length, weight, and sex on *R. salmoninarum* infection in kidney and liver tissues to determine if any of these covariates affected the state or observation process [44]. Only models incorporating individual effects were included because length, weight, and sex are known to be correlated because female fish are larger than male fish. No covariates were determined to be statistically significantly associated with infection status (S1 Table); therefore, these covariates were not used in the occupancy modeling.

The static multistate occupancy model was fit in a Bayesian hierarchical framework (S1 Equation) and was used to calculate the probability of infection $\left(\Psi_{k}\right)$ for each state of infection (k) for each fish (i), and detection probability in kidney $\left(p_{2}\right)$ and liver tissues $\left(p_{3}\right)$ for each assay (DFAT and qPCR; modeled independently) between two surveys (j):


$$\left[p_{2},p_{3},z_{i},\beta_{\Psi ,1},\beta_{\Psi ,2},\beta_{\Psi ,3},\beta_{\Psi ,4}|y_{i,j}\right]\propto$$

$$\prod_{i=1}^{781}\prod_{j=1}^{2}\left[y_{i,j}|p_{2},p_{3},z_{i}\right]\left[z_{i}|\beta_{\Psi ,1},\beta_{\Psi ,2},\beta_{\Psi ,3},\beta_{\Psi ,4}\right]\left[p_{2}\right]\left[p_{3}\right]\left[\beta_{\Psi ,1}\right]\left[\beta_{\Psi ,2}\right]\left[\beta_{\Psi ,3}\right]\left[\beta_{\Psi ,4}\right]$$ (2)

**Fig 3:**
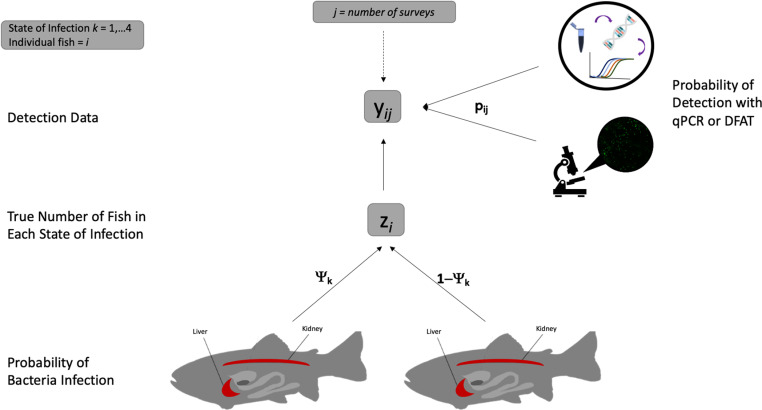
Conceptual hierarchical occupancy model for *Renibacterium salmoninarum* detections. Conceptual diagram of hierarchical occupancy model where $\Psi_{i}$ is the probability of infection for each state of infection, $z_{i}$is the true occupancy within each state of infection, $p_{ij}$ is the probability of detecting *Renibacterium salmoninarum* from either DFAT or qPCR (modeled independently) for the state of infection i during survey j, and $y_{ij}$ is the detection data (1, 2, 3 or 4) for each state of infection i during survey j.

The observed dataset $\left(y_{i,j}\right)$ was derived from 16 possible combinations of detections with 781 fish (i) with two surveys per fish (j) and consisted of the four infection states described above (Table 1). Two surveys per fish were reasonable for this model because MacKenzie and Royle [17] indicate if the diagnostic specificities of the assay(s) is over 0.8 then two surveys is sufficient. In our case the optimized DFAT and qPCR assays have diagnostic specificities of 0.85 and 1.0, respectively [22]. The model was split into two process models: the state process model and the observation process model [45] which both affect the outcome of determining infection status in individual fish. The state process model describes that the tissues representing the states of infection (k=1,2,3,or4) are either infected with *R. salmoninarum* (with probability $\Psi_{k}$) or not infected (with probability $1-\Psi_{k}$) in each fish and $z_{i}$ represents the true unknown latent state of infection for each fish derived from each $\Psi_{k}$ (Fig 3). The observation process model describes the presence or absence of *R. salmoninarum* among each state of infection $\left(p_{i,j}\right)$. These process models are described in detail below.

### State process model

The state process model describes the probability of infection $\left(\Psi_{k}\right)$ in a fish for each state (k) and the true latent state of an infected fish$\left(z_{i}\right)$. The latent state is calculated because of the possibility of false negative results. $z_{i}$ can only be equal to 1, 2, 3, or 4 indicating each true state of infection and is modeled as a categorical random variable from Φ:


$$z_{i}\sim categorical\left(\Phi \right)$$ (3)

$$\Phi =[\Psi_{1}\Psi_{2}\Psi_{3}\Psi_{4}]$$ (4)

Probabilities of infection are calculated for each of the four states: $\Psi_{1}$ is the probability of infection for state 1, $\Psi_{2}$ is the probability of infection for state 2, $\Psi_{3}$ is the probability of infection for state 3, and $\Psi_{4}$ is the probability of infection for state 4. We assigned a Dirichlet prior [40] for the probabilities of infection and constrained the four probabilities by sampling the hyperparameters $\left(\beta_{\Psi ,k}\right)$ from a non-informative gamma distribution (gamma(1,1)). This Dirichlet prior is the most commonly used prior distribution for categorical variables and allows us to describe the probabilities of outcomes [44,46]. Specifically, the distribution represents the marginal probabilities such that the values of the $\Psi_{k}$ sum to one. For instance, the probability of infection for kidney tissues is equivalent to ${\mathrm{Pr}\left(\Psi^{Kidney}\right)=\Psi }_{2}+\Psi_{4}$, the probability of infection for liver tissues is equivalent to $\mathrm{Pr}(\Psi^{Liver})=\Psi_{3}+\Psi_{4}$, and the probability of infection within either tissue is ${{Pr(\Psi }^{Kidney}+\Psi }^{Liver})=\Psi_{2}+\Psi_{3}+\Psi_{4}.$

### Observation process model

The observation process model describes the presence or absence of *R. salmoninarum* within each state of infection. In the observation process model, the observation process $\left(P_{i,j}\right)$ is linked to the observed data for each assay based on the repeated surveys (j) with each tissue.


$$y_{i,j}=Categorical(P_{i,j},z_{i})$$ (5)

We define two detection probabilities $\left(p_{2},p_{3}\right)$ with each assay model. Specifically, we were most interested in understanding the detection probability for each assay on kidney ${(p}_{2})$ and liver tissues $\left(p_{3}\right)$. Thus, we formulated an observation matrix for the parameters $p_{2}$ and $p_{3}$, and arranged them where each row indicates the true state of infection, and each column indicates the observed state of infection and is as follows:


$$P_{i}=\begin{array}{c} 1\\ \begin{array}{c} 2\\ \begin{array}{c} 3\\ 4 \end{array} \end{array} \end{array}\left[\begin{array}{c} \begin{array}{cc} & 1\\ & 1 \end{array}\\ \begin{array}{c} 1-p_{2}\\ \begin{array}{c} 1-p_{3}\\ \left(1-p_{2}\right)*\left(1-p_{3}\right) \end{array} \end{array} \end{array}\begin{array}{c} \begin{array}{cc} & 2\\ & 0 \end{array}\\ \begin{array}{c} p_{2}\\ \begin{array}{c} 0\\ p_{2}*\left(1-p_{3}\right) \end{array} \end{array} \end{array}\begin{array}{c} \begin{array}{cc} & 3\\ & 0 \end{array}\\ \begin{array}{c} 0\\ \begin{array}{c} p_{3}\\ \left(1-p_{2}\right)*p_{3} \end{array} \end{array} \end{array}\begin{array}{c} \begin{array}{cc} & 4\\ & 0 \end{array}\\ \begin{array}{c} 0\\ \begin{array}{c} 0\\ p_{2}*p_{3} \end{array} \end{array} \end{array}\right]$$

If the true infection state is state 1 {K−L−} (first row of $P_{i}$), it is impossible to observe infection states 2, 3, or 4 because the fish is not infected. Therefore, the probability must be 1. When the true state of infection is state 2 {K+L−} (second row of $P_{i}$), the fish could be observed in either state 1 or state 2. As such, the probability of detecting the fish in state 1 given the true state of 2, a false negative from the kidney tissue, is $\mathrm{Pr}(1-p_{2})$ and in state 2 given the true state of 2, a true positive, is $\mathrm{Pr}(p_{2})$. Similarly, when the true state of infection is state 3 (third row of $P_{i}$), the fish could be observed in either state 1 or state 3. Therefore, the probability we detect the fish in state 1 given the true state of 3 is $Pr(1-p_{3})$ and in state 3 is $\mathrm{Pr}(p_{3})$ and state 2 or 4 cannot be observed. Finally, if the true state of infection is state 4 (row four in $P_{i}$) it is possible to detect fish in all four states and the probabilities are defined in $P_{i}$ matrix. The parameters $p_{2}$ and $p_{3}$ are defined as the probability of detection for kidney (state 2 {K+L−}) or liver (state 3 {K−L+}), respectively, and can be derived from $P_{i}$. Non-informative priors were used for $p_{2}$ and $p_{3}$ drawn from a uniform distribution between 0 and 1.

### Model implementation

Posterior probability distributions of the model parameters $\left(p_{2},p_{3},\beta_{\Psi ,1},\beta_{\Psi ,2},\beta_{\Psi ,3},\beta_{\Psi ,4}\right)$ were estimated with separate models for each assay (DFAT, qPCR) using a Monte Carlo-Markov chain (MCMC) algorithm in program JAGS (version 4.3.0) within program R with the library rjags. Both assay models were fit for each state of infection using 10,000 MCMC iterations for three chains with a thinning interval of two, and a burn in value of 1000 for each chain. Model convergence was determined based on the Gelman-Rubin statistic (Rhat) comparing within-chain variance to between-chain variance and was considered acceptable when the Rhat value was less than 1.1 [47]. The models were validated by fitting the model with simulated data to known parameters and recovering the parameters from the model. Additionally, initial analysis of the occupancy model was focused on maximizing the area under the curve (AUC) to ensure appropriate model significance. We computed the AUC with the multiclass.roc function in the pROC package version 1.18.0 in R Studio version 4.2.1.

**Fig 4:**
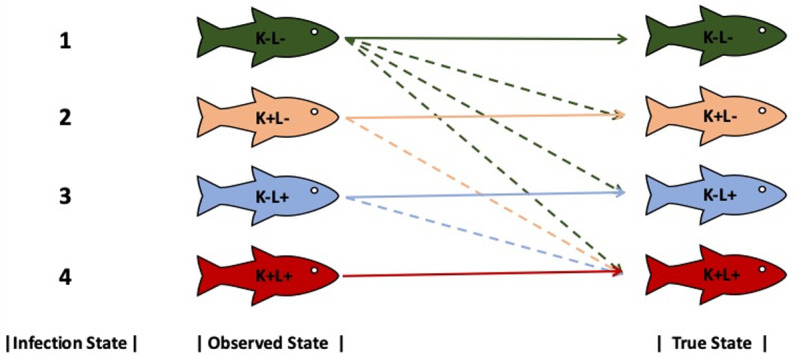
Observed versus true infection states for *Renibacterium salmoninarum* detection. Conceptual diagram of observed and true infection states and possible combinations of how a true infection state may be observed with DFAT or qPCR (indicated by colored lines; K: Kidney, L: Liver, − : negative detection, + : positive detection). Solid lines indicate the probability the observed fish is in the true state and dashed lines indicate the probability of the observed fish is in a different state.

The model calculates the probability of detection from what is observed given truth. However, for most situations we felt it would be more useful to evaluate the probability of an observed state being true or not (Fig 4). For instance, if we observe a fish in state 1, the true state could be any of the four states due to false negative results with kidney or liver tissues (states 2–4) or because the true state is state 1. We used the probability of detections from $P_{i,j}$ for each of the DFAT and qPCR models (S1 Fig) and transformed the values to determine the probability of the true state of infection given what was observed. The new probabilities are derived by taking each value in a row of the matrix $P_{i}$ (true value) and dividing them by the sum of their column values (observed state) from $P_{i}$. For instance, if we observe state 2 and want to know the probability that the true state of infection is state 2, then, from $P_{i}$, we can divide the row value, $p_{2}$, by the sum of the second column, $p_{2}+(p_{2}*\left(1-p_{3}\right)$. To determine the probability that the possible true state of infection is state 4 when we observe state 2, from $P_{i}$, we divide the row value of $p_{2}*(1-p_{3})$ by the sum of the second column, $p_{2}+{(p}_{2}*(1-p_{3})$). These probabilities are what we use to discuss the results below.

### Conditional probability

Using the probability of infection for kidney $\Psi_{2}$ and liver tissues ($\Psi_{3}$; Table 1) from the posterior distribution, we computed the conditional probability of detecting *R. salmoninarum* in K number of surveys with a specific tissue and assay type given the tissue is infected. Conditional probability of detection (referred to hereafter as DSe) was modeled as a function of the number of surveys for DFAT or qPCR assays on each tissue. We modified Equation 1 from Chaudhary et al. [18] where the numerator is the product of the estimated probability of *R. salmoninarum* detection for a survey in a tissue(s), given that the tissue(s) is infected. The denominator is the estimated probability neither kidney nor liver tissue is infected with *R. salmoninarum* (state 1). For example, when estimating the DSe from liver tissue (p^LK), we can compute p^LK where the sum of ${\hat{\Psi }}^{state3}$ and${\hat{\Psi }}^{state4}$ is the marginal probability of occupancy for liver as:


p^LK=Ψ^state3+state4[1−(1−p^L)K]1−Ψ^state1 (6)

## Results

### Tissue collection summary

We evaluated *R. salmoninarum* infection status among 781 cutthroat trout (392 2-year-old males and 389 3-year-old females) during the 2019 spawning season at the CPW Poudre Rearing Unit. Average weights of male fish were 242.7 ± 22.5 g (range 124 − 358 g), and average weights of female fish were 515.82 ± 135.86 g (range 157 − 1,089 g). The multinomial regression indicated no significant differences in *R. salmoninarum* infections between male and female fish, or fish size, for any tissue or assay. Therefore, we did not include sex, length, or weight in the analysis (S1 Table). In total, we collected 781 spleen samples, 776 liver samples, and 778 kidney samples from the 781 fish evaluated. Some samples were not included in the evaluation due to mishandling of the tissues and the potential for tissue contamination.

### qPCR standard curve

**Fig 5:**
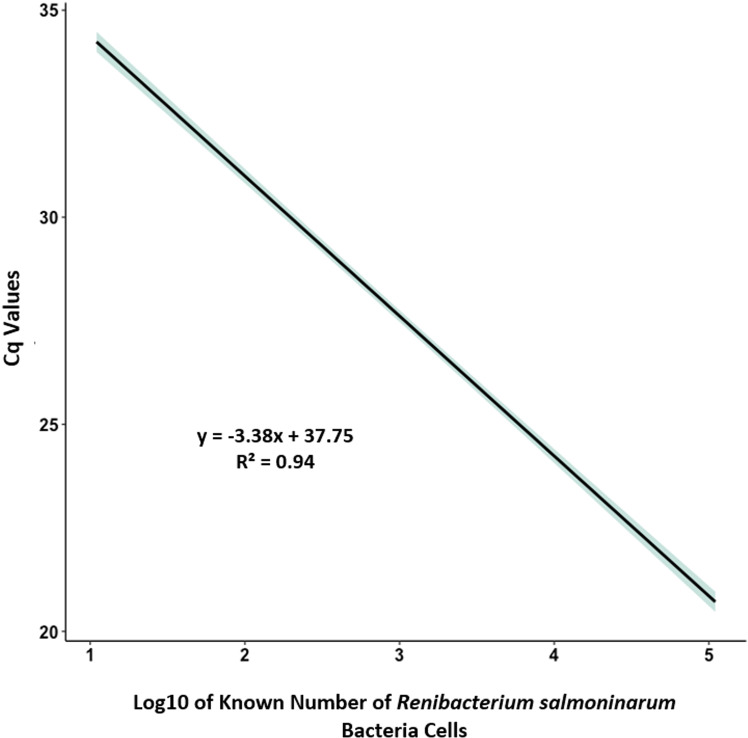
Standard curve for quantification of *Renibacterium salmoninarum* using qPCR. Standard curve generated from 58, five-log_10_ serial dilutions of *R. salmoninarum* with associated confidence intervals*.* Cq values are plotted as a function of five-log_10_ of the known number of bacterial cells that were quantified by membrane-filtration fluorescent antibody test from pure culture of *R. salmoninarum* collected from rainbow trout at the Colorado Parks and Wildlife Bellvue Fish Research Hatchery. The green shaded area around the linear fit represents the standard error.

The original stock of bacterial cells for the development of the standard curve was equivalent to the 4.4 x 10^7^ cells mL^-1^. The total PCR product from the serial dilutions ranged from 2.2 x 10^5^ to 2.2 x 10^1^ bacterial cells mL^-1^. Since 5 μL of each standard per qPCR reaction was used, the final five-point standard curve generated a linear dynamic range from 1.1 x 10^5^ to 1.1 x 10 bacterial cells mL^-1^. The standard curve was linear $\left(R^{2}=0.94\right)$ indicating that the number of cells was correlated with the Cq value (Fig 5). The slope of the standard curve was -3.38, which corresponds to a qPCR amplification efficiency of 97.6% (MIQE standards; [48]). We estimated the maximum Cq value from the intercept and used that estimate as the positive detection cutoff value of 37.75, corresponding to an analytical sensitivity of 1.1 bacterial cells mL^-1^. The Cq value of 37.75 is an acceptable maximum Cq value to consider tissues positive for *R. salmoninarum* using qPCR [49].

### Tissue and assay data

**Fig 6:**
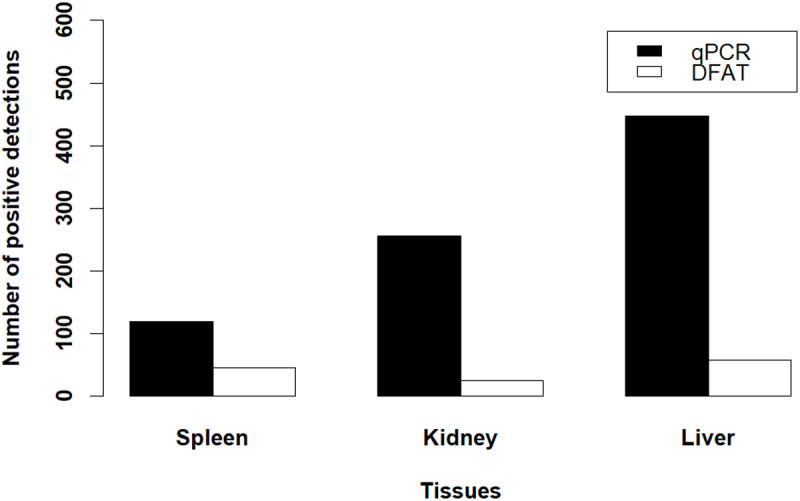
Comparison of tissue-specific detection of *Renibacterium salmoninarum* using DFAT and qPCR. Number of positive *Renibacterium salmoninarum* detections from three tissues (spleen, kidney, liver) using qPCR (black bars) and DFAT (white bars). Total number of samples for each tissue are as follows: 781 spleen samples, 778 kidney samples, and 776 liver samples.

We detected *R. salmoninarum* in 591 (75.6%) fish by any assay or tissue. The number of *R. salmoninarum* detections with DFAT were lower than detections with qPCR overall. Using DFAT, *R. salmoninarum* was detected in 108 fish (13.8% of all fish tested: 52 males; 56 females) from any tissue sample, whereas *R. salmoninarum* was detected in 566 fish (72.5%: 288 males; 278 females) with qPCR. Among the fish with detections by DFAT, bacteria were detected in only 25 kidney tissues (4.2% of all fish with detections), 58 liver tissues (9.8%), and 45 spleen tissues (7.6%) (Fig 6), whereas we had more detections in tissues with qPCR. Specifically, bacteria were detected in 256 kidney tissues (43.3% of all fish with detections), 447 liver tissues (75.6%), and 120 spleen tissues (20.3%) with qPCR (Fig 6). The occurrence of detections in multiple tissues from the same fish with the same assay was low. Among the 256 kidney tissue detections and 447 liver tissue detections by qPCR, we only detected *R. salmoninarum* in both tissues from 151 fish. Similarly, we only detected *R. salmoninarum* by DFAT in 8 kidney and liver tissues within the same fish.

### Multi-state occupancy model

Both assays were modeled independently to estimate the probability of infection for each of the four states. The AUC was greater than 0.80 for each model (DFAT: 0.90, qPCR: 0.80) indicating that the model had good discriminatory ability.

### Probability of detection

**Fig 7:**
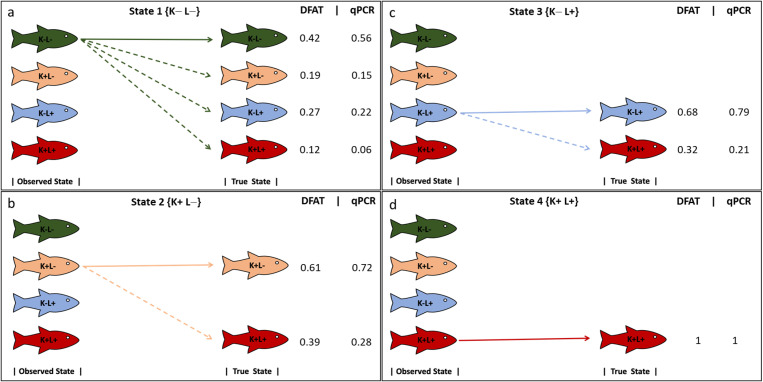
Detection probabilities of true infection states for *Renibacterium salmoninarum.* Each panel represents the probability that the observed state of infection was the true state of infection or not (i.e., the detection probability of the true state of infection) when using either DFAT or qPCR. Solid lines represent the probability of observing a fish in the true state of infection. Dashed lines indicate the probability of observing a state of infection different from the true infection state due to false negative results with DFAT and/or qPCR. (a: State 1 {K−L−}, b: State 2 {K+L−}, c: State 3 {K−L+}, d: State 4 {K+L+}).

The probability of detection varied between assay type and within each state of infection (Fig 7). In state 4, both kidney and liver were positive and we assumed the true state was the observed state because our assays were not expected to produce false positive results. Thus, detection probability with both assays for state 4 {K+L+} was 1.0 (Fig 7d). The probability of detection was low for DFAT analyses in both kidney $\left(p_{2}=0.61;CI:0.39-0.65\right)$ and liver tissues $\left(p_{3}=0.68;CI:0.58-0.81\right)$ and overlapping credible intervals suggested no differences in detection probability between the tissues. Detection probabilities indicated a high potential for false negative results when using DFAT. For instance, when the observed state of infection 1 {K−L−} was the true state, the probability of correctly detecting this state was 0.42 (CI: 0.34 − 0.53), and the probability of obtaining a false negative result with DFAT was 0.58 (CI: 0.47 − 0.66, Fig 7a). Additionally, when the observed state was state 2 {K+L−}, the probability of detection was 0.61 (CI: 0.58 − 0.65), with a probability of a false negative for liver tissues, where the true infection was not detected and should have been state 4 {K+L+}, of 0.39 (CI: 0.35 − 0.42, Fig 7b). The results were similar when the observed state was state 3 {K−L+}, with a slightly higher probability (0.68) of the observed state being the true state (CI: 0.34 − 0.81, Fig 7c), indicating a false negative probability of 0.32 (CI: 0.19 − 0.66) among kidney tissues.

Overall, the detection probability when using qPCR was higher than DFAT (Fig 7). The qPCR detection probability was 0.72 for kidney tissue $\left(p_{2};CI:0.70-0.74\right)$ and 0.79 for liver tissue $\left(p_{3};CI:0.77-0.81\right)$ and non-overlapping credible intervals suggested that liver tissue had a higher detection probability than kidney tissue. Similar to DFAT, qPCR also exhibited a high probability of false negative results. For instance, the probability of detecting state 1 {K−L−} was 0.56 (CI: 0.52 − 0.60), indicating a probability of a false negative result with qPCR of 0.44 (CI: 0.40 − 0.48; Fig 7a). The probability of detecting the true state of state 2 {K+L−} was 0.72 (CI: 0.70 − 0.74), indicating the probability of a false negative with liver tissues was 0.28 (CI: 0.26 − 0.30; Fig 7b). The results were similar when the observed state was state 3 {K−L+}, with a slightly higher probability of the observed state being the true state (0.79; CI: 0.76 − 0.82) and a probability of a false negative with kidney tissues of 0.21 (CI: 0.18 − 0.24; Fig 7c).

### Probability of infection

The estimates derived from our state process model represent the probability the fish are in a specific state of infection (Table 1). The DFAT model predicted that 653 fish (0.83; CI: 0.78 − 0.98) were negative for *R. salmoninarum* in both tissues (state 1 {K−L−}), and the probability of infection was highest in liver tissue (0.10; CI: 0.06 − 0.16) and lowest in kidney tissue (0.009; CI: < 0.001 − 0.003). Marginal credible intervals for the probability of infection for kidney and liver tissues do not overlap, suggesting that the probability of infection was higher in liver tissues. The Dirichlet prior allowed us to constrain the probability of infection $\left(\Psi_{k}\right)$ within each state such that we could calculate the marginal probability of infection for both tissues. Thus, the marginal probability of infection for liver tissue (state 3 {K−L+} and state 4 {K+L+}) was the highest, with an estimated 112 infected fish (0.16; CI: 0.11 − 0.24). The marginal probability of infection was lower for kidney tissue (state 2 {K+L−} and state 4 {K+L+}), with an estimated 38 infected fish (0.07; CI: 0.004 − 0.10). Overall, a combined (state 2, 3, and 4) 118 total fish (0.17; CI: 0.09 − 0.27) were estimated to be infected using DFAT alone.

Higher probabilities of infection were predicted from the qPCR model compared to the DFAT model and only 159 fish (0.21; CI: 0.17 − 0.25) were estimated to have no infection in either kidney or liver tissue. The probability of infection was highest in the liver tissue (0.43; CI: 0.38 − 0.43) and lowest in the kidney tissues (0.10; CI: 0.08 − 0.13). Marginal credible intervals from qPCR suggest that the probability of infection in liver tissue was greater than when testing kidney tissue, with no overlap. The marginal probability of infection for liver tissue (state 3 {K+L+} and 4 {K+L+}) estimated a total of 528 infected fish (0.68; CI: 0.60 − 0.78). The marginal probability of infection for kidney tissue estimated a total of 289 infected fish (0.37; CI: 0.30 − 0.43) predicted to be in states 2 {K+L−} and state 4 {K+L+}. Thus, the total probability of infection in both tissues, i.e., state 2, 3, and 4, was 615 total infected fish (0.79; CI: 0.68 − 0.92). Overall, the probability of infection was greatest when analyzing liver tissues with either DFAT or qPCR, and there were large discrepancies between assays.

### Diagnostic sensitivity

**Fig 8:**
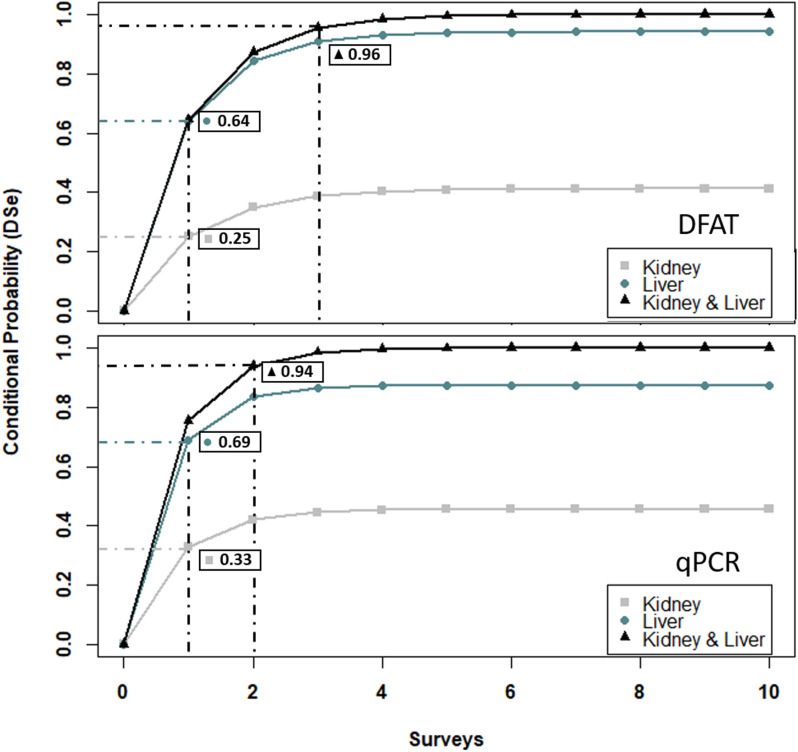
Conditional probability of detecting *Renibacterium salmoninarum* in different tissues. Conditional probability of detecting *Renibacterium salmoninarum* (DSe) in liver (blue circles), kidney (grey squares), or both tissues combined (black triangles) is shown as the function of the number of surveys conducted using either DFAT or qPCR. DSe values are indicated next to the dotted lines for cases where only one survey was conducted with kidney or liver tissue for each assay, as well as when DSe exceeds 90% (after three surveys with DFAT or two surveys with qPCR).

The calculated DSe (conditional probability) for *R. salmoninarum* given a tissue was infected was low when testing kidney tissues with DFAT (0.25; Eqn 6; Fig 8) compared to testing kidney tissues with qPCR (0.33; Eqn 6; Fig 8). The estimates of DSe were higher when testing liver tissue with both DFAT (0.64) and qPCR (0.69). Additionally, the DSe of both assays increased as the number of surveys (K in Eqn. 6; x-axis in Fig 8) conducted on the liver, kidney, or both tissues increased, reaching a plateau after 3–4 surveys. However, even when multiple surveys were conducted, the DSe for kidney tissues was never greater than 0.50 when using either DFAT or qPCR. Thus, our results indicate that to achieve a detection probability of greater than 90%, three surveys with DFAT or two surveys with qPCR (Eqn 6; Fig 8) need to be completed on individual liver tissues or a combination of liver and kidney tissues when testing cutthroat trout for *R. salmoninarum*.

## Discussion

The goals for diagnosing an infection in fish health are to use sampling and testing methods that can detect low levels of pathogens in fish tissues. The current standard for testing salmonids for *R. salmoninarum* in hatcheries and the wild is to use DFAT for initial screening and PCR (nested or quantitative) for confirmation. Together, they can be used to screen for and confirm *R. salmoninarum* presence in tissues. However, estimated detection probabilities for these two tests still vary between species and tissues, despite decades of research [22,28]. Here, we used a multistate model to estimate the probability that the observed infection state from dual kidney and liver testing with DFAT and qPCR matched the true infection state for each fish. Overall, our model estimates suggest both assays produced many false negatives (failure to detect bacteria when it was present). But qPCR had fewer false negatives than DFAT. The true infection state was often misjudged by both assays. Yet multiple surveys increased the conditional detection probability, boosting DSe in *R. salmoninarum* results with either assay.

Occupancy modeling has become increasingly popular in disease ecology to estimate pathogen prevalence while accounting for uncertainties in laboratory assays [49–52]. These models have successfully determined malaria detection probabilities using qPCR on Eurasian blue tit blood samples (*Cyanistes caeruleus*; [51]), avian influenza virus detection in waterfowl populations [49], and even bacterial kidney disease in Atlantic salmon (Laurin et al. 2018 [53]. We adapted a similar approach using multiple tissues, conceptually analogous to a single-season multistate occupancy model, which to our knowledge has not been completed before. This allowed us to estimate *R. salmoninarum* detection probabilities between DFAT and qPCR across multiple tissues in cutthroat trout.

Salmonids are the principal reservoirs of infection, and mortalities among Pacific salmon (*Oncorhynchus* spp.) and Atlantic salmon (*Salmo salar*) can reach 80% and 40%, respectively [54,55]. The AFS Fish Health Blue Book recommends sampling kidney tissue to test for *R. salmoninarum*, as kidney is often the primary site of infection of bacterial pathogens [55,56]. Despite high *R. salmoninarum* prevalence among Colorado cutthroat trout populations, we rarely observe signs of clinical disease in kidney tissues or at a population level [38], thus is it difficult to assess disease prevalence in aquaculture facilities or wild populations. We focused on testing other tissues (liver and spleen) for *R. salmoninarum* presence in cutthroat trout because the pathogen can establish in multiple organs, which may provide higher detection capabilities to assess if the population is infected with the bacteria.

Our study revealed detection of *R. salmoninarum* in liver tissue occurs more than kidney and spleen tissues (Fig 6). The kidney is pivotal for clearing bacteria from the blood through passive uptake and phagocytosis by reticuloendothelial cells [57,58]. However, the liver may play a vital role in the innate immune response in teleost fish. The kidney acts as the first line of defense through phagocytosis, and the liver removes foreign materials from the blood circulation by activation of the innate immune response [59]. Recent studies from our laboratory [60,61], have also shown that the liver may become infected before the kidney tissues after intraperitoneal injection. The liver tissue remained infected throughout the disease’s progression in the fish, suggesting that testing liver tissues may provide a higher chance of detection since the liver is one of the first tissues infected and remains infected. However, infections patterns observed across different assays and tissues may reflect various stages of infections [20] and the use of multiple assay types and tissues can lead to discrepancies in determining whether a fish are positive or negative for *R. salmoninarum* infection [26,62]. Variability in the detection of *R. salmoninarum* between DFAT and qPCR was observed across all three tissues (Fig 6), and our DSe model results (Fig 8) further emphasize this. Future studies are needed to investigate the progression and pathogenesis of *R. salmoninarum* infections to understand which tissues should be tested at various time points during the disease course.

One challenge with testing fish for pathogens is the limited knowledge about the number of times a single tissue sample must be tested to ensure a high DSe [18]. To estimate the probability that a tissue sample will test positive for a pathogen given the tissue is infected, we modeled the conditional probability of detection (a.k.a. DSe; Fig 8) using the probability of pathogen detection and probability of infection from our occupancy model (Fig 7). Compared to kidney tissue, testing liver tissue repeatedly with either qPCR or DFAT assays results in much higher DSe. Specifically, testing liver tissue with two surveys with qPCR or three surveys with DFAT led to DSe increasing to 90%. Despite up to ten surveys, testing individual kidney tissue with either assay never produced a DSe above 50% (Eqn 6; Fig 8).

Notably, standard fish health inspections use one survey with the presumptive DFAT test. Our results indicate DFAT had a surprisingly low DSe of just 25% for detecting infection in kidney tissues (Eqn 6; Fig 8). This is much lower than expected for a routine diagnostic method and indicates a high rate of false negative results. DSp for DFAT and qPCR have previously been determined as 0.85 and 1.0, respectively [22], yet are not considered a gold standard for testing due to the inconsistency among the tests. In a statewide survey to determine the prevalence and distribution of *R. salmoninarum* in Colorado, DFAT and qPCR were used to screen kidney tissues in naturally infected cutthroat trout and rainbow trout populations. Positive detections with qPCR resulted in an estimated 23.5% of populations that were infected with *R. salmoninarum*, whereas only 4.8% were determined infected with DFAT. In addition, the two assays were in agreement on infection status in only one of the populations tested [38]. In contrast, detection of *R. salmoninarum* with DFAT and qPCR in kidney tissues was near 100% in Chinook salmon [63].

The reason for the apparent low level of detection with DFAT in our study is currently unknown. Poor correlation between assays may be strongly influenced by bacterial load in tissues or by the diagnostic limitations of each assay. Other studies have shown that the analysis of kidney by two different assays (i.e., ELISA and qPCR) do not generate redundant results likely because the assays measure different bacterial macromolecules and can therefore report different states of infections [64]. Additionally, our study and others focusing on *R. salmoninarum* detection, revealed that inland trout often have low infection levels compared to many Pacific Northwest salmonids [38,39,65]. DFAT struggles to detect low level infections due to minimal fluorescence or technician oversight when few bacterial cells are present. Therefore, the low bacterial loads common in inland trout likely explain the lack of DFAT detection in our study. Given inland trout’s tendency for low-level infections, homogenizing and testing both kidney and liver tissues as one sample will likely increase the DSe (Fig 8). Alternatively, if that is not possible, collecting and testing liver rather than kidney tissue with qPCR, but testing repeatedly can increase detection of *R. salmoninarum*. Modeling DSe from detection probabilities for cheaper and less time-consuming assays such as ELISA can also be considered, especially if qPCR for all samples or multiple DFAT surveys are cost or time prohibitive.

While our Bayesian multistate occupancy model provided valuable insights into the detection probabilities of *R. salmoninarum*, there are several areas where the model’s performance could be improved. One key limitation is the restricted scope of the dataset, as it focused on cutthroat trout and did not include species with known higher infection rates, such as rainbow trout or Chinook salmon (Fig 1b), or other internal tissues (Fig 1a). To enhance the model’s robustness and applicability, it may be trained with a broader dataset that includes species exhibiting a wider range of infection dynamics across multiple populations or tissues. Additionally, other pathogens could be considered. For example, incorporating additional pathogens such as *Myxobolus cerebralis* (the causative agent of whirling disease) and alternative locations of infection within the fish could provide a more comprehensive understanding of disease dynamics when using this model.

Despite the limitations, we do not believe that the use of *R. salmoninarum* infections in cutthroat trout, as opposed to other commonly studied species like rainbow trout or Chinook salmon, jeopardized the model’s performance. Preliminary findings from our ongoing laboratory studies [66] suggest that cutthroat trout may be as susceptible to *R. salmoninarum* as Chinook salmon. In these studies, one-year-old cutthroat trout exhibited high mortality rates (98%) following injection with a genetically distinct *R. salmoninarum* strain isolated from trout in Colorado hatcheries, similar to Chinook salmon (99%; [66]). These findings support that the model accurately predicted *R. salmoninarum* infection dynamics in cutthroat trout, which are highly susceptible to the pathogen. However, training the model with other species, different *R. salmoninarum* isolates and tissues, or testing with a broader range of pathogens would help to better account for variations in infection dynamics and detection rates across fish species. These additions will enhance the model’s predictive power and its usefulness for fish health management in both hatchery and wild populations.

## Conclusion

Our work demonstrates the ongoing value of occupancy modeling in disease ecology for estimates of DSe when the infection status is unknown and when multiple tissues may be infected. Our Bayesian multistate occupancy model accounted for diagnostic uncertainty across two tests (DFAT and qPCR) to identify the best tissue and assay for detecting *R. salmoninarum* in cutthroat trout. Without a gold standard confirmation test, DSe and false negatives are hard to quantify but are important for fish health diagnostics. When the DSe of an assay for a specific species and tissue is low, false negatives increase, potentially allowing infected fish to be stocked unknowingly. Ultimately, false negatives underestimate pathogen prevalence. Using a Bayesian multistate occupancy modeling approach to estimate diagnostic sensitivity across tissues and assays, which does not require a known infection status, will overcome the lack of a gold standard and possible false negative results. By doing this we found that detection of *R. salmoninarum* with DFAT has relatively low DSe compared to qPCR for kidney and liver tissues. Continued testing for *R. salmoninarum* in fish is most effective using liver or a combination of liver and kidney with qPCR.

## Supporting information

S1 Appendix References supporting Fig 1. This appendix provides a curated list of references used to create Fig 1. Studies were identified through a comprehensive search in Web of Science and Google Scholar using specific search terms associated with bacterial kidney disease, historical references to Dee disease, *Cornyebacterium* spp., and *Renibacterium salmoninarum* research spanning from 1900 to 2021. Relevant studies were also sourced from citations within initial search results. Data extraction was conducted manually in August 2021.
(DOCX)

S1 Table Multinomial logistic regression results. Multinomial logistic regression results (z-test statistic and p-value, α = 0.05) comparing the effects of covariates (sex, length, or weight) on *Renibacterium salmoninarum* detection using either DFAT or qPCR and for each state of infection.
(DOCX)

S1 Equation Bayesian Hierarchical model for static multistate occupancy analysis of *Renibacterium salmoninarum* infection states. Model statement for the static multistate occupancy model in a Bayesian hierarchical framework.
(DOCX)

S1 Fig Model predictions of infection states probability. Values represent the probability of observing an infection state given the probability of the true state of infection for each of the assay models (DFAT top, qPCR bottom).
(DOCX)

S1 Data Data supporting the findings of these study. The data supporting the findings of the study are included. These data include the detections of *Renibacterium salmoninarum* from direct fluorescent antibody tests and quantitative polymerase chain reaction used to inform the model.
(DOCX)
