# Supplementary material for: Leveraging detection uncertainty to estimate Renibacterium salmoninarum infection status among multiple tissues and assays
Source: PLoS One. 2025 May 8;20(5):e0323010. doi: 10.1371/journal.pone.0323010 (PMC12061193; doi:10.1371/journal.pone.0323010)
Supplement: S1 Appendix — This appendix provides a curated list of references used to create Fig 1. Studies were identified through a comprehensive search in Web of Science and Google Scholar using specific search terms associated with bacterial kidney disease, historical references to Dee disease, Cornyebacterium spp., and Renibacterium salmoninarum research spanning from 1900 to 2021. Relevant studies were also sourced from citations within initial search results. Data extraction was conducted manually in August 2021. (DOCX) [file pone.0323010.s001.docx]

**S1 Appendix. References supporting Figure 1.** This appendix provides a curated list of references used to create Figure 1. Studies were identified through a comprehensive search in Web of Science and Google Scholar using specific search terms associated with bacterial kidney disease, historical references to Dee disease, Cornyebacterium spp., and R. salmoninarum research spanning from 1900 to 2021. Relevant studies were also sourced from citations within initial search results. Data extraction was conducted manually in August 2021.

1. Ajmal M, Hobbs BC. Causes and effects of columnaris-type diseases in fish: Species of Corynebacterium and Pasteurella isolated from diseased salmon, trout and rudd. Nature 1967; 215(5097): 142−143. doi: 10.1038/215141a0.
2. Alcorn S, Murray AL, Pascho RJ, Varney, J. A cohabitation challenge to compare the efficacies of vaccines for bacterial kidney disease (BKD) in Chinook salmon *Oncorhynchus tshawytscha*. Dis. Aquat. Organ 2005; 63(2-3): 151−160. doi: 10.3354/dao063151
3. Alexander SM. The molecular differentiation of *Renibacterium salmoninarum* isolates. 2002. Dissertation, University of Plymouth.
4. Armstrong RD, Martin SW, Evelyn TP, Hicks B, Dorward WJ, Ferguson HW. A field evaluation of an indirect fluorescent antibody-based broodstock screening test used to control the vertical transmission of *Renibacterium salmoninarum* in Chinook salmon (*Oncorhynchus tshawytscha*). Can. J. Vet Res. 1989; 53(4): 385. PMID: 2686828
5. Austin B, Embley TM. Goodfellow M. Selective isolation of *Renibacterium salmoninarum.*FEMS Microbiol. Lett. 1983; 17(1/3): 111−114. doi: 10.1111/j.1574-6968.1983.tb00383.x
6. Austin B, Rayment JN. Epizootiology of *Renibacterium salmoninarum*, the causal agent of bacterial kidney disease in salmonid fish. J. Fish Dis. 1985; 8(6): 505−509. doi: [10.1111/j.1365-2761.1985.tb00965.x](https://doi.org/10.1111/j.1365-2761.1985.tb00965.x)
7. Banner CR, Rohovec JS, Fryer JL. *Renibacterium salmoninarum* as a cause of mortality among Chinook salmon in salt water. J. World Maric. Soc. 1983; 14(1−4): 236−239.
8. Banner CR, Long JJ, Fryer JL, Rohovec JS. Occurrence of salmonid fish infected with *Renibacterium salmoninarum* in the Pacific Ocean. J. Fish Dis. 1986; 9(3): 273−275. doi: [10.1111/j.1365-2761.1986.tb01013.x](https://doi.org/10.1111/j.1365-2761.1986.tb01013.x)
9. Bayliss SC, Verner-Jeffreys DW, Ryder D, Suarez R, Ramirez R, Romero J, Feil EJ. Genomic epidemiology of the commercially important pathogen *Renibacterium salmoninarum* within the Chilean salmon industry. Microb. Genom. 2018; 4(9). PMID: 30040063
10. Beacham TD, Evelyn TPT. Genetic variation in disease resistance and growth of Chinook, Coho, and chum salmon with respect to vibriosis, furunculosis, and bacterial kidney disease. Trans. Am. Fish Soc. 1992; 121(4): 456−485. doi: [10.1577/1548-8659(1992)121<0456:GVIDRA>2.3.CO;2](https://doi.org/10.1577/1548-8659(1992)121%3C0456:GVIDRA%3E2.3.CO;2)
11. Beacham TD, Evelyn TPT. Population and genetic variation in resistance of Chinook salmon to vibriosis, furunculosis, and bacterial kidney disease. J. Aquati. Anim. Health 1992: 4(3): 153−167. doi: [10.1577/1548-8667(1992)004<0153:PAGVIR>2.3.CO;2](http://dx.doi.org/10.1577/1548-8667(1992)004%3C0153:PAGVIR%3E2.3.CO;2)
12. Belding DL, Merrill B. A preliminary report upon a hatchery disease of the Salmonidae. Trans. Am. Fish Soc. 1935; 65(1): 76−84.
13. Bethke J, Poblete‐Morales M, Irgang R, Yáñez A, Avendaño‐Herrera R. Iron acquisition and siderophore production in the fish pathogen *Renibacterium salmoninarum*. J. Fish Dis. 2016; 39(11): 1275−1283.
14. Bethke J, Quezada J, Poblete-Morales M, Irgang R, Yáňez A. Biochemical, serological, and genetic characterization of *Renibacterium salmoninarum* isolates recovered from salmonids in Chile. Bull. Eur. Ass. Fish Pathol. 2017; 37(4): 169-180.
15. Bland M. Epizootic investigation into the presence of bacterial kidney disease (BKD) in rainbow trout farms in Scotland 2005. Fisheries Research Services Internal Report 2007; (14/07).
16. Brenkman SJ, Mumford SL, House M, Patterson C. Establishing baseline information on the geographic distribution of fish pathogens endemic in Pacific salmonids prior to dam removal and subsequent recolonization by anadromous fish in the Elwha River, Washington. Northwest Sci., 2008; 82(1): 142−152.
17. Brown LL, Albright LJ, Evelyn TPT. Control of vertical transmission of *Renibacterium salmoninarum* by injection of antibiotics into maturing female Coho salmon *Oncorhynchus kisutch*. Dis. Aquat. Organ 1990; 9(2): 127−131. doi: [10.3354/dao009127](http://dx.doi.org/10.3354/dao009127)
18. Bruno DW. Changes in serum parameters of rainbow trout, *Salmo gairdneri* Richardson, and Atlantic salmon, *Salmo salar* L., infected with *Renibacterium salmoninarum*. J. Fish Dis. 1986; 9(3): 205−211. doi: [10.1111/j.1365-2761.1986.tb01005.x](http://dx.doi.org/10.1111/j.1365-2761.1986.tb01005.x)
19. Bruno DW, Munro ALS. Haematological assessment of rainbow trout, *Salmo gairdneri* Richardson, and Atlantic salmon, *Salmo salar* L., infected with *Renibacterium salmoninarum*. J. Fish Dis. 1986; 9(3): 195−204. doi: [10.1111/J.1365-2761.1986.TB01004.X](https://doi.org/10.1111/J.1365-2761.1986.TB01004.X)
20. Bruno DW, Munro AL. Observations on *Renibacterium salmoninarum* and the salmonid egg. Dis. Aquat. Organ. 1986; 1(2):83−87.
21. Bruno DW. Prevalence and diagnosis of bacterial kidney disease (BKD) in Scotland between 1990 and 2002. Dis. Aquat. Organ. 2004; 59(2): 125−130. doi: [10.3354/dao059125](https://doi.org/10.3354/dao059125)
22. Bullock GL, Stuckey HM. Fluorescent antibody identification and detection of the Corynebacterium causing kidney disease of salmonids. J. Fish Res. Board Can. 1975; 32(11): 2224−2227. doi:10.1139/f75-263
23. Bullock GL, Stuckey HM, Mulcahy D. Corynebacterial kidney disease: Egg transmission following iodophore disinfection. Fish Health News 1978; 7(2): 51−52.
24. Bullock GL, Griffin BR, Stuckey HM. Detection of *Corynebacterium salmoninus* by direct fluorescent antibody test. Can. J. Fish Aquat. Sci. 1980: 37(4): 719−721. doi:10.1139/f80-092
25. Burnley TA, Stryhn H, Burnley HJ, Hammell KL. Randomized clinical field trial of a bacterial kidney disease vaccine in Atlantic salmon, *Salmo salar* L. J. Fish Dis. 2010; 33(7): 545−557. PMID: 20367741
26. Campbell WB, Emlen JM. Developmental instability analysis of BKD-infected spring Chinook salmon, *Oncorhynchus tshawytscha*, prior to seawater exposure. Oikos 1996; 540−548.
27. Campos‐Perez JJ, Ward M, Grabowski PS, Ellis AE, Secombes CJ. The gills are an important site of iNOS expression in rainbow trout *Oncorhynchus mykiss* after challenge with the Gram‐positive pathogen *Renibacterium salmoninarum*. Immunol. 2000; 99(1):153−161. PMID: 10651954
28. Chambers E, Barker G. Comparison of culture media for the isolation of *Renibacterium salmoninarum* from naturally infected rainbow trout (*Oncorhynchus mykiss*). Bull. Eur. Ass. Fish Pathol. 2006; *26*(3): 137.
29. Chase DM, Pascho RJ. Development of a nested polymerase chain reaction for amplification of a sequence of the p57 gene of *Renibacterium salmoninarum* that provides a highly sensitive method for detection of the bacterium in salmonid kidney. Dis. Aquat. Organ 1998; 34(3): 223−229.
30. Chase DM, Elliott DG, Pascho RJ. Detection and quantification of *Renibacterium salmoninarum DNA* in salmonid tissues by real-time quantitative polymerase chain reaction analysis. J. Vet. Diagn. Invest. 2006; 18(4): 375−380. doi: 10.1177/104063870601800409.
31. Chen PK, Bullock GL, Stuckey HM, Bullock AC. Serological diagnosis of corynebacterial kidney disease of salmonids. J. Fish Board Can. 1974; 31(12): 1939−1940.
32. Cipriano, R. C., Starliper, C. E., & Schachte, J. H. (1985). Comparative sensitivities of diagnostic procedures used to detect bacterial kidney disease in salmonid fishes. *Journal of Wildlife Diseases*, *21*(2): 144−148. doi: 1[0.7589/0090-3558-21.2.144](https://doi.org/10.7589/0090-3558-21.2.144)
33. Cvitanich JD. Improvements in the direct fluorescent antibody technique for the detection, identification, and quantification of *Renibacterium salmoninarum* in salmonid kidney smears. J. Aquat. Anim. Health 1994; 6(1): 1−12. doi: [10.1577/1548-8667(1994)006<0001:IITDFA>2.3.CO;2](https://doi.org/10.1577/1548-8667(1994)006%3C0001:IITDFA%3E2.3.CO;2)
34. Dale OB, Gutenberger SK, Rohovec JS. Estimation of variation of virulence of *Renibacterium salmoninarum* by survival analysis of experimental infection of salmonid fish. J. Fish Dis.1997; 20(3): 177−183.
35. Dannevig BH, Lauve A, Press CM, Landsverk T. Receptor-mediated endocytosis and phagocytosis by rainbow trout head kidney sinusoidal cells. Fish Shellfish Immunol. 1994; 4(1): 3−18.
36. Del Cerro A, Márquez I, Prieto JM. Genetic diversity and antimicrobial resistance of *Flavobacterium psychrophilum* isolated from cultured rainbow trout, *Oncorhynchus mykiss* (Walbaum), in Spain. J. Fish Dis. 2010; 33(4): 285−291.
37. Delghandi MR, Menanteau-Ledouble S, Waldner K, El-Matbouli M. *Renibacterium salmoninarum* and Mycobacterium spp.: Two bacterial pathogens present at low levels in wild brown trout (*Salmo trutta fario*) populations in Austrian rivers. BMC Vet. Res. 2020; 16: 1−12. doi: 10.1186/s12917-020-2260-7.
38. Densmore CL, Smith SA, Holladay SD. In vitro effects of the extracellular protein of *Renibacterium salmoninarum* on phagocyte function in brook trout (*Salvelinus fontinalis*). Vet. Immunol. Immunopathol. 1998; 62(4): 349−357. doi: [10.1016/s0165-2427(98)00101-9](https://doi.org/10.1016/s0165-2427(98)00101-9)
39. Eissa AE. Bacterial kidney disease (BKD) in Michigan salmonids. Michigan State University 2005.
40. Elliott DG, Pascho RJ, Bullock GL. Developments in the control of bacterial kidney disease of salmonid fishes. Dis. Aquat. Organ 1989; 6(3): 201−215. doi: [10.3354/DAO006201](https://doi.org/10.3354/DAO006201)
41. Elliott DG, Pascho RJ. Juvenile fish transportation: impact of bacterial kidney disease on survival of spring/summer Chinook salmon stocks, 1989. US Army Corps of Engineers, Walla Walla District; 1991.
42. Elliott DG, Pascho RJ, Palmisano AN. Brood stock segregation for the control of bacterial kidney disease can affect mortality of progeny Chinook salmon (*Oncorhynchus tshawytscha*) in seawater. Aquac. 1995; 132(1-2): 133−144. doi: [10.1016/0044-8486(94)00380-7](https://doi.org/10.1016/0044-8486(94)00380-7)
43. Elliott DG, McKibben CL. Comparison of two fluorescent antibody techniques (FATs) for detection and quantification of *Renibacterium salmoninarum* in coelomic fluid of spawning Chinook salmon *Oncorhynchus tshawytscha*. Dis. Aquat. Organ. 1997; 30(1): 37−43. doi: 10.3354/dao030037.
44. Eslamloo K, Kumar S, Caballero-Solares A, Gnanagobal H, Santander J, Rise ML. Profiling the transcriptome response of Atlantic salmon head kidney to formalin-killed *Renibacterium salmoninarum*. Fish Shellfish Immunol. 2020; 98: 937−949. doi: [10.1016/j.fsi.2019.11.057](https://doi.org/10.1016/j.fsi.2019.11.057)
45. Evelyn TPT. The agglutinin response in sockeye salmon vaccinated intraperitoneally with a heat-killed preparation of the bacterium responsible for salmonid kidney disease. J. Wildl. Dis. 1971; 7(4):328−335. doi: 10.7589/0090-3558-7.4.328
46. Evelyn TPT, Hoskins GE, Bell GR. First record of bacterial kidney disease in an apparently wild salmonid in British Columbia. J. Fish Board Can. 1973; 30(10): 1578−1580. doi: 10.1139/f73-249.
47. Evelyn TPT, Ketcheson JE, Prosperi-Porta L. The clinical significance of immunofluorescence-based diagnoses of the bacterial kidney disease carrier. Fish Pathol. 1981; 15(3-4): 293−300. doi: [10.3147/jsfp.15.293](https://doi.org/10.3147/jsfp.15.293)
48. Evelyn TPT, Ketcheson JE, Prosperi‐Porta L. Further evidence for the presence of *Renibacterium salmoninarum* in salmonid eggs and for the failure of povidone‐iodine to reduce the intra‐ovum infection rate in water‐hardened eggs. J. Fish Dis. 1984; 7(3): 173−182. doi: [10.1111/j.1365-2761.1984.tb00921.x](https://doi.org/10.1111/j.1365-2761.1984.tb00921.x)
49. Evelyn TPT, Ketcheson JE, Prosperi-Porta L. Use of erythromycin as a means of preventing vertical transmission of *Renibacterium salmoninarum*. Dis. Aquat. Organ 1986; 2(1): 7−11.
50. Evelyn TPT, Prosperi‐Porta L, Ketcheson JE. Persistence of the kidney‐disease bacterium, *Renibacterium salmoninarum*, in Coho salmon*, Oncorhynchus kisutch* (Walbaum), eggs treated during and after water‐hardening with povidone‐iodine. J. Fish Dis. 1986; 9(5): 461−464. doi: [10.1111/j.1365-2761.1986.tb01040.x](http://dx.doi.org/10.1111/j.1365-2761.1986.tb01040.x)
51. Evelyn TPT, Bell GR, Prosperi-Porta L, Ketcheson JE. A simple technique for accelerating the growth of the kidney disease bacterium *Renibacterium salmoninarum* on a commonly used culture medium (KDM2). Dis. Aquat. Organ 1989; 7(23): 231−234. doi: [10.3354/dao007231](http://dx.doi.org/10.3354/dao007231)
52. Faisal M, Schulz C, Eissa A, Brenden T, Winters A, Whelan G, VanAmberg J. Epidemiological investigation of *Renibacterium salmoninarum* in three *Oncorhynchus* spp. in Michigan from 2001 to 2010. Prev. Vet. Med. 2012; 107(3−4): 260−274. doi: [10.1016/j.prevetmed.2012.06.003](https://doi.org/10.1016/j.prevetmed.2012.06.003)
53. Ferguson HW. Renal portal phagocytosis of bacteria in rainbow trout (*Salmo gairdneri* Richardson): Ultrastructural observations. Can. J. Zool. 1984; 62(12): 2505−2511. doi: [10.1139/z84-367](https://doi.org/10.1139/z84-367)
54. Fetherman ER, Neuschwanger B, Davis T, Wells CL, Kraft A. Efficacy of erymicin 200 injections for reducing *Renibacterium salmoninarum* and controlling vertical transmission in an inland rainbow trout brood stock. Pathogens. 2020; 9(7): 547. doi: 10.3390/pathogens9070547.
55. Fredriksen Å, Endresen C, Wergeland HI. Immunosuppressive effect of a low molecular weight surface protein from *Renibacterium salmoninarum* on lymphocytes from Atlantic salmon (*Salmo salar* L.). Fish Shellfish Immunol. 1997; 7(4): 273−282.
56. Grandón M, Irgang R, Saavedra J, Mancilla M, Avendaño‐Herrera R. Proposed protocol for performing MIC testing to determine the antimicrobial susceptibility of *Renibacterium salmoninarum* in Chilean salmon farms. J. Fish Dis. 2021; 44(3): 287−296. doi: 10.1111/jfd.13281
57. Griffiths SG, Melville KJ, Salonius K. Reduction of *Renibacterium salmoninarum* culture activity in Atlantic salmon following vaccination with avirulent strains. Fish Shellfish Immunol. 1998; 8(8): 607−619. doi: [10.1006/fsim.1998.0169](https://doi.org/10.1006/fsim.1998.0169)
58. Griffiths SG, Lynch WH. Instability of the major soluble antigen produced by *Renibacterium salmoninarum*. J. Fish Dis. 1991; 14(1): 55−66. doi: [10.1111/j.1365-2761.1991.tb00576.x](https://doi.org/10.1111/j.1365-2761.1991.tb00576.x)
59. Griffiths SG, Olivier G, Fildes J, Lynch WH. Comparison of western blot, direct fluorescent antibody and drop-plate culture methods for the detection of *Renibacterium salmoninarum* in Atlantic salmon (*Salmo salar* L.). Aquac. 1991; 97(2−3): 117−129. doi:[10.1016/0044-8486(91)90259-A](https://ui.adsabs.harvard.edu/link_gateway/1991Aquac..97..117G/doi:10.1016/0044-8486(91)90259-A)
60. Groman DB, Klontz GW. Chemotherapy and prophylaxis of bacterial kidney disease with erythromycin. J. World Maric. Soc. 1983; 14(1−4): 226−235. doi: [10.1111/j.1749-7345.1983.tb00078.x](http://dx.doi.org/10.1111/j.1749-7345.1983.tb00078.x)
61. Gudmundsdóttir S, Helgason S, Benediktsdottir E. Comparison of the effectiveness of three different growth media for primary isolation of *Renibacterium salmoninarum* from Atlantic salmon, *Salmo salar L*., broodfish. J. Fish Dis. 1991; 14(1): 89−96. doi: 10.1111/j.1365-2761.1991.tb00579.x.
62. Gudmundsdóttir S, Applegate LJ, Árnason ÍÖ, Kristmundsson Á, Purcell MK, Elliott DG. Detecting *Renibacterium salmoninarum* in wild brown trout by use of multiple organ samples and diagnostic methods. Bull. Euro. Ass. Fish Pathol. 2017; 37: 31−40.
63. Gutenberger SK. Phylogeny and intracellular survival of *Renibacterium salmoninarum*. Thesis. 1993.
64. Guz L, Puk K. Molecular detection of in *Renibacterium salmoninarum* in rainbow trout (*Oncorhynchus mykiss*) from Poland. Fish. Aquat. Life. 2020; 28(4): 234−237. doi: [10.2478/aopf-2020-0028](https://doi.org/10.2478/aopf-2020-0028)
65. Hall LM, Duguid S, Wallace IS, Murray AG. Estimating the prevalence of *Renibacterium salmoninarum*‐infected salmonid production sites. J. Fish Dis. 2015; 38(2): 231−235. doi: [10.1111/jfd.12234](https://doi.org/10.1111/jfd.12234)
66. Hard JJ, Elliott DG, Pascho RJ, Chase DM, Park LK, Winton JR, Campton DE. Genetic effects of ELISA-based segregation for control of bacterial kidney disease in Chinook salmon (*Oncorhynchus tshawytscha*). Can. J. Fish Aquat. Sci. 2006; 63(12): 2793−2808. doi: [10.1139/F06-163](https://doi.org/10.1139/F06-163)
67. Hardie LJ, Ellis AE, Secombes CJ. In vitro activation of rainbow trout macrophages stimulates inhibition of *Renibacterium salmoninarum* growth concomitant with augmented generation of respiratory burst products. Dis. Aquat. Organ 1996; 25(3): 175−183.
68. Hariharan H, Qian B, Despres B, Kibenge FS, Heaney SB, Rainnie DJ. Development of a specific biotinylated DNA probe for the detection of *Renibacterium salmoninarum*. Can J. Vet. Res. 1995; 59(4): 306.
69. Haukenes AH, Moffitt CM. Hatchery evaluation of erythromycin phosphate injections in prespawning spring Chinook salmon. N. Am. J. Aquac. 2002; 64(3): 167−174. doi: [10.1577/1548-8454](http://dx.doi.org/10.1577/1548-8454%282002%29064%3C0167%3AHEOEPI%3E2.0.CO%3B2)
70. Hendricks JD, Leek SL. Kidney disease postorbital lesions in spring Chinook salmon (*Oncorhynchus tshawytscha*). Trans. Am. Fish. Soc. 1975; 104(4): 805−807. doi: [10.1577/1548-8659(1975)104<805:KDPLIS>2.0.CO;2](https://doi.org/10.1577/1548-8659(1975)104%3C805:KDPLIS%3E2.0.CO;2)
71. Hirvelä-Koski V. *Renibacterium salmoninarum*: Effect of hypochlorite treatment, and survival in water. Dis. Aquat. Organ 2004; 59(1): 27−33. doi: [10.3354/dao059027](https://doi.org/10.3354/dao059027)
72. Hoffmann RW, Bell GR, Pfeil-Putzien C, Ogawa M. Detection of *Renibacterium salmoninarum* in tissue sections by different methods—a comparative study with special regard to the indirect immunohistochemical peroxidase technique. Fish Pathol. 1989; 24(2): 101−104.
73. Hsu HM, Bowser PR, Schachte Jr JH. Development and evaluation of a monoclonal-antibody-based enzyme-linked immunosorbent assay for the diagnosis of *Renibacterium salmoninarum* infection. J. Aquat. Anim. Health 1991; 3(3): 168−175. doi: [10.1577/1548-8667(1991)003<0168:DAEOAM>2.3.CO;2](https://doi.org/10.1577/1548-8667(1991)003%3C0168:DAEOAM%3E2.3.CO;2)
74. Hsu HM, Wooster GA, Bowser PR. Efficacy of enrofloxacin for the treatment of salmonids with bacterial kidney disease, caused by *Renibacterium salmoninarum*. J. Aquat. Anim. Health 1994; 6(3): 220−223. doi: [10.1577/1548-8667(1994)006<0220:EOEFTT>2.3.CO;2](https://doi.org/10.1577/1548-8667(1994)006%3C0220:EOEFTT%3E2.3.CO;2)
75. Iwama GK, Greek GL, Randall DJ. Changes in selected haematological parameters in juvenile Chinook salmon subjected to a bacterial challenge and a toxicant. J. Fish. Biol. 1986; 28(5): 563−572. doi: [0.1111/j.1095-8649.1986.tb05193.x](https://doi.org/10.1111/j.1095-8649.1986.tb05193.x)
76. Jansson E. Hongslo T, Höglund J, Ljungberg O. Comparative evaluation of bacterial culture and two ELISA techniques for the detection of *Renibacterium salmoninarum* antigens in salmonid kidney tissues. Dis. Aquat. Organ 1996; 27(3): 197−206.
77. Jansson E, Ljungberg O. Detection of humoral antibodies to *Renibacterium salmoninarum* in rainbow trout *Oncorhynchus mykiss* and Atlantic salmon *Salmo salar* challenged by immersion and in naturally infected populations. Dis. Aquat. Organ 1998; 33(2): 93−99. doi: [10.3354/dao027197](http://dx.doi.org/10.3354/dao027197)
78. Jonas JL, Schneeberger PJ, Clapp DF, Wolgamood M, Wright G, Lasee B. Presence of the BKD-causing bacterium *Renibacterium salmoninarum* in Lake whitefish and bloaters in the Laurentian Great Lakes. Adv. Limnol. 2002; 57: 447−452.
79. Jones DT, Moffitt CM, Peters KK. Temperature-mediated differences in bacterial kidney disease expression and survival in *Renibacterium salmoninarum*-challenged bull trout and other salmonids. N. Am. J. Fish Manag. 2007; 27(2): 695−706. doi: [10.1577/M06-002.1](https://doi.org/10.1577/M06-002.1)
80. Kent ML, Poppe TT. Diseases of seawater netpen-reared salmonid fishes. Pacific Biological Station, Nanaimo. British Columbia, Canada. 1998.
81. Kimura N, Wakabayashi H, Kudo S. Studies on bacterial gill disease in salmonids— Selection of bacterium transmitting gill disease. Fish Pathol. 1978; 12(4): 233−242.
82. Kimura T, Yoshimizu M. Rapid method for detection of bacterial kidney disease of salmonid (BKD) by coagglutination of antibody sensitized protein-A-containing staphylococci. Bull. Jap. Soc. Sci. Fish. 1981.
83. Königsson MH, Ballagi A, Jansson E, Johansson KE. Detection of *Renibacterium salmoninarum* in tissue samples by sequence capture and fluorescent PCR based on the 16S rRNA gene. Vet Microbiol. 2005; 105(3−4): 235−243. doi: [10.1016/j.vetmic.2004.11.007](https://doi.org/10.1016/j.vetmic.2004.11.007)
84. Lall SP, Paterson WD, Hines JA, Adams NJ. Control of bacterial kidney disease in Atlantic salmon, *Salmo salar* L., by dietary modification. J. Fish Dis. 1985; 8(1): 113−124. doi: [10.1111/j.1365-2761.1985.tb01192.x](https://doi.org/10.1111/j.1365-2761.1985.tb01192.x)
85. Lee EGH, Gordon MR. Immunofluorescence screening of *Renibacterium salmoninarum* in the tissues and eggs of farmed Chinook salmon spawners. Aquac. 1987; 65(1): 7−14. doi:[10.1016/0044-8486(87)90266-3](https://ui.adsabs.harvard.edu/link_gateway/1987Aquac..65....7L/doi:10.1016/0044-8486(87)90266-3)
86. Lee EGH. Technique for enumeration of *Renibacterium salmoninarum* in fish kidney tissues. J. Aquat. Anim. Health 1989; 1(1): 25−28.
87. Lee EGH, Evelyn TPT. Effect of *Renibacterium salmoninarum* levels in the ovarian fluid of spawning Chinook salmon on the prevalence of the pathogen in their eggs and progeny. Dis. Aquat. Organ 1989; 7(3): 179−184.
88. Leggatt RA, Biagi CA, Sakhrani D, Dominelli R, Eliason EJ, Farrell AP, Devlin RH. Fitness component assessments of wild-type and growth hormone transgenic Coho salmon reared in seawater mesocosms. Aquac. 2017; 473: 31−42. doi: [10.1016/j.aquaculture.2017.01.022](https://doi.org/10.1016/j.aquaculture.2017.01.022)
89. León G, Maulén N, Figueroa J, Villanueva J, Rodríguez C, Vera MI, Krauskopf M. A PCR-based assay for the identification of the fish pathogen *Renibacterium salmoninarum*. FEMS Microbiol. Lett. 1994; 115(2−3): 131−136. doi: [10.1111/j.1574-6968.1994.tb06627.x](https://doi.org/10.1111/j.1574-6968.1994.tb06627.x)
90. Lovely JE, Cabo C, Griffiths SG, Lynch WH. Detection of *Renibacterium salmoninarum* infection in asymptomatic Atlantic salmon. J. Aquat. Anim. Health 1994; 6(2), 126−132. doi: 10.1577/1548-8667(1994)006<0126:DORSII>2.3.CO;2
91. Maclean DG, Yoder WG. Kidney disease among Michigan salmon in 1967. Prog. Fish C. 1970; *32*(1): 26−30.
92. Madsen L, Dalsgaard I, Barnes AC, Davidson GA, Hiney MP, McIntosh D. Methodology in fish diseases research. 1998.
93. Matsui T, Nishizawa T, Yoshimizu M. Modification of KDM-2 with culture-spent medium for isolation of *Renibacterium salmoninarum*. Fish Pathol. 2009; 44(3), 139−144. doi: [10.3147/jsfp.44.139](https://doi.org/10.3147/jsfp.44.139)
94. Mattsso, JG, Gerdorf H, Jansson E, Hongslo T, Göbel UB, Johansson KE. Rapid identification of *Renibacterium salmoninarum* using an oligonucleotide probe complementary to 16S rRNA. Mol. Cell Probes. 1993; 7(1): 25−33. doi: [10.1006/mcpr.1993.1004](https://doi.org/10.1006/mcpr.1993.1004)
95. Maule AG, Rondorf DW, Beeman J, Haner P. Incidence of *Renibacterium salmoninarum* infections in juvenile hatchery spring Chinook salmon in the Columbia and Snake rivers. J. Aquat. Anim. Health 1996; 8(1), 37−46. doi: [10.1577/1548-8667(1996)008<0037:IORSII>2.3.CO;2](https://doi.org/10.1577/1548-8667(1996)008%3C0037:IORSII%3E2.3.CO;2)
96. McCarthy, D. H., Croy, T. R., & Amend, D. F. (1984). Immunization of rainbow trout, *Salmo gairdneri* Richardson, against bacterial kidney disease: Preliminary efficacy evaluation. *Journal of Fish Diseases*, 1984; 7(1): 65−71.
97. McIntosh D, Flano E, Grayson TH, Gilpin ML, Austin B, Villena AJ. Production of putative virulence factors by *Renibacterium salmoninarum* grown in cell culture. Microbiol. 1997; 143(10): 3349−3356. doi: [10.1099/00221287-143-10-3349](https://doi.org/10.1099/00221287-143-10-3349)
98. McIntosh D, Austin B, Fla E, Villena A, Matinez‐Pereda JA, Tarazona J. Lack of uptake of *Renibacterium salmoninarum* by gill epithelia of rainbow trout. J. Fish Biol. 2000; 56(5): 1053−1061. doi: [10.1111/j.1095-8649.2000.tb02122.x](https://doi.org/10.1111/j.1095-8649.2000.tb02122.x)
99. Metzger DC, Elliott DG, Wargo A, Park LK, Purcell MK. Pathological and immunological responses associated with differential survival of Chinook salmon following *Renibacterium salmoninarum* challenge. Dis. Aquat. Organ 2010; 90(1): 31-41. doi: [10.3354/dao02214](https://doi.org/10.3354/dao02214)

100. Meyers TR, Short S, Lipson K, Farrington C. ELISA for the detection of antigen of *Renibacterium salmoninarum* (Rs) in fish tissues. Fish Pathology Section Laboratory Manual 2000; 9.

101. Miriam A, Griffiths SG, Lovely JE, Lynch WH. PCR and probe-PCR assays to monitor broodstock Atlantic salmon (*Salmo salar* L.) ovarian fluid and kidney tissue for presence of DNA of the fish pathogen *Renibacterium salmoninarum*. J. Clin. Microbiol. 1997; 35(6):1322−1326. doi: 10.1128/jcm.35.6.1322-1326.1997.

102. Mitchum DL, Sherman LE, Baxter GT. Bacterial kidney disease in feral populations of brook trout (*Salvelinus fontinalis*), brown trout (*Salmo trutta*), and rainbow trout (*Salmo gairdneri*). J. Fish Board Can. 1979; 36(11): 1370−1376. doi: [10.1139/f79-196](http://dx.doi.org/10.1139/f79-196)

103. Mitchum DL, Sherman LE. Transmission of bacterial kidney disease from wild to stocked hatchery trout. Can. J. Fish Aquat. Sci. 1981; 38(5): 547−551. doi: 10.1139/f81- 077

104. Moles A. Effect of bacterial kidney disease on saltwater adaptation of Coho salmon smolts. J. Aquat. Anim. Health 1997; 9(3): 230−233. doi: [10.1577/1548- 8667(1997)009<0230:EOBKDO>2.3.CO;2](https://doi.org/10.1577/1548-8667(1997)009%3C0230:EOBKDO%3E2.3.CO;2)

105. Moffitt CM, Bjornn TC. Protection of Chinook salmon smolts with oral doses of erythromycin against acute challenges of *Renibacterium salmoninarum*. J. Aquat. Anim. Health 1989; 1(3): 227−232.

106. Moffitt CM. Survival of juvenile Chinook salmon challenged with *Renibacterium salmoninarum* and administered oral doses of erythromycin thiocyanate for different durations. J. Aquat. Anim. Health 1992; 4(2): 119−125. doi: 10.1577/1548- 8667(1992)004&lt;0119:SOJCSC&gt;2.3.CO;2

107. Murray AG, Soje J. Epidemiology and economics of bacterial kidney disease to inform control policy. Fish Vet. J. 2012; 13: 31−35.

108. Murray AG. Implications of leaky boundaries for compartmentalized control of pathogens: A modelling case study for bacterial kidney disease in Scottish salmon aquaculture. Ecol. Modell. 2013; 250: 177−182. doi: 10.1016/j.ecolmodel.2012.11.004

109. Nilsen H, Jensen BB, Sunde EB, Rørvik S. The surveillance and control programme for bacterial kidney disease (BKD) in Norway. Annual report 2011. Norwegian Veterinary Institute. 2012.

110. O’Connor G, Hoffnagle TL. Use of ELISA to monitor bacterial kidney disease in naturally spawning Chinook salmon. Dis. Aquat. Organ 2007; 77(2): 137−142. doi: [10.3354/dao01839](https://doi.org/10.3354/dao01839)

111. O’Farrell CL, Strom MS. Differential expression of the virulence-associated protein p57 and characterization of its duplicated gene msa in virulent and attenuated strains of *Renibacterium salmoninarum*. Dis. Aquat. Organ 1999; 38: 11−123. doi: [10.3354/dao038115](https://doi.org/10.3354/dao038115)

112. O'Halloran J, Coombs K, Carpenter E, Whitman K, Johnson G. Prescreening Atlantic salmon, *Salmo salar* L., broodstock for the presence of *Renibacterium salmoninarum* by indirect fluorescent antibody testing. J. Fish Dis. 1995; 18(1): 83−85. doi: [10.1111/j.1365-2761.1995.tb01269.x](https://doi.org/10.1111/j.1365-2761.1995.tb01269.x)

113. Olivier G, Griffiths SG, Fildes J, Lynch WH. The use of Western blot and electroimmunotransfer blot assays to monitor bacterial kidney disease in experimentally challenged Atlantic salmon, *Salmo salar* L. J. Fish Dis. 1992; 15(3): 229−241. doi: [10.1111/j.1365-2761.1992.tb00659.x](https://ui.adsabs.harvard.edu/link_gateway/1992JFDis..15..229O/doi:10.1111/j.1365-2761.1992.tb00659.x)

114. Ordal ET, Earp BJ. Cultivation and transmission of etiological agent of kidney disease in salmonid fishes. Proc. Soc. Exp. Biol. Med. 1956; 92(1): 85−88. doi: [10.3181/00379727- 92-22392](https://doi.org/10.3181/00379727-92-22392)

115. Paclibare JO, Evelyn TPT, Albright LJ. A comparative evaluation of various methods for detection of the kidney disease bacterium *Renibacterium salmoninarum* in salmonids. Bull. Aquac. Assoc. Can. 1988; 88: 110−112.

116. Paclibare JO. Aspects of the epizootiology of bacterial kidney disease in salmon farms in British Columbia. Thesis. Simon Fraser University. 1989.

117. Pascho RJ, Elliott DG, Mallett RW, Mulcahy D. Comparison of five techniques for the detection of *Renibacterium salmoninarum* in adult Coho salmon. Trans. Am. Fish. Soc. 1987; 116(6): 882−890. doi: [10.1577/1548-8659(1987)116<882:COFTFT>2.0.CO;2](https://doi.org/10.1577/1548-8659(1987)116%3C882:COFTFT%3E2.0.CO;2)

118. Pascho, R. J., & Mulcahy, D. Enzyme-linked immunosorbent assay for a soluble antigen of *Renibacterium salmoninarum*, the causative agent of salmonid bacterial kidney disease. Can. J. Fish. Aquat. Sci. 1987; 44(1): 183−191. doi: [10.1139/f87-024](https://doi.org/10.1139/f87-024)

119. Pascho RJ, Elliott DG, Streufert JM. (1991). Brood stock segregation of spring Chinook salmon *Oncorhynchus tshawytscha* by use of the enzyme linked immunosorbent assay (ELISA) and fluorescent antibody technique (FAT) affects the prevalence and levels of *Renibacterium salmoninarum* infection in progeny. Dis. Aquat. Organ. 1991;12: 25–40. doi: 10.3354/dao012025.

120. Pascho RJ, Elliott DG, Achord S. Monitoring of the in‐river migration of smolts from two groups of spring Chinook salmon, *Oncorhynchus tshawytscha* (Walbaum), with different profiles of *Renibacterium salmoninarum* infection. Aquac. Res. 1993; 24(2):, 163−169. doi: [10.1111/j.1365-2109.1993.tb00537.x](https://doi.org/10.1111/j.1365-2109.1993.tb00537.x)

121. Pascho RJ, Chase D, McKibben CL. Comparison of the membrane-filtration fluorescent antibody test, the enzyme-linked immunosorbent assay, and the polymerase chain reaction to detect *Renibacterium salmoninarum* in salmonid ovarian fluid*.* J. Vet. Diagn. Invest. 1998; 10(1): 60−66. doi: 10.1177/104063879801000111.

122. Paterson WD, Gallant C, Desautels D, Marshall L. Detection of bacterial kidney disease in wild salmonids in the Margaree River system and adjacent waters using an indirect fluorescent antibody technique. J. Fish Board Can. 1979; 36(12): 1464−1468. doi: [10.1139/f79-21](https://doi.org/10.1139/f79-213)

123. Paterson WD, Desautels D, Weber JM. The immune response of Atlantic salmon, *Salmo salar* L., to the causative agent of bacterial kidney disease, *Renibacterium salmoninarum*. J. Fish Dis. 1981; 4(2): 99−111. doi: [10.1111/j.1365- 2761.1981.tb01115.x](https://doi.org/10.1111/j.1365-2761.1981.tb01115.x)

124. Paterson WD, Lall SP, Desautels D. Studies on bacterial kidney disease in Atlantic salmon (*Salmo salar*) in Canada. Fish Pathol. 1981; 15(3−4): 283−292.

125. Pedersen K, Skall HF, Lassen‐Nielsen AM, Nielsen TF, Henriksen NH, Olesen NJ. Surveillance of health status on eight marine Rainbow trout, *Oncorhynchus mykiss* (Walbaum), farms in Denmark in 2006. J. Fish Dis. 2008; 31(9): 659−667. doi: [10.1111/j.1365-2761.2008.00941.x](https://doi.org/10.1111/j.1365-2761.2008.00941.x)

126. Peters KK, Moffitt CM. Optimal dosage of erythromycin thiocyanate in a new feed additive to control bacterial kidney disease. J. Aquat. Anim. Health 1996; 8(3): 229−240.

127. Pfeil-Putzien C, Hoffmann R, Popp W. Preliminary report on the occurrence of bacterial kidney disease in Germany. Bull. Euro. Ass. Fish Pathol. 1985; 5(2): 30−31. doi: [10.1111/j.1365-2761.2007.00868.x](https://doi.org/10.1111/j.1365-2761.2007.00868.x)

128. Piganelli JD, Wiens GD, Kaattari SL. Elevated temperature treatment as a novel method for decreasing p57 on the cell surface of *Renibacterium salmoninarum*. Dis. Aquat. Anim. 1999; 36(1): 29−35. doi: [10.3354/dao036029](https://doi.org/10.3354/dao036029)

129. Pippy JHC. Kidney disease in juvenile Atlantic salmon (*Salmo salar*) in the Margaree River. J. Fish Res. Board Can. 1969; 26(9): 2535−2537. doi: [10.1139/f69-247](https://www.sciencegate.app/app/redirect#aHR0cHM6Ly9keC5kb2kub3JnLzEwLjExMzkvZjY5LTI0Nw==)

130. Rhodes LD, Nilsson WB, Strom MS. Sensitive detection of *Renibacterium salmoninarum* in whole fry, blood, and other tissues of Pacific salmon by reverse transcription- polymerase chain reaction. Mol. Mar. Biol. Biotechnol. 1998; 7(4): 270−279. PMID: 9892717

131. Rhodes LD, Coady AM, Deinhard RK. Identification of a third msa gene in *Renibacterium salmoninarum* and the associated virulence phenotype. Appl. Environ. Microbiol. 2004; 70(11): 6488−6494. doi: [10.1128/AEM.70.11.6488-6494.2004](https://doi.org/10.1128%2FAEM.70.11.6488-6494.2004)

132. Rhodes LD, Rathbone CK, Corbett SC, Harrell LW, Strom MS. Efficacy of cellular vaccines and genetic adjuvants against bacterial kidney disease in Chinook salmon (*Oncorhynchus tshawytscha).* Fish Shellfish Immunol. 2004; 16(4): 461−474. doi: [10.1016/j.fsi.2003.08.004](https://doi.org/10.1016/j.fsi.2003.08.004)

133. Rhodes LD, Durkin C, Nance SL, Rice CA. Prevalence and analysis of *Renibacterium salmoninarum* infection among juvenile Chinook salmon *Oncorhynchus tshawytscha* in North Puget Sound. Dis. Aquat. Organ 2006; 71(3): 179−190. doi: [10.3354/dao071179](http://dx.doi.org/10.3354/dao071179)

134. Rhodes LD, Nguyen OT, Deinhard RK, White TM, Harrell LW, Roberts MC. Characterization of *Renibacterium salmoninarum* with reduced susceptibility to macrolide antibiotics by a standardized antibiotic susceptibility test. Dis. Aquat. Organ 2008; 80(3): 173−180. doi: [10.3354/dao01959](https://doi.org/10.3354/dao01959)

135. Rhodes LD, Wallis S, Demlow SE. Genes associated with an effective host response by Chinook salmon to *Renibacterium salmoninarum*. Dev. Comp. Immunol. 2009; 33(2): 176−186. doi: 1[0.1016/j.dci.2008.08.006](https://doi.org/10.1016/j.dci.2008.08.006)

136. Rhodes LD, Rice CA, Greene CM, Teel DJ, Nance SL, Moran P, Gezhegne SB. Nearshore ecosystem predictors of a bacterial infection in juvenile Chinook salmon. Mar. Ecol. Prog. Ser. 2011; 432: 161−172. doi: [10.3354/meps09160](http://dx.doi.org/10.3354/meps09160)

137. Richards CA, Murphy CA, Brenden TO, Loch TP, Faisal M. Detection accuracy of *Renibacterium salmoninarum* in Chinook salmon, *Oncorhynchus tshawytscha* (Walbaum) from non-lethally collected samples: Effects of exposure route and disease severity. Prev. Vet. Med. 2017; 145: 110−120. doi: [10.1016/j.prevetmed.2017.06.001](http://dx.doi.org/10.1016/j.prevetmed.2017.06.001)

138. Riepe TB, Vincent V, Milano V, Fetherman ER, Winkelman DL. Evidence for the use of mucus swabs to detect *Renibacterium salmoninarum* in brook trout. Pathogens 2021; 10(4): 460. doi: [10.3390/pathogens10040460](https://doi.org/10.3390/pathogens10040460)

139. Rimaila-Pärnänen E. First case of bacterial kidney disease (BKD) in whitefish (*Coregonus lavaretus*) in Finland. Bull. Euro. Ass. Fish Pathol. 2002; 22(6): 403−404.

140. Rockey DD, Gilkey LL, Wiens GD, Kaattari SL. Monoclonal antibody-based analysis of the *Renibacterium salmoninarum* p57 protein in spawning Chinook and Coho salmon. J. Aquat. Anim. Health 1991; 3(1): 23−30.

141. Rockey DD, Turaga PD, Wiens GD, Cook BA, Kaattari SL. Serine proteinase of *Renibacterium salmoninarum* digests a major autologous extracellular and cell-surface protein. Can. J. Microbiol. 1991; 37(10): 758−763. doi: [10.1139/m91-130](https://doi.org/10.1139/m91-130)

142. Rozas-Serri M, Lobos C, Correa R, Ildefonso R, Vásquez J, Muñoz A, Schwerter F. Atlantic salmon pre-smolt survivors of *Renibacterium salmoninarum* infection show inhibited cell-mediated adaptive immune response and a higher risk of death during the late stage of infection at lower water temperatures. Front. Immunol. 2020; 11: 1378. doi: [10.3389/fimmu.2020.01378](https://doi.org/10.3389/fimmu.2020.01378)

143. Sakai M, Atsuta S, Kobayashi M. Attempted vaccination of rainbow trout *Oncorhynchus mykiss* against bacterial kidney disease. Bull. Japan. Soc. Sci. Fish 1989; 55(12): 2105−2109.

144. Sakai M, Atsuta S, Kobayashi M. Comparison of methods used to detect *Renibacterium salmoninarum*, the causative agent of bacterial kidney disease. J. Aquat. Anim. Health 1989; 1(1): 21−24.

145. Sakai M, Ogasawara K, Atsuta S, Kobayashi M. Comparative sensitivity of carp, *Cyprinus carpio* L. and rainbow trout, *Salmo gairdneri* Richardson, to *Renibacterium salmoninarum*. J. Fish Dis. 1989; 12(4): 367−372. doi: [10.1111/j.1365- 2761.1989.tb00325.x](http://dx.doi.org/10.1111/j.1365-2761.1989.tb00325.x)

146. Sakai M, Atsuta S, Kobatachi M. The detection of serum antibody to *Renibacterium salmoninarum* in pen‐cultured Coho salmon, *Oncorhynchus kisutch* (Walbaum). J. Fish Dis. 1991; 14(2): 243−246.

147. Sakai M, Atsuta S, Kobayashi M. Susceptibility of five salmonid fishes to *Renibacterium salmoninarum.* Fish Pathol. 1991; 26(3): 159−160. doi: [10.3147/jsfp.26.159](https://doi.org/10.3147/jsfp.26.159)

148. Sakai M, Kobayashi M. Detection of *Renibacterium salmoninarum*, the causative agent of bacterial kidney disease in salmonid fish, from pen-cultured Coho salmon. Appl. Environ. Microbiol. 1991; 58(3): 1061−1063. doi: [10.1128/aem.58.3.1061-1063.1992](https://doi.org/10.1128/aem.58.3.1061-1063.1992)

149. Salonius K, Siderakis C, MacKinnon AM, Griffiths SG. Use of *Arthrobacter davidanieli* as a live vaccine against *Renibacterium salmoninarum* and *Piscirickettsia salmonis* in salmonids. Dev. Biol. 2005; 121: 189−197. PMID: 15962482

150. Sanders JE, Pilcher KS, Fryer JL. Relation of water temperature to bacterial kidney disease in Coho salmon (*Oncorhynchus kisutch*), Sockeye salmon (*O. nerka*), and steelhead trout (*Salmo gairdneri*). J. Fish Board Can. 1978; 35(1): 8−11. doi: [10.1139/f78-00](https://doi.org/10.1139/f78-002)2

151. Sanders JE, Barros RMJ. Evidence by the fluorescent antibody test for the occurrence of *Renibacterium salmoninarum* among salmonid fish in Chile. J. Wildl. Dis. 1986; 22(2): 255−257. doi: [10.7589/0090-3558-22.2.255](https://doi.org/10.7589/0090-3558-22.2.255)

152. Sanders JE, Long JJ, Arakawa CK, Bartholomew JL, Rohovec JS. Prevalence of *Renibacterium salmoninarum* among downstream migrating salmonids in the Columbia River. J. Aquat. Anim. Health 1992; 4(1): 72−75. doi: [10.1577/1548- 8667(1992)004<0072:PORSAD>2.3.CO;2](https://doi.org/10.1577/1548-8667(1992)004%3C0072:PORSAD%3E2.3.CO;2)

153. Savas H, Altinok I, Cakmak E, Firidin S. Isolation of *Renibacterium salmoninarum* from cultured black sea salmon (*Salmo trutta labrax*): first report in Turkey. B. Euro. Assoc. Fish Pathol. 2006; 26(6): 238.

154. Schreck CB, Stahl TP, Davis LE, Roby DD, Clemens BJ. Mortality estimates of juvenile spring–summer Chinook salmon in the lower Columbia River and estuary, 1992–1998: Evidence for delayed mortality? Trans. Am. Fish Soc. 2006; 135(2): 457−475. doi: 10.1577/t05-184.1

155. Senson PR, Stevenson RM. Production of the 57 kDa major surface antigen by a non- agglutinating strain of the fish pathogen *Renibacterium salmoninarum*. Dis. Aquat. Organ 1999; 38(1): 23−31. doi: [10.3354/dao038023](https://doi.org/10.3354/dao038023)

156. Sergeenko NV, Ustimenko EA, Eliseikina MG, Kuhlevskiy AD, Bochkova EV, Ryazanova TV. First report of bacterial kidney disease in Coho salmon *Oncorhynchus kisutch* in Russia. Dis. Aquat. Organ 2020; 140: 31−36. doi: [10.3354/dao03486](https://doi.org/10.3354/dao03486)

157. Sigurjónsdóttir H, Jónsdóttir H, Benediktsdóttir E. Measures applied to control *Renibacterium salmoninarum* infection in Atlantic salmon: A retrospective study of two sea ranches in Iceland. Aquac. 2000; 186(3−4): 193−203.

158. Smith IW. The occurrence and pathology of Dee disease. Freshw. Salm. Fish Res. 1964; 34: 1−12.

159. Snieszko SF, Griffin PJ. Kidney disease in brook trout and its treatment. Prog. Fish C. 1955; 17(1): 3−13.

160. Snyman H, Cai H, Kamaitis M. Bacterial kidney disease (BKD) in rainbow trout (*Oncorhynchus mykiss).* AHL Newsletter 2020; 24(2): 17−19.

161. Souter BW, Dwilow AG, Knight K. *Renibacterium salmoninarum* in wild Arctic charr *Salvelinus alpinus* and lake trout *S. namaycush* from the Northwest Territories, Canada. Dis. Aquat. Organ 1987; 3(2): 151−154. doi: 10.3354/dao0031531

162. Speare DJ, Ostland VE, Ferguson HW. Pathology associated with meningoencephalitis during bacterial kidney disease of salmonids. Res. Vet. Sci. 1993; 54(1): 25−31. doi: [10.1016/0034-5288(93)90006-2](https://doi.org/10.1016/0034-5288(93)90006-2)

163. Speare DJ. Differences in patterns of meningoencephalitis due to bacterial kidney disease in farmed Atlantic and Chinook salmon. Res. Vet. Sci. 1997; 62(1): 79−80. doi: [10.1016/s0034-5288(97)90185-5](https://doi.org/10.1016/s0034-5288(97)90185-5)

164. Starliper CE. Teska JD. Relevance of *Renibacterium salmoninarum* in an asymptomatic carrier population of brook trout, *Salvelinus fontinalis* (Mitchill). J. Fish Dis. 1995; 18: 383−387. doi: [10.1111/j.1365-2761.1995.tb00330.x](https://doi.org/10.1111/j.1365-2761.1995.tb00330.x)

165. Starliper CE. Genetic diversity of North American isolates of *Renibacterium salmoninarum*. Dis. Aquat. Organ 1996; 27(3): 207−213.

166. Starliper CE, Smith DR, Shatzer T. Virulence of *Renibacterium salmoninarum* to salmonids. J. Aquat. Anim. Health 1997; 9(1): 1−7.

167. Starliper CE, Schill WB, Mathias J. Performance of serum-free broth media for growth of *Renibacterium salmoninarum*. Dis. Aquat. Organ 1998; 34(1): 21−26. doi: [10.3354/dao034021](https://doi.org/10.3354/dao034021)

168. Suzumoto BK, Schreck CB, McIntyre JD. Relative resistances of three transferrin genotypes of Coho salmon (*Oncorhynchus kisutch*) and their hematological responses to bacterial kidney disease. J. Fish Board Can. 1977; 34(1): 1−8. doi: [10.1139/f77-00](https://doi.org/10.1139/f77-001)

169. Teska JD. In vitro growth of the bacterial kidney disease organism *Renibacterium salmoninarum* on a nonserum, noncharcoal-based “homospecies-metabolite” medium. J. Wildl. Dis. 1994; 30(3): 383−388. doi: 10.7589/0090-3558-30.3.383.

170. Teska JD, Dawson A, Starliper CE, Tillinghast D. A multiple‐technique approach to investigating the presumptive low‐level detection of *Renibacterium salmoninarum* at a broodstock hatchery in Maine. J. Aquat. Anim. Health 1995; 7(3): 251−256.

171. Thorarinsson R, Landolt ML, Elliott DG, Pascho RJ, Hardy RW. Effect of dietary vitamin E and selenium on growth, survival and the prevalence of *Renibacterium salmoninarum* infection in Chinook salmon (*Oncorhynchus tshawytscha*). Aquac. 1994; 121(4): 343−358. doi: [10.1016/0044-8486(94)90269-0](https://doi.org/10.1016/0044-8486(94)90269-0)

172. Toranzo AE, Barja JL, Colwell RR, Hetrick FM. Characterization of plasmids in bacterial fish pathogen. Infect. Immun. 1983; 39(1): 184−192. doi: [10.1128/iai.39.1.184- 192.1983](https://doi.org/10.1128%2Fiai.39.1.184-192.1983)

173. Turaga PS, Wiens GD, Kaattari SL. Analysis of *Renibacterium salmoninarum* antigen production in situ. Fish Pathol. 1987; 22(4): 209−214.

174. Turaga P, Wiens G, Kaattari S. Bacterial kidney disease: The potential role of soluble protein antigen(s). J. Fish Biol. 1987; 31: 191−194. doi: [10.1111/j.1095- 8649.1987.tb05312.x](https://doi.org/10.1111/j.1095-8649.1987.tb05312.x)

175. Turgut E, Thompson K, Ellis AE, Adams A. Persistence of Renibacterium salmoninarum in experimentally infected rainbow trout (*Oncorhynchus mykiss*). Euro. Assoc. Fish Pathol. Bull. 2008; 28(2): 66−76.

176. Wallace IS, Munro LA, Kilburn R, Hall M, Black J, Raynard RS, Murray AG. A report on the effectiveness of cage and farm-level fallowing of the control of bacterial kidney disease and sleeping disease on large cage-based trout farms in Scotland. Scottish Marine and Freshwater Science Reports, 2011; 2: 36

177. Wallis J. Kidney disease in adult Chinook salmon and its transmission by feeding to young Chinook salmon. Research Briefs 1955.

178. Wedemeyer GA, Ross AJ. Nutritional factors in the biochemical pathology of corynebacterial kidney disease in the Coho salmon (*Oncorhynchus kisutch*). J. Fish Board Can. 1973; 30(2): 296−298. doi: [10.1139/f73-052](https://doi.org/10.1139/f73-052)

179. White MR, Albregts SR, Wu CC, Breidert B. The use of kidney biopsy of broodstock steelhead trout (*Oncorhyncus mykiss*) to determine the status of bacterial kidney disease infection. J. Vet. Dian. Invest. 1996; 8(4): 519−522. doi: [10.1177/104063879600800429](https://doi.org/10.1177/104063879600800429)

180. Wiens GD, Kaattari SL. Monoclonal antibody analysis of common surface protein(s) of *Renibacterium salmoninarum*. Fish Pathol. 1989; 24(1): 1−7. doi: [10.3147/jsfp.24.1](https://doi.org/10.3147/jsfp.24.1)

181. Wiens GD, Kaattari SL. Monoclonal antibody characterization of a leukoagglutinin produced by *Renibacterium salmoninarum*. Infect. Immun. 1991; 59(2): 631−637. doi: [10.1128/iai.59.2.631-637.1991](https://doi.org/10.1128%2Fiai.59.2.631-637.1991)

182. Wiens GD. Bacterial kidney disease (*Renibacterium salmoninarum*). Fish diseases and disorders: viral, bacterial and fungal infections 1999; 3: 269−301.

183. Winter GW, Schreck CB, McIntyre JD. Resistance of different stocks and transferrin genotypes of Coho salmon, *Oncorhynchus kisutch*, and steelhead trout, *Salmo gairdneri*, to bacterial kidney disease and vibriosis. Fish Bull. 1980; 77(4): 795−802.

184. Wolf K, Dunbar CE. Methods of infecting trout with kidney disease and some effects of temperature on experimental infections (No. 286). US Department of the Interior, Fish and Wildlife Service 1959.

185. Wood PA, Kaattari SL. Enhanced immunogenicity of *Renibacterium salmoninarum* in Chinook salmon after removal of the bacterial cell surface-associated 57 kDa protein. Dis. Aquat. Organ 1996; 25(1-2): 71−79. doi: 10.3354/dao025071

186. Wood EM, Yasutake WT. Histopathology of kidney disease in fish. Am. J. Pathol. 1956; 32:845−857. PMID 13339956.

187. Young CL, Chapman GB. Ultrastructural aspects of the causative agent and renal histopathology of bacterial kidney disease in brook trout (*Salvelinus fontinalis*). J. Fish Board Can. 1978; 35(9): 1234−1248.

188. Zajac D, Brunson R, Comstock R, Gilliam K. Relationship between coded wire tagging and bacterial kidney disease in Coho salmon. Prog. Fish C. 1988; 50(3): 187−188.
