# Supplementary material for: Leveraging detection uncertainty to estimate Renibacterium salmoninarum infection status among multiple tissues and assays
Source: PLoS One. 2025 May 8;20(5):e0323010. doi: 10.1371/journal.pone.0323010 (PMC12061193; doi:10.1371/journal.pone.0323010)
Supplement: S1 Table — Multinomial logistic regression results (z-test statistic and p-value, α = 0.05) comparing the effects of covariates (sex, length, or weight) on Renibacterium salmoninarum detection using either DFAT or qPCR and for each state of infection. (DOCX) [file pone.0323010.s002.docx]

**S1 Table. Multinomial logistic regression results.** Multinomial logistic regression results (z-test statistic and p-value, α = 0.05) comparing the effects of covariates (sex, length, or weight) on Renibacterium salmoninarum detection using either DFAT or qPCR and for each state of infection.

| **Model** | **Covariate** | **State of Infection** | **Z** | **p-value** |
| --- | --- | --- | --- | --- |
| **qPCR** | Sex | State 1 {K− L−} | 0.38 | 0.71 |
|  |  | State 2 {K+ L−} | -0.38 | 0.71 |
|  |  | State 3 {K− L+} | 0.99 | 0.32 |
|  |  | State 4 {K+ L+} | 0.21 | 0.83 |
|  | Length | State 1 {K− L−} | 0.50 | 0.61 |
|  |  | State 2 {K+ L−} | -0.50 | 0.61 |
|  |  | State 3 {K− L+} | -1.22 | 0.22 |
|  |  | State 4 {K+ L+} | -0.48 | 0.63 |
|  | Weight | State 1 {K− L−} | 1.13 | 0.26 |
|  |  | State 2 {K+ L−} | -1.13 | 0.26 |
|  |  | State 3 {K− L+} | -0.82 | 0.41 |
|  |  | State 4 {K+ L+} | -1.11 | 0.27 |
| **DFAT** | Sex | State 1 {K− L−} | 1.26 | 0.21 |
|  |  | State 2 {K+ L−} | -1.26 | 0.21 |
|  |  | State 3 {K− L+} | -0.26 | 0.79 |
|  |  | State 4 {K+ L+} | -0.73 | 0.46 |
|  | Length | State 1 {K− L−} | -1.24 | 0.21 |
|  |  | State 2 {K+ L−} | 1.24 | 0.21 |
|  |  | State 3 {K− L+} | -0.66 | 0.51 |
|  |  | State 4 {K+ L+} | 0.15 | 0.88 |
|  | Weight | State 1 {K− L−} | -1.23 | 0.22 |
|  |  | State 2 {K+ L−} | 1.23 | 0.22 |
|  |  | State 3 {K− L+} | -0.40 | 0.69 |
|  |  | State 4 {K+ L+} | 0.08 | 0.93 |
