# Supplementary material for: Leveraging detection uncertainty to estimate Renibacterium salmoninarum infection status among multiple tissues and assays
Source: PLoS One. 2025 May 8;20(5):e0323010. doi: 10.1371/journal.pone.0323010 (PMC12061193; doi:10.1371/journal.pone.0323010)
Supplement: S1 Equation — Model statement for the static multistate occupancy model in a Bayesian hierarchical framework. (DOCX) [file pone.0323010.s003.docx]

**S1 Equation. Bayesian Hierarchical model for static multistate occupancy analysis of Renibacterium salmoninarum infection states.** Model statement for the static multistate occupancy model in a Bayesian hierarchical framework.

$$y_{i,j} \sim\mathrm{categorical}\boldsymbol{(P}_{i,j}*z_{i})$$

$$z_{i} \sim categorical(\boldsymbol{\phi}\boldsymbol{)}$$

$$\boldsymbol{\phi}=\left[ \Psi_{1}, \Psi_{2},\Psi_{3},\Psi_{4} \right]$$

$\boldsymbol{P}_{i,j}$ = $\left[ \begin{matrix} 1 \\ \begin{matrix} 1-p_{2} \\ \begin{matrix} 1-p_{3} \\ \left( 1-p_{2} \right)*\left( 1-p_{3} \right) \end{matrix} \end{matrix} \end{matrix}\begin{matrix} 0 \\ \begin{matrix} p_{2} \\ \begin{matrix} 0 \\ p_{2}*\left( 1-p_{3} \right) \end{matrix} \end{matrix} \end{matrix}\begin{matrix} 0 \\ \begin{matrix} 0 \\ \begin{matrix} p_{3} \\ \left( 1-p_{2} \right)*p_{3} \end{matrix} \end{matrix} \end{matrix}\begin{matrix} 0 \\ \begin{matrix} 0 \\ \begin{matrix} 0 \\ p_{2}*p_{3} \end{matrix} \end{matrix} \end{matrix} \right]$

$\beta_{\Psi,k} \sim\mathrm{gamma}\left( 1,1 \right) k=1,2,3,\mathrm{or} 4$

$$\Psi_{1}= \frac{\beta_{\Psi,1}}{\sum_{i=1}^{4} \beta_{\Psi,k}}$$

$$\Psi_{2}= \frac{\beta_{\Psi,2}}{\sum_{i=1}^{4} \beta_{\Psi,k}}$$

$$\Psi_{3}= \frac{\beta_{\Psi,3}}{\sum_{i=1}^{4} \beta_{\Psi,k}}$$

$$\Psi_{4}= \frac{\beta_{\Psi,4}}{\sum_{i=1}^{4} \beta_{\Psi,k}}$$

$$p_{2} \sim\mathrm{uniform}\left( 0,1 \right)$$

$$p_{3} \sim\mathrm{uniform}\left( 0,1 \right)$$
