# Supplementary material for: Leveraging detection uncertainty to estimate Renibacterium salmoninarum infection status among multiple tissues and assays
Source: PLoS One. 2025 May 8;20(5):e0323010. doi: 10.1371/journal.pone.0323010 (PMC12061193; doi:10.1371/journal.pone.0323010)
Supplement: S1 Fig — Values represent the probability of observing an infection state given the probability of the true state of infection for each of the assay models (DFAT top, qPCR bottom). (DOCX) [file pone.0323010.s004.docx]

**S1 Fig. Model predictions of infection states probability.** Values represent the probability of observing an infection state given the probability of the true state of infection for each of the assay models (DFAT top, qPCR bottom).

$$\boldsymbol{Observed State}$$

$$\boldsymbol{1 2 3 4}$$

$\boldsymbol{True State}\begin{matrix} \boldsymbol{1} \\ \begin{matrix} \boldsymbol{2} \\ \begin{matrix} \boldsymbol{3} \\ \boldsymbol{4} \end{matrix} \end{matrix} \end{matrix}$ $\left[ \begin{matrix} 1 \\ \begin{matrix} 0.46 \\ \begin{matrix} 0.64 \\ 0.29 \end{matrix} \end{matrix} \end{matrix}\begin{matrix} 0 \\ \begin{matrix} 0.54 \\ \begin{matrix} 0 \\ 0.35 \end{matrix} \end{matrix} \end{matrix}\begin{matrix} 0 \\ \begin{matrix} 0 \\ \begin{matrix} 0.36 \\ 0.17 \end{matrix} \end{matrix} \end{matrix}\begin{matrix} 0 \\ \begin{matrix} 0 \\ \begin{matrix} 0 \\ 0.19 \end{matrix} \end{matrix} \end{matrix} \right]$

$$\boldsymbol{Observed State}$$

$$\boldsymbol{1 2 3 4}$$

$\boldsymbol{True State}\begin{matrix} \boldsymbol{1} \\ \begin{matrix} \boldsymbol{2} \\ \begin{matrix} \boldsymbol{3} \\ \boldsymbol{4} \end{matrix} \end{matrix} \end{matrix}$ $\left[ \begin{matrix} 1 \\ \begin{matrix} 0.27 \\ \begin{matrix} 0.39 \\ 0.11 \end{matrix} \end{matrix} \end{matrix}\begin{matrix} 0 \\ \begin{matrix} 0.73 \\ \begin{matrix} 0 \\ 0.28 \end{matrix} \end{matrix} \end{matrix}\begin{matrix} 0 \\ \begin{matrix} 0 \\ \begin{matrix} 0.61 \\ 0.16 \end{matrix} \end{matrix} \end{matrix}\begin{matrix} 0 \\ \begin{matrix} 0 \\ \begin{matrix} 0 \\ 0.45 \end{matrix} \end{matrix} \end{matrix} \right]$
