# Supplementary material for: Leveraging detection uncertainty to estimate Renibacterium salmoninarum infection status among multiple tissues and assays
Source: PLoS One. 2025 May 8;20(5):e0323010. doi: 10.1371/journal.pone.0323010 (PMC12061193; doi:10.1371/journal.pone.0323010)
Supplement: S1 Data — The data supporting the findings of the study are included. These data include the detections of Renibacterium salmoninarum from direct fluorescent antibody tests and quantitative polymerase chain reaction used to inform the model. (DOCX) [file pone.0323010.s005.docx]

| **Fish ID** | **Sx** | | **L** | | **Wt** | | **SP** | | **SP.D1** | | **SP.D2** | | **SP.P1** | | **SP.P2** | | **SP.P1.CQ** | | **SP.P2.CQ** | | **SP.Q** | | | **LV** | **LV.D1** | | **LV.D2** | | **LV.P1** | | **LV.P2** | | **LV.P1.CQ** | | **LV.P2.CQ** | | **LV.Q** | | | **KD** | **KD.D1** | | **KD.D2** | | **KD.P1** | | **KD.P2** | | **KD.P1** | | **KD.P2** | | **KD.Q** | |  |  |
| --- | --- | --- | --- | --- | --- | --- | --- | --- | --- | --- | --- | --- | --- | --- | --- | --- | --- | --- | --- | --- | --- | --- | --- | --- | --- | --- | --- | --- | --- | --- | --- | --- | --- | --- | --- | --- | --- | --- | --- | --- | --- | --- | --- | --- | --- | --- | --- | --- | --- | --- | --- | --- | --- | --- | --- | --- |
| 001 | F | | 380 | | 819 | | SP2001 | | 0 | | 0 | | 1 | | 1 | | 36.001 | | 37.217 | | 2.364443058 | | | LV2801 | 0 | | 0 | | 1 | | 1 | | 33.757 | | 37.148 | | 8.340984431 | | | KD3601 | 0 | | 0 | | 0 | | 1 | | 38.023 | | 34.95 | | 6.733513856 | |  |  |
| 002 | M | | 273 | | 295 | | SP2002 | | 0 | | 0 | | 1 | | 1 | | 37.041 | | 37.628 | | 1.353709831 | | | LV2803 | 0 | | 0 | | 1 | | 1 | | 34.435 | | 36.552 | | 5.912012597 | | | KD3602 | 0 | | 0 | | 1 | | 1 | | 37.484 | | 36.595 | | 1.697355962 | |  |  |
| 003 | F | | 355 | | 555 | | SP2003 | | 0 | | 0 | | 1 | | 1 | | 37.332 | | 35.681 | | 2.711040627 | | | LV2804 | 0 | | 0 | | 0 | | 1 | | 38.158 | | 32.374 | | 38.92460155 | | | KD3603 | 0 | | 0 | | 0 | | 1 | | 38.836 | | 37.024 | | 1.639650387 | |  |  |
| 004 | M | | 250 | | 162 | | SP2004 | | 0 | | 0 | | 1 | | 1 | | 36.892 | | 36.526 | | 2.047827148 | | | LV2802 | 0 | | 0 | | 1 | | 1 | | 34.875 | | 34.274 | | 8.878683062 | | | KD3604 | 0 | | 0 | | 1 | | 1 | | 36.704 | | 36.853 | | 1.940569401 | |  |  |
| 005 | F | | 390 | | 742 | | SP2005 | | 0 | | 0 | | 1 | | 1 | | 37.136 | | 36.500 | | 1.931053599 | | | LV2805 | 0 | | 0 | | 1 | | 1 | | 34.375 | | 35.081 | | 8.060147335 | | | KD3605 | 1 | | 1 | | 0 | | 0 | | 40 | | 38.362 | | 0 | |  |  |
| 006 | M | | 265 | | 171 | | SP2006 | | 0 | | 0 | | 0 | | 1 | | 38.121 | | 36.489 | | 2.360503191 | | | LV2806 | 0 | | 0 | | 1 | | 1 | | 36.961 | | 36.581 | | 1.964333722 | | | KD3606 | 0 | | 0 | | 1 | | 0 | | 34.716 | | 0 | | 7.896944851 | |  |  |
| 007 | F | | 345 | | 480 | | SP2007 | | 0 | | 0 | | 0 | | 1 | | 0.00 | | 37.211 | | 1.443565647 | | | LV2807 | 0 | | 0 | | 1 | | 1 | | 36.827 | | 32.323 | | 21.08777922 | | | KD3607 | 0 | | 0 | | 0 | | 1 | | 38.694 | | 36.91 | | 1.772035946 | |  |  |
| 008 | M | | 252 | | 163 | | SP2008 | | 0 | | 0 | | 0 | | 0 | | 39.149 | | 38.541 | | 0 | | | LV2808 | 0 | | 0 | | 1 | | 1 | | 34.226 | | 36.83 | | 6.448424858 | | | KD3608 | 0 | | 0 | | 0 | | 0 | | 38.861 | | 39.48 | | 0 | |  |  |
| 009 | F | | 256 | | 588 | | SP2009 | | 0 | | 0 | | 1 | | 1 | | 36.693 | | 37.134 | | 1.787790905 | | | LV2809 | 0 | | 0 | | 1 | | 1 | | 33.907 | | 34.646 | | 10.99193592 | | | KD3609 | 0 | | 0 | | 0 | | 0 | | 37.966 | | 37.983 | | 0 | |  |  |
| 010 | M | | 282 | | 201 | | SP2010 | | 0 | | 0 | | 1 | | 0 | | 36.537 | | 38.932 | | 2.284578901 | | | LV2810 | 0 | | 0 | | 1 | | 1 | | 35.137 | | 35.834 | | 4.807971914 | | | KD3610 | 0 | | 0 | | 0 | | 0 | | 38.719 | | 38.751 | | 0 | |  |  |
| 011 | F | | 408 | | 814 | | SP2011 | | 0 | | 0 | | 1 | | 1 | | 34.082 | | 33.877 | | 13.07295495 | | | LV2811 | 0 | | 0 | | 1 | | 1 | | 35.633 | | 35.215 | | 4.925130249 | | | KD3611 | 0 | | 0 | | 1 | | 1 | | 31.878 | | 32.228 | | 48.78136336 | |  |  |
| 012 | M | | 280 | | 225 | | SP2028 | | 0 | | 0 | | 1 | | 1 | | 36.92 | | 36.856 | | 1.799815506 | | | LV2829 | 0 | | 0 | | 1 | | 1 | | 35.886 | | 35.999 | | 3.427528933 | | | KD3629 | 0 | | 0 | | 1 | | 1 | | 34.095 | | 35.216 | | 8.836130662 | |  |  |
| 013 | F | | 344 | | 382 | | SP2013 | | 1 | | 1 | | 1 | | 1 | | 15.478 | | 14.723 | | 5175309.961 | | | LV2813 | 1 | | 0 | | 1 | | 1 | | 20.605 | | 21.35 | | 94437.90024 | | | KD3613 | 1 | | 1 | | 1 | | 1 | | 20.728 | | 20.232 | | 130209.6762 | |  |  |
| 014 | M | | 224 | | 114 | | SP2012 | | 0 | | 0 | | 1 | | 1 | | 37.346 | | 36.388 | | 1.922676128 | | | LV2812 | 0 | | 0 | | 1 | | 1 | | 35.229 | | 35.802 | | 4.668562483 | | | KD3612 | 0 | | 0 | | 1 | | 1 | | 35.5 | | 36.189 | | 3.762663049 | |  |  |
| 015 | F | | 320 | | 419 | | SP2015 | | 0 | | 0 | | 1 | | 1 | | 31.328 | | 30.534 | | 107.8329701 | | | LV2815 | 0 | | 0 | | 1 | | 1 | | 34.179 | | 32.774 | | 20.51299787 | | | KD3615 | 1 | | 1 | | 1 | | 1 | | 33.017 | | 32.74 | | 27.72823584 | |  |  |
| 016 | M | | 264 | | 206 | | SP2014 | | 0 | | 1 | | 1 | | 1 | | 30.215 | | 31.545 | | 118.9196155 | | | LV2814 | 0 | | 0 | | 1 | | 1 | | 29.705 | | 33.729 | | 127.5968986 | | | KD3614 | 1 | | 1 | | 1 | | 1 | | 31.321 | | 31.212 | | 82.81757562 | |  |  |
| 017 | F | | 358 | | 492 | | SP2018 | | 0 | | 0 | | 1 | | 1 | | 33.013 | | 32.968 | | 25.58079775 | | | LV2818 | 0 | | 1 | | 1 | | 1 | | 35.698 | | 35.337 | | 4.609445298 | | | KD3618 | 0 | | 0 | | 1 | | 1 | | 32.179 | | 29.436 | | 166.1915514 | |  |  |
| 019 | F | | 301 | | 336 | | SP2016 | | 0 | | 0 | | 1 | | 1 | | 34.19 | | 32.306 | | 26.03453479 | | | LV2816 | 0 | | 0 | | 1 | | 1 | | 33.442 | | 35.744 | | 11.36368058 | | | KD3616 | 1 | | 1 | | 1 | | 1 | | 33.682 | | 34.955 | | 11.34053217 | |  |  |
| 020 | M | | 250 | | 164 | | SP2032 | | 0 | | 0 | | 0 | | 0 | | 38.01 | | 37.995 | | 0 | | | LV2832 | 0 | | 0 | | 1 | | 1 | | 36.16 | | 37.015 | | 2.301568594 | | | KD3632 | 0 | | 0 | | 1 | | 1 | | 37.692 | | 35.93 | | 2.247285839 | |  |  |
| 021 | F | | 337 | | 430 | | SP2019 | | 0 | | 0 | | 1 | | 1 | | 30.608 | | 30.327 | | 143.2694505 | | | LV2819 | 0 | | 0 | | 1 | | 1 | | 35.479 | | 34.785 | | 6.115391615 | | | KD3619 | 0 | | 0 | | 1 | | 1 | | 33.761 | | 34.132 | | 13.44419644 | |  |  |
| 022 | M | | 290 | | 246 | | SP2017 | | 0 | | 0 | | 1 | | 1 | | 30.083 | | 32.074 | | 116.5304771 | | | LV2817 | 0 | | 0 | | 1 | | 1 | | 34.17 | | 33.712 | | 13.55083312 | | | KD3617 | 0 | | 1 | | 1 | | 1 | | 34.207 | | 34.061 | | 11.75307941 | |  |  |
| 023 | F | | 367 | | 540 | | SP2020 | | 1 | | 0 | | 1 | | 1 | | 31.488 | | 31.522 | | 70.35646868 | | | LV2820 | 0 | | 0 | | 1 | | 1 | | 33.361 | | 34.02 | | 16.27979631 | | | KD3620 | 0 | | 0 | | 1 | | 1 | | 34.341 | | 35.275 | | 7.795681122 | |  |  |
| 024 | M | | 252 | | 167 | | SP2030 | | 0 | | 0 | | 1 | | 1 | | 33.65 | | 32.411 | | 27.15582713 | | | LV2830 | 0 | | 0 | | 0 | | 1 | | 38.257 | | 36.695 | | 2.051491219 | | | KD3630 | 0 | | 0 | | 1 | | 1 | | 35.691 | | 36.76 | | 3.013791438 | |  |  |
| 025 | F | | 340 | | 476 | | SP2021 | | 0 | | 0 | | 1 | | 1 | | 33.141 | | 32.894 | | 25.20061334 | | | LV2821 | 0 | | 0 | | 1 | | 1 | | 35.301 | | 35.846 | | 4.479688067 | | | KD3621 | 0 | | 0 | | 1 | | 1 | | 35.648 | | 35.91 | | 3.843699931 | |  |  |
| 026 | M | | 251 | | 172 | | SP2029 | | 0 | | 0 | | 0 | | 0 | | 0.00 | | 0.00 | | 0 | | | LV2828 | 0 | | 0 | | 1 | | 0 | | 35.429 | | 37.853 | | 4.859071139 | | | KD3628 | 0 | | 0 | | 1 | | 1 | | 35.003 | | 36.641 | | 4.311563733 | |  |  |
| 027 | F | | 378 | | 559 | | SP2023 | | 0 | | 0 | | 0 | | 0 | | 38.00 | | 0.00 | | 0 | | | LV2823 | 0 | | 0 | | 1 | | 1 | | 36.073 | | 35.909 | | 3.318870449 | | | KD3623 | 0 | | 0 | | 1 | | 1 | | 36.138 | | 34.168 | | 7.233922956 | |  |  |
| 028 | M | | 271 | | 182 | | SP2026 | | 0 | | 0 | | 0 | | 0 | | 0.00 | | 38.088 | | 0 | | | LV2826 | 0 | | 0 | | 1 | | 0 | | 37.208 | | 38.363 | | 1.446518327 | | | KD3626 | 0 | | 0 | | 1 | | 0 | | 34.566 | | 37.87 | | 8.746397265 | |  |  |
| 029 | F | | 352 | | 473 | | SP2025 | | 0 | | 0 | | 1 | | 0 | | 37.54 | | 0.00 | | 1.152196444 | | | LV2825 | 0 | | 0 | | 1 | | 1 | | 36.077 | | 37.283 | | 2.249828633 | | | KD3625 | 0 | | 0 | | 1 | | 1 | | 35.856 | | 32.581 | | 18.71940721 | |  |  |
| 030 | M | | 240 | | 139 | | SP2031 | | 0 | | 1 | | 0 | | 0 | | 37.95 | | 0.00 | | 0 | | | LV2831 | 0 | | 0 | | 1 | | 1 | | 36.375 | | 36.149 | | 2.76335297 | | | KD3631 | 0 | | 0 | | 1 | | 1 | | 36.216 | | 36.035 | | 3.029372297 | |  |  |
| 031 | F | | 368 | | 651 | | SP2022 | | 0 | | 0 | | 0 | | 0 | | 37.81 | | 38.031 | | 0 | | | LV2822 | 0 | | 0 | | 1 | | 1 | | 35.463 | | 35.164 | | 5.284036641 | | | KD3622 | 0 | | 0 | | 1 | | 1 | | 35.743 | | 35.65 | | 4.051766124 | |  |  |
| 032 | M | | 250 | | 153 | | SP2027 | | 0 | | 0 | | 0 | | 0 | | 0.00 | | 0.00 | | 0 | | | LV2827 | 0 | | 0 | | 1 | | 0 | | 34.012 | | 38.111 | | 12.75565449 | | | KD3627 | 0 | | 0 | | 1 | | 1 | | 35.8 | | 34.501 | | 6.458198656 | |  |  |
| 033 | F | | 422 | | 898 | | SP2033 | | 1 | | 0 | | 0 | | 0 | | 0.00 | | 0.00 | | 0 | | | LV2833 | 0 | | 0 | | 1 | | 1 | | 37.052 | | 36.44 | | 2.024644699 | | | KD3633 | 0 | | 0 | | 1 | | 0 | | 36.888 | | 38 | | 1.798788661 | |  |  |
| 034 | M | | 244 | | 154 | | SP2024 | | 0 | | 0 | | 1 | | 0 | | 36.13 | | 38.695 | | 3.006171926 | | | LV2824 | 0 | | 0 | | 1 | | 1 | | 34.625 | | 35.537 | | 6.458179813 | | | KD3624 | 0 | | 0 | | 1 | | 1 | | 36.118 | | 35.146 | | 4.465569942 | |  |  |
| 035 | F | | 335 | | 434 | | SP2035 | | 0 | | 0 | | 1 | | 1 | | 36.75 | | 34.937 | | 4.388101485 | | | LV2835 | 1 | | 0 | | 1 | | 1 | | 33.793 | | 32.885 | | 21.14546012 | | | KD3635 | 0 | | 0 | | 1 | | 1 | | 35.772 | | 34.897 | | 5.413886066 | |  |  |
| 036 | M | | 277 | | 237 | | SP2034 | | 0 | | 0 | | 1 | | 1 | | 33.24 | | 34.960 | | 14.11944841 | | | LV2834 | 0 | | 0 | | 1 | | 0 | | 37.303 | | 37.904 | | 1.355885076 | | | KD3634 | 0 | | 0 | | 0 | | 1 | | 38.061 | | 37.168 | | 1.486469373 | |  |  |
| 037 | F | | 368 | | 521 | | SP2039 | | 0 | | 0 | | 1 | | 1 | | 36.90 | | 37.194 | | 1.621654681 | | | LV2839 | 0 | | 0 | | 0 | | 1 | | 0 | | 36.691 | | 2.057087973 | | | KD3639 | 0 | | 0 | | 1 | | 1 | | 34.475 | | 35.721 | | 6.644187289 | |  |  |
| 038 | M | | 241 | | 164 | | SP2038 | | 1 | | 1 | | 0 | | 0 | | 37.92 | | 0.00 | | 0 | | | LV2838 | 0 | | 0 | | 1 | | 1 | | 37.639 | | 36.408 | | 1.786462283 | | | KD3638 | 0 | | 0 | | 1 | | 0 | | 37.049 | | 38.489 | | 1.611967347 | |  |  |
| 039 | F | | 325 | | 414 | | SP2037 | | 0 | | 0 | | 1 | | 1 | | 36.68 | | 35.930 | | 2.764123434 | | | LV2837 | 0 | | 0 | | 1 | | 1 | | 36.787 | | 36.881 | | 1.867135613 | | | KD3637 | 0 | | 0 | | 1 | | 1 | | 36.494 | | 36.866 | | 2.089211682 | |  |  |
| 041 | F | | 378 | | 717 | | SP2041 | | 0 | | 0 | | 0 | | 0 | | 0.00 | | 38.010 | | 0 | | | LV2841 | 0 | | 0 | | 1 | | 1 | | 37.007 | | 34.448 | | 5.568558126 | | | KD3641 | 0 | | 0 | | 0 | | 1 | | 38.001 | | 37.027 | | 1.63630348 | |  |  |
| 042 | M | | 256 | | 184 | | SP2036 | | 1 | | 0 | | 0 | | 0 | | 38.08 | | 0.00 | | 0 | | | LV2836 | 0 | | 0 | | 0 | | 1 | | 39.772 | | 36.803 | | 1.906001309 | | | KD3636 | 0 | | 0 | | 1 | | 1 | | 35.454 | | 37.547 | | 2.96265614 | |  |  |
| 043 | F | | 337 | | 506 | | SP2043 | | 0 | | 0 | | 0 | | 1 | | 38.38 | | 37.686 | | 1.044554827 | | | LV2843 | 0 | | 0 | | 1 | | 1 | | 36.442 | | 34.479 | | 5.858814681 | | | KD3643 | 0 | | 0 | | 1 | | 0 | | 36.806 | | 37.88 | | 1.902110718 | |  |  |
| 044 | M | | 262 | | 174 | | SP2040 | | 0 | | 0 | | 0 | | 1 | | 0.00 | | 35.932 | | 3.449574601 | | | LV2840 | 0 | | 0 | | 1 | | 1 | | 35.361 | | 36.037 | | 4.150451167 | | | KD3640 | 0 | | 0 | | 1 | | 1 | | 36.054 | | 36.564 | | 2.708734136 | |  |  |
| 045 | F | | 399 | | 705 | | SP2044 | | 1 | | 1 | | 0 | | 0 | | 38.00 | | 0.00 | | 0 | | | LV2844 | 0 | | 0 | | 0 | | 0 | | 0 | | 0 | | 0 | | | KD3644 | 0 | | 0 | | 0 | | 1 | | 38.953 | | 36.91 | | 1.772035946 | |  |  |
| 046 | M | | 263 | | 177 | | SP2046 | | 0 | | 0 | | 0 | | 1 | | 0.00 | | 36.555 | | 2.256741162 | | | LV2846 | 0 | | 0 | | 0 | | 0 | | 0 | | 0 | | 0 | | | KD3646 | 0 | | 0 | | 0 | | 0 | | 38.815 | | 39.345 | | 0 | |  |  |
| 047 | F | | 328 | | 409 | | SP2054 | | 0 | | 0 | | 0 | | 0 | | 38.52 | | 38.753 | | 0 | | | LV2854 | 0 | | 0 | | 1 | | 1 | | 33.823 | | 36.56 | | 8.378559787 | | | KD3654 | 0 | | 0 | | 0 | | 0 | | 0 | | 0 | | 0 | |  |  |
| 048 | M | | 218 | | 103 | | SP2048 | | 0 | | 0 | | 1 | | 0 | | 37.59 | | 0.00 | | 1.117417587 | | | LV2848 | 0 | | 0 | | 1 | | 1 | | 36.587 | | 36.708 | | 2.120746731 | | | KD3648 | 0 | | 0 | | 1 | | 0 | | 36.582 | | 38.553 | | 2.215619268 | |  |  |
| 051 | F | | 358 | | 538 | | SP2050 | | 0 | | 0 | | 1 | | 1 | | 36.36 | | 36.636 | | 2.359964252 | | | LV2850 | 0 | | 0 | | 0 | | 1 | | 37.994 | | 37.055 | | 1.605393264 | | | KD3650 | 0 | | 0 | | 0 | | 0 | | 0 | | 38.165 | | 0 | |  |  |
| 052 | M | | 285 | | 238 | | SP2042 | | 0 | | 0 | | 0 | | 0 | | 0.00 | | 0.00 | | 0 | | | LV2842 | 0 | | 0 | | 1 | | 1 | | 36.208 | | 35.49 | | 3.759867838 | | | KD3642 | 0 | | 0 | | 0 | | 0 | | 38.852 | | 39.84 | | 0 | |  |  |
| 053 | F | | 341 | | 483 | | SP2056 | | 1 | | 0 | | 0 | | 0 | | 0.00 | | 38.216 | | 0 | | | LV2856 | 0 | | 0 | | 1 | | 1 | | 37.171 | | 35.623 | | 2.870532661 | | | KD3656 | 0 | | 0 | | 0 | | 0 | | 38.365 | | 38.856 | | 0 | |  |  |
| 054 | M | | 290 | | 231 | | SP2055 | | 1 | | 0 | | 0 | | 0 | | 0.00 | | 0.00 | | 0 | | | LV2855 | 0 | | 0 | | 0 | | 0 | | 0 | | 38.842 | | 0 | | | KD3655 | 0 | | 0 | | 0 | | 1 | | 38.215 | | 37.029 | | 1.634076005 | |  |  |
| 055 | F | | 348 | | 458 | | SP2053 | | 0 | | 0 | | 0 | | 0 | | 0.00 | | 0.00 | | 0 | | | LV2853 | 0 | | 0 | | 0 | | 1 | | 0 | | 37.403 | | 1.266609724 | | | KD3653 | 0 | | 0 | | 0 | | 0 | | 38.289 | | 38.328 | | 0 | |  |  |
| 056 | M | | 233 | | 134 | | SP2045 | | 0 | | 0 | | 0 | | 0 | | 0.00 | | 0.00 | | 0 | | | LV2845 | 0 | | 0 | | 1 | | 1 | | 37.452 | | 36.426 | | 1.84451572 | | | KD3645 | 0 | | 0 | | 0 | | 0 | | 40.284 | | 0 | | 0 | |  |  |
| 057 | F | | 346 | | 598 | | SP2057 | | 0 | | 1 | | 1 | | 0 | | 36.92 | | 0.00 | | 1.757611639 | | | LV2857 | 0 | | 0 | | 1 | | 1 | | 37.459 | | 37.105 | | 1.385425034 | | | KD3657 | 0 | | 0 | | 0 | | 0 | | 0 | | 38.376 | | 0 | |  |  |
| 059 | F | | 385 | | 812 | | SP2060 | | 1 | | 1 | | 1 | | 1 | | 24.11 | | 23.732 | | 12411.62651 | | | LV2859 | 1 | | 1 | | 1 | | 0 | | 36.438 | | 0 | | 2.443939349 | | | KD3660 | 0 | | 0 | | 1 | | 1 | | 29.306 | | 28.704 | | 394.3120328 | |  |  |
| 060 | M | | 252 | | 147 | | SP2051 | | 0 | | 0 | | 0 | | 1 | | 0.00 | | 36.964 | | 1.708044942 | | | LV2851 | 0 | | 0 | | 0 | | 0 | | 0 | | 0 | | 0 | | | KD3651 | 0 | | 0 | | 0 | | 0 | | 38.019 | | 38.424 | | 0 | |  |  |
| 061 | F | | 369 | | 601 | | SP2062 | | 0 | | 0 | | 1 | | 0 | | 36.97 | | 38.169 | | 1.696450872 | | | LV2862 | 0 | | 0 | | 0 | | 0 | | 0 | | 0 | | 0 | | | KD3662 | 0 | | 0 | | 1 | | 1 | | 35.615 | | 35.518 | | 4.427082179 | |  |  |
| 062 | M | | 229 | | 121 | | SP2049 | | 0 | | 0 | | 1 | | 1 | | 36.71 | | 34.936 | | 4.412951338 | | | LV2849 | 0 | | 0 | | 1 | | 0 | | 37.612 | | 0 | | 1.098551665 | | | KD3649 | 0 | | 0 | | 1 | | 0 | | 37.738 | | 38.636 | | 1.008206766 | |  |  |
| 063 | F | | 356 | | 540 | | SP2063 | | 0 | | 0 | | 0 | | 0 | | 0.00 | | 0.00 | | 0 | | | LV2863 | 0 | | 0 | | 0 | | 0 | | 0 | | 0 | | 0 | | | KD3663 | 0 | | 0 | | 0 | | 1 | | 0 | | 36.839 | | 1.85983481 | |  |  |
| 064 | M | | 271 | | 194 | | SP2052 | | 0 | | 0 | | 0 | | 0 | | 0.00 | | 0.00 | | 0 | | | LV2852 | 0 | | 0 | | 0 | | 0 | | 0 | | 0 | | 0 | | | KD3652 | 0 | | 0 | | 0 | | 1 | | 39.437 | | 36.589 | | 2.205080908 | |  |  |
| 066 | M | | 265 | | 192 | | SP2058 | | 0 | | 1 | | 1 | | 0 | | 36.43 | | 0.00 | | 2.453947276 | | | LV2858 | 0 | | 0 | | 0 | | 1 | | 0 | | 37.189 | | 1.465359392 | | | KD3658 | 0 | | 0 | | 0 | | 0 | | 38.283 | | 38.2 | | 0 | |  |  |
| 067 | F | | 373 | | 816 | | SP2065 | | 1 | | 1 | | 0 | | 0 | | 0.00 | | 38.503 | | 0 | | | LV2865 | 0 | | 0 | | 0 | | 1 | | 0 | | 36.873 | | 1.817260349 | | | KD3665 | 0 | | 0 | | 1 | | 0 | | 36.945 | | 38.518 | | 1.730292421 | |  |  |
| 068 | M | | 244 | | 152 | | SP2059 | | 0 | | 1 | | 0 | | 0 | | 0.00 | | 39.331 | | 0 | | | LV2860 | 1 | | 0 | | 0 | | 1 | | 0 | | 27.213 | | 1308.733491 | | | KD3659 | 0 | | 0 | | 1 | | 0 | | 37.108 | | 38.094 | | 1.548474305 | |  |  |
| 070 | M | | 255 | | 164 | | SP2064 | | 1 | | 1 | | 0 | | 0 | | 0.00 | | 0.00 | | 0 | | | LV2864 | 0 | | 0 | | 1 | | 1 | | 34.542 | | 35.757 | | 6.388397431 | | | KD3664 | 0 | | 0 | | 1 | | 1 | | 35.159 | | 35.737 | | 4.889819454 | |  |  |
| 072 | M | | 235 | | 127 | | SP2061 | | 0 | | 0 | | 0 | | 0 | | 0.00 | | 0.00 | | 0 | | | LV2861 | 1 | | 0 | | 0 | | 0 | | 0 | | 38.837 | | 0 | | | KD3661 | 0 | | 0 | | 1 | | 0 | | 34.404 | | 38.472 | | 9.766723684 | |  |  |
| 073 | F | | 338 | | 411 | | SP2066 | | 1 | | 1 | | 0 | | 0 | | 0.00 | | 0.00 | | 0 | | | LV2866 | 0 | | 0 | | 0 | | 1 | | 0 | | 36.246 | | 2.785378021 | | | KD3666 | 0 | | 0 | | 0 | | 0 | | 0 | | 38.135 | | 0 | |  |  |
| 074 | M | | 247 | | 153 | | SP2067 | | 0 | | 0 | | 0 | | 0 | | 0.00 | | 0.00 | | 0 | | | LV2867 | 0 | | 0 | | 0 | | 1 | | 0 | | 36.072 | | 3.135836809 | | | KD3667 | 0 | | 0 | | 0 | | 0 | | 0 | | 38.023 | | 0 | |  |  |
| 075 | F | | 338 | | 468 | | SP2071 | | 1 | | 0 | | 0 | | 0 | | 0.00 | | 0.00 | | 0 | | | LV2871 | 0 | | 0 | | 0 | | 1 | | 0 | | 37.107 | | 1.549529339 | | | KD3671 | 0 | | 0 | | 0 | | 1 | | 0 | | 37.069 | | 1.590157814 | |  |  |
| 076 | M | | 263 | | 198 | | SP2069 | | 0 | | 0 | | 0 | | 0 | | 0.00 | | 0.00 | | 0 | | | LV2869 | 0 | | 0 | | 0 | | 1 | | 0 | | 37.558 | | 1.139708325 | | | KD3669 | 0 | | 0 | | 1 | | 0 | | 37.253 | | 0 | | 1.402855412 | |  |  |
| 077 | F | | 370 | | 590 | | SP2073 | | 0 | | 0 | | 0 | | 0 | | 39.04 | | 38.591 | | 0 | | | LV2873 | 0 | | 0 | | 1 | | 1 | | 36.578 | | 35.185 | | 3.979620946 | | | KD3673 | 0 | | 0 | | 1 | | 0 | | 36.836 | | 0 | | 1.86363893 | |  |  |
| 078 | M | | 250 | | 168 | | SP2068 | | 0 | | 0 | | 0 | | 0 | | 0.00 | | 0.00 | | 0 | | | LV2868 | 0 | | 0 | | 0 | | 1 | | 37.796 | | 36.548 | | 2.267526414 | | | KD3668 | 0 | | 0 | | 1 | | 1 | | 36.672 | | 36.769 | | 2.017268278 | |  |  |
| 079 | F | | 375 | | 623 | | SP2072 | | 0 | | 0 | | 0 | | 0 | | 0.00 | | 0.00 | | 0 | | | LV2872 | 0 | | 0 | | 0 | | 1 | | 38 | | 36.157 | | 2.959445866 | | | KD3672 | 0 | | 0 | | 0 | | 0 | | 0 | | 0 | | 0 | |  |  |
| 080 | M | | 279 | | 232 | | SP2070 | | 0 | | 0 | | 0 | | 0 | | 0.00 | | 0.00 | | 0 | | | LV2870 | 0 | | 0 | | 0 | | 1 | | 0 | | 37.53 | | 1.161652251 | | | KD3670 | 0 | | 0 | | 1 | | 0 | | 37.52 | | 38.014 | | 1.169591341 | |  |  |
| 081 | F | | 340 | | 485 | | SP2075 | | 0 | | 0 | | 0 | | 0 | | 0.00 | | 0.00 | | 0 | | | LV2875 | 0 | | 0 | | 1 | | 1 | | 36.893 | | 34.988 | | 4.177073036 | | | KD3675 | 0 | | 0 | | 0 | | 0 | | 37.972 | | 0 | | 0 | |  |  |
| 082 | M | | 242 | | 141 | | SP2074 | | 0 | | 0 | | 0 | | 0 | | 38.00 | | 0.00 | | 0 | | | LV2874 | 0 | | 0 | | 1 | | 1 | | 37.197 | | 36.701 | | 1.750260606 | | | KD3674 | 0 | | 0 | | 1 | | 0 | | 36.936 | | 0 | | 1.740931611 | |  |  |
| 083 | F | | 360 | | 649 | | SP2087 | | 0 | | 1 | | 0 | | 0 | | 0.00 | | 0.00 | | 0 | | | LV2887 | 0 | | 0 | | 1 | | 0 | | 35.194 | | 38.227 | | 5.702514588 | | | KD3687 | 0 | | 0 | | 0 | | 0 | | 0 | | 0 | | 0 | |  |  |
| 084 | M | | 220 | | 100 | | SP2082 | | 1 | | 0 | | 0 | | 0 | | 0.00 | | 0.00 | | 0 | | | LV2882 | 0 | | 0 | | 1 | | 0 | | 36.467 | | 0 | | 2.396140091 | | | KD3682 | 0 | | 0 | | 0 | | 0 | | 0 | | 0 | | 0 | |  |  |
| 085 | F | | 365 | | 604 | | SP2077 | | 1 | | 1 | | 0 | | 0 | | 0.00 | | 0.00 | | 0 | | | LV2878 | 0 | | 0 | | 1 | | 1 | | 34.32 | | 37.13 | | 5.933622438 | | | KD3678 | 0 | | 0 | | 0 | | 0 | | 0 | | 0 | | 0 | |  |  |
| 086 | M | | 242 | | 146 | | SP2078 | | 1 | | 1 | | 0 | | 0 | | 0.00 | | 0.00 | | 0 | | | LV2877 | 0 | | 0 | | 1 | | 1 | | 35.875 | | 37.027 | | 2.611217352 | | | KD3677 | 0 | | 0 | | 1 | | 0 | | 37.551 | | 38.779 | | 1.145155135 | |  |  |
| 087 | F | | 342 | | 521 | | SP2084 | | 0 | | 1 | | 0 | | 0 | | 0.00 | | 0.00 | | 0 | | | LV2884 | 0 | | 0 | | 1 | | 0 | | 37.055 | | 38.853 | | 1.605393264 | | | KD3684 | 0 | | 0 | | 0 | | 0 | | 0 | | 38.529 | | 0 | |  |  |
| 088 | M | | 211 | | 108 | | SP2076 | | 1 | | 0 | | 0 | | 0 | | 0.00 | | 0.00 | | 0 | | | LV2876 | 0 | | 0 | | 1 | | 1 | | 36.262 | | 36.107 | | 2.908577543 | | | KD3676 | 0 | | 0 | | 0 | | 1 | | 38.081 | | 37.547 | | 1.148279278 | |  |  |
| 089 | F | | 380 | | 735 | | SP2080 | | 0 | | 0 | | 0 | | 0 | | 0.00 | | 0.00 | | 0 | | | LV2881 | 0 | | 0 | | 1 | | 1 | | 35.136 | | 35.572 | | 5.170210018 | | | KD3681 | 0 | | 0 | | 0 | | 0 | | 38.604 | | 0 | | 0 | |  |  |
| 090 | M | | 273 | | 252 | | SP2085 | | 0 | | 0 | | 0 | | 0 | | 0.00 | | 0.00 | | 0 | | | LV2885 | 0 | | 0 | | 1 | | 0 | | 37.134 | | 0 | | 1.52129412 | | | KD3685 | 0 | | 0 | | 0 | | 0 | | 37.999 | | 38.437 | | 0 | |  |  |
| 091 | F | | 331 | | 453 | | SP2086 | | 0 | | 1 | | 0 | | 0 | | 0.00 | | 0.00 | | 0 | | | LV2886 | 0 | | 0 | | 1 | | 0 | | 35.748 | | 38.408 | | 3.91014495 | | | KD3686 | 0 | | 0 | | 0 | | 0 | | 38.941 | | 39.227 | | 0 | |  |  |
| 092 | M | | 254 | | 169 | | SP2079 | | 0 | | 0 | | 0 | | 0 | | 0.00 | | 0.00 | | 0 | | | LV2879 | 0 | | 0 | | 1 | | 1 | | 35.47 | | 37.044 | | 3.171361641 | | | KD3679 | 0 | | 0 | | 0 | | 0 | | 39.118 | | 0 | | 0 | |  |  |
| 093 | F | | 315 | | 396 | | SP2089 | | 0 | | 1 | | 0 | | 0 | | 0.00 | | 0.00 | | 0 | | | LV2889 | 0 | | 0 | | 0 | | 1 | | 0 | | 37.406 | | 1.26402428 | | | KD3689 | 0 | | 0 | | 0 | | 0 | | 0 | | 39.121 | | 0 | |  |  |
| 094 | M | | 258 | | 177 | | SP2083 | | 1 | | 0 | | 0 | | 0 | | 0.00 | | 0.00 | | 0 | | | LV2883 | 0 | | 0 | | 0 | | 0 | | 0 | | 38.451 | | 0 | | | KD3683 | 0 | | 0 | | 0 | | 0 | | 0 | | 39.068 | | 0 | |  |  |
| 095 | F | | 361 | | 583 | | SP2093 | |  | |  | | 0 | | 0 | | 0.00 | | 38.908 | | 0 | | | LV2893 | 0 | | 0 | | 1 | | 1 | | 36.935 | | 37.373 | | 1.517437296 | | | KD3693 | 0 | | 0 | | 0 | | 0 | | 0 | | 0 | | 0 | |  |  |
| 096 | M | | 228 | | 117 | | SP2081 | | 1 | | 0 | | 0 | | 0 | | 0.00 | | 0.00 | | 0 | | | LV2880 | 0 | | 0 | | 1 | | 1 | | 36.19 | | 35.776 | | 3.364975552 | | | KD3680 | 0 | | 0 | | 0 | | 0 | | 38.396 | | 0 | | 0 | |  |  |
| 097 | F | | 373 | | 682 | | SP2092 | |  | |  | | 0 | | 0 | | 0.00 | | 0.00 | | 0 | | | LV2892 | 0 | | 0 | | 1 | | 0 | | 36.058 | | 0 | | 3.165881554 | | | KD3692 | 0 | | 0 | | 0 | | 0 | | 0 | | 0 | | 0 | |  |  |
| 098 | M | | 252 | | 154 | | SP2090 | | 0 | | 0 | | 0 | | 0 | | 0.00 | | 0.00 | | 0 | | | LV2890 | 0 | | 0 | | 0 | | 1 | | 38.043 | | 37.51 | | 1.17758469 | | | KD3690 | 0 | | 0 | | 0 | | 0 | | 39.312 | | 39.127 | | 0 | |  |  |
| 099 | F | | 352 | | 529 | | SP2099 | |  | |  | | 0 | | 0 | | 0.00 | | 37.767 | | 0 | | | LV2899 | 0 | | 0 | | 1 | | 0 | | 37.28 | | 0 | | 1.377292857 | | | KD3699 | 0 | | 0 | | 0 | | 0 | | 0 | | 0 | | 0 | |  |  |
| 100 | M | | 235 | | 149 | | SP2088 | | 0 | | 0 | | 0 | | 0 | | 0.00 | | 0.00 | | 0 | | | LV2888 | 0 | | 0 | | 1 | | 1 | | 36.054 | | 35.271 | | 4.292835403 | | | KD3688 | 0 | | 0 | | 0 | | 0 | | 0 | | 40 | | 0 | |  |  |
| 101 | F | | 354 | | 459 | | SP2098 | |  | |  | | 0 | | 0 | | 0.00 | | 0.00 | | 0 | | | LV2898 | 0 | | 0 | | 1 | | 1 | | 36.927 | | 37.383 | | 1.517808955 | | | KD3698 | 0 | | 0 | | 0 | | 1 | | 40 | | 36.267 | | 2.745821689 | |  |  |
| 102 | M | | 240 | | 154 | | SP2091 | |  | |  | | 0 | | 0 | | 0.00 | | 0.00 | | 0 | | | LV2891 | 0 | | 0 | | 1 | | 1 | | 36.086 | | 37.302 | | 2.231443043 | | | KD3691 | 0 | | 0 | | 0 | | 0 | | 0 | | 38.401 | | 0 | |  |  |
| 103 | F | | 366 | | 684 | | SP2095 | |  | |  | | 0 | | 0 | | 38.87 | | 0.00 | | 0 | | | LV2895 | 0 | | 0 | | 0 | | 1 | | 38.4 | | 37.384 | | 1.28310746 | | | KD3695 | 0 | | 0 | | 0 | | 0 | | 0 | | 0 | | 0 | |  |  |
| 104 | M | | 245 | | 169 | | SP2097 | |  | |  | | 0 | | 0 | | 0.00 | | 0.00 | | 0 | | | LV2897 | 0 | | 0 | | 0 | | 1 | | 38.058 | | 37.665 | | 1.059602693 | | | KD3697 | 0 | | 0 | | 0 | | 0 | | 0 | | 0 | | 0 | |  |  |
| 105 | F | | 345 | | 456 | | SP2104 | |  | |  | | 0 | | 0 | | 0.00 | | 0.00 | | 0 | | | LV2904 | 0 | | 0 | |  | |  | |  | |  | |  | | | KD3704 | 0 | | 0 | | 1 | | 0 | | 37.024 | | 38.216 | | 1.639650387 | |  |  |
| 106 | M | | 280 | | 260 | | SP2096 | |  | |  | | 0 | | 0 | | 0.00 | | 0.00 | | 0 | | | LV2896 | 0 | | 0 | | 1 | | 1 | | 36.672 | | 35.898 | | 2.807136208 | | | KD3696 | 0 | | 0 | | 1 | | 0 | | 37.188 | | 0 | | 1.466357797 | |  |  |
| 107 | F | | 372 | | 670 | | SP2105 | |  | |  | | 0 | | 0 | | 0.00 | | 0.00 | | 0 | | | LV2905 | 0 | | 0 | | 0 | | 0 | | 0 | | 0 | | 0 | | | KD3705 | 0 | | 0 | | 0 | | 0 | | 39.005 | | 0 | | 0 | |  |  |
| 108 | M | | 242 | | 151 | | SP2094 | |  | |  | | 0 | | 0 | | 0.00 | | 0.00 | | 0 | | | LV2894 | 0 | | 0 | | 1 | | 0 | | 37.375 | | 0 | | 1.290997007 | | | KD3694 | 0 | | 0 | | 0 | | 0 | | 0 | | 0 | | 0 | |  |  |
| 109 | F | | 366 | | 732 | | SP2101 | |  | |  | | 0 | | 0 | | 0.00 | | 38.000 | | 0 | | | LV2901 | 0 | | 0 | | 0 | | 0 | | 0 | | 0 | | 0 | | | KD3701 | 0 | | 0 | | 0 | | 0 | | 0 | | 39.089 | | 0 | |  |  |
| 110 | M | | 198 | | 78 | | SP2103 | |  | |  | | 0 | | 0 | | 0.00 | | 0.00 | | 0 | | | LV2903 | 0 | | 0 | | 1 | | 1 | | 37.011 | | 36.686 | | 1.859169165 | | | KD3703 | 0 | | 0 | | 0 | | 1 | | 38.053 | | 37.085 | | 1.572922836 | |  |  |
| 111 | F | | 227 | | 348 | | SP2110 | |  | |  | | 0 | | 0 | | 0.00 | | 0.00 | | 0 | | | LV2910 | 0 | | 0 | | 0 | | 1 | | 37.959 | | 37.197 | | 1.457396584 | | | KD3710 | 0 | | 0 | | 0 | | 0 | | 38.737 | | 0 | | 0 | |  |  |
| 112 | M | | 257 | | 193 | | SP2102 | |  | |  | | 0 | | 0 | | 0.00 | | 37.953 | | 0 | | | LV2902 | 0 | | 0 | | 1 | | 0 | | 37.443 | | 0 | | 1.232567729 | | | KD3702 | 0 | | 0 | | 0 | | 0 | | 38.711 | | 38.647 | | 0 | |  |  |
| 113 | F | | 284 | | 293 | | SP2114 | |  | |  | | 0 | | 0 | | 0.00 | | 0.00 | | 0 | | | LV2914 | 0 | | 0 | | 0 | | 1 | | 0 | | 37.643 | | 1.075599687 | | | KD3714 | 0 | | 0 | | 0 | | 0 | | 0 | | 0 | | 0 | |  |  |
| 114 | M | | 244 | | 141 | | SP2100 | |  | |  | | 0 | | 0 | | 0.00 | | 0.00 | | 0 | | | LV2900 | 0 | | 0 | | 1 | | 0 | | 36.164 | | 0 | | 2.945369572 | | | KD3700 | 0 | | 0 | | 0 | | 0 | | 38.091 | | 0 | | 0 | |  |  |
| 115 | F | | 386 | | 778 | | SP2107 | |  | |  | | 0 | | 0 | | 0.00 | | 0.00 | | 0 | | | LV2907 | 0 | | 0 | | 0 | | 1 | | 0 | | 36.835 | | 1.864908698 | | | KD3707 | 0 | | 0 | | 0 | | 0 | | 38.1 | | 0 | | 0 | |  |  |
| 116 | M | | 248 | | 150 | | SP2109 | |  | |  | | 0 | | 0 | | 39.95 | | 0.00 | | 0 | | | LV2909 | 0 | | 0 | | 1 | | 0 | | 37.473 | | 0 | | 1.20763801 | | | KD3709 | 0 | | 0 | | 0 | | 0 | | 0 | | 0 | | 0 | |  |  |
| 117 | F | | 359 | | 585 | | SP2115 | |  | |  | | 1 | | 1 | | 36.94 | | 36.631 | | 1.94013651 | | | LV2915 | 0 | | 0 | | 0 | | 1 | | 37.862 | | 35.456 | | 4.7705301 | | | KD3715 | 0 | | 0 | | 0 | | 1 | | 37.936 | | 37.356 | | 1.30781239 | |  |  |
| 118 | M | | 250 | | 148 | | SP2106 | |  | |  | | 0 | | 0 | | 0.00 | | 0.00 | | 0 | | | LV2906 | 0 | | 0 | | 0 | | 1 | | 0 | | 37.725 | | 1.01717345 | | | KD3706 | 0 | | 0 | | 1 | | 0 | | 37.382 | | 0 | | 1.284856515 | |  |  |
| 119 | F | | 361 | | 631 | | SP2116 | |  | |  | | 0 | | 0 | | 0.00 | | 0.00 | | 0 | | | LV2916 | 0 | | 0 | | 0 | | 0 | | 0 | | 37.802 | | 0 | | | KD3716 | 0 | | 0 | | 0 | | 0 | | 0 | | 38.901 | | 0 | |  |  |
| 120 | M | | 237 | | 124 | | SP2108 | |  | |  | | 0 | | 0 | | 0.00 | | 0.00 | | 0 | | | LV2908 | 0 | | 0 | | 1 | | 0 | | 36.749 | | 37.835 | | 1.977408645 | | | KD3708 | 0 | | 0 | | 0 | | 0 | | 38.642 | | 39.177 | | 0 | |  |  |
| 121 | F | | 368 | | 659 | | SP2117 | |  | |  | | 0 | | 0 | | 0.00 | | 0.00 | | 0 | | | LV2917 | 0 | | 0 | | 1 | | 0 | | 35.216 | | 37.823 | | 5.617703209 | | | KD3717 | 0 | | 0 | | 0 | | 0 | | 0 | | 0 | | 0 | |  |  |
| 122 | M | | 225 | | 112 | | SP2112 | |  | |  | | 0 | | 0 | | 38.57 | | 0.00 | | 0 | | | LV2912 | 0 | | 0 | | 1 | | 0 | | 37.33 | | 0 | | 1.331178405 | | | KD3712 | 0 | | 0 | | 0 | | 0 | | 0 | | 0 | | 0 | |  |  |
| 123 | F | | 363 | | 526 | | SP2119 | |  | |  | | 0 | | 0 | | 0.00 | | 0.00 | | 0 | | | LV2919 | 0 | | 0 | | 1 | | 1 | | 36.759 | | 36.811 | | 1.929815098 | | | KD3719 | 0 | | 0 | | 0 | | 0 | | 0 | | 0 | | 0 | |  |  |
| 124 | M | | 274 | | 199 | | SP2111 | |  | |  | | 0 | | 0 | | 0.00 | | 0.00 | | 0 | | | LV2911 | 0 | | 0 | | 0 | | 0 | | 0 | | 0 | | 0 | | | KD3711 | 0 | | 0 | | 0 | | 0 | | 0 | | 38.932 | | 0 | |  |  |
| 125 | F | | 349 | | 545 | | SP2123 | | 0 | | 0 | | 0 | | 0 | | 0.00 | | 0.00 | | 0 | | | LV2923 | 0 | | 0 | | 1 | | 0 | | 37.324 | | 0 | | 1.336629579 | | | KD3723 | 0 | | 0 | | 0 | | 0 | | 39.968 | | 37.784 | | 0 | |  |  |
| 126 | M | | 279 | | 248 | | SP2113 | |  | |  | | 0 | | 0 | | 0.00 | | 0.00 | | 0 | | | LV2913 | 0 | | 0 | | 1 | | 0 | | 37.139 | | 0 | | 1.516122119 | | | KD3713 | 0 | | 0 | | 0 | | 0 | | 0 | | 38.793 | | 0 | |  |  |
| 127 | F | | 349 | | 505 | | SP2124 | | 0 | | 0 | | 0 | | 0 | | 0.00 | | 0.00 | | 0 | | | LV2924 | 0 | | 0 | | 1 | | 0 | | 36.808 | | 0 | | 1.899521404 | | | KD3724 | 0 | | 0 | | 0 | | 1 | | 38.326 | | 35.469 | | 4.72847647 | |  |  |
| 128 | M | | 240 | | 143 | | SP2121 | | 0 | | 0 | | 0 | | 0 | | 0.00 | | 0.00 | | 0 | | | LV2921 | 0 | | 0 | | 1 | | 1 | | 36.871 | | 35.322 | | 3.523076463 | | | KD3721 | 0 | | 0 | | 0 | | 0 | | 39.883 | | 37.902 | | 0 | |  |  |
| 129 | F | | 315 | | 417 | | SP2133 | | 0 | | 0 | | 0 | | 0 | | 0.00 | | 0.00 | | 0 | | | LV2933 | 0 | | 0 | | 1 | | 1 | | 35.534 | | 36.212 | | 3.687168748 | | | KD3733 | 0 | | 0 | | 1 | | 0 | | 37.133 | | 40 | | 1.522330636 | |  |  |
| 130 | M | | 241 | | 139 | | SP2122 | | 0 | | 0 | | 0 | | 0 | | 0.00 | | 39.442 | | 0 | | | LV2922 | 0 | | 0 | | 1 | | 1 | | 37.357 | | 36.884 | | 1.55530897 | | | KD3722 | 0 | | 0 | | 0 | | 0 | | 38.217 | | 0 | | 0 | |  |  |
| 131 | F | | 344 | | 512 | | SP2129 | | 0 | | 0 | | 0 | | 0 | | 0.00 | | 0.00 | | 0 | | | LV2929 | 0 | | 0 | | 1 | | 1 | | 35.814 | | 36.559 | | 2.994432808 | | | KD3729 | 0 | | 0 | | 0 | | 0 | | 38.047 | | 0 | | 0 | |  |  |
| 132 | M | | 245 | | 142 | | SP2118 | |  | |  | | 0 | | 0 | | 0.00 | | 0.00 | | 0 | | | LV2918 | 0 | | 0 | | 1 | | 0 | | 35.571 | | 38.001 | | 4.411127067 | | | KD3718 | 0 | | 0 | | 0 | | 0 | | 0 | | 0 | | 0 | |  |  |
| 133 | F | | 382 | | 672 | | SP2125 | | 0 | | 0 | | 0 | | 0 | | 0.00 | | 0.00 | | 0 | | | LV2925 | 0 | | 0 | | 1 | | 0 | | 37.348 | | 37.981 | | 1.314957912 | | | KD3725 |  | |  | |  | |  | |  | |  | |  | |  |  |
| 134 | M | | 299 | | 272 | | SP2120 | |  | |  | | 0 | | 0 | | 0.00 | | 0.00 | | 0 | | | LV2920 | 0 | | 0 | | 0 | | 1 | | 37.922 | | 36.745 | | 1.982803291 | | | KD3720 | 0 | | 0 | | 0 | | 0 | | 0 | | 0 | | 0 | |  |  |
| 136 | M | | 246 | | 152 | | SP2128 | | 0 | | 0 | | 0 | | 0 | | 0.00 | | 0.00 | | 0 | | | LV2928 | 0 | | 0 | | 1 | | 1 | | 37.083 | | 34.88 | | 4.31869681 | | | KD3728 | 0 | | 0 | | 0 | | 0 | | 0 | | 0 | | 0 | |  |  |
| 137 | F | | 314 | | 383 | | SP2130 | | 0 | | 0 | | 0 | | 0 | | 0.00 | | 0.00 | | 0 | | | LV2930 | 0 | | 0 | | 1 | | 0 | | 36.367 | | 38 | | 2.565028934 | | | KD3730 | 0 | | 0 | | 0 | | 0 | | 38.603 | | 0 | | 0 | |  |  |
| 138 | M | | 224 | | 124 | | SP2127 | | 0 | | 0 | | 0 | | 0 | | 0.00 | | 0.00 | | 0 | | | LV2927 | 0 | | 0 | | 0 | | 0 | | 0 | | 0 | | 0 | | | KD3727 | 0 | | 0 | | 0 | | 0 | | 38.416 | | 0 | | 0 | |  |  |
| 139 | F | | 331 | | 474 | | SP2131 | | 0 | | 0 | | 1 | | 0 | | 36.50 | | 0.00 | | 2.346077671 | | | LV2931 | 0 | | 0 | | 1 | | 0 | | 37.593 | | 0 | | 1.112860425 | | | KD3731 | 0 | | 0 | | 0 | | 0 | | 0 | | 38.999 | | 0 | |  |  |
| 140 | M | | 219 | | 118 | | SP2126 | | 1 | | 0 | | 0 | | 0 | | 0.00 | | 0.00 | | 0 | | | LV2926 | 0 | | 0 | | 1 | | 1 | | 37.359 | | 34.865 | | 4.219996128 | | | KD3726 | 0 | | 0 | | 0 | | 0 | | 0 | | 40.216 | | 0 | |  |  |
| 141 | F | | 312 | | 432 | | SP2141 | | 0 | | 0 | | 0 | | 1 | | 0.00 | | 37.325 | | 1.335719502 | | | LV2941 | 0 | | 0 | | 0 | | 1 | | 0 | | 36.304 | | 2.677489078 | | | KD3741 | 0 | | 0 | | 0 | | 0 | | 0 | | 0 | | 0 | |  |  |
| 143 | F | | 365 | | 521 | | SP2136 | | 0 | | 0 | | 0 | | 0 | | 0.00 | | 0.00 | | 0 | | | LV2936 | 0 | | 0 | | 1 | | 1 | | 35.856 | | 37.402 | | 2.450156852 | | | KD3736 | 0 | | 0 | | 0 | | 0 | | 0 | | 38.829 | | 0 | |  |  |
| 144 | M | | 237 | | 128 | | SP2132 | | 0 | | 0 | | 0 | | 0 | | 0.00 | | 0.00 | | 0 | | | LV2932 | 0 | | 0 | | 1 | | 1 | | 37.008 | | 36.223 | | 2.24348614 | | | KD3732 | 0 | | 0 | | 0 | | 0 | | 38.311 | | 0 | | 0 | |  |  |
| 145 | F | | 342 | | 471 | | SP2139 | | 0 | | 0 | | 0 | | 0 | | 0.00 | | 38.347 | | 0 | | | LV2939 | 0 | | 0 | | 0 | | 1 | | 0 | | 35.782 | | 3.820635756 | | | KD3739 | 0 | | 0 | | 1 | | 0 | | 36.773 | | 0 | | 1.9453476 | |  |  |
| 146 | M | | 254 | | 167 | | SP2135 | | 0 | | 0 | | 1 | | 0 | | 37.20 | | 0.00 | | 1.45046463 | | | LV2935 | 0 | | 0 | | 1 | | 1 | | 36.838 | | 35.755 | | 2.876324371 | | | KD3735 | 0 | | 0 | | 0 | | 0 | | 0 | | 0 | | 0 | |  |  |
| 147 | F | | 362 | | 618 | | SP2144 | | 0 | | 0 | | 0 | | 0 | | 0.00 | | 0.00 | | 0 | | | LV2944 | 0 | | 0 | | 0 | | 1 | | 0 | | 36.539 | | 2.281468939 | | | KD3744 | 0 | | 0 | | 0 | | 0 | | 0 | | 0 | | 0 | |  |  |
| 148 | M | | 182 | | 69 | | SP2134 | | 0 | | 0 | | 0 | | 0 | | 0.00 | | 0.00 | | 0 | | | LV2934 | 0 | | 0 | | 0 | | 1 | | 37.925 | | 35.062 | | 6.238958371 | | | KD3734 | 0 | | 0 | | 0 | | 0 | | 0 | | 0 | | 0 | |  |  |
| 149 | F | | 363 | | 667 | | SP2171 | | 0 | | 0 | | 0 | | 0 | | 0.00 | | 0.00 | | 0 | | | LV2971 | 0 | | 0 | | 1 | | 0 | | 37.53 | | 0 | | 1.161652251 | | | KD3771 | 0 | | 0 | | 0 | | 0 | | 0 | | 0 | | 0 | |  |  |
| 150 | M | | 248 | | 147 | | SP2138 | | 0 | | 0 | | 0 | | 0 | | 0.00 | | 0.00 | | 0 | | | LV2938 | 0 | | 0 | | 1 | | 1 | | 36.302 | | 36.256 | | 2.723804987 | | | KD3738 | 0 | | 0 | | 0 | | 0 | | 0 | | 38.959 | | 0 | |  |  |
| 151 | F | | 356 | | 495 | | SP2147 | | 0 | | 0 | | 0 | | 1 | | 0.00 | | 37.451 | | 1.225869918 | | | LV2947 | 0 | | 0 | | 1 | | 1 | | 35.999 | | 37.416 | | 2.275569162 | | | KD3747 | 0 | | 0 | | 1 | | 0 | | 36.877 | | 0 | | 1.812316098 | |  |  |
| 152 | M | | 251 | | 178 | | SP2140 | | 0 | | 0 | | 0 | | 0 | | 0.00 | | 0.00 | | 0 | | | LV2940 | 0 | | 0 | | 1 | | 1 | | 36.495 | | 35.683 | | 3.219009983 | | | KD3740 | 0 | | 0 | | 0 | | 0 | | 0 | | 0 | | 0 | |  |  |
| 153 | F | | 338 | | 490 | | SP2150 | | 0 | | 0 | | 0 | | 0 | | 0.00 | | 0.00 | | 0 | | | LV2950 | 0 | | 0 | | 1 | | 1 | | 37.379 | | 36.761 | | 1.624398591 | | | KD3750 | 0 | | 0 | | 0 | | 0 | | 0 | | 0 | | 0 | |  |  |
| 154 | M | | 258 | | 179 | | SP2137 | | 0 | | 0 | | 1 | | 0 | | 36.41 | | 0.00 | | 2.496090017 | | | LV2937 | 0 | | 0 | | 1 | | 1 | | 37.01 | | 37.618 | | 1.374715737 | | | KD3737 | 0 | | 0 | | 0 | | 0 | | 0 | | 39.919 | | 0 | |  |  |
| 155 | F | | 360 | | 590 | | SP2162 | | 0 | | 0 | | 0 | | 0 | | 0.00 | | 0.00 | | 0 | | | LV2962 | 0 | | 0 | | 0 | | 1 | | 0 | | 36.632 | | 2.141436013 | | | KD3762 | 0 | | 0 | | 0 | | 0 | | 0 | | 0 | | 0 | |  |  |
| 156 | M | | 245 | | 159 | | SP2142 | | 0 | | 0 | | 0 | | 0 | | 0.00 | | 0.00 | | 0 | | | LV2942 | 0 | | 0 | | 1 | | 0 | | 36.103 | | 0 | | 3.070319948 | | | KD3742 | 0 | | 0 | | 1 | | 1 | | 36.88 | | 35.386 | | 3.406051344 | |  |  |
| 157 | F | | 324 | | 465 | | SP2154 | | 0 | | 0 | | 0 | | 0 | | 0.00 | | 0.00 | | 0 | | | LV2954 | 0 | | 0 | | 0 | | 1 | | 38.079 | | 37.086 | | 1.571851873 | | | KD3754 | 0 | | 0 | | 0 | | 0 | | 0 | | 0 | | 0 | |  |  |
| 158 | M | | 215 | | 107 | | SP2145 | | 0 | | 0 | | 0 | | 0 | | 0.00 | | 0.00 | | 0 | | | LV2945 | 0 | | 0 | | 1 | | 0 | | 37.159 | | 0 | | 1.495609348 | | | KD3745 | 0 | | 0 | | 0 | | 1 | | 0 | | 37.673 | | 1.053844779 | |  |  |
| 159 | F | | 362 | | 648 | | SP2152 | | 0 | | 0 | | 0 | | 0 | | 38.60 | | 0.00 | | 0 | | | LV2952 | 0 | | 0 | | 1 | | 1 | | 37.322 | | 35.887 | | 2.447695921 | | | KD3752 | 0 | | 0 | | 0 | | 0 | | 0 | | 0 | | 0 | |  |  |
| 160 | M | | 247 | | 156 | | SP2143 | | 0 | | 0 | | 0 | | 0 | | 0.00 | | 0.00 | | 0 | | | LV2943 | 0 | | 0 | | 1 | | 0 | | 37.222 | | 0 | | 1.432790626 | | | KD3743 | 0 | | 0 | | 0 | | 0 | | 37.853 | | 0 | | 0 | |  |  |
| 161 | F | | 312 | | 421 | | SP2160 | | 0 | | 0 | | 0 | | 0 | | 0.00 | | 0.00 | | 0 | | | LV2960 | 0 | | 0 | | 0 | | 1 | | 0 | | 35.748 | | 3.91014495 | | | KD3760 | 0 | | 0 | | 0 | | 0 | | 0 | | 0 | | 0 | |  |  |
| 162 | M | | 218 | | 110 | | SP2149 | | 0 | | 0 | | 0 | | 0 | | 0.00 | | 0.00 | | 0 | | | LV2949 | 0 | | 0 | | 1 | | 0 | | 35.917 | | 0 | | 3.484998143 | | | KD3749 | 0 | | 0 | | 0 | | 0 | | 0 | | 0 | | 0 | |  |  |
| 163 | F | | 324 | | 454 | | SP2169 | | 0 | | 0 | | 0 | | 0 | | 0.00 | | 0.00 | | 0 | | | LV2969 | 0 | | 0 | | 0 | | 0 | | 0 | | 38.113 | | 0 | | | KD3769 | 0 | | 0 | | 0 | | 0 | | 39.102 | | 0 | | 0 | |  |  |
| 164 | M | | 225 | | 121 | | SP2146 | | 0 | | 0 | | 0 | | 0 | | 38.00 | | 0.00 | | 0 | | | LV2946 | 0 | | 0 | | 1 | | 1 | | 36.714 | | 36.958 | | 1.870076632 | | | KD3746 | 0 | | 0 | | 0 | | 1 | | 0 | | 36.871 | | 1.81973753 | |  |  |
| 165 | F | | 354 | | 532 | | SP2156 | | 0 | | 0 | | 0 | | 0 | | 0.00 | | 0.00 | | 0 | | | LV2956 | 0 | | 0 | | 1 | | 1 | | 36.018 | | 36.881 | | 2.530352292 | | | KD3756 | 0 | | 0 | | 0 | | 0 | | 0 | | 0 | | 0 | |  |  |
| 166 | M | | 227 | | 118 | | SP2148 | | 0 | | 0 | | 0 | | 0 | | 0.00 | | 0.00 | | 0 | | | LV2948 | 0 | | 0 | | 1 | | 1 | | 35.686 | | 36.853 | | 2.960492734 | | | KD3748 | 0 | | 0 | | 1 | | 0 | | 36.815 | | 37.8 | | 1.890486529 | |  |  |
| 167 | F | | 348 | | 542 | | SP2170 | | 0 | | 0 | | 0 | | 0 | | 0.00 | | 0.00 | | 0 | | | LV2970 | 0 | | 0 | | 1 | | 1 | | 36.608 | | 36.806 | | 2.039419743 | | | KD3770 | 0 | | 0 | | 0 | | 0 | | 0 | | 0 | | 0 | |  |  |
| 168 | M | | 253 | | 161 | | SP2151 | | 0 | | 0 | | 0 | | 0 | | 0.00 | | 0.00 | | 0 | | | LV2951 | 0 | | 0 | | 1 | | 0 | | 35.56 | | 0 | | 4.444300082 | | | KD3751 | 0 | | 0 | | 1 | | 0 | | 35.819 | | 0 | | 3.725555285 | |  |  |
| 169 | F | | 343 | | 488 | | SP2168 | | 0 | | 0 | | 0 | | 0 | | 0.00 | | 0.00 | | 0 | | | LV2968 | 0 | | 0 | | 1 | | 1 | | 37.227 | | 37.505 | | 1.30476067 | | | KD3768 | 0 | | 0 | | 0 | | 0 | | 0 | | 38.678 | | 0 | |  |  |
| 170 | M | | 225 | | 123 | | SP2153 | | 0 | | 0 | | 0 | | 0 | | 0.00 | | 0.00 | | 0 | | | LV2953 | 0 | | 0 | | 1 | | 0 | | 37.423 | | 0 | | 1.249472797 | | | KD3753 | 0 | | 0 | | 0 | | 0 | | 0 | | 0 | | 0 | |  |  |
| 171 | F | | 355 | | 563 | | SP2165 | | 0 | | 0 | | 0 | | 0 | | 0.00 | | 0.00 | | 0 | | | LV2965 | 0 | | 0 | | 1 | | 1 | | 36.766 | | 36.744 | | 1.969399456 | | | KD3765 | 0 | | 0 | | 0 | | 0 | | 0 | | 0 | | 0 | |  |  |
| 172 | M | | 263 | | 206 | | SP2155 | | 0 | | 0 | | 0 | | 0 | | 0.00 | | 0.00 | | 0 | | | LV2955 | 0 | | 0 | | 1 | | 0 | | 35.056 | | 38 | | 6.264506895 | | | KD3755 | 0 | | 0 | | 1 | | 0 | | 37.409 | | 0 | | 1.261444112 | |  |  |
| 173 | F | | 381 | | 787 | | SP2167 | | 0 | | 0 | | 0 | | 0 | | 0.00 | | 0.00 | | 0 | | | LV2967 | 0 | | 0 | | 0 | | 1 | | 37.779 | | 36.583 | | 2.21411071 | | | KD3767 | 0 | | 0 | | 0 | | 0 | | 0 | | 0 | | 0 | |  |  |
| 174 | M | | 250 | | 170 | | SP2159 | | 1 | | 1 | | 0 | | 0 | | 0.00 | | 0.00 | | 0 | | | LV2959 | 0 | | 0 | | 0 | | 0 | | 0 | | 0 | | 0 | | | KD3759 | 0 | | 0 | | 0 | | 1 | | 38.075 | | 35.842 | | 3.667647579 | |  |  |
| 175 | F | | 383 | | 710 | | SP2166 | | 0 | | 0 | | 0 | | 0 | | 0.00 | | 0.00 | | 0 | | | LV2966 | 0 | | 0 | | 0 | | 1 | | 38.061 | | 37.723 | | 1.01856 | | | KD3766 | 0 | | 0 | | 0 | | 0 | | 0 | | 0 | | 0 | |  |  |
| 176 | M | | 268 | | 207 | | SP2158 | | 0 | | 0 | | 0 | | 0 | | 0.00 | | 0.00 | | 0 | | | LV2958 | 0 | | 0 | | 1 | | 1 | | 37.417 | | 36.116 | | 2.148921779 | | | KD3758 | 0 | | 0 | | 1 | | 1 | | 36.689 | | 36.968 | | 1.881644954 | |  |  |
| 177 | F | | 351 | | 506 | | SP2172 | | 0 | | 0 | | 0 | | 0 | | 0.00 | | 0.00 | | 0 | | | LV2972 | 0 | | 0 | | 0 | | 1 | | 38.193 | | 34.309 | | 10.41957393 | | | KD3772 | 0 | | 0 | | 0 | | 0 | | 0 | | 0 | | 0 | |  |  |
| 178 | M | | 222 | | 113 | | SP2157 | | 0 | | 0 | | 0 | | 0 | | 0.00 | | 0.00 | | 0 | | | LV2957 | 0 | | 0 | | 1 | | 1 | | 37.291 | | 36.542 | | 1.821912219 | | | KD3757 | 0 | | 0 | | 0 | | 0 | | 0 | | 0 | | 0 | |  |  |
| 179 | F | | 330 | | 372 | | SP2173 | | 0 | | 0 | | 0 | | 0 | | 0.00 | | 0.00 | | 0 | | | LV2973 | 0 | | 0 | | 1 | | 1 | | 36.768 | | 36.412 | | 2.219793899 | | | KD3773 | 0 | | 0 | | 0 | | 0 | | 0 | | 0 | | 0 | |  |  |
| 180 | M | | 253 | | 179 | | SP2164 | | 0 | | 0 | | 0 | | 0 | | 0.00 | | 0.00 | | 0 | | | LV2964 | 0 | | 0 | | 1 | | 1 | | 33.997 | | 36.512 | | 7.605227396 | | | KD3764 | 0 | | 0 | | 0 | | 0 | | 0 | | 0 | | 0 | |  |  |
| 181 | F | | 313 | | 336 | | SP2181 | | 0 | | 0 | | 0 | | 0 | | 0.00 | | 0.00 | | 0 | | | LV2981 | 0 | | 0 | | 1 | | 1 | | 36.146 | | 36.312 | | 2.822320659 | | | KD3781 | 0 | | 0 | | 0 | | 0 | | 0 | | 0 | | 0 | |  |  |
| 182 | M | | 255 | | 170 | | SP2163 | | 0 | | 0 | | 0 | | 0 | | 0.00 | | 0.00 | | 0 | | | LV2963 | 0 | | 0 | | 0 | | 1 | | 0 | | 37.512 | | 1.175981662 | | | KD3763 | 0 | | 0 | | 0 | | 0 | | 0 | | 0 | | 0 | |  |  |
| 183 | F | | 357 | | 638 | | SP2175 | | 0 | | 0 | | 0 | | 0 | | 0.00 | | 0.00 | | 0 | | | LV2975 | 0 | | 0 | | 1 | | 1 | | 36.816 | | 34.828 | | 4.603069176 | | | KD3775 | 0 | | 0 | | 0 | | 0 | | 0 | | 38 | | 0 | |  |  |
| 184 | M | | 225 | | 117 | | SP2161 | | 0 | | 0 | | 0 | | 0 | | 0.00 | | 0.00 | | 0 | | | LV2961 | 0 | | 0 | | 1 | | 1 | | 36.827 | | 35.841 | | 2.772622263 | | | KD3761 | 0 | | 0 | | 0 | | 0 | | 0 | | 0 | | 0 | |  |  |
| 185 | F | | 353 | | 621 | | SP2178 | | 0 | | 0 | | 0 | | 0 | | 0.00 | | 0.00 | | 0 | | | LV2978 | 0 | | 0 | | 1 | | 0 | | 36.908 | | 37.802 | | 1.77445148 | | | KD3778 | 0 | | 0 | | 0 | | 1 | | 0 | | 36.13 | | 3.014373181 | |  |  |
| 186 | M | | 253 | | 173 | | SP2174 | | 0 | | 0 | | 0 | | 0 | | 0.00 | | 0.00 | | 0 | | | LV2974 | 0 | | 0 | | 1 | | 1 | | 36.797 | | 37.549 | | 1.530261262 | | | KD3774 | 0 | | 0 | | 1 | | 0 | | 37.735 | | 38.747 | | 1.010268959 | |  |  |
| 187 | F | | 349 | | 576 | | SP2188 | | 0 | | 0 | | 0 | | 0 | | 0.00 | | 0.00 | | 0 | | | LV2988 | 0 | | 0 | | 1 | | 1 | | 37.006 | | 36.331 | | 2.144288241 | | | KD3788 | 0 | | 0 | | 0 | | 0 | | 0 | | 0 | | 0 | |  |  |
| 188 | M | | 235 | | 139 | | SP2177 | | 0 | | 0 | | 0 | | 0 | | 0.00 | | 0.00 | | 0 | | | LV2977 | 0 | | 0 | | 1 | | 0 | | 35.34 | | 39.084 | | 5.162731193 | | | KD3777 | 0 | | 0 | | 0 | | 0 | | 0 | | 0 | | 0 | |  |  |
| 189 | F | | 370 | | 749 | | SP2183 | | 1 | | 0 | | 0 | | 0 | | 0.00 | | 0.00 | | 0 | | | LV2983 | 0 | | 0 | | 1 | | 1 | | 35.739 | | 36.237 | | 3.36834614 | | | KD3783 | 0 | | 0 | | 0 | | 0 | | 0 | | 0 | | 0 | |  |  |
| 190 | M | | 234 | | 128 | | SP2176 | | 0 | | 0 | | 0 | | 0 | | 0.00 | | 0.00 | | 0 | | | LV2976 | 0 | | 0 | | 1 | | 1 | | 34.943 | | 35.747 | | 5.339251626 | | | KD3776 | 0 | | 0 | | 0 | | 0 | | 0 | | 38.532 | | 0 | |  |  |
| 191 | F | | 340 | | 458 | | SP2185 | | 0 | | 0 | | 0 | | 0 | | 0.00 | | 0.00 | | 0 | | | LV2985 | 0 | | 0 | | 1 | | 1 | | 35.921 | | 36.015 | | 3.367745049 | | | KD3785 | 0 | | 0 | | 0 | | 0 | | 0 | | 0 | | 0 | |  |  |
| 192 | M | | 228 | | 111 | | SP2179 | | 0 | | 0 | | 0 | | 0 | | 0.00 | | 0.00 | | 0 | | | LV2979 | 0 | | 0 | | 1 | | 0 | | 37.535 | | 37.987 | | 1.157702937 | | | KD3779 | 0 | | 0 | | 0 | | 0 | | 0 | | 0 | | 0 | |  |  |
| 193 | F | | 356 | | 551 | | SP2190 | | 0 | | 0 | | 0 | | 0 | | 0.00 | | 0.00 | | 0 | | | LV2990 | 0 | | 0 | | 1 | | 1 | | 35.549 | | 36.489 | | 3.41911288 | | | KD3790 | 0 | | 0 | | 0 | | 0 | | 38 | | 0 | | 0 | |  |  |
| 195 | F | | 311 | | 360 | | SP2196 | | 0 | | 0 | | 0 | | 0 | | 0.00 | | 0.00 | | 0 | | | LV2996 | 0 | | 0 | | 0 | | 1 | | 0 | | 36.77 | | 1.949326628 | | | KD3796 | 0 | | 0 | | 0 | | 0 | | 0 | | 0 | | 0 | |  |  |
| 196 | M | | 227 | | 142 | | SP2180 | | 0 | | 0 | | 0 | | 0 | | 0.00 | | 0.00 | | 0 | | | LV2980 | 0 | | 0 | | 1 | | 1 | | 35.816 | | 36.318 | | 3.192627412 | | | KD3780 | 0 | | 0 | | 0 | | 0 | | 0 | | 0 | | 0 | |  |  |
| 197 | F | | 355 | | 535 | | SP2208 | | 0 | | 0 | | 0 | | 0 | | 0.00 | | 0.00 | | 0 | | | LV3008 | 0 | | 0 | | 1 | | 1 | | 37.741 | | 37.673 | | 1.02999678 | | | KD3808 | 0 | | 0 | | 0 | | 0 | | 0 | | 0 | | 0 | |  |  |
| 198 | M | | 264 | | 195 | | SP2182 | | 0 | | 0 | | 0 | | 0 | | 0.00 | | 0.00 | | 0 | | | LV2982 | 0 | | 0 | | 1 | | 1 | | 35.208 | | 35.17 | | 5.722446917 | | | KD3782 | 0 | | 0 | | 0 | | 0 | | 0 | | 38.808 | | 0 | |  |  |
| 199 | F | | 367 | | 680 | | SP2198 | | 0 | | 0 | | 0 | | 0 | | 0.00 | | 0.00 | | 0 | | | LV2998 | 1 | | 0 | | 1 | | 0 | | 36.53 | | 38 | | 2.295497193 | | | KD3798 | 0 | | 0 | | 0 | | 0 | | 0 | | 0 | | 0 | |  |  |
| 200 | M | | 243 | | 146 | | SP2184 | | 0 | | 0 | | 0 | | 0 | | 0.00 | | 0.00 | | 0 | | | LV2984 | 0 | | 0 | | 1 | | 1 | | 36.791 | | 35.89 | | 2.735661562 | | | KD3784 | 0 | | 0 | | 1 | | 0 | | 36.389 | | 38.688 | | 2.526880213 | |  |  |
| 201 | F | | 366 | | 667 | | SP2193 | | 0 | | 0 | | 0 | | 0 | | 0.00 | | 0.00 | | 0 | | | LV2993 | 0 | | 0 | | 1 | | 0 | | 37.66 | | 0 | | 1.063217354 | | | KD3793 | 0 | | 0 | | 0 | | 0 | | 0 | | 0 | | 0 | |  |  |
| 202 | M | | 225 | | 129 | | SP2186 | | 0 | | 0 | | 0 | | 0 | | 0.00 | | 0.00 | | 0 | | | LV2986 | 0 | | 0 | | 1 | | 1 | | 37.744 | | 36.721 | | 1.509788319 | | | KD3786 | 0 | | 0 | | 0 | | 0 | | 0 | | 0 | | 0 | |  |  |
| 203 | F | | 367 | | 628 | | SP2201 | | 0 | | 0 | | 0 | | 0 | | 0.00 | | 0.00 | | 0 | | | LV3001 | 0 | | 0 | | 1 | | 1 | | 37.405 | | 36.414 | | 1.874551567 | | | KD3801 | 0 | | 0 | | 0 | | 0 | | 0 | | 0 | | 0 | |  |  |
| 204 | M | | 221 | | 108 | | SP2187 | | 0 | | 0 | | 0 | | 0 | | 0.00 | | 0.00 | | 0 | | | LV2987 | 0 | | 0 | | 1 | | 1 | | 35.791 | | 36.196 | | 3.339577836 | | | KD3787 | 0 | | 0 | | 0 | | 0 | | 0 | | 39.08 | | 0 | |  |  |
| 205 | F | | 370 | | 762 | | SP2203 | | 0 | | 0 | | 0 | | 0 | | 0.00 | | 0.00 | | 0 | | | LV3003 | 0 | | 0 | | 1 | | 1 | | 34.817 | | 37.595 | | 4.241655058 | | | KD3803 | 0 | | 0 | | 0 | | 0 | | 0 | | 0 | | 0 | |  |  |
| 206 | M | | 244 | | 165 | | SP2189 | | 0 | | 0 | | 0 | | 0 | | 0.00 | | 0.00 | | 0 | | | LV2989 | 0 | | 0 | | 1 | | 1 | | 36.128 | | 36.958 | | 2.366760789 | | | KD3789 | 0 | | 0 | | 0 | | 0 | | 0 | | 0 | | 0 | |  |  |
| 207 | F | | 372 | | 662 | | SP2206 | | 0 | | 0 | | 0 | | 0 | | 0.00 | | 0.00 | | 0 | | | LV3006 | 0 | | 0 | | 1 | | 0 | | 36.765 | | 0 | | 1.955976436 | | | KD3806 | 0 | | 0 | | 0 | | 0 | | 0 | | 0 | | 0 | |  |  |
| 208 | M | | 260 | | 186 | | SP2191 | | 0 | | 0 | | 0 | | 0 | | 0.00 | | 0.00 | | 0 | | | LV2991 | 0 | | 0 | | 1 | | 1 | | 36.445 | | 36.308 | | 2.551259698 | | | KD3979 | 0 | | 0 | | 0 | | 0 | | 0 | | 0 | | 0 | |  |  |
| 209 | F | | 344 | | 522 | | SP2214 | | 0 | | 0 | | 0 | | 0 | | 0.00 | | 0.00 | | 0 | | | LV3014 | 0 | | 0 | | 0 | | 1 | | 0 | | 37.211 | | 1.443565647 | | | KD3814 | 0 | | 0 | | 0 | | 0 | | 0 | | 0 | | 0 | |  |  |
| 210 | M | | 233 | | 131 | | SP2192 | | 0 | | 0 | | 0 | | 0 | | 0.00 | | 0.00 | | 0 | | | LV2992 | 0 | | 0 | | 1 | | 1 | | 37.371 | | 37.497 | | 1.241288398 | | | KD3792 | 0 | | 0 | | 0 | | 0 | | 38.817 | | 0 | | 0 | |  |  |
| 211 | F | | 331 | | 437 | | SP2218 | | 0 | | 0 | | 0 | | 0 | | 0.00 | | 0.00 | | 0 | | | LV3018 | 0 | | 0 | | 1 | | 0 | | 36.97 | | 0 | | 1.701079026 | | | KD3818 | 0 | | 0 | | 0 | | 0 | | 0 | | 0 | | 0 | |  |  |
| 212 | M | | 275 | | 237 | | SP2194 | | 0 | | 0 | | 0 | | 0 | | 0.00 | | 0.00 | | 0 | | | LV2994 | 0 | | 0 | | 0 | | 1 | | 38.268 | | 36.362 | | 2.57377911 | | | KD3794 | 0 | | 0 | | 0 | | 0 | | 0 | | 0 | | 0 | |  |  |
| 213 | F | | 350 | | 502 | | SP2216 | | 0 | | 0 | | 0 | | 0 | | 0.00 | | 0.00 | | 0 | | | LV3016 | 0 | | 0 | | 1 | | 1 | | 36.835 | | 37.071 | | 1.726450927 | | | KD3816 | 0 | | 0 | | 0 | | 0 | | 0 | | 0 | | 0 | |  |  |
| 214 | M | | 253 | | 179 | | SP2195 | | 0 | | 0 | | 0 | | 0 | | 0.00 | | 0.00 | | 0 | | | LV2995 | 0 | | 0 | | 0 | | 1 | | 0 | | 36.574 | | 2.227724794 | | | KD3795 | 0 | | 0 | | 0 | | 0 | | 0 | | 0 | | 0 | |  |  |
| 216 | M | | 246 | | 143 | | SP2207 | | 0 | | 0 | | 0 | | 0 | | 0.00 | | 0.00 | | 0 | | | LV3007 | 0 | | 0 | | 0 | | 0 | | 37.905 | | 38.011 | | 0 | | | KD3807 | 0 | | 0 | | 0 | | 0 | | 0 | | 0 | | 0 | |  |  |
| 217 | F | | 347 | | 527 | | SP2219 | | 0 | | 0 | | 0 | | 0 | | 0.00 | | 0.00 | | 0 | | | LV3019 | 0 | | 0 | | 1 | | 0 | | 36.927 | | 38.184 | | 1.75163622 | | | KD3819 | 0 | | 0 | | 0 | | 0 | | 0 | | 0 | | 0 | |  |  |
| 218 | M | | 272 | | 250 | | SP2197 | | 0 | | 0 | | 0 | | 0 | | 0.00 | | 0.00 | | 0 | | | LV2997 | 0 | | 0 | | 1 | | 1 | | 37.32 | | 35.404 | | 3.141397122 | | | KD3797 | 0 | | 0 | | 0 | | 0 | | 0 | | 0 | | 0 | |  |  |
| 219 | F | | 365 | | 568 | | SP2217 | | 0 | | 0 | | 0 | | 0 | | 0.00 | | 0.00 | | 0 | | | LV3017 | 0 | | 0 | | 0 | | 0 | | 0 | | 0 | | 0 | | | KD3817 | 0 | | 0 | | 0 | | 0 | | 0 | | 0 | | 0 | |  |  |
| 220 | M | | 242 | | 166 | | SP2200 | | 0 | | 0 | | 0 | | 0 | | 0.00 | | 0.00 | | 0 | | | LV3000 | 0 | | 0 | | 1 | | 1 | | 37.409 | | 36.968 | | 1.482420974 | | | KD3800 | 0 | | 0 | | 0 | | 0 | | 0 | | 0 | | 0 | |  |  |
| 221 | F | | 335 | | 486 | | SP2211 | | 0 | | 0 | | 0 | | 0 | | 0.00 | | 0.00 | | 0 | | | LV3011 | 0 | | 0 | | 1 | | 1 | | 36.783 | | 36.487 | | 2.147931814 | | | KD3811 | 0 | | 0 | | 0 | | 0 | | 0 | | 0 | | 0 | |  |  |
| 222 | M | | 215 | | 108 | | SP2199 | | 0 | | 0 | | 0 | | 0 | | 0.00 | | 0.00 | | 0 | | | LV2999 | 0 | | 0 | | 0 | | 1 | | 38.166 | | 36.603 | | 2.184154322 | | | KD3799 | 0 | | 0 | | 0 | | 0 | | 39.042 | | 39.119 | | 0 | |  |  |
| 223 | F | | 364 | | 643 | | SP2215 | | 0 | | 0 | | 0 | | 0 | | 0.00 | | 0.00 | | 0 | | | LV3015 | 0 | | 0 | | 0 | | 1 | | 0 | | 37.715 | | 1.024125128 | | | KD3815 | 0 | | 0 | | 0 | | 0 | | 0 | | 0 | | 0 | |  |  |
| 224 | M | | 267 | | 197 | | SP2202 | | 0 | | 0 | | 0 | | 0 | | 0.00 | | 0.00 | | 0 | | | LV3002 | 0 | | 0 | | 1 | | 0 | | 37.193 | | 0 | | 1.461372565 | | | KD3802 | 0 | | 0 | | 0 | | 0 | | 0 | | 0 | | 0 | |  |  |
| 225 | F | | 324 | | 410 | | SP2220 | | 0 | | 0 | | 0 | | 0 | | 0.00 | | 0.00 | | 0 | | | LV3020 | 0 | | 0 | | 0 | | 1 | | 0 | | 37.293 | | 1.365151614 | | | KD3820 | 0 | | 0 | | 0 | | 0 | | 0 | | 0 | | 0 | |  |  |
| 226 | M | | 219 | | 118 | | SP2204 | | 0 | | 0 | | 0 | | 0 | | 0.00 | | 0.00 | | 0 | | | LV3004 | 0 | | 0 | | 1 | | 0 | | 36.41 | | 0 | | 2.490994917 | | | KD3804 | 0 | | 0 | | 0 | | 0 | | 0 | | 0 | | 0 | |  |  |
| 227 | F | | 334 | | 430 | | SP2213 | | 0 | | 0 | | 0 | | 0 | | 0.00 | | 0.00 | | 0 | | | LV3013 | 0 | | 0 | | 1 | | 0 | | 36.655 | | 0 | | 2.108150868 | | | KD3813 | 0 | | 0 | | 0 | | 0 | | 0 | | 0 | | 0 | |  |  |
| 228 | M | | 228 | | 129 | | SP2205 | | 0 | | 0 | | 0 | | 0 | | 0.00 | | 0.00 | | 0 | | | LV3005 | 0 | | 0 | | 1 | | 1 | | 37.736 | | 36.294 | | 1.85268448 | | | KD3805 | 0 | | 0 | | 0 | | 0 | | 0 | | 39.407 | | 0 | |  |  |
| 229 | F | | 306 | | 369 | | SP2223 | | 1 | | 1 | | 1 | | 1 | | 32.25 | | 32.143 | | 43.95588762 | | | LV3023 | 1 | | 1 | | 1 | | 1 | | 28.285 | | 28.64 | | 562.8782117 | | | KD3823 | 0 | | 0 | | 1 | | 1 | | 27.587 | | 27.989 | | 892.9415522 | |  |  |
| 230 | M | | 255 | | 183 | | SP2212 | | 0 | | 0 | | 0 | | 0 | | 0.00 | | 0.00 | | 0 | | | LV3012 | 0 | | 0 | | 1 | | 1 | | 36.6 | | 36.147 | | 2.58414672 | | | KD3812 | 0 | | 0 | | 0 | | 0 | | 0 | | 0 | | 0 | |  |  |
| 231 | F | | 376 | | 751 | | SP2225 | | 0 | | 0 | | 0 | | 0 | | 0.00 | | 0.00 | | 0 | | | LV3025 | 0 | | 0 | | 0 | | 0 | | 0 | | 0 | | 0 | | | KD3825 | 0 | | 0 | | 0 | | 0 | | 0 | | 0 | | 0 | |  |  |
| 233 | F | | 307 | | 388 | | SP2236 | | 0 | | 0 | | 0 | | 0 | | 0.00 | | 0.00 | | 0 | | | LV3036 | 0 | | 0 | | 1 | | 1 | | 36.29 | | 36.401 | | 2.604726926 | | | KD3836 | 0 | | 0 | | 0 | | 0 | | 0 | | 0 | | 0 | |  |  |
| 234 | M | | 225 | | 116 | | SP2210 | | 0 | | 0 | | 0 | | 0 | | 0.00 | | 0.00 | | 0 | | | LV3010 | 0 | | 0 | | 1 | | 1 | | 37.053 | | 36.118 | | 2.323346538 | | | KD3810 | 0 | | 0 | | 0 | | 0 | | 0 | | 0 | | 0 | |  |  |
| 235 | F | | 338 | | 435 | | SP2228 | | 0 | | 0 | | 0 | | 0 | | 0.00 | | 0.00 | | 0 | | | LV3028 | 0 | | 0 | | 1 | | 0 | | 37.389 | | 0 | | 1.27874523 | | | KD3828 | 0 | | 0 | | 0 | | 0 | | 0 | | 0 | | 0 | |  |  |
| 236 | M | | 254 | | 168 | | SP2224 | | 0 | | 0 | | 0 | | 0 | | 0.00 | | 0.00 | | 0 | | | LV3024 | 0 | | 0 | | 0 | | 0 | | 0 | | 0 | | 0 | | | KD3824 | 0 | | 0 | | 0 | | 0 | | 0 | | 0 | | 0 | |  |  |
| 237 | F | | 326 | | 375 | | SP2233 | | 0 | | 0 | | 0 | | 0 | | 0.00 | | 0.00 | | 0 | | | LV3033 | 0 | | 0 | | 1 | | 0 | | 37.061 | | 0 | | 1.598845992 | | | KD3833 | 0 | | 0 | | 0 | | 0 | | 0 | | 0 | | 0 | |  |  |
| 238 | M | | 298 | | 319 | | SP2221 | | 0 | | 0 | | 0 | | 0 | | 0.00 | | 0.00 | | 0 | | | LV3021 | 0 | | 0 | | 1 | | 0 | | 37.229 | | 0 | | 1.425975708 | | | KD3821 | 0 | | 0 | | 0 | | 0 | | 0 | | 0 | | 0 | |  |  |
| 239 | F | | 363 | | 564 | | SP2230 | | 0 | | 0 | | 0 | | 0 | | 0.00 | | 0.00 | | 0 | | | LV3030 | 0 | | 0 | | 0 | | 1 | | 0 | | 37.525 | | 1.165615037 | | | KD3830 | 0 | | 0 | | 0 | | 0 | | 0 | | 0 | | 0 | |  |  |
| 240 | M | | 255 | | 176 | | SP2222 | | 0 | | 0 | | 0 | | 0 | | 0.00 | | 0.00 | | 0 | | | LV3022 | 0 | | 0 | | 0 | | 0 | | 37.761 | | 0 | | 0 | | | KD3822 | 0 | | 0 | | 0 | | 0 | | 0 | | 0 | | 0 | |  |  |
| 241 | F | | 366 | | 610 | | SP2237 | | 0 | | 0 | | 0 | | 0 | | 0.00 | | 0.00 | | 0 | | | LV3037 | 0 | | 0 | | 0 | | 0 | | 0 | | 0 | | 0 | | | KD3837 | 0 | | 0 | | 0 | | 0 | | 0 | | 0 | | 0 | |  |  |
| 242 | M | | 203 | | 88 | | SP2226 | | 0 | | 0 | | 0 | | 0 | | 0.00 | | 0.00 | | 0 | | | LV3026 | 0 | | 0 | | 0 | | 0 | | 0 | | 0 | | 0 | | | KD3826 | 0 | | 0 | | 0 | | 0 | | 0 | | 0 | | 0 | |  |  |
| 243 | F | | 353 | | 594 | | SP2235 | | 0 | | 0 | | 0 | | 0 | | 0.00 | | 0.00 | | 0 | | | LV3035 | 0 | | 0 | | 0 | | 0 | | 38.048 | | 0 | | 0 | | | KD3835 | 0 | | 0 | | 0 | | 0 | | 0 | | 0 | | 0 | |  |  |
| 244 | M | | 259 | | 175 | | SP2227 | | 0 | | 0 | | 0 | | 0 | | 0.00 | | 0.00 | | 0 | | | LV3027 | 0 | | 0 | | 0 | | 0 | | 0 | | 38.155 | | 0 | | | KD3827 | 0 | | 0 | | 0 | | 0 | | 0 | | 0 | | 0 | |  |  |
| 245 | F | | 336 | | 506 | | SP2238 | | 0 | | 0 | | 0 | | 0 | | 0.00 | | 0.00 | | 0 | | | LV3038 | 0 | | 0 | | 0 | | 0 | | 0 | | 38.147 | | 0 | | | KD3838 | 0 | | 0 | | 0 | | 0 | | 0 | | 0 | | 0 | |  |  |
| 246 | M | | 226 | | 115 | | SP2231 | | 0 | | 0 | | 0 | | 0 | | 0.00 | | 0.00 | | 0 | | | LV3031 | 0 | | 0 | | 1 | | 1 | | 36.775 | | 36.493 | | 2.148390179 | | | KD3831 | 0 | | 0 | | 0 | | 0 | | 0 | | 0 | | 0 | |  |  |
| 247 | F | | 370 | | 548 | | SP2239 | | 0 | | 0 | | 0 | | 0 | | 0.00 | | 0.00 | | 0 | | | LV3040 | 0 | | 0 | | 1 | | 0 | | 35.545 | | 38.949 | | 4.489938419 | | | KD3840 | 0 | | 0 | | 0 | | 0 | | 0 | | 0 | | 0 | |  |  |
| 248 | M | | 260 | | 179 | | SP2234 | | 0 | | 0 | | 0 | | 0 | | 0.00 | | 0.00 | | 0 | | | LV3034 | 0 | | 0 | | 1 | | 1 | | 36.848 | | 37.307 | | 1.600332538 | | | KD3834 | 0 | | 0 | | 0 | | 0 | | 0 | | 0 | | 0 | |  |  |
| 249 | F | | 321 | | 392 | | SP2242 | | 0 | | 0 | | 0 | | 0 | | 0.00 | | 0.00 | | 0 | | | LV3043 | 0 | | 0 | | 0 | | 1 | | 0 | | 37.426 | | 1.246922333 | | | KD3843 | 0 | | 0 | | 0 | | 0 | | 0 | | 0 | | 0 | |  |  |
| 250 | M | | 225 | | 118 | | SP2232 | | 0 | | 0 | | 0 | | 0 | | 0.00 | | 0.00 | | 0 | | | LV3032 | 0 | | 0 | | 0 | | 1 | | 38.596 | | 36.854 | | 1.840930371 | | | KD3832 | 0 | | 0 | | 0 | | 0 | | 0 | | 0 | | 0 | |  |  |
| 251 | F | | 331 | | 406 | | SP2248 | | 0 | | 0 | | 0 | | 0 | | 0.00 | | 0.00 | | 0 | | | LV3048 | 0 | | 0 | | 1 | | 0 | | 36.324 | | 0 | | 2.641263291 | | | KD3848 | 0 | | 0 | | 0 | | 0 | | 0 | | 0 | | 0 | |  |  |
| 252 | M | | 226 | | 118 | | SP2229 | | 0 | | 0 | | 0 | | 0 | | 0.00 | | 0.00 | | 0 | | | LV3029 | 0 | | 0 | | 0 | | 0 | | 0 | | 37.834 | | 0 | | | KD3829 | 0 | | 0 | | 0 | | 0 | | 0 | | 0 | | 0 | |  |  |
| 253 | F | | 323 | | 345 | | SP2244 | | 0 | | 0 | | 0 | | 0 | | 0.00 | | 0.00 | | 0 | | | LV3046 | 0 | | 0 | | 0 | | 0 | | 0 | | 0 | | 0 | | | KD3846 | 0 | | 0 | | 0 | | 0 | | 0 | | 0 | | 0 | |  |  |
| 254 | M | | 233 | | 123 | | SP2241 | | 0 | | 0 | | 0 | | 0 | | 0.00 | | 0.00 | | 0 | | | LV3041 | 0 | | 0 | | 1 | | 1 | | 36.679 | | 37.715 | | 1.54904757 | | | KD3841 | 0 | | 0 | | 0 | | 0 | | 0 | | 0 | | 0 | |  |  |
| 255 | F | | 371 | | 585 | | SP2250 | | 0 | | 0 | | 0 | | 0 | | 0.00 | | 0.00 | | 0 | | | LV3051 | 0 | | 0 | | 0 | | 1 | | 0 | | 37.567 | | 1.132743333 | | | KD3850 | 0 | | 0 | | 0 | | 0 | | 0 | | 0 | | 0 | |  |  |
| 256 | M | | 262 | | 185 | | SP2240 | | 0 | | 0 | | 0 | | 0 | | 0.00 | | 0.00 | | 0 | | | LV3039 | 0 | | 0 | | 0 | | 1 | | 0 | | 37.71 | | 1.027618763 | | | KD3839 | 0 | | 0 | | 0 | | 0 | | 0 | | 0 | | 0 | |  |  |
| 257 | F | | 379 | | 621 | | SP2256 | | 0 | | 0 | | 0 | | 0 | | 0.00 | | 39.647 | | 0 | | | LV3056 | 0 | | 0 | | 0 | | 0 | | 0 | | 37.801 | | 0 | | | KD3857 | 0 | | 0 | | 0 | | 0 | | 0 | | 0 | | 0 | |  |  |
| 258 | M | | 244 | | 158 | | SP2243 | | 0 | | 0 | | 0 | | 0 | | 0.00 | | 0.00 | | 0 | | | LV3042 | 0 | | 0 | | 0 | | 0 | | 0 | | 0 | | 0 | | | KD3842 | 0 | | 0 | | 0 | | 0 | | 0 | | 39.052 | | 0 | |  |  |
| 259 | F | | 313 | | 321 | | SP2252 | | 0 | | 0 | | 0 | | 0 | | 0.00 | | 0.00 | | 0 | | | LV3052 | 0 | | 0 | | 0 | | 1 | | 0 | | 37.411 | | 1.259726928 | | | KD3856 | 0 | | 0 | | 0 | | 0 | | 0 | | 0 | | 0 | |  |  |
| 260 | M | | 237 | | 133 | | SP2247 | | 0 | | 0 | | 0 | | 0 | | 0.00 | | 0.00 | | 0 | | | LV3047 | 0 | | 0 | | 1 | | 0 | | 36.098 | | 0 | | 3.08079384 | | | KD3847 | 0 | | 0 | | 0 | | 0 | | 0 | | 0 | | 0 | |  |  |
| 261 | F | | 373 | | 565 | | SP2259 | | 0 | | 0 | | 0 | | 0 | | 0.00 | | 0.00 | | 0 | | | LV3059 | 0 | | 0 | | 1 | | 1 | | 37.739 | | 37.665 | | 1.033561498 | | | KD3860 | 0 | | 0 | | 0 | | 0 | | 0 | | 0 | | 0 | |  |  |
| 262 | M | | 202 | | 81 | | SP2245 | | 0 | | 0 | | 0 | | 0 | | 0.00 | | 0.00 | | 0 | | | LV3044 | 0 | | 0 | | 1 | | 1 | | 37.231 | | 36.88 | | 1.616325644 | | | KD3844 | 0 | | 0 | | 0 | | 0 | | 39.266 | | 0 | | 0 | |  |  |
| 263 | F | | 370 | | 617 | | SP2254 | | 0 | | 0 | | 0 | | 0 | | 0.00 | | 38.958 | | 0 | | | LV3054 | 0 | | 0 | | 1 | | 1 | | 37.7 | | 37.364 | | 1.167673762 | | | KD3854 | 0 | | 0 | | 0 | | 0 | | 0 | | 0 | | 0 | |  |  |
| 264 | M | | 234 | | 145 | | SP2246 | | 0 | | 0 | | 0 | | 0 | | 0.00 | | 0.00 | | 0 | | | LV3045 | 0 | | 0 | | 0 | | 0 | | 0 | | 37.895 | | 0 | | | KD3845 | 0 | | 0 | | 0 | | 0 | | 0 | | 0 | | 0 | |  |  |
| 266 | M | | 248 | | 168 | | SP2253 | | 0 | | 0 | | 0 | | 0 | | 0.00 | | 38.000 | | 0 | | | LV3053 | 0 | | 0 | | 1 | | 0 | | 37.585 | | 38.55 | | 1.118940784 | | | KD3853 | 0 | | 0 | | 0 | | 0 | | 0 | | 0 | | 0 | |  |  |
| 267 | F | | 322 | | 406 | | SP2262 | | 0 | | 0 | | 0 | | 0 | | 0.00 | | 0.00 | | 0 | | | LV3062 | 0 | | 0 | | 0 | | 0 | | 0 | | 0 | | 0 | | | KD3863 | 0 | | 0 | | 0 | | 0 | | 0 | | 0 | | 0 | |  |  |
| 268 | M | | 247 | | 156 | | SP2249 | | 0 | | 0 | | 0 | | 0 | | 0.00 | | 0.00 | | 0 | | | LV3049 | 0 | | 0 | | 1 | | 1 | | 36.435 | | 36.754 | | 2.209812087 | | | KD3849 | 0 | | 0 | | 0 | | 0 | | 0 | | 0 | | 0 | |  |  |
| 269 | F | | 393 | | 724 | | SP2264 | | 0 | | 0 | | 0 | | 0 | | 0.00 | | 0.00 | | 0 | | | LV3064 | 0 | | 0 | | 0 | | 0 | | 0 | | 0 | | 0 | | | KD3865 | 0 | | 0 | | 0 | | 0 | | 0 | | 0 | | 0 | |  |  |
| 270 | M | | 233 | | 128 | | SP2251 | | 0 | | 0 | | 0 | | 0 | | 0.00 | | 0.00 | | 0 | | | LV3050 | 0 | | 0 | | 1 | | 0 | | 36.513 | | 37.919 | | 2.322230778 | | | KD3851 | 0 | | 0 | | 0 | | 0 | | 0 | | 0 | | 0 | |  |  |
| 271 | F | | 367 | | 592 | | SP2260 | | 0 | | 0 | | 0 | | 0 | | 0.00 | | 0.00 | | 0 | | | LV3060 | 0 | | 0 | | 1 | | 0 | | 36.186 | | 38 | | 2.901564186 | | | KD3861 | 0 | | 0 | | 0 | | 0 | | 0 | | 0 | | 0 | |  |  |
| 272 | M | | 262 | | 188 | | SP2255 | | 0 | | 0 | | 0 | | 0 | | 0.00 | | 0.00 | | 0 | | | LV3055 | 0 | | 0 | | 0 | | 1 | | 38.033 | | 36.654 | | 2.109587232 | | | KD3855 | 0 | | 0 | | 0 | | 0 | | 0 | | 0 | | 0 | |  |  |
| 273 | F | | 356 | | 628 | | SP2266 | | 0 | | 0 | | 0 | | 0 | | 0.00 | | 0.00 | | 0 | | | LV3066 | 0 | | 0 | | 0 | | 0 | | 0 | | 37.834 | | 0 | | | KD3867 | 0 | | 0 | | 0 | | 0 | | 0 | | 0 | | 0 | |  |  |
| 274 | M | | 216 | | 122 | | SP2257 | | 0 | | 0 | | 0 | | 0 | | 0.00 | | 0.00 | | 0 | | | LV3057 | 0 | | 0 | | 0 | | 0 | | 0 | | 0 | | 0 | | | KD3858 | 0 | | 0 | | 0 | | 0 | | 0 | | 0 | | 0 | |  |  |
| 275 | F | | 370 | | 716 | | SP2269 | | 0 | | 0 | | 0 | | 0 | | 0.00 | | 0.00 | | 0 | | | LV3069 | 0 | | 0 | | 0 | | 0 | | 0 | | 0 | | 0 | | | KD3871 | 0 | | 0 | | 0 | | 0 | | 0 | | 0 | | 0 | |  |  |
| 276 | M | | 224 | | 122 | | SP2258 | | 0 | | 0 | | 0 | | 0 | | 0.00 | | 0.00 | | 0 | | | LV3058 | 0 | | 0 | | 0 | | 0 | | 0 | | 0 | | 0 | | | KD3859 | 0 | | 0 | | 0 | | 0 | | 0 | | 0 | | 0 | |  |  |
| 277 | F | | 429 | | 1089 | | SP2272 | | 0 | | 0 | | 0 | | 0 | | 0.00 | | 0.00 | | 0 | | | LV3072 | 0 | | 0 | | 0 | | 0 | | 0 | | 0 | | 0 | | | KD3873 | 0 | | 0 | | 0 | | 0 | | 0 | | 0 | | 0 | |  |  |
| 278 | M | | 243 | | 148 | | SP2265 | | 0 | | 0 | | 0 | | 0 | | 0.00 | | 0.00 | | 0 | | | LV3065 | 0 | | 0 | | 0 | | 0 | | 0 | | 0 | | 0 | | | KD3866 | 0 | | 0 | | 0 | | 0 | | 0 | | 0 | | 0 | |  |  |
| 279 | F | | 353 | | 496 | | SP2274 | | 0 | | 0 | | 0 | | 0 | | 0.00 | | 0.00 | | 0 | | | LV3075 | 0 | | 0 | | 1 | | 0 | | 36.715 | | 0 | | 2.023735033 | | | KD3876 | 0 | | 0 | | 0 | | 0 | | 0 | | 0 | | 0 | |  |  |
| 280 | M | | 239 | | 131 | | SP2261 | | 0 | | 0 | | 0 | | 0 | | 0.00 | | 0.00 | | 0 | | | LV3061 | 0 | | 0 | | 1 | | 0 | | 36.906 | | 0 | | 1.776870307 | | | KD3862 | 0 | | 0 | | 0 | | 0 | | 0 | | 0 | | 0 | |  |  |
| 281 | F | | 340 | | 454 | | SP2279 | | 0 | | 0 | | 0 | | 0 | | 0.00 | | 0.00 | | 0 | | | LV3079 | 0 | | 0 | | 1 | | 0 | | 36.931 | | 0 | | 1.746870514 | | | KD3880 | 0 | | 0 | | 0 | | 0 | | 0 | | 0 | | 0 | |  |  |
| 282 | M | | 231 | | 139 | | SP2263 | | 0 | | 0 | | 0 | | 0 | | 0.00 | | 0.00 | | 0 | | | LV3063 | 0 | | 0 | | 1 | | 0 | | 37.241 | | 0 | | 1.414368318 | | | KD3864 | 0 | | 0 | | 0 | | 0 | | 0 | | 0 | | 0 | |  |  |
| 283 | F | | 412 | | 820 | | SP2277 | | 0 | | 0 | | 0 | | 0 | | 0.00 | | 0.00 | | 0 | | | LV3077 | 0 | | 0 | | 1 | | 0 | | 36.343 | | 0 | | 2.607302875 | | | KD3878 | 0 | | 0 | | 0 | | 0 | | 0 | | 0 | | 0 | |  |  |
| 284 | M | | 247 | | 161 | | SP2267 | | 0 | | 0 | | 0 | | 0 | | 0.00 | | 0.00 | | 0 | | | LV3067 | 0 | | 0 | | 0 | | 0 | | 0 | | 0 | | 0 | | | KD3868 | 0 | | 0 | | 0 | | 0 | | 0 | | 0 | | 0 | |  |  |
| 285 | F | | 335 | | 438 | | SP2282 | | 0 | | 0 | | 0 | | 0 | | 0.00 | | 0.00 | | 0 | | | LV3082 | 0 | | 0 | | 0 | | 0 | | 0 | | 0 | | 0 | | | KD3883 | 0 | | 1 | | 0 | | 0 | | 0 | | 0 | | 0 | |  |  |
| 286 | M | | 193 | | 77 | | SP2270 | | 0 | | 0 | | 0 | | 0 | | 0.00 | | 0.00 | | 0 | | | LV3070 | 0 | | 0 | | 0 | | 0 | | 0 | | 0 | | 0 | | | KD3870 | 0 | | 0 | | 0 | | 0 | | 0 | | 0 | | 0 | |  |  |
| 287 | F | | 356 | | 510 | | SP2284 | | 0 | | 0 | | 0 | | 0 | | 0.00 | | 0.00 | | 0 | | | LV3084 | 0 | | 0 | | 0 | | 0 | | 0 | | 37.872 | | 0 | | | KD3886 | 0 | | 0 | | 0 | | 0 | | 0 | | 0 | | 0 | |  |  |
| 288 | M | | 248 | | 157 | | SP2268 | | 0 | | 0 | | 0 | | 0 | | 0.00 | | 0.00 | | 0 | | | LV3068 | 0 | | 0 | | 0 | | 0 | | 0 | | 0 | | 0 | | | KD3869 | 0 | | 0 | | 0 | | 0 | | 0 | | 0 | | 0 | |  |  |
| 289 | F | | 386 | | 723 | | SP2287 | | 0 | | 0 | | 0 | | 0 | | 0.00 | | 0.00 | | 0 | | | LV3086 | 0 | | 0 | | 0 | | 1 | | 38 | | 36.887 | | 1.800014244 | | | KD3888 | 0 | | 0 | | 0 | | 0 | | 0 | | 0 | | 0 | |  |  |
| 290 | M | | 245 | | 137 | | SP2271 | | 0 | | 0 | | 0 | | 0 | | 0.00 | | 0.00 | | 0 | | | LV3071 | 0 | | 0 | | 0 | | 1 | | 0 | | 36.486 | | 2.365331381 | | | KD3872 | 0 | | 0 | | 0 | | 0 | | 0 | | 0 | | 0 | |  |  |
| 291 | F | | 281 | | 644 | | SP2286 | | 0 | | 0 | | 0 | | 0 | | 0.00 | | 0.00 | | 0 | | | LV3087 | 0 | | 0 | | 1 | | 1 | | 36.453 | | 37.563 | | 1.777465674 | | | KD3887 | 0 | | 0 | | 0 | | 0 | | 0 | | 0 | | 0 | |  |  |
| 292 | M | | 221 | | 95 | | SP2273 | | 0 | | 0 | | 0 | | 0 | | 0.00 | | 0.00 | | 0 | | | LV3073 | 0 | | 0 | | 0 | | 1 | | 0 | | 37.134 | | 1.52129412 | | | KD3874 | 0 | | 0 | | 0 | | 0 | | 0 | | 0 | | 0 | |  |  |
| 293 | F | | 370 | | 585 | | SP2288 | | 0 | | 0 | | 0 | | 0 | | 0.00 | | 0.00 | | 0 | | | LV3088 | 0 | | 0 | | 0 | | 0 | | 0 | | 0 | | 0 | | | KD3889 | 0 | | 1 | | 0 | | 0 | | 0 | | 0 | | 0 | |  |  |
| 294 | M | | 255 | | 178 | | SP2275 | | 0 | | 0 | | 0 | | 0 | | 0.00 | | 0.00 | | 0 | | | LV3074 | 0 | | 0 | | 0 | | 0 | | 0 | | 37.918 | | 0 | | | KD3875 | 0 | | 0 | | 0 | | 0 | | 0 | | 0 | | 0 | |  |  |
| 295 | F | | 389 | | 759 | | SP2289 | | 0 | | 0 | | 0 | | 0 | | 0.00 | | 0.00 | | 0 | | | LV3089 | 0 | | 0 | | 1 | | 1 | | 37.341 | | 37.15 | | 1.413023893 | | | KD3890 | 0 | | 0 | | 0 | | 0 | | 0 | | 0 | | 0 | |  |  |
| 296 | M | | 204 | | 88 | | SP2276 | | 0 | | 0 | | 0 | | 0 | | 0.00 | | 0.00 | | 0 | | | LV3076 | 0 | | 0 | | 0 | | 0 | | 38.257 | | 0 | | 0 | | | KD3877 | 0 | | 0 | | 0 | | 0 | | 0 | | 0 | | 0 | |  |  |
| 297 | F | | 427 | | 975 | | SP2290 | | 0 | | 0 | | 0 | | 0 | | 0.00 | | 0.00 | | 0 | | | LV3090 | 0 | | 0 | | 1 | | 0 | | 37.705 | | 0 | | 1.031124315 | | | KD3891 | 0 | | 0 | | 0 | | 0 | | 0 | | 0 | | 0 | |  |  |
| 298 | M | | 292 | | 202 | | SP2278 | | 0 | | 0 | | 0 | | 0 | | 0.00 | | 0.00 | | 0 | | | LV3078 | 0 | | 0 | | 1 | | 0 | | 36.689 | | 38.185 | | 2.059892072 | | | KD3879 | 0 | | 0 | | 0 | | 0 | | 0 | | 0 | | 0 | |  |  |
| 299 | F | | 345 | | 509 | | SP2291 | | 0 | | 0 | | 0 | | 0 | | 0.00 | | 0.00 | | 0 | | | LV3091 | 0 | | 0 | | 0 | | 0 | | 38.999 | | 0 | | 0 | | | KD3892 | 0 | | 0 | | 0 | | 0 | | 0 | | 0 | | 0 | |  |  |
| 300 | M | | 204 | | 130 | | SP2280 | | 0 | | 0 | | 0 | | 0 | | 0.00 | | 0.00 | | 0 | | | LV3080 | 0 | | 0 | | 1 | | 0 | | 35.191 | | 37.885 | | 5.714178553 | | | KD3881 | 0 | | 0 | | 0 | | 0 | | 0 | | 0 | | 0 | |  |  |
| 301 | F | | 365 | | 561 | | SP2292 | | 0 | | 0 | | 0 | | 0 | | 0.00 | | 0.00 | | 0 | | | LV3092 | 0 | | 0 | | 1 | | 0 | | 34.862 | | 0 | | 7.149443089 | | | KD3893 | 0 | | 0 | | 0 | | 0 | | 0 | | 0 | | 0 | |  |  |
| 302 | M | | 251 | | 172 | | SP2285 | | 0 | | 0 | | 0 | | 0 | | 0.00 | | 0.00 | | 0 | | | LV3085 | 0 | | 0 | | 1 | | 1 | | 37.695 | | 35.81 | | 2.393317125 | | | KD3885 | 0 | | 0 | | 0 | | 0 | | 0 | | 0 | | 0 | |  |  |
| 303 | F | | 361 | | 553 | | SP2293 | | 0 | | 0 | | 0 | | 0 | | 0.00 | | 0.00 | | 0 | | | LV3093 | 0 | | 0 | | 0 | | 0 | | 0 | | 0 | | 0 | | | KD3894 | 0 | | 0 | | 0 | | 0 | | 0 | | 0 | | 0 | |  |  |
| 304 | M | | 260 | | 179 | | SP2281 | | 1 | | 1 | | 1 | | 1 | | 31.79 | | 31.348 | | 68.07595471 | | | LV3081 | 1 | | 1 | | 1 | | 1 | | 31.301 | | 32.013 | | 65.30657457 | | | KD3882 | 1 | | 1 | | 1 | | 1 | | 26.991 | | 27.15 | | 1444.239339 | |  |  |
| 305 | F | | 357 | | 508 | | SP2295 | | 0 | | 0 | | 0 | | 0 | | 0.00 | | 38.000 | | 0 | | | LV3095 | 0 | | 0 | | 1 | | 1 | | 37.618 | | 37.051 | | 1.351922222 | | | KD3896 | 0 | | 0 | | 0 | | 0 | | 0 | | 0 | | 0 | |  |  |
| 306 | M | | 244 | | 146 | | SP2283 | | 0 | | 0 | | 0 | | 0 | | 0.00 | | 0.00 | | 0 | | | LV3083 | 0 | | 0 | | 0 | | 1 | | 0 | | 34.278 | | 10.64191486 | | | KD3884 | 0 | | 0 | | 0 | | 0 | | 0 | | 0 | | 0 | |  |  |
| 307 | F | | 372 | | 637 | | SP2297 | | 0 | | 0 | | 0 | | 0 | | 0.00 | | 0.00 | | 0 | | | LV3097 | 0 | | 0 | | 0 | | 0 | | 0 | | 0 | | 0 | | | KD3898 | 0 | | 0 | | 0 | | 0 | | 0 | | 0 | | 0 | |  |  |
| 308 | M | | 204 | | 268 | | SP2294 | | 0 | | 0 | | 0 | | 0 | | 0.00 | | 0.00 | | 0 | | | LV3094 | 0 | | 0 | | 0 | | 0 | | 0 | | 0 | | 0 | | | KD3895 | 0 | | 0 | | 0 | | 0 | | 0 | | 0 | | 0 | |  |  |
| 309 | F | | 339 | | 436 | | SP2304 | | 0 | | 0 | | 0 | | 0 | | 0.00 | | 0.00 | | 0 | | | LV3104 | 0 | | 0 | | 0 | | 0 | | 0 | | 39.24 | | 0 | | | KD3905 | 0 | | 0 | | 0 | | 0 | | 0 | | 0 | | 0 | |  |  |
| 310 | M | | 229 | | 123 | | SP2296 | | 0 | | 0 | | 0 | | 0 | | 39.54 | | 0.00 | | 0 | | | LV3096 | 0 | | 0 | | 1 | | 0 | | 35.579 | | 0 | | 4.387156866 | | | KD3897 | 0 | | 0 | | 0 | | 0 | | 39.664 | | 0 | | 0 | |  |  |
| 311 | F | | 343 | | 527 | | SP2299 | | 0 | | 0 | | 0 | | 0 | | 0.00 | | 0.00 | | 0 | | | Lv3099 | 0 | | 0 | | 1 | | 1 | | 35.573 | | 36.759 | | 3.184554211 | | | KD3902 | 0 | | 0 | | 0 | | 0 | | 0 | | 0 | | 0 | |  |  |
| 312 | M | | 255 | | 174 | | SP2298 | | 0 | | 0 | | 0 | | 0 | | 0.00 | | 0.00 | | 0 | | | LV3098 | 0 | | 0 | | 1 | | 0 | | 35.813 | | 38.408 | | 3.740811428 | | | KD3899 | 0 | | 0 | | 0 | | 0 | | 0 | | 0 | | 0 | |  |  |
| 313 | F | | 389 | | 727 | | SP2302 | | 0 | | 0 | | 0 | | 0 | | 0.00 | | 0.00 | | 0 | | | LV3102 | 0 | | 0 | | 0 | | 0 | | 0 | | 37.753 | | 0 | | | KD3903 | 0 | | 0 | | 0 | | 0 | | 0 | | 0 | | 0 | |  |  |
| 314 | M | | 233 | | 135 | | SP2301 | | 0 | | 0 | | 0 | | 0 | | 0.00 | | 0.00 | | 0 | | | LV3101 | 0 | | 0 | | 0 | | 0 | | 38.917 | | 0 | | 0 | | | KD3901 | 0 | | 0 | | 0 | | 0 | | 0 | | 38 | | 0 | |  |  |
| 315 | F | | 323 | | 411 | | SP2307 | | 0 | | 0 | | 0 | | 0 | | 0.00 | | 0.00 | | 0 | | | LV3107 | 0 | | 0 | | 0 | | 0 | | 0 | | 0 | | 0 | | | KD3908 | 0 | | 0 | | 0 | | 0 | | 0 | | 0 | | 0 | |  |  |
| 316 | M | | 231 | | 121 | | SP2303 | | 0 | | 0 | | 0 | | 0 | | 0.00 | | 0.00 | | 0 | | | LV3103 | 0 | | 0 | | 1 | | 0 | | 36.479 | | 0 | | 2.376635599 | | | KD3904 | 0 | | 0 | | 0 | | 0 | | 0 | | 0 | | 0 | |  |  |
| 317 | F | | 321 | | 417 | | SP2309 | | 0 | | 0 | | 0 | | 0 | | 0.00 | | 0.00 | | 0 | | | LV3109 | 0 | | 0 | | 0 | | 0 | | 0 | | 0 | | 0 | | | KD3910 | 0 | | 0 | | 0 | | 0 | | 0 | | 0 | | 0 | |  |  |
| 318 | M | | 229 | | 113 | | SP2300 | | 0 | | 0 | | 0 | | 0 | | 0.00 | | 0.00 | | 0 | | | LV3100 | 0 | | 0 | | 0 | | 0 | | 0 | | 0 | | 0 | | | KD3900 | 0 | | 0 | | 0 | | 0 | | 0 | | 0 | | 0 | |  |  |
| 319 | F | | 379 | | 690 | | SP2312 | | 0 | | 0 | | 0 | | 0 | | 0.00 | | 0.00 | | 0 | | | LV3112 | 0 | | 0 | | 0 | | 0 | | 0 | | 0 | | 0 | | | KD3913 | 0 | | 0 | | 0 | | 0 | | 0 | | 0 | | 0 | |  |  |
| 320 | M | | 234 | | 144 | | SP2305 | | 0 | | 0 | | 0 | | 0 | | 0.00 | | 0.00 | | 0 | | | LV3105 | 0 | | 0 | | 0 | | 0 | | 37.829 | | 0 | | 0 | | | KD3906 | 0 | | 0 | | 0 | | 0 | | 0 | | 0 | | 0 | |  |  |
| 321 | F | | 342 | | 464 | | SP2314 | | 0 | | 0 | | 0 | | 0 | | 0.00 | | 0.00 | | 0 | | | LV3114 | 0 | | 0 | | 1 | | 1 | | 37.022 | | 36.618 | | 1.901919386 | | | KD3915 | 0 | | 0 | | 0 | | 0 | | 0 | | 0 | | 0 | |  |  |
| 322 | M | | 253 | | 154 | | SP2306 | | 0 | | 0 | | 0 | | 0 | | 0.00 | | 0.00 | | 0 | | | LV3106 | 0 | | 0 | | 0 | | 0 | | 0 | | 0 | | 0 | | | KD3907 | 0 | | 0 | | 0 | | 0 | | 0 | | 0 | | 0 | |  |  |
| 323 | F | | 306 | | 365 | | SP2319 | | 0 | | 0 | | 0 | | 0 | | 0.00 | | 0.00 | | 0 | | | LV3119 | 0 | | 0 | | 1 | | 0 | | 36.719 | | 39.046 | | 2.018229024 | | | KD3920 | 0 | | 0 | | 0 | | 0 | | 0 | | 0 | | 0 | |  |  |
| 324 | M | | 222 | | 124 | | SP2330 | | 0 | | 0 | | 0 | | 0 | | 0.00 | | 0.00 | | 0 | | | LV3130 | 0 | | 0 | | 0 | | 0 | | 0 | | 0 | | 0 | | | KD3931 | 0 | | 0 | | 0 | | 0 | | 0 | | 0 | | 0 | |  |  |
| 325 | F | | 343 | | 506 | | SP2317 | | 0 | | 0 | | 0 | | 0 | | 0.00 | | 0.00 | | 0 | | | LV3117 | 0 | | 0 | | 0 | | 0 | | 0 | | 0 | | 0 | | | KD3918 | 0 | | 0 | | 0 | | 0 | | 0 | | 0 | | 0 | |  |  |
| 326 | M | | 272 | | 208 | | SP2311 | | 0 | | 0 | | 0 | | 0 | | 0.00 | | 0.00 | | 0 | | | LV3111 | 0 | | 0 | | 0 | | 0 | | 0 | | 0 | | 0 | | | KD3912 | 0 | | 0 | | 0 | | 0 | | 0 | | 0 | | 0 | |  |  |
| 327 | F | | 346 | | 465 | | SP2322 | | 0 | | 0 | | 0 | | 0 | | 0.00 | | 0.00 | | 0 | | | LV3122 | 0 | | 0 | | 0 | | 1 | | 0 | | 36.573 | | 2.229242628 | | | KD3923 | 0 | | 0 | | 0 | | 0 | | 0 | | 0 | | 0 | |  |  |
| 328 | M | | 201 | | 85 | | SP2308 | | 0 | | 0 | | 0 | | 0 | | 0.00 | | 0.00 | | 0 | | | LV3108 | 0 | | 0 | | 0 | | 0 | | 0 | | 0 | | 0 | | | KD3909 | 0 | | 0 | | 0 | | 0 | | 0 | | 0 | | 0 | |  |  |
| 329 | F | | 343 | | 455 | | SP2324 | | 0 | | 0 | | 0 | | 0 | | 0.00 | | 0.00 | | 0 | | | LV3124 | 0 | | 0 | | 0 | | 0 | | 0 | | 39.149 | | 0 | | | KD3925 | 0 | | 0 | | 0 | | 0 | | 0 | | 0 | | 0 | |  |  |
| 330 | M | | 219 | | 112 | | SP2310 | | 0 | | 0 | | 0 | | 0 | | 0.00 | | 0.00 | | 0 | | | LV3110 | 0 | | 0 | | 1 | | 0 | | 37.137 | | 0 | | 1.518188806 | | | KD3911 | 0 | | 0 | | 0 | | 0 | | 0 | | 0 | | 0 | |  |  |
| 331 | F | | 335 | | 484 | | SP2331 | | 0 | | 0 | | 0 | | 0 | | 0.00 | | 0.00 | | 0 | | | LV3131 | 0 | | 0 | | 0 | | 1 | | 0 | | 37.396 | | 1.272663012 | | | KD3932 | 0 | | 0 | | 0 | | 0 | | 0 | | 0 | | 0 | |  |  |
| 332 | M | | 235 | | 128 | | SP2321 | | 0 | | 0 | | 0 | | 0 | | 0.00 | | 0.00 | | 0 | | | LV3121 | 0 | | 0 | | 0 | | 0 | | 38 | | 0 | | 0 | | | KD3922 | 0 | | 0 | | 0 | | 0 | | 0 | | 0 | | 0 | |  |  |
| 333 | F | | 366 | | 631 | | SP2328 | | 0 | | 0 | | 0 | | 0 | | 0.00 | | 0.00 | | 0 | | | LV3128 | 0 | | 0 | | 0 | | 0 | | 0 | | 38 | | 0 | | | KD3929 | 0 | | 0 | | 0 | | 0 | | 0 | | 0 | | 0 | |  |  |
| 334 | M | | 225 | | 108 | | SP2315 | | 0 | | 0 | | 0 | | 0 | | 0.00 | | 0.00 | | 0 | | | LV3115 | 0 | | 0 | | 1 | | 0 | | 36.584 | | 38.194 | | 2.21260318 | | | KD3916 | 0 | | 0 | | 0 | | 0 | | 0 | | 0 | | 0 | |  |  |
| 335 | F | | 306 | | 332 | | SP2325 | | 1 | | 1 | | 1 | | 1 | | 34.31 | | 32.690 | | 20.88562456 | | | LV3125 | 1 | | 1 | | 1 | | 1 | | 30.148 | | 30.287 | | 169.2796012 | | | KD3926 | 1 | | 1 | | 1 | | 1 | | 29.562 | | 30.699 | | 193.0311875 | |  |  |
| 336 | M | | 251 | | 152 | | SP2313 | | 0 | | 0 | | 0 | | 0 | | 0.00 | | 0.00 | | 0 | | | LV3113 | 0 | | 0 | | 0 | | 0 | | 0 | | 0 | | 0 | | | KD3914 | 0 | | 0 | | 0 | | 0 | | 0 | | 0 | | 0 | |  |  |
| 338 | M | | 227 | | 125 | | SP2316 | | 0 | | 0 | | 0 | | 0 | | 0.00 | | 0.00 | | 0 | | | LV3116 | 0 | | 0 | | 0 | | 0 | | 0 | | 0 | | 0 | | | KD3917 | 0 | | 0 | | 0 | | 0 | | 0 | | 0 | | 0 | |  |  |
| 339 | F | | 350 | | 548 | | SP2333 | | 0 | | 0 | | 0 | | 0 | | 0.00 | | 0.00 | | 0 | | | LV3133 | 0 | | 0 | | 0 | | 0 | | 38.376 | | 0 | | 0 | | | KD3934 | 0 | | 0 | | 0 | | 0 | | 0 | | 0 | | 0 | |  |  |
| 340 | M | | 248 | | 152 | | SP2318 | | 0 | | 0 | | 0 | | 0 | | 0.00 | | 0.00 | | 0 | | | LV3118 | 0 | | 0 | | 0 | | 0 | | 0 | | 0 | | 0 | | | KD3919 | 0 | | 0 | | 0 | | 0 | | 0 | | 0 | | 0 | |  |  |
| 341 | F | | 371 | | 586 | | SP2335 | | 0 | | 0 | | 0 | | 0 | | 0.00 | | 0.00 | | 0 | | | LV3135 | 0 | | 0 | | 0 | | 0 | | 0 | | 0 | | 0 | | | KD3936 | 1 | | 0 | | 0 | | 0 | | 0 | | 0 | | 0 | |  |  |
| 342 | M | | 231 | | 156 | | SP2320 | | 0 | | 0 | | 0 | | 0 | | 0.00 | | 0.00 | | 0 | | | LV3120 | 0 | | 0 | | 0 | | 1 | | 0 | | 36.992 | | 1.67577951 | | | KD3921 | 0 | | 0 | | 0 | | 0 | | 0 | | 0 | | 0 | |  |  |
| 343 | F | | 360 | | 541 | | SP2340 | | 0 | | 0 | | 0 | | 0 | | 0.00 | | 0.00 | | 0 | | | LV3140 | 0 | | 0 | | 0 | | 0 | | 0 | | 0 | | 0 | | | KD3941 | 0 | | 0 | | 0 | | 0 | | 0 | | 0 | | 0 | |  |  |
| 344 | M | | 203 | | 96 | | SP2323 | | 0 | | 0 | | 0 | | 0 | | 0.00 | | 0.00 | | 0 | | | LV3123 | 0 | | 0 | | 1 | | 0 | | 37.608 | | 0 | | 1.101548667 | | | KD3924 | 0 | | 0 | | 0 | | 0 | | 0 | | 0 | | 0 | |  |  |
| 345 | F | | 405 | | 896 | | SP2336 | | 0 | | 0 | | 0 | | 0 | | 0.00 | | 0.00 | | 0 | | | LV3136 | 0 | | 0 | | 0 | | 0 | | 0 | | 0 | | 0 | | | KD3937 | 0 | | 0 | | 0 | | 0 | | 0 | | 0 | | 0 | |  |  |
| 346 | M | | 282 | | 209 | | SP2329 | | 0 | | 0 | | 0 | | 0 | | 0.00 | | 0.00 | | 0 | | | LV3129 | 0 | | 0 | | 0 | | 1 | | 0 | | 36.796 | | 1.915110331 | | | KD3930 | 0 | | 0 | | 0 | | 0 | | 39.034 | | 0 | | 0 | |  |  |
| 347 | F | | 335 | | 531 | | SP2337 | | 0 | | 0 | | 0 | | 0 | | 0.00 | | 0.00 | | 0 | | | LV3137 | 0 | | 0 | | 0 | | 0 | | 0 | | 0 | | 0 | | | KD3938 | 0 | | 0 | | 0 | | 0 | | 0 | | 0 | | 0 | |  |  |
| 348 | M | | 242 | | 149 | | SP2334 | | 0 | | 0 | | 0 | | 0 | | 0.00 | | 0.00 | | 0 | | | LV3134 | 0 | | 0 | | 1 | | 0 | | 37.51 | | 0 | | 1.17758469 | | | KD3935 | 0 | | 0 | | 0 | | 0 | | 0 | | 0 | | 0 | |  |  |
| 349 | F | | 376 | | 692 | | SP2338 | | 0 | | 0 | | 0 | | 0 | | 0.00 | | 0.00 | | 0 | | | LV3138 | 0 | | 0 | | 0 | | 0 | | 0 | | 0 | | 0 | | | KD3939 | 0 | | 0 | | 0 | | 0 | | 0 | | 0 | | 0 | |  |  |
| 350 | M | | 256 | | 163 | | SP2332 | | 0 | | 0 | | 0 | | 0 | | 0.00 | | 0.00 | | 0 | | | LV3132 | 0 | | 0 | | 0 | | 0 | | 0 | | 39.002 | | 0 | | | KD3933 | 0 | | 0 | | 0 | | 0 | | 0 | | 0 | | 0 | |  |  |
| 351 | F | | 371 | | 575 | | SP2339 | | 0 | | 0 | | 0 | | 0 | | 0.00 | | 0.00 | | 0 | | | LV3139 | 0 | | 0 | | 0 | | 0 | | 0 | | 0 | | 0 | | | KD3940 | 0 | | 0 | | 0 | | 0 | | 0 | | 0 | | 0 | |  |  |
| 352 | M | | 221 | | 113 | | SP2327 | | 0 | | 0 | | 0 | | 0 | | 0.00 | | 0.00 | | 0 | | | LV3127 | 0 | | 0 | | 1 | | 1 | | 37.658 | | 37.312 | | 1.206132826 | | | KD3928 | 0 | | 0 | | 0 | | 0 | | 0 | | 0 | | 0 | |  |  |
| 353 | F | | 352 | | 488 | | SP2342 | | 0 | | 0 | | 0 | | 0 | | 0.00 | | 0.00 | | 0 | | | LV3142 | 0 | | 0 | | 0 | | 0 | | 0 | | 0 | | 0 | | | KD3943 | 0 | | 0 | | 0 | | 0 | | 0 | | 0 | | 0 | |  |  |
| 354 | M | | 251 | | 148 | | SP2326 | | 0 | | 0 | | 0 | | 0 | | 0.00 | | 0.00 | | 0 | | | LV3126 | 0 | | 0 | | 0 | | 1 | | 0 | | 37.409 | | 1.261444112 | | | KD3927 | 0 | | 0 | | 0 | | 0 | | 0 | | 38.196 | | 0 | |  |  |
| 355 | F | | 351 | | 628 | | SP2345 | | 0 | | 0 | | 0 | | 0 | | 39.02 | | 0.00 | | 0 | | | LV3145 | 0 | | 0 | | 1 | | 1 | | 36.715 | | 36.163 | | 2.485555699 | | | KD3947 | 0 | | 0 | | 0 | | 0 | | 0 | | 0 | | 0 | |  |  |
| 356 | M | | 274 | | 209 | | SP2341 | | 0 | | 0 | | 0 | | 0 | | 0.00 | | 0.00 | | 0 | | | LV3141 | 0 | | 0 | | 0 | | 0 | | 0 | | 0 | | 0 | | | KD3942 | 1 | | 0 | | 0 | | 0 | | 0 | | 0 | | 0 | |  |  |
| 357 | F | | 362 | | 618 | | SP2343 | | 0 | | 0 | | 0 | | 0 | | 0.00 | | 0.00 | | 0 | | | LV3143 | 0 | | 0 | | 0 | | 0 | | 0 | | 0 | | 0 | | | KD3944 | 0 | | 0 | | 0 | | 0 | | 0 | | 0 | | 0 | |  |  |
| 358 | M | | 214 | | 112 | | SP2344 | | 0 | | 0 | | 0 | | 0 | | 0.00 | | 0.00 | | 0 | | | LV3144 | 0 | | 0 | | 1 | | 1 | | 35.3 | | 36.455 | | 3.860562045 | | | KD3945 | 0 | | 0 | | 0 | | 0 | | 0 | | 0 | | 0 | |  |  |
| 359 | F | | 345 | | 418 | | SP2348 | | 0 | | 0 | | 0 | | 0 | | 0.00 | | 0.00 | | 0 | | | LV3148 | 0 | | 0 | | 0 | | 1 | | 0 | | 37.34 | | 1.322142475 | | | KD3951 | 0 | | 0 | | 0 | | 0 | | 0 | | 0 | | 0 | |  |  |
| 360 | M | | 248 | | 165 | | SP2346 | | 0 | | 0 | | 0 | | 0 | | 0.00 | | 0.00 | | 0 | | | LV3146 | 0 | | 0 | | 1 | | 1 | | 36.806 | | 36.088 | | 2.501979828 | | | KD3946 | 0 | | 0 | | 0 | | 0 | | 0 | | 0 | | 0 | |  |  |
| 361 | F | | 360 | | 508 | | SP2350 | | 0 | | 0 | | 0 | | 0 | | 0.00 | | 0.00 | | 0 | | | LV3150 | 0 | | 0 | | 0 | | 0 | | 37.866 | | 0 | | 0 | | | KD3953 | 0 | | 1 | | 0 | | 0 | | 0 | | 0 | | 0 | |  |  |
| 362 | M | | 245 | | 153 | | SP2351 | | 0 | | 0 | | 0 | | 0 | | 0.00 | | 0.00 | | 0 | | | LV3151 | 0 | | 0 | | 1 | | 0 | | 37.037 | | 0 | | 1.625196385 | | | KD3952 | 0 | | 0 | | 0 | | 0 | | 0 | | 0 | | 0 | |  |  |
| 363 | F | | 373 | | 670 | | SP2353 | | 0 | | 0 | | 0 | | 0 | | 0.00 | | 0.00 | | 0 | | | LV3153 | 0 | | 0 | | 1 | | 0 | | 36.726 | | 0 | | 2.008629529 | | | KD3955 | 0 | | 1 | | 0 | | 0 | | 0 | | 0 | | 0 | |  |  |
| 364 | M | | 237 | | 157 | | SP2347 | | 0 | | 0 | | 0 | | 0 | | 0.00 | | 0.00 | | 0 | | | LV3147 | 0 | | 0 | | 1 | | 1 | | 36.073 | | 36.322 | | 2.889282701 | | | KD3948 | 0 | | 0 | | 0 | | 0 | | 0 | | 0 | | 0 | |  |  |
| 365 | F | | 323 | | 368 | | SP2356 | | 0 | | 0 | | 0 | | 0 | | 0.00 | | 0.00 | | 0 | | | LV3156 | 0 | | 0 | | 1 | | 1 | | 35.523 | | 35.816 | | 4.145449656 | | | KD3958 | 0 | | 0 | | 0 | | 0 | | 0 | | 39.648 | | 0 | |  |  |
| 366 | M | | 211 | | 93 | | SP2349 | | 0 | | 0 | | 0 | | 0 | | 0.00 | | 0.00 | | 0 | | | LV3149 | 0 | | 0 | | 0 | | 1 | | 0 | | 37.177 | | 1.477385253 | | | KD3950 | 0 | | 0 | | 0 | | 0 | | 0 | | 0 | | 0 | |  |  |
| 367 | F | | 354 | | 508 | | SP2358 | | 0 | | 0 | | 0 | | 0 | | 0.00 | | 0.00 | | 0 | | | LV3158 | 0 | | 0 | | 1 | | 1 | | 35.818 | | 37.49 | | 2.460914648 | | | KD3960 | 0 | | 0 | | 0 | | 0 | | 0 | | 39.727 | | 0 | |  |  |
| 368 | M | | 234 | | 178 | | SP2401 | | 0 | | 0 | | 0 | | 0 | | 0.00 | | 0.00 | | 0 | | | LV3176 | 0 | | 0 | | 0 | | 1 | | 0 | | 36.945 | | 1.730292421 | | | KD4003 | 0 | | 0 | | 0 | | 0 | | 0 | | 0 | | 0 | |  |  |
| 369 | F | | 359 | | 723 | | SP2363 | | 0 | | 0 | | 0 | | 0 | | 0.00 | | 0.00 | | 0 | | | LV3163 | 0 | | 0 | | 1 | | 1 | | 37.009 | | 36.1 | | 2.366543946 | | | KD3965 | 0 | | 0 | | 0 | | 0 | | 0 | | 0 | | 0 | |  |  |
| 370 | M | | 231 | | 128 | | SP2352 | | 0 | | 0 | | 0 | | 0 | | 0.00 | | 0.00 | | 0 | | | LV3152 | 0 | | 0 | | 1 | | 0 | | 37.424 | | 0 | | 1.248622063 | | | KD3954 | 0 | | 0 | | 0 | | 0 | | 0 | | 0 | | 0 | |  |  |
| 371 | F | | 335 | | 428 | | SP2368 | | 0 | | 0 | | 0 | | 0 | | 0.00 | | 0.00 | | 0 | | | LV3168 | 0 | | 0 | | 0 | | 0 | | 0 | | 0 | | 0 | | | KD3970 | 0 | | 0 | | 0 | | 0 | | 0 | | 0 | | 0 | |  |  |
| 372 | M | | 253 | | 158 | | SP2354 | | 0 | | 0 | | 0 | | 0 | | 0.00 | | 0.00 | | 0 | | | LV3154 | 0 | | 0 | | 1 | | 1 | | 37.622 | | 37.611 | | 1.095197468 | | | KD3956 | 0 | | 0 | | 0 | | 0 | | 0 | | 0 | | 0 | |  |  |
| 373 | F | | 382 | | 681 | | SP2361 | | 0 | | 0 | | 0 | | 0 | | 0.00 | | 0.00 | | 0 | | | LV3161 | 0 | | 0 | | 0 | | 1 | | 0 | | 36.692 | | 2.055687354 | | | KD3963 | 0 | | 0 | | 0 | | 0 | | 0 | | 0 | | 0 | |  |  |
| 374 | M | | 358 | | 164 | | SP2359 | | 0 | | 0 | | 0 | | 0 | | 0.00 | | 0.00 | | 0 | | | LV3159 | 0 | | 0 | | 1 | | 1 | | 36.06 | | 37.271 | | 2.273666709 | | | KD3961 | 0 | | 0 | | 0 | | 0 | | 0 | | 0 | | 0 | |  |  |
| 375 | F | | 345 | | 520 | | SP2366 | | 0 | | 0 | | 0 | | 0 | | 0.00 | | 0.00 | | 0 | | | LV3166 | 0 | | 0 | | 0 | | 1 | | 0 | | 36.463 | | 2.402677094 | | | KD3968 | 0 | | 0 | | 0 | | 0 | | 0 | | 0 | | 0 | |  |  |
| 376 | M | | 246 | | 151 | | SP2357 | | 0 | | 0 | | 0 | | 0 | | 0.00 | | 0.00 | | 0 | | | LV3157 | 0 | | 0 | | 1 | | 1 | | 36.558 | | 36.414 | | 2.368176128 | | | KD3959 | 0 | | 0 | | 0 | | 0 | | 0 | | 0 | | 0 | |  |  |
| 377 | F | | 304 | | 287 | | SP2370 | | 0 | | 0 | | 0 | | 0 | | 0.00 | | 0.00 | | 0 | | | LV3170 | 0 | | 0 | | 1 | | 0 | | 36.555 | | 0 | | 2.256741162 | | | KD3972 | 0 | | 0 | | 0 | | 0 | | 0 | | 0 | | 0 | |  |  |
| 378 | M | | 250 | | 139 | | SP2355 | | 0 | | 0 | | 0 | | 0 | | 0.00 | | 39.550 | | 0 | | | LV3155 | 0 | | 0 | | 1 | | 1 | | 37.143 | | 36.03 | | 2.369417283 | | | KD3957 | 0 | | 0 | | 0 | | 0 | | 0 | | 0 | | 0 | |  |  |
| 379 | F | | 339 | | 441 | | SP2375 | | 0 | | 0 | | 0 | | 0 | | 0.00 | | 0.00 | | 0 | | | LV3175 | 0 | | 0 | | 0 | | 0 | | 37.987 | | 0 | | 0 | | | KD4002 | 0 | | 0 | | 0 | | 0 | | 0 | | 0 | | 0 | |  |  |
| 380 | M | | 269 | | 171 | | SP2360 | | 0 | | 0 | | 0 | | 0 | | 0.00 | | 0.00 | | 0 | | | LV3160 | 0 | | 0 | | 1 | | 1 | | 36.358 | | 36.966 | | 2.143260271 | | | KD3962 | 0 | | 0 | | 0 | | 0 | | 0 | | 0 | | 0 | |  |  |
| 381 | F | | 401 | | 736 | | SP2373 | | 0 | | 0 | | 0 | | 0 | | 0.00 | | 0.00 | | 0 | | | LV3173 | 0 | | 0 | | 0 | | 0 | | 37.842 | | 0 | | 0 | | | KD3975 | 0 | | 0 | | 0 | | 0 | | 0 | | 0 | | 0 | |  |  |
| 382 | M | | 272 | | 213 | | SP2362 | | 0 | | 0 | | 0 | | 0 | | 0.00 | | 0.00 | | 0 | | | LV3162 | 0 | | 0 | | 1 | | 1 | | 35.921 | | 36.407 | | 2.985803234 | | | KD3964 | 0 | | 0 | | 0 | | 0 | | 0 | | 0 | | 0 | |  |  |
| 383 | F | | 385 | | 688 | | SP2403 | | 0 | | 0 | | 0 | | 1 | | 38.14 | | 37.165 | | 1.489509808 | | | LV3178 | 0 | | 0 | | 1 | | 0 | | 35.345 | | 38.046 | | 5.145179257 | | | KD4005 | 0 | | 0 | | 0 | | 0 | | 0 | | 0 | | 0 | |  |  |
| 384 | M | | 243 | | 134 | | SP2364 | | 0 | | 0 | | 0 | | 0 | | 0.00 | | 0.00 | | 0 | | | LV3164 | 0 | | 0 | | 1 | | 1 | | 36.288 | | 36.514 | | 2.513738372 | | | KD3966 | 0 | | 0 | | 0 | | 0 | | 0 | | 0 | | 0 | |  |  |
| 385 | F | | 399 | | 713 | | SP2405 | | 0 | | 0 | | 0 | | 0 | | 0.00 | | 0.00 | | 0 | | | LV3180 | 0 | | 0 | | 0 | | 0 | | 0 | | 0 | | 0 | | | KD4007 | 0 | | 0 | | 0 | | 0 | | 0 | | 0 | | 0 | |  |  |
| 386 | M | | 247 | | 172 | | SP2367 | | 0 | | 0 | | 0 | | 0 | | 0.00 | | 0.00 | | 0 | | | LV3167 | 0 | | 0 | | 1 | | 1 | | 36.522 | | 37.692 | | 1.674166985 | | | KD3969 | 0 | | 0 | | 0 | | 0 | | 0 | | 0 | | 0 | |  |  |
| 387 | F | | 404 | | 835 | | SP2407 | | 0 | | 0 | | 0 | | 0 | | 0.00 | | 38.191 | | 0 | | | LV3182 | 0 | | 0 | | 0 | | 0 | | 0 | | 0 | | 0 | | | KD4009 | 0 | | 0 | | 0 | | 0 | | 0 | | 0 | | 0 | |  |  |
| 388 | M | | 244 | | 153 | | SP2365 | | 0 | | 0 | | 0 | | 0 | | 0.00 | | 0.00 | | 0 | | | LV3165 | 0 | | 0 | | 0 | | 0 | | 0 | | 0 | | 0 | | | KD3967 | 0 | | 0 | | 0 | | 0 | | 0 | | 0 | | 0 | |  |  |
| 389 | F | | 335 | | 460 | | SP2404 | | 0 | | 0 | | 0 | | 0 | | 0.00 | | 0.00 | | 0 | | | LV3179 | 0 | | 0 | | 1 | | 1 | | 37.208 | | 36.876 | | 1.630034613 | | | KD4006 | 0 | | 0 | | 0 | | 0 | | 0 | | 0 | | 0 | |  |  |
| 390 | M | | 237 | | 130 | | SP2369 | | 0 | | 0 | | 0 | | 0 | | 0.00 | | 0.00 | | 0 | | | LV3169 | 0 | | 0 | | 0 | | 1 | | 37.88 | | 37.125 | | 1.530648226 | | | KD3971 | 0 | | 0 | | 0 | | 0 | | 0 | | 0 | | 0 | |  |  |
| 391 | F | | 351 | | 549 | | SP2410 | | 0 | | 0 | | 0 | | 0 | | 0.00 | | 0.00 | | 0 | | | LV3185 | 0 | | 0 | | 0 | | 0 | | 37.925 | | 0 | | 0 | | | KD4012 | 0 | | 0 | | 0 | | 0 | | 39.744 | | 0 | | 0 | |  |  |
| 392 | M | | 242 | | 151 | | SP2374 | | 0 | | 0 | | 0 | | 0 | | 0.00 | | 0.00 | | 0 | | | LV3174 | 0 | | 0 | | 0 | | 0 | | 0 | | 0 | | 0 | | | KD4001 | 0 | | 0 | | 0 | | 0 | | 0 | | 0 | | 0 | |  |  |
| 393 | F | | 332 | | 453 | | SP2402 | | 0 | | 0 | | 0 | | 0 | | 37.84 | | 0.00 | | 0 | | | LV3177 | 0 | | 0 | | 0 | | 0 | | 0 | | 0 | | 0 | | | KD4004 | 1 | | 0 | | 0 | | 0 | | 0 | | 0 | | 0 | |  |  |
| 394 | M | | 210 | | 108 | | SP2371 | | 0 | | 0 | | 0 | | 0 | | 0.00 | | 39.496 | | 0 | | | LV3171 | 0 | | 0 | | 0 | | 1 | | 0 | | 34.603 | | 8.528734128 | | | KD3973 | 0 | | 0 | | 0 | | 0 | | 0 | | 0 | | 0 | |  |  |
| 395 | F | | 380 | | 736 | | SP2412 | | 0 | | 0 | | 0 | | 0 | | 0.00 | | 0.00 | | 0 | | | LV3188 | 0 | | 0 | | 0 | | 0 | | 0 | | 0 | | 0 | | | KD4014 | 0 | | 0 | | 0 | | 0 | | 0 | | 0 | | 0 | |  |  |
| 396 | M | | 236 | | 131 | | SP2372 | | 0 | | 0 | | 0 | | 0 | | 0.00 | | 0.00 | | 0 | | | LV3172 | 0 | | 0 | | 0 | | 0 | | 37.838 | | 0 | | 0 | | | KD3974 | 0 | | 0 | | 0 | | 0 | | 0 | | 0 | | 0 | |  |  |
| 397 | F | | 316 | | 518 | | SP2414 | | 0 | | 0 | | 0 | | 0 | | 0.00 | | 0.00 | | 0 | | | LV3189 | 0 | | 0 | | 0 | | 0 | | 0 | | 0 | | 0 | | | KD4016 | 0 | | 0 | | 0 | | 0 | | 0 | | 0 | | 0 | |  |  |
| 398 | M | | 228 | | 118 | | SP2415 | | 0 | | 0 | | 0 | | 0 | | 0.00 | | 0.00 | | 0 | | | LV3190 | 0 | | 0 | | 0 | | 0 | | 0 | | 0 | | 0 | | | KD4017 | 0 | | 0 | | 0 | | 0 | | 0 | | 0 | | 0 | |  |  |
| 399 | F | | 352 | | 517 | | SP2406 | | 0 | | 0 | | 0 | | 0 | | 0.00 | | 0.00 | | 0 | | | LV3181 | 0 | | 0 | | 1 | | 0 | | 37.331 | | 0 | | 1.33027204 | | | KD4008 | 0 | | 0 | | 0 | | 0 | | 0 | | 0 | | 0 | |  |  |
| 400 | M | | 262 | | 195 | | SP2409 | | 0 | | 0 | | 0 | | 0 | | 0.00 | | 0.00 | | 0 | | | LV3184 | 0 | | 0 | | 1 | | 0 | | 37.063 | | 0 | | 1.596669507 | | | KD4011 | 0 | | 0 | | 0 | | 0 | | 0 | | 0 | | 0 | |  |  |
| 401 | F | | 361 | | 572 | | SP2419 | | 0 | | 0 | | 0 | | 1 | | 0.00 | | 37.645 | | 1.074135489 | | | LV3194 | 0 | | 0 | | 1 | | 0 | | 25.226 | | 0 | | 5065.322065 | | | KD4021 | 0 | | 0 | | 0 | | 0 | | 0 | | 0 | | 0 | |  |  |
| 402 | M | | 253 | | 152 | | SP2408 | | 0 | | 0 | | 0 | | 1 | | 38.96 | | 36.811 | | 1.895644039 | | | LV3183 | 0 | | 0 | | 0 | | 0 | | 0 | | 0 | | 0 | | | KD4010 | 0 | | 1 | | 0 | | 0 | | 0 | | 0 | | 0 | |  |  |
| 403 | F | | 353 | | 510 | | SP2422 | | 0 | | 0 | | 0 | | 0 | | 0.00 | | 0.00 | | 0 | | | LV3197 | 0 | | 0 | | 1 | | 0 | | 37.511 | | 0 | | 1.176782903 | | | KD4024 | 0 | | 0 | | 0 | | 0 | | 0 | | 0 | | 0 | |  |  |
| 404 | M | | 263 | | 157 | | SP2416 | | 0 | | 0 | | 0 | | 0 | | 0.00 | | 0.00 | | 0 | | | LV3191 | 0 | | 0 | | 0 | | 0 | | 0 | | 0 | | 0 | | | KD4018 | 0 | | 0 | | 0 | | 0 | | 0 | | 0 | | 0 | |  |  |
| 405 | F | | 346 | | 516 | | SP2417 | | 0 | | 0 | | 0 | | 0 | | 0.00 | | 0.00 | | 0 | | | LV3192 | 0 | | 0 | | 1 | | 1 | | 36.825 | | 37.695 | | 1.457912699 | | | KD4019 | 0 | | 0 | | 0 | | 0 | | 0 | | 0 | | 0 | |  |  |
| 406 | M | | 259 | | 184 | | SP2413 | | 0 | | 0 | | 0 | | 0 | | 0.00 | | 0.00 | | 0 | | | LV3187 | 0 | | 0 | | 0 | | 0 | | 0 | | 0 | | 0 | | | KD4015 | 0 | | 0 | | 0 | | 0 | | 0 | | 0 | | 0 | |  |  |
| 407 | F | | 306 | | 306 | | SP2424 | | 0 | | 0 | | 0 | | 0 | | 0.00 | | 0.00 | | 0 | | | LV3200 | 0 | | 0 | | 1 | | 1 | | 36.824 | | 36.022 | | 2.561700651 | | | KD3977 | 0 | | 0 | | 0 | | 0 | | 0 | | 0 | | 0 | |  |  |
| 408 | M | | 207 | | 116 | | SP2411 | | 0 | | 0 | | 0 | | 0 | | 0.00 | | 0.00 | | 0 | | | LV3186 | 0 | | 0 | | 1 | | 0 | | 35.901 | | 0 | | 3.523184292 | | | KD4013 | 0 | | 0 | | 0 | | 0 | | 0 | | 0 | | 0 | |  |  |
| 409 | F | | 364 | | 553 | | SP2427 | | 0 | | 0 | | 0 | | 0 | | 0.00 | | 0.00 | | 0 | | | LV3202 | 0 | | 0 | | 0 | | 1 | | 38.339 | | 36.919 | | 1.761206671 | | | KD3979 | 0 | | 0 | | 0 | | 0 | | 0 | | 0 | | 0 | |  |  |
| 410 | M | | 276 | | 223 | | SP2420 | | 0 | | 0 | | 0 | | 0 | | 0.00 | | 0.00 | | 0 | | | LV3195 | 0 | | 0 | | 0 | | 0 | | 0 | | 0 | | 0 | | | KD4022 | 0 | | 0 | | 0 | | 0 | | 0 | | 0 | | 0 | |  |  |
| 411 | F | | 394 | | 726 | | SP2429 | | 0 | | 0 | | 0 | | 0 | | 0.00 | | 0.00 | | 0 | | | LV3204 | 0 | | 0 | | 0 | | 1 | | 0 | | 37.283 | | 1.374481482 | | | KD3981 | 0 | | 0 | | 0 | | 0 | | 0 | | 0 | | 0 | |  |  |
| 412 | M | | 275 | | 218 | | SP2418 | | 0 | | 0 | | 0 | | 0 | | 0.00 | | 0.00 | | 0 | | | LV3193 | 0 | | 0 | | 0 | | 0 | | 0 | | 0 | | 0 | | | KD4020 | 0 | | 0 | | 0 | | 0 | | 0 | | 0 | | 0 | |  |  |
| 413 | F | | 304 | | 309 | | SP2428 | | 0 | | 0 | | 0 | | 0 | | 0.00 | | 0.00 | | 0 | | | LV3203 | 0 | | 0 | | 1 | | 0 | | 36.658 | | 0 | | 2.103847643 | | | KD3980 | 0 | | 0 | | 0 | | 0 | | 0 | | 0 | | 0 | |  |  |
| 414 | M | | 239 | | 131 | | SP2421 | | 0 | | 0 | | 0 | | 0 | | 0.00 | | 0.00 | | 0 | | | LV3196 | 0 | | 0 | | 1 | | 0 | | 36.902 | | 0 | | 1.781717856 | | | KD4023 | 0 | | 0 | | 0 | | 0 | | 0 | | 0 | | 0 | |  |  |
| 415 | F | | 321 | | 369 | | SP2431 | | 0 | | 0 | | 0 | | 0 | | 0.00 | | 0.00 | | 0 | | | LV3206 | 0 | | 0 | | 0 | | 0 | | 38.443 | | 0 | | 0 | | | KD3983 | 0 | | 0 | | 0 | | 0 | | 0 | | 0 | | 0 | |  |  |
| 416 | M | | 256 | | 176 | | SP2426 | | 0 | | 0 | | 0 | | 0 | | 0.00 | | 0.00 | | 0 | | | LV3201 | 0 | | 0 | | 1 | | 1 | | 35.667 | | 36.102 | | 3.602169759 | | | KD3978 | 0 | | 0 | | 0 | | 0 | | 0 | | 0 | | 0 | |  |  |
| 417 | F | | 381 | | 685 | | SP2436 | | 0 | | 0 | | 0 | | 0 | | 0.00 | | 0.00 | | 0 | | | LV3211 | 0 | | 0 | | 0 | | 1 | | 0 | | 35.987 | | 3.322741128 | | | KD3988 | 0 | | 0 | | 0 | | 0 | | 0 | | 0 | | 0 | |  |  |
| 418 | M | | 277 | | 221 | | SP2423 | | 0 | | 0 | | 0 | | 0 | | 0.00 | | 0.00 | | 0 | | | LV3198 | 0 | | 0 | | 0 | | 0 | | 38.052 | | 37.93 | | 0 | | | KD4025 | 0 | | 0 | | 0 | | 0 | | 0 | | 0 | | 0 | |  |  |
| 419 | F | | 353 | | 516 | | SP2433 | | 0 | | 0 | | 0 | | 0 | | 0.00 | | 0.00 | | 0 | | | LV3209 | 0 | | 0 | | 0 | | 0 | | 0 | | 0 | | 0 | | | KD3986 | 0 | | 0 | | 0 | | 0 | | 0 | | 0 | | 0 | |  |  |
| 420 | M | | 238 | | 142 | | SP2425 | | 0 | | 0 | | 0 | | 0 | | 0.00 | | 0.00 | | 0 | | | LV3199 | 0 | | 0 | | 0 | | 0 | | 0 | | 0 | | 0 | | | KD3976 | 0 | | 0 | | 0 | | 0 | | 0 | | 0 | | 0 | |  |  |
| 421 | F | | 356 | | 522 | | SP2438 | | 0 | | 0 | | 0 | | 0 | | 0.00 | | 0.00 | | 0 | | | LV3213 | 0 | | 0 | | 0 | | 0 | | 0 | | 0 | | 0 | | | KD3990 | 0 | | 0 | | 0 | | 0 | | 0 | | 0 | | 0 | |  |  |
| 422 | M | | 222 | | 109 | | SP2379 | | 0 | | 0 | | 0 | | 0 | | 0.00 | | 0.00 | | 0 | | | LV3229 | 0 | | 0 | | 0 | | 1 | | 0 | | 37.69 | | 1.041712889 | | | KD4031 | 0 | | 0 | | 0 | | 0 | | 0 | | 0 | | 0 | |  |  |
| 423 | F | | 382 | | 619 | | SP2442 | | 0 | | 0 | | 0 | | 0 | | 0.00 | | 0.00 | | 0 | | | LV3217 | 0 | | 0 | | 0 | | 1 | | 0 | | 36.568 | | 2.236847323 | | | KD3994 | 0 | | 0 | | 0 | | 0 | | 0 | | 0 | | 0 | |  |  |
| 424 | M | | 259 | | 176 | | SP2432 | | 0 | | 0 | | 0 | | 0 | | 0.00 | | 0.00 | | 0 | | | LV3207 | 0 | | 0 | | 1 | | 0 | | 37.509 | | 0 | | 1.178387023 | | | KD3984 | 0 | | 0 | | 0 | | 0 | | 0 | | 0 | | 0 | |  |  |
| 425 | F | | 395 | | 598 | | SP2440 | | 0 | | 0 | | 0 | | 0 | | 0.00 | | 0.00 | | 0 | | | LV3215 | 0 | | 0 | | 0 | | 0 | | 0 | | 38 | | 0 | | | KD3992 | 0 | | 0 | | 0 | | 0 | | 0 | | 0 | | 0 | |  |  |
| 426 | M | | 215 | | 98 | | SP2430 | | 0 | | 0 | | 0 | | 0 | | 0.00 | | 0.00 | | 0 | | | LV3205 | 0 | | 0 | | 1 | | 1 | | 36.497 | | 36.551 | | 2.305287005 | | | KD3982 | 0 | | 0 | | 0 | | 0 | | 0 | | 0 | | 0 | |  |  |
| 427 | F | | 375 | | 662 | | SP2446 | | 0 | | 0 | | 0 | | 0 | | 0.00 | | 0.00 | | 0 | | | LV3221 | 0 | | 0 | | 0 | | 0 | | 0 | | 0 | | 0 | | | KD3998 | 0 | | 0 | | 0 | | 0 | | 0 | | 0 | | 0 | |  |  |
| 428 | M | | 248 | | 144 | | SP2437 | | 0 | | 0 | | 0 | | 0 | | 0.00 | | 0.00 | | 0 | | | LV3212 | 0 | | 0 | | 0 | | 0 | | 0 | | 0 | | 0 | | | KD3989 | 0 | | 0 | | 0 | | 0 | | 0 | | 0 | | 0 | |  |  |
| 429 | F | | 331 | | 459 | | SP2448 | | 0 | | 0 | | 0 | | 0 | | 0.00 | | 0.00 | | 0 | | | LV3223 | 0 | | 0 | | 0 | | 1 | | 0 | | 37.626 | | 1.088126226 | | | KD4000 | 0 | | 0 | | 0 | | 0 | | 0 | | 0 | | 0 | |  |  |
| 450 | M | | 245 | | 143 | | SP2435 | | 0 | | 0 | | 0 | | 0 | | 0.00 | | 0.00 | | 0 | | | LV3210 | 0 | | 0 | | 0 | | 0 | | 0 | | 0 | | 0 | | | KD3987 | 0 | | 0 | | 0 | | 0 | | 0 | | 39.313 | | 0 | |  |  |
| 451 | F | | 374 | | 586 | | SP2385 | | 0 | | 0 | | 0 | | 0 | | 0.00 | | 0.00 | | 0 | | | LV3235 | 0 | | 0 | | 0 | | 0 | | 38.759 | | 37.94 | | 0 | | | KD4037 | 0 | | 0 | | 0 | | 0 | | 0 | | 0 | | 0 | |  |  |
| 452 | M | | 232 | | 128 | | SP2434 | | 0 | | 0 | | 0 | | 0 | | 0.00 | | 0.00 | | 0 | | | LV3208 | 0 | | 0 | | 0 | | 1 | | 0 | | 37.711 | | 1.026919083 | | | KD3985 | 0 | | 0 | | 0 | | 0 | | 0 | | 0 | | 0 | |  |  |
| 453 | F | | 363 | | 548 | | SP2384 | | 0 | | 0 | | 0 | | 0 | | 0.00 | | 0.00 | | 0 | | | LV3234 | 0 | | 0 | | 1 | | 0 | | 36.637 | | 0 | | 2.134155691 | | | KD4036 | 0 | | 0 | | 0 | | 0 | | 0 | | 0 | | 0 | |  |  |
| 454 | M | | 231 | | 118 | | SP2441 | | 0 | | 0 | | 0 | | 0 | | 0.00 | | 0.00 | | 0 | | | LV3216 | 0 | | 0 | | 0 | | 0 | | 0 | | 0 | | 0 | | | KD3993 | 0 | | 0 | | 0 | | 0 | | 0 | | 0 | | 0 | |  |  |
| 455 | F | | 334 | | 448 | | SP2377 | | 0 | | 0 | | 0 | | 0 | | 0.00 | | 0.00 | | 0 | | | LV3227 | 0 | | 0 | | 1 | | 0 | | 36.965 | | 0 | | 1.706881978 | | | KD4029 | 0 | | 0 | | 0 | | 0 | | 0 | | 0 | | 0 | |  |  |
| 456 | M | | 253 | | 169 | | SP2439 | | 0 | | 0 | | 0 | | 0 | | 0.00 | | 0.00 | | 0 | | | LV3214 | 0 | | 0 | | 0 | | 0 | | 0 | | 38.919 | | 0 | | | KD3991 | 0 | | 0 | | 0 | | 0 | | 0 | | 0 | | 0 | |  |  |
| 457 | F | | 316 | | 340 | | SP2381 | | 0 | | 0 | | 0 | | 0 | | 0.00 | | 0.00 | | 0 | | | LV3231 | 0 | | 0 | | 0 | | 0 | | 0 | | 37.873 | | 0 | | | KD4033 | 0 | | 0 | | 0 | | 0 | | 0 | | 0 | | 0 | |  |  |
| 458 | M | | 233 | | 129 | | SP2376 | | 0 | | 0 | | 0 | | 0 | | 0.00 | | 0.00 | | 0 | | | LV3226 | 0 | | 0 | | 0 | | 0 | | 0 | | 0 | | 0 | | | KD4028 | 0 | | 0 | | 0 | | 0 | | 0 | | 0 | | 0 | |  |  |
| 459 | F | | 366 | | 551 | | SP2450 | | 0 | | 0 | | 0 | | 0 | | 0.00 | | 0.00 | | 0 | | | LV3225 | 0 | | 0 | | 0 | | 0 | | 0 | | 37.893 | | 0 | | | KD4027 | 0 | | 0 | | 0 | | 0 | | 0 | | 0 | | 0 | |  |  |
| 460 | M | | 250 | | 161 | | SP2444 | | 0 | | 0 | | 0 | | 0 | | 0.00 | | 0.00 | | 0 | | | LV3219 | 0 | | 0 | | 0 | | 0 | | 0 | | 0 | | 0 | | | KD3996 | 0 | | 0 | | 0 | | 0 | | 0 | | 0 | | 0 | |  |  |
| 461 | F | | 349 | | 506 | | SP2383 | | 0 | | 0 | | 0 | | 0 | | 0.00 | | 0.00 | | 0 | | | LV3233 | 0 | | 0 | | 0 | | 1 | | 0 | | 36.438 | | 2.443939349 | | | KD4035 | 0 | | 0 | | 0 | | 0 | | 0 | | 0 | | 0 | |  |  |
| 462 | M | | 221 | | 102 | | SP2443 | | 0 | | 0 | | 0 | | 0 | | 0.00 | | 0.00 | | 0 | | | LV3218 | 0 | | 0 | | 0 | | 0 | | 0 | | 0 | | 0 | | | KD3995 | 0 | | 0 | | 0 | | 0 | | 0 | | 0 | | 0 | |  |  |
| 463 | F | | 296 | | 343 | | SP2386 | | 0 | | 0 | | 0 | | 0 | | 0.00 | | 0.00 | | 0 | | | LV3236 | 0 | | 0 | | 1 | | 0 | | 37.55 | | 0 | | 1.145935373 | | | KD4038 | 0 | | 0 | | 0 | | 0 | | 0 | | 0 | | 0 | |  |  |
| 464 | M | | 285 | | 209 | | SP2378 | | 0 | | 0 | | 0 | | 0 | | 0.00 | | 0.00 | | 0 | | | LV3228 | 0 | | 0 | | 0 | | 1 | | 37.801 | | 36 | | 3.293450184 | | | KD4030 | 0 | | 0 | | 0 | | 0 | | 0 | | 0 | | 0 | |  |  |
| 465 | F | | 373 | | 586 | | SP2380 | | 0 | | 0 | | 0 | | 0 | | 0.00 | | 0.00 | | 0 | | | LV3230 | 0 | | 0 | | 0 | | 0 | | 0 | | 37.929 | | 0 | | | KD4032 | 0 | | 0 | | 0 | | 0 | | 0 | | 0 | | 0 | |  |  |
| 466 | M | | 229 | | 126 | | SP2447 | | 0 | | 0 | | 0 | | 0 | | 0.00 | | 0.00 | | 0 | | | LV3222 | 0 | | 0 | | 1 | | 0 | | 37.484 | | 0 | | 1.198623994 | | | KD3999 | 0 | | 0 | | 0 | | 0 | | 0 | | 0 | | 0 | |  |  |
| 467 | F | | 375 | | 650 | | SP2382 | | 0 | | 0 | | 0 | | 0 | | 0.00 | | 0.00 | | 0 | | | LV3232 | 0 | | 0 | | 1 | | 0 | | 37.597 | | 0 | | 1.109832647 | | | KD4034 | 0 | | 0 | | 0 | | 0 | | 0 | | 0 | | 0 | |  |  |
| 468 | M | | 284 | | 214 | | SP2445 | | 0 | | 0 | | 0 | | 0 | | 0.00 | | 0.00 | | 0 | | | LV3220 | 0 | | 0 | | 0 | | 0 | | 0 | | 38.457 | | 0 | | | KD3997 | 0 | | 0 | | 0 | | 0 | | 0 | | 0 | | 0 | |  |  |
| 469 | F | | 290 | | 297 | | SP2387 | | 0 | | 0 | | 0 | | 0 | | 0.00 | | 0.00 | | 0 | | | LV3237 | 0 | | 0 | | 1 | | 0 | | 36.942 | | 0 | | 1.733831574 | | | KD4039 | 0 | | 0 | | 0 | | 0 | | 0 | | 0 | | 0 | |  |  |
| 470 | M | | 275 | | 202 | | SP2449 | | 0 | | 0 | | 0 | | 0 | | 0.00 | | 0.00 | | 0 | | | LV3224 | 0 | | 0 | | 1 | | 0 | | 37.054 | | 0 | | 1.60648708 | | | KD4026 | 0 | | 0 | | 0 | | 0 | | 0 | | 0 | | 0 | |  |  |
| 472 | M | | 260 | | 167 | | SP2519 | | 0 | | 0 | | 0 | | 0 | | 0.00 | | 0.00 | | 0 | | | LV3319 | 0 | | 0 | | 0 | | 0 | | 37.966 | | 0 | | 0 | | | KD4122 | 0 | | 0 | | 0 | | 0 | | 0 | | 0 | | 0 | |  |  |
| 473 | F | | 370 | | 678 | | SP2389 | | 0 | | 0 | | 0 | | 0 | | 0.00 | | 0.00 | | 0 | | | LV3239 | 0 | | 0 | | 0 | | 1 | | 0 | | 36.039 | | 3.207117521 | | | KD4041 | 0 | | 0 | | 0 | | 0 | | 0 | | 0 | | 0 | |  |  |
| 474 | M | | 230 | | 111 | | SP2388 | | 0 | | 0 | | 0 | | 0 | | 0.00 | | 0.00 | | 0 | | | LV3238 | 0 | | 0 | | 0 | | 1 | | 38.574 | | 37.452 | | 1.225035254 | | | KD4040 | 0 | | 0 | | 0 | | 0 | | 0 | | 0 | | 0 | |  |  |
| 475 | F | | 320 | | 443 | | SP2394 | | 0 | | 0 | | 0 | | 0 | | 0.00 | | 0.00 | | 0 | | | LV3244 | 0 | | 0 | | 1 | | 0 | | 37.725 | | 0 | | 1.01717345 | | | KD4046 | 0 | | 0 | | 0 | | 0 | | 0 | | 0 | | 0 | |  |  |
| 476 | M | | 210 | | 108 | | SP2390 | | 0 | | 0 | | 0 | | 0 | | 0.00 | | 0.00 | | 0 | | | LV3240 | 0 | | 0 | | 0 | | 1 | | 0 | | 37.715 | | 1.024125128 | | | KD4042 | 0 | | 0 | | 0 | | 0 | | 0 | | 0 | | 0 | |  |  |
| 477 | F | | 340 | | 487 | | SP2399 | | 0 | | 0 | | 0 | | 0 | | 0.00 | | 0.00 | | 0 | | | LV3249 | 0 | | 1 | | 0 | | 1 | | 0 | | 35.919 | | 3.480254067 | | | KD4051 | 0 | | 0 | | 0 | | 0 | | 0 | | 0 | | 0 | |  |  |
| 478 | M | | 260 | | 168 | | SP2395 | | 0 | | 0 | | 0 | | 0 | | 0.00 | | 0.00 | | 0 | | | LV3245 | 0 | | 0 | | 0 | | 1 | | 38.061 | | 37.629 | | 1.085905108 | | | KD4047 | 0 | | 0 | | 0 | | 0 | | 0 | | 0 | | 0 | |  |  |
| 479 | F | | 411 | | 903 | | SP2397 | | 0 | | 0 | | 0 | | 0 | | 0.00 | | 0.00 | | 0 | | | LV3247 | 0 | | 0 | | 0 | | 0 | | 0 | | 37.793 | | 0 | | | KD4049 | 0 | | 0 | | 0 | | 0 | | 0 | | 0 | | 0 | |  |  |
| 480 | M | | 232 | | 113 | | SP2391 | | 0 | | 0 | | 0 | | 0 | | 0.00 | | 0.00 | | 0 | | | LV3241 | 0 | | 0 | | 0 | | 1 | | 0 | | 36.499 | | 2.344480287 | | | KD4043 | 0 | | 0 | | 0 | | 0 | | 0 | | 0 | | 0 | |  |  |
| 481 | F | | 345 | | 567 | | SP2451 | | 0 | | 0 | | 0 | | 0 | | 0.00 | | 0.00 | | 0 | | | LV3251 | 0 | | 0 | | 1 | | 1 | | 37.586 | | 37.106 | | 1.334382009 | | | KD4053 | 0 | | 0 | | 0 | | 0 | | 0 | | 0 | | 0 | |  |  |
| 482 | M | | 220 | | 158 | | SP2393 | | 0 | | 0 | | 0 | | 0 | | 0.00 | | 0.00 | | 0 | | | LV3243 | 0 | | 0 | | 0 | | 0 | | 0 | | 0 | | 0 | | | KD4045 | 0 | | 0 | | 0 | | 0 | | 0 | | 0 | | 0 | |  |  |
| 483 | F | | 315 | | 344 | | SP2454 | | 0 | | 0 | | 0 | | 0 | | 0.00 | | 0.00 | | 0 | | | LV3254 | 1 | | 1 | | 1 | | 0 | | 37.7 | | 0 | | 1.034641827 | | | KD4056 | 0 | | 0 | | 0 | | 0 | | 0 | | 0 | | 0 | |  |  |
| 484 | M | | 210 | | 98 | | SP2455 | | 0 | | 0 | | 0 | | 0 | | 0.00 | | 0.00 | | 0 | | | LV3255 | 0 | | 0 | | 0 | | 0 | | 0 | | 0 | | 0 | | | KD4057 | 0 | | 0 | | 0 | | 0 | | 0 | | 0 | | 0 | |  |  |
| 485 | F | | 375 | | 602 | | SP2458 | | 0 | | 0 | | 0 | | 0 | | 0.00 | | 0.00 | | 0 | | | LV3258 | 0 | | 0 | | 1 | | 1 | | 33.931 | | 36.446 | | 7.954905877 | | | KD4063 |  | |  | |  | |  | |  | |  | |  | |  |  |
| 486 | M | | 250 | | 175 | | SP2398 | | 0 | | 0 | | 0 | | 0 | | 0.00 | | 0.00 | | 0 | | | LV3248 | 0 | | 0 | | 1 | | 1 | | 36.97 | | 36.843 | | 1.777926876 | | | KD4050 | 0 | | 0 | | 0 | | 0 | | 0 | | 0 | | 0 | |  |  |
| 487 | F | | 345 | | 522 | | SP2456 | | 0 | | 0 | | 0 | | 0 | | 0.00 | | 0.00 | | 0 | | | LV3256 | 0 | | 0 | | 0 | | 1 | | 0 | | 37.001 | | 1.665538478 | | | KD4058 | 0 | | 0 | | 0 | | 0 | | 0 | | 0 | | 0 | |  |  |
| 488 | M | | 231 | | 136 | | SP2396 | | 0 | | 0 | | 0 | | 0 | | 0.00 | | 0.00 | | 0 | | | LV3246 | 0 | | 0 | | 1 | | 1 | | 37.037 | | 36.711 | | 1.827226225 | | | KD4048 | 0 | | 0 | | 0 | | 0 | | 0 | | 0 | | 0 | |  |  |
| 489 | F | | 335 | | 417 | | SP2461 | | 0 | | 0 | | 0 | | 0 | | 0.00 | | 0.00 | | 0 | | | LV3261 | 0 | | 0 | | 0 | | 1 | | 38.341 | | 35.285 | | 5.359799549 | | | KD4065 | 0 | | 0 | | 1 | | 0 | | 36.868 | | 38.258 | | 1.823459634 | |  |  |
| 490 | M | | 226 | | 105 | | SP2400 | | 0 | | 0 | | 0 | | 0 | | 0.00 | | 0.00 | | 0 | | | LV3250 | 0 | | 1 | | 0 | | 0 | | 0 | | 0 | | 0 | | | KD4052 | 0 | | 0 | | 0 | | 0 | | 0 | | 0 | | 0 | |  |  |
| 491 | F | | 352 | | 508 | | SP2459 | | 0 | | 0 | | 0 | | 0 | | 0.00 | | 0.00 | | 0 | | | LV3259 | 0 | | 0 | | 0 | | 0 | | 38.576 | | 37.935 | | 0 | | | KD4061 | 0 | | 0 | | 1 | | 0 | | 37.336 | | 38.087 | | 1.325749463 | |  |  |
| 492 | M | | 233 | | 129 | | SP2453 | | 0 | | 0 | | 0 | | 0 | | 0.00 | | 0.00 | | 0 | | | LV3253 | 0 | | 1 | | 1 | | 0 | | 37.119 | | 38.289 | | 1.536916228 | | | KD4055 | 0 | | 0 | | 0 | | 0 | | 0 | | 0 | | 0 | |  |  |
| 493 | F | | 225 | | 410 | | SP2468 | | 0 | | 0 | | 0 | | 0 | | 0.00 | | 0.00 | | 0 | | | LV3268 | 0 | | 0 | | 1 | | 1 | | 37.392 | | 37.202 | | 1.364288415 | | | KD4071 | 0 | | 0 | | 0 | | 0 | | 0 | | 0 | | 0 | |  |  |
| 494 | M | | 236 | | 130 | | SP | |  | |  | | 0 | | 0 | | 0.00 | | 0.00 | | 0 | | | LV3257 | 1 | | 1 | | 0 | | 0 | | 0 | | 0 | | 0 | | | KD4059 | 0 | | 0 | | 0 | | 0 | | 0 | | 0 | | 0 | |  |  |
| 495 | F | | 374 | | 630 | | SP2463 | | 0 | | 0 | | 0 | | 0 | | 0.00 | | 0.00 | | 0 | | | LV3263 | 0 | | 0 | | 1 | | 1 | | 35.671 | | 33.519 | | 10.98316968 | | | KD4066 | 1 | | 1 | | 1 | | 0 | | 36.84 | | 37.916 | | 1.858568496 | |  |  |
| 496 | M | | 225 | | 121 | | SP2452 | | 0 | | 0 | | 0 | | 0 | | 0.00 | | 0.00 | | 0 | | | LV3252 | 0 | | 1 | | 0 | | 1 | | 0 | | 37.517 | | 1.171983632 | | | KD4054 | 0 | | 0 | | 0 | | 0 | | 0 | | 0 | | 0 | |  |  |
| 497 | F | | 334 | | 445 | | SP2465 | | 0 | | 0 | | 0 | | 0 | | 0.00 | | 0.00 | | 0 | | | LV3265 | 0 | | 0 | | 1 | | 1 | | 34.521 | | 33.79 | | 11.92822808 | | | KD4068 | 0 | | 0 | | 1 | | 0 | | 35.992 | | 0 | | 3.311444677 | |  |  |
| 498 | M | | 228 | | 131 | | SP2464 | | 1 | | 1 | | 1 | | 1 | | 28.27 | | 27.887 | | 732.2313132 | | | LV3264 | 1 | | 1 | | 1 | | 1 | | 20.815 | | 19.949 | | 143246.6787 | | | KD4067 | 1 | | 1 | | 1 | | 1 | | 25.417 | | 25.353 | | 4546.507475 | |  |  |
| 499 | F | | 310 | | 338 | | SP2470 | | 0 | | 0 | | 0 | | 0 | | 0.00 | | 0.00 | | 0 | | | LV3270 | 1 | | 0 | | 1 | | 1 | | 35.153 | | 34.127 | | 8.829335194 | | | KD4073 | 0 | | 0 | | 1 | | 0 | | 37.564 | | 0 | | 1.135060255 | |  |  |
| 500 | M | | 214 | | 99 | | SP2462 | | 0 | | 0 | | 0 | | 0 | | 0.00 | | 0.00 | | 0 | | | LV3262 | 1 | | 0 | | 1 | | 1 | | 36.025 | | 36.303 | | 2.958579283 | | | KD4064 | 0 | | 0 | | 0 | | 0 | | 0 | | 37.806 | | 0 | |  |  |
| 502 | M | | 240 | | 117 | | SP2460 | | 0 | | 0 | | 0 | | 0 | | 0.00 | | 0.00 | | 0 | | | LV3260 | 0 | | 1 | | 1 | | 1 | | 36.82 | | 36.221 | | 2.35863597 | | | KD4062 | 0 | | 0 | | 0 | | 0 | | 0 | | 0 | | 0 | |  |  |
| 503 | F | | 345 | | 586 | | SP2472 | | 1 | | 1 | | 1 | | 1 | | 21.00 | | 21.349 | | 80618.44851 | | | LV3272 | 1 | | 1 | | 1 | | 1 | | 21.681 | | 21.718 | | 55947.72793 | | | KD4075 | 1 | | 1 | | 1 | | 1 | | 23.886 | | 22.168 | | 26638.7526 | |  |  |
| 504 | M | | 235 | | 115 | | SP2475 | | 0 | | 0 | | 0 | | 0 | | 38.76 | | 39.214 | | 0 | | | LV3275 | 0 | | 1 | | 1 | | 1 | | 36.111 | | 36.272 | | 2.895061174 | | | KD4078 | 0 | | 0 | | 1 | | 0 | | 37.154 | | 38.247 | | 1.500711374 | |  |  |
| 505 | F | | 305 | | 350 | | SP2474 | | 0 | | 0 | | 0 | | 0 | | 0.00 | | 0.00 | | 0 | | | LV3274 | 0 | | 0 | | 1 | | 1 | | 34.233 | | 36.621 | | 6.565338719 | | | KD4077 | 1 | | 1 | | 1 | | 1 | | 37.693 | | 36.686 | | 1.551845951 | |  |  |
| 506 | M | | 219 | | 109 | | SP2466 | | 0 | | 0 | | 0 | | 0 | | 0.00 | | 0.00 | | 0 | | | LV3266 | 0 | | 0 | | 1 | | 1 | | 34.415 | | 35.704 | | 6.861461772 | | | KD4069 | 0 | | 0 | | 1 | | 0 | | 37.48 | | 37.92 | | 1.201894007 | |  |  |
| 507 | F | | 335 | | 560 | | SP2476 | | 0 | | 0 | | 1 | | 0 | | 37.48 | | 39.010 | | 1.201894007 | | | LV3276 | 1 | | 1 | | 1 | | 1 | | 36.15 | | 36.76 | | 2.468119181 | | | KD4079 | 0 | | 0 | | 1 | | 0 | | 36.368 | | 0 | | 2.563282472 | |  |  |
| 508 | M | | 225 | | 121 | | SP2467 | | 0 | | 0 | | 0 | | 0 | | 0.00 | | 0.00 | | 0 | | | LV3267 | 1 | | 1 | | 1 | | 1 | | 36.702 | | 36.554 | | 2.150006141 | | | KD4070 | 0 | | 0 | | 1 | | 0 | | 37.494 | | 0 | | 1.190487832 | |  |  |
| 509 | F | | 385 | | 654 | | SP2477 | | 0 | | 0 | | 0 | | 0 | | 0.00 | | 0.00 | | 0 | | | LV3277 | 0 | | 0 | | 1 | | 1 | | 35.483 | | 36.97 | | 3.192340732 | | | KD4080 | 0 | | 0 | | 0 | | 0 | | 38.23 | | 37.877 | | 0 | |  |  |
| 510 | M | | 245 | | 156 | | SP2471 | | 0 | | 0 | | 0 | | 0 | | 0.00 | | 0.00 | | 0 | | | LV3271 | 1 | | 0 | | 1 | | 1 | | 33.679 | | 36.266 | | 9.375400631 | | | KD4074 | 0 | | 0 | | 0 | | 1 | | 0 | | 35.07 | | 6.205055678 | |  |  |
| 511 | F | | 355 | | 632 | | SP2478 | | 0 | | 0 | | 0 | | 0 | | 0.00 | | 0.00 | | 0 | | | LV3278 | 0 | | 0 | | 1 | | 0 | | 36.193 | | 37.757 | | 2.8877632 | | | KD4081 | 0 | | 0 | | 1 | |  | | 36.921 | |  | | 1.758809167 | |  |  |
| 512 | M | | 246 | | 163 | | SP2473 | | 0 | | 0 | | 0 | | 0 | | 0.00 | | 0.00 | | 0 | | | LV3273 | 0 | | 0 | | 1 | | 1 | | 34.989 | | 36.181 | | 4.73423384 | | | KD4076 | 0 | | 0 | | 1 | | 1 | | 34.991 | | 35.173 | | 6.166372199 | |  |  |
| 513 | F | | 345 | | 522 | | SP2479 | | 0 | | 0 | | 0 | | 0 | | 0.00 | | 0.00 | | 0 | | | LV3279 | 0 | | 0 | | 0 | | 0 | | 37.805 | | 0 | | 0 | | | KD4082 | 0 | | 0 | | 0 | | 0 | | 0 | | 0 | | 0 | |  |  |
| 514 | M | | 247 | | 144 | | SP2469 | | 0 | | 0 | | 0 | | 0 | | 0.00 | | 0.00 | | 0 | | | LV3269 | 0 | | 0 | | 1 | | 1 | | 35.694 | | 32.747 | | 17.12427161 | | | KD4072 | 0 | | 0 | | 0 | | 0 | | 0 | | 0 | | 0 | |  |  |
| 515 | F | | 360 | | 629 | | SP2482 | | 0 | | 0 | | 0 | | 0 | | 0.00 | | 0.00 | | 0 | | | LV3282 | 0 | | 0 | | 0 | | 1 | | 0 | | 36.252 | | 2.77401842 | | | KD4085 | 0 | | 0 | | 0 | | 0 | | 0 | | 0 | | 0 | |  |  |
| 517 | F | | 365 | | 603 | | SP2481 | | 0 | | 0 | | 0 | | 0 | | 0.00 | | 0.00 | | 0 | | | LV3281 | 0 | | 0 | | 0 | | 1 | | 0 | | 36.945 | | 1.730292421 | | | KD4084 | 0 | | 0 | | 0 | | 0 | | 0 | | 0 | | 0 | |  |  |
| 518 | M | | 270 | | 197 | | SP2485 | | 0 | | 0 | | 0 | | 0 | | 0.00 | | 0.00 | | 0 | | | LV3285 | 0 | | 0 | | 0 | | 0 | | 0 | | 0 | | 0 | | | KD4088 | 0 | | 0 | | 0 | | 0 | | 0 | | 0 | | 0 | |  |  |
| 519 | F | | 310 | | 374 | | SP2484 | | 0 | | 0 | | 0 | | 0 | | 0.00 | | 0.00 | | 0 | | | LV3284 | 0 | | 0 | | 0 | | 0 | | 0 | | 0 | | 0 | | | KD4087 | 0 | | 0 | | 0 | | 0 | | 0 | | 38.556 | | 0 | |  |  |
| 520 | M | | 230 | | 118 | | SP2480 | | 0 | | 0 | | 0 | | 0 | | 0.00 | | 0.00 | | 0 | | | LV3280 | 0 | | 0 | | 0 | | 0 | | 0 | | 0 | | 0 | | | KD4083 | 0 | | 0 | | 0 | | 0 | | 0 | | 0 | | 0 | |  |  |
| 521 | F | | 353 | | 599 | | SP2486 | | 0 | | 0 | | 0 | | 0 | | 0.00 | | 0.00 | | 0 | | | LV3286 | 0 | | 0 | | 0 | | 1 | | 38.674 | | 37.635 | | 1.081476464 | | | KD4089 | 0 | | 0 | | 0 | | 0 | | 0 | | 0 | | 0 | |  |  |
| 522 | M | | 255 | | 163 | | SP2487 | | 0 | | 0 | | 0 | | 0 | | 0.00 | | 0.00 | | 0 | | | LV3287 | 0 | | 1 | | 0 | | 1 | | 0 | | 37.509 | | 1.178387023 | | | KD4090 | 0 | | 0 | | 0 | | 0 | | 39.726 | | 0 | | 0 | |  |  |
| 523 | F | | 334 | | 333 | | SP2492 | | 0 | | 0 | | 0 | | 0 | | 0.00 | | 0.00 | | 0 | | | LV3292 | 0 | | 0 | | 0 | | 0 | | 37.774 | | 0 | | 0 | | | KD4095 | 0 | | 0 | | 0 | | 0 | | 0 | | 0 | | 0 | |  |  |
| 524 | M | | 252 | | 144 | | SP2501 | | 0 | | 0 | | 0 | | 0 | | 0.00 | | 0.00 | | 0 | | | LV3301 | 0 | | 0 | | 0 | | 1 | | 0 | | 37.576 | | 1.125820907 | | | KD4104 | 0 | | 0 | | 0 | | 0 | | 0 | | 0 | | 0 | |  |  |
| 525 | F | | 360 | | 583 | | SP2497 | | 0 | | 0 | | 0 | | 0 | | 0.00 | | 0.00 | | 0 | | | LV3297 | 0 | | 1 | | 0 | | 1 | | 0 | | 36.917 | | 1.763607443 | | | KD4100 | 0 | | 0 | | 0 | | 0 | | 0 | | 0 | | 0 | |  |  |
| 526 | M | | 207 | | 89 | | SP2491 | | 0 | | 0 | | 0 | | 0 | | 0.00 | | 0.00 | | 0 | | | LV3291 | 0 | | 0 | | 1 | | 0 | | 37.543 | | 0 | | 1.151411943 | | | KD4094 | 0 | | 0 | | 0 | | 0 | | 0 | | 39.046 | | 0 | |  |  |
| 527 | F | | 545 | | 519 | | SP2494 | | 0 | | 0 | | 0 | | 0 | | 0.00 | | 0.00 | | 0 | | | LV3294 | 0 | | 0 | | 0 | | 1 | | 38.072 | | 37.166 | | 1.488495639 | | | KD4097 | 0 | | 0 | | 0 | | 0 | | 0 | | 0 | | 0 | |  |  |
| 528 | M | | 239 | | 121 | | SP2488 | | 0 | | 0 | | 0 | | 0 | | 0.00 | | 0.00 | | 0 | | | LV3288 | 0 | | 0 | | 0 | | 0 | | 0 | | 0 | | 0 | | | KD4091 | 0 | | 0 | | 0 | | 0 | | 0 | | 0 | | 0 | |  |  |
| 529 | F | | 315 | | 337 | | SP2500 | | 0 | | 0 | | 0 | | 0 | | 0.00 | | 0.00 | | 0 | | | LV3300 | 0 | | 0 | | 0 | | 0 | | 0 | | 0 | | 0 | | | KD4103 | 0 | | 0 | | 0 | | 0 | | 38.125 | | 0 | | 0 | |  |  |
| 530 | M | | 250 | | 182 | | SP2503 | | 0 | | 0 | | 0 | | 0 | | 0.00 | | 37.948 | | 0 | | | LV3303 | 0 | | 0 | | 0 | | 1 | | 37.787 | | 37.745 | | 1.003411336 | | | KD4106 | 0 | | 0 | | 1 | | 0 | | 36.976 | | 0 | | 1.694141519 | |  |  |
| 531 | F | | 342 | | 518 | | SP2502 | | 0 | | 0 | | 0 | | 0 | | 0.00 | | 39.150 | | 0 | | | LV3302 | 0 | | 0 | | 1 | | 0 | | 36.744 | | 0 | | 1.98415425 | | | KD4105 | 0 | | 0 | | 0 | | 1 | | 0 | | 37.117 | | 1.539011261 | |  |  |
| 532 | M | | 273 | | 205 | | SP2490 | | 0 | | 0 | | 0 | | 0 | | 0.00 | | 0.00 | | 0 | | | LV3290 | 1 | | 0 | | 1 | | 1 | | 36.479 | | 37.009 | | 2.016561745 | | | KD4093 | 0 | | 0 | | 0 | | 0 | | 0 | | 0 | | 0 | |  |  |
| 533 | F | | 326 | | 505 | | SP2505 | | 0 | | 0 | | 0 | | 0 | | 0.00 | | 0.00 | | 0 | | | LV3305 | 0 | | 0 | | 0 | | 0 | | 37.771 | | 0 | | 0 | | | KD4108 | 0 | | 0 | | 0 | | 0 | | 0 | | 38.091 | | 0 | |  |  |
| 534 | M | | 257 | | 154 | | SP2493 | | 0 | | 0 | | 0 | | 0 | | 0.00 | | 0.00 | | 0 | | | LV3293 | 0 | | 0 | | 1 | | 0 | | 37.65 | | 0 | | 1.07048371 | | | KD4096 | 0 | | 0 | | 0 | | 0 | | 0 | | 0 | | 0 | |  |  |
| 535 | F | | 338 | | 406 | | SP2507 | | 0 | | 0 | | 0 | | 0 | | 38.59 | | 0.00 | | 0 | | | LV3307 | 0 | | 0 | | 0 | | 0 | | 0 | | 0 | | 0 | | | KD4110 | 0 | | 0 | | 0 | | 0 | | 0 | | 0 | | 0 | |  |  |
| 536 | M | | 220 | | 99 | | SP2496 | | 0 | | 0 | | 0 | | 0 | | 0.00 | | 0.00 | | 0 | | | LV3296 | 0 | | 0 | | 0 | | 0 | | 0 | | 0 | | 0 | | | KD4099 | 0 | | 0 | | 0 | | 0 | | 0 | | 0 | | 0 | |  |  |
| 537 | F | | 310 | | 346 | | SP2510 | | 0 | | 0 | | 0 | | 0 | | 0.00 | | 0.00 | | 0 | | | LV3310 | 0 | | 0 | | 1 | | 0 | | 36.553 | | 0 | | 2.259817417 | | | KD4113 | 0 | | 0 | | 0 | | 0 | | 0 | | 38 | | 0 | |  |  |
| 538 | M | | 226 | | 126 | | SP2495 | | 0 | | 0 | | 0 | | 0 | | 0.00 | | 0.00 | | 0 | | | LV3295 | 0 | | 0 | | 1 | | 0 | | 37.623 | | 0 | | 1.090351888 | | | KD4098 | 0 | | 0 | | 0 | | 0 | | 38.79 | | 0 | | 0 | |  |  |
| 539 | F | | 310 | | 355 | | SP2517 | | 0 | | 0 | | 0 | | 0 | | 0.00 | | 0.00 | | 0 | | | LV3317 | 0 | | 0 | | 1 | | 1 | | 36.572 | | 37.609 | | 1.665780073 | | | KD4120 | 0 | | 0 | | 0 | |  | | 0 | |  | | 0 | |  |  |
| 540 | M | | 255 | | 143 | | SP2498 | | 0 | | 0 | | 0 | | 0 | | 0.00 | | 0.00 | | 0 | | | LV3298 | 0 | | 1 | | 0 | | 0 | | 0 | | 0 | | 0 | | | KD4101 | 0 | | 0 | | 0 | | 0 | | 0 | | 0 | | 0 | |  |  |
| 541 | F | | 374 | | 575 | | SP2512 | | 0 | | 0 | | 0 | | 0 | | 0.00 | | 0.00 | | 0 | | | LV3312 | 0 | | 0 | | 0 | | 1 | | 0 | | 37.633 | | 1.082950668 | | | KD4115 | 0 | | 0 | | 1 | | 1 | | 37.245 | | 37.049 | | 1.511243785 | |  |  |
| 542 | M | | 206 | | 77 | | SP2518 | | 0 | | 0 | | 0 | | 0 | | 0.00 | | 0.00 | | 0 | | | LV3318 | 0 | | 0 | | 0 | | 0 | | 37.925 | | 38.006 | | 0 | | | KD4121 | 0 | | 0 | | 0 | | 0 | | 0 | | 37.863 | | 0 | |  |  |
| 543 | F | | 363 | | 549 | | SP2520 | | 1 | | 0 | | 1 | | 1 | | 36.60 | | 36.276 | | 2.46256467 | | | LV3320 | 0 | | 0 | | 1 | | 0 | | 13.712 | | 0 | | 12896013.94 | | | KD4123 | 0 | | 0 | | 0 | | 0 | | 0 | | 0 | | 0 | |  |  |
| 544 | M | | 203 | | 76 | | SP2499 | | 0 | | 0 | | 0 | | 0 | | 0.00 | | 0.00 | | 0 | | | LV3299 | 0 | | 1 | | 0 | | 0 | | 38.384 | | 0 | | 0 | | | KD4102 | 0 | | 0 | | 0 | | 0 | | 0 | | 37.852 | | 0 | |  |  |
| 545 | F | | 365 | | 626 | | SP2515 | | 0 | | 0 | | 0 | | 0 | | 0.00 | | 0.00 | | 0 | | | LV3315 | 0 | | 0 | | 1 | | 1 | | 37.682 | | 36.609 | | 1.611325604 | | | KD4118 | 0 | | 0 | | 0 | | 1 | | 0 | | 37.525 | | 1.165615037 | |  |  |
| 546 | M | | 221 | | 114 | | SP2504 | | 0 | | 0 | | 0 | | 0 | | 0.00 | | 0.00 | | 0 | | | LV3304 | 0 | | 0 | | 1 | | 0 | | 37.142 | | 0 | | 1.513027361 | | | KD4107 | 0 | | 0 | | 0 | | 0 | | 0 | | 38.6 | | 0 | |  |  |
| 547 | F | | 326 | | 409 | | SP2522 | | 0 | | 0 | | 0 | | 0 | | 0.00 | | 0.00 | | 0 | | | LV3322 | 0 | | 0 | | 0 | | 0 | | 0 | | 39.198 | | 0 | | | KD4125 | 0 | | 0 | | 0 | | 0 | | 0 | | 0 | | 0 | |  |  |
| 548 | M | | 200 | | 68 | | SP2506 | | 0 | | 0 | | 0 | | 0 | | 38.84 | | 0.00 | | 0 | | | LV3306 | 0 | | 0 | | 1 | | 0 | | 37.254 | | 0 | | 1.401900244 | | | KD4109 | 0 | | 0 | | 1 | | 0 | | 33.883 | | 0 | | 13.92711072 | |  |  |
| 549 | F | | 330 | | 416 | | SP2524 | | 0 | | 0 | | 0 | | 0 | | 39.49 | | 0.00 | | 0 | | | LV3324 | 0 | | 0 | | 1 | | 0 | | 36.618 | | 0 | | 2.161953311 | | | KD4127 | 0 | | 0 | | 0 | | 0 | | 0 | | 0 | | 0 | |  |  |
| 550 | M | | 260 | | 187 | | SP2508 | | 0 | | 0 | | 0 | | 0 | | 0.00 | | 0.00 | | 0 | | | LV3308 | 0 | | 0 | | 0 | | 0 | | 0 | | 0 | | 0 | | | KD4111 | 0 | | 0 | | 0 | | 0 | | 0 | | 0 | | 0 | |  |  |
| 551 | F | | 380 | | 709 | | SP2523 | | 0 | | 0 | | 0 | | 0 | | 0.00 | | 0.00 | | 0 | | | LV3323 | 0 | | 0 | | 0 | | 0 | | 0 | | 0 | | 0 | | | KD4126 | 0 | | 0 | | 0 | | 0 | | 0 | | 0 | | 0 | |  |  |
| 552 | M | | 219 | | 86 | | SP2509 | | 0 | | 0 | | 0 | | 0 | | 0.00 | | 0.00 | | 0 | | | LV3309 | 0 | | 0 | | 0 | | 0 | | 38.647 | | 0 | | 0 | | | KD4112 | 0 | | 1 | | 0 | | 0 | | 0 | | 38.189 | | 0 | |  |  |
| 553 | F | | 311 | | 349 | | SP2525 | | 0 | | 0 | | 0 | | 0 | | 0.00 | | 0.00 | | 0 | | | LV3325 | 0 | | 0 | | 0 | | 0 | | 38 | | 0 | | 0 | | | KD4128 | 0 | | 0 | | 0 | | 0 | | 0 | | 0 | | 0 | |  |  |
| 554 | M | | 220 | | 109 | | SP2511 | | 0 | | 0 | | 0 | | 0 | | 0.00 | | 0.00 | | 0 | | | LV3311 | 0 | | 1 | | 1 | | 1 | | 36.411 | | 36.998 | | 2.079122023 | | | KD4114 | 0 | | 0 | | 0 | | 0 | | 0 | | 0 | | 0 | |  |  |
| 555 | F | | 355 | | 592 | | SP2521 | | 0 | | 0 | | 0 | | 0 | | 0.00 | | 0.00 | | 0 | | | LV3321 | 0 | | 0 | | 0 | | 0 | | 0 | | 0 | | 0 | | | KD4124 | 0 | | 0 | | 0 | | 0 | | 0 | | 0 | | 0 | |  |  |
| 556 | M | | 250 | | 152 | | SP2513 | | 0 | | 1 | | 1 | | 1 | | 30.82 | | 30.168 | | 143.7231983 | | | LV3313 | 0 | | 0 | | 1 | | 1 | | 35.666 | | 37.198 | | 2.795573583 | | | KD4116 | 0 | | 0 | | 1 | | 1 | | 36.842 | | 35.3 | | 3.580678947 | |  |  |
| 557 | F | | 315 | | 368 | | SP2526 | | 0 | | 0 | | 0 | | 0 | | 0.00 | | 0.00 | | 0 | | | LV3326 | 0 | | 0 | | 0 | | 1 | | 0 | | 37.119 | | 1.536916228 | | | KD4129 | 0 | | 0 | | 0 | | 0 | | 0 | | 0 | | 0 | |  |  |
| 558 | M | | 265 | | 165 | | SP2514 | | 0 | | 0 | | 1 | | 1 | | 36.36 | | 37.013 | | 2.119030291 | | | LV3314 | 1 | | 0 | | 0 | | 1 | | 0 | | 36.295 | | 2.693952374 | | | KD4117 | 0 | | 0 | | 1 | | 1 | | 36.521 | | 35.756 | | 3.099254403 | |  |  |
| 559 | F | | 310 | | 304 | | SP2527 | | 0 | | 0 | | 0 | | 0 | | 0.00 | | 0.00 | | 0 | | | LV3327 | 0 | | 0 | | 0 | | 0 | | 0 | | 0 | | 0 | | | KD4130 | 0 | | 0 | | 0 | | 0 | | 0 | | 0 | | 0 | |  |  |
| 560 | M | | 204 | | 84 | | SP2516 | | 1 | | 0 | | 0 | | 0 | | 38.89 | | 0.00 | | 0 | | | LV3316 | 0 | | 0 | | 0 | | 0 | | 0 | | 0 | | 0 | | | KD4119 | 0 | | 0 | | 1 | | 0 | | 36.648 | | 0 | | 2.118225988 | |  |  |
| 561 | F | | 330 | | 375 | | SP2529 | | 0 | | 0 | | 0 | | 0 | | 0.00 | | 0.00 | | 0 | | | LV3329 | 0 | | 0 | | 0 | | 0 | | 0 | | 39.293 | | 0 | | | KD4132 | 0 | | 0 | | 0 | | 0 | | 0 | | 0 | | 0 | |  |  |
| 562 | M | | 222 | | 160 | | SP2564 | | 0 | | 0 | | 1 | | 1 | | 37.21 | | 36.275 | | 2.087233228 | | | LV3364 | 0 | | 0 | | 0 | | 0 | | 0 | | 0 | | 0 | | | KD4168 | 0 | | 0 | | 0 | | 0 | | 0 | | 0 | | 0 | |  |  |
| 563 | F | | 313 | | 330 | | SP2531 | | 0 | | 0 | | 0 | | 0 | | 0.00 | | 0.00 | | 0 | | | LV3331 | 0 | | 0 | | 0 | | 0 | | 0 | | 0 | | 0 | | | KD4136 | 0 | | 0 | | 0 | | 0 | | 0 | | 0 | | 0 | |  |  |
| 564 | M | | 222 | | 269 | | SP2528 | | 0 | | 0 | | 0 | | 0 | | 0.00 | | 0.00 | | 0 | | | LV3328 | 0 | | 0 | | 1 | | 0 | | 37.475 | | 0 | | 1.205994071 | | | KD4131 | 0 | | 0 | | 0 | | 0 | | 0 | | 0 | | 0 | |  |  |
| 565 | F | | 290 | | 266 | | SP2533 | | 0 | | 0 | | 0 | | 0 | | 0.00 | | 0.00 | | 0 | | | LV3333 | 0 | | 0 | | 1 | | 0 | | 36.651 | | 0 | | 2.113902197 | | | KD4137 | 0 | | 0 | | 0 | | 0 | | 0 | | 0 | | 0 | |  |  |
| 566 | M | | 124 | | 109 | | SP2530 | | 0 | | 0 | | 0 | | 0 | | 0.00 | | 0.00 | | 0 | | | LV3330 | 0 | | 0 | | 0 | | 0 | | 0 | | 37.874 | | 0 | | | KD4133 | 0 | | 0 | | 0 | | 0 | | 0 | | 0 | | 0 | |  |  |
| 567 | F | | 331 | | 422 | | SP2536 | | 0 | | 0 | | 0 | | 0 | | 0.00 | | 0.00 | | 0 | | | LV3336 | 0 | | 1 | | 0 | | 0 | | 0 | | 0 | | 0 | | | KD4140 | 0 | | 0 | | 0 | | 0 | | 0 | | 0 | | 0 | |  |  |
| 568 | M | | 245 | | 152 | | SP2532 | | 0 | | 0 | | 0 | | 0 | | 0.00 | | 0.00 | | 0 | | | LV3332 | 0 | | 0 | | 0 | | 1 | | 0 | | 37.582 | | 1.121229474 | | | KD4135 | 0 | | 0 | | 0 | | 0 | | 0 | | 0 | | 0 | |  |  |
| 569 | F | | 360 | | 494 | | SP2539 | | 0 | | 0 | | 0 | | 0 | | 0.00 | | 0.00 | | 0 | | | LV3339 | 0 | | 0 | | 0 | | 0 | | 0 | | 0 | | 0 | | | KD4143 | 0 | | 0 | | 0 | | 0 | | 0 | | 0 | | 0 | |  |  |
| 570 | M | | 235 | | 125 | | SP2535 | | 0 | | 0 | | 0 | | 0 | | 0.00 | | 0.00 | | 0 | | | LV3335 | 0 | | 0 | | 0 | | 0 | | 39.442 | | 0 | | 0 | | | KD4139 | 0 | | 0 | | 0 | | 0 | | 0 | | 0 | | 0 | |  |  |
| 571 | F | | 328 | | 504 | | SP2543 | | 0 | | 0 | | 0 | | 0 | | 0.00 | | 0.00 | | 0 | | | LV3343 | 0 | | 0 | | 0 | | 0 | | 0 | | 0 | | 0 | | | KD4147 | 0 | | 0 | | 0 | | 0 | | 0 | | 0 | | 0 | |  |  |
| 572 | M | | 260 | | 185 | | SP2534 | | 0 | | 0 | | 0 | | 0 | | 0.00 | | 0.00 | | 0 | | | LV3334 | 0 | | 0 | | 1 | | 0 | | 34.679 | | 0 | | 8.098483997 | | | KD4138 | 0 | | 0 | | 0 | | 0 | | 0 | | 0 | | 0 | |  |  |
| 573 | F | | 350 | | 529 | | SP2541 | | 0 | | 0 | | 0 | | 0 | | 0.00 | | 0.00 | | 0 | | | LV3341 | 0 | | 0 | | 1 | | 0 | | 37.271 | | 0 | | 1.38576153 | | | KD4145 | 0 | | 0 | | 0 | | 0 | | 0 | | 0 | | 0 | |  |  |
| 574 | M | | 250 | | 170 | | SP2540 | | 0 | | 0 | | 0 | | 0 | | 0.00 | | 0.00 | | 0 | | | LV3340 | 0 | | 0 | | 1 | | 0 | | 37.654 | | 0 | | 1.067571227 | | | KD4144 | 0 | | 0 | | 0 | | 0 | | 0 | | 0 | | 0 | |  |  |
| 575 | F | | 335 | | 409 | | SP2545 | | 0 | | 0 | | 0 | | 0 | | 0.00 | | 0.00 | | 0 | | | LV3345 | 0 | | 0 | | 0 | | 0 | | 38.119 | | 0 | | 0 | | | KD4149 | 0 | | 0 | | 0 | | 0 | | 0 | | 0 | | 0 | |  |  |
| 576 | M | | 245 | | 154 | | SP2538 | | 0 | | 0 | | 0 | | 0 | | 0.00 | | 0.00 | | 0 | | | LV3338 | 0 | | 0 | | 0 | | 0 | | 0 | | 0 | | 0 | | | KD4142 | 0 | | 0 | | 0 | | 0 | | 0 | | 0 | | 0 | |  |  |
| 577 | F | | 315 | | 383 | | SP2548 | | 0 | | 0 | | 0 | | 0 | | 0.00 | | 0.00 | | 0 | | | LV3348 | 0 | | 0 | | 0 | | 0 | | 0 | | 0 | | 0 | | | KD4152 | 0 | | 0 | | 0 | | 0 | | 0 | | 0 | | 0 | |  |  |
| 578 | M | | 250 | | 166 | | SP2537 | | 0 | | 0 | | 0 | | 0 | | 0.00 | | 0.00 | | 0 | | | LV3337 | 0 | | 0 | | 1 | | 0 | | 35.248 | | 38.674 | | 5.496587818 | | | KD4141 | 0 | | 0 | | 0 | | 0 | | 0 | | 0 | | 0 | |  |  |
| 579 | F | | 350 | | 543 | | SP2551 | | 0 | | 0 | | 0 | | 0 | | 0.00 | | 0.00 | | 0 | | | LV3351 | 0 | | 0 | | 0 | | 0 | | 0 | | 0 | | 0 | | | KD4155 | 0 | | 0 | | 0 | | 0 | | 0 | | 0 | | 0 | |  |  |
| 580 | M | | 230 | | 130 | | SP2552 | | 0 | | 0 | | 0 | | 0 | | 0.00 | | 39.839 | | 0 | | |  |  | |  | |  | |  | |  | |  | |  | | | KD4156 | 0 | | 0 | | 0 | | 0 | | 0 | | 0 | | 0 | |  |  |
| 582 | M | | 285 | | 241 | | SP2544 | | 0 | | 0 | | 0 | | 0 | | 0.00 | | 0.00 | | 0 | | | LV3342 | 0 | | 0 | | 0 | | 0 | | 0 | | 0 | | 0 | | | KD4148 | 0 | | 0 | | 0 | | 0 | | 0 | | 0 | | 0 | |  |  |
| 583 | F | | 335 | | 470 | | SP2559 | | 0 | | 0 | | 0 | | 0 | | 0.00 | | 0.00 | | 0 | | | LV3359 | 1 | | 0 | | 0 | | 0 | | 0 | | 0 | | 0 | | | KD4163 | 0 | | 0 | | 0 | | 0 | | 0 | | 0 | | 0 | |  |  |
| 584 | M | | 220 | | 87 | | SP2542 | | 0 | | 0 | | 0 | | 0 | | 0.00 | | 0.00 | | 0 | | | LV3342 | 0 | | 0 | | 0 | | 0 | | 0 | | 0 | | 0 | | | KD4146 | 0 | | 0 | | 0 | | 0 | | 39.684 | | 0 | | 0 | |  |  |
| 585 | F | | 299 | | 354 | | SP2556 | | 0 | | 0 | | 0 | | 0 | | 0.00 | | 0.00 | | 0 | | | LV3356 | 0 | | 0 | | 0 | | 0 | | 0 | | 0 | | 0 | | | KD4161 | 0 | | 0 | | 0 | | 0 | | 0 | | 0 | | 0 | |  |  |
| 586 | M | | 250 | | 165 | | SP2546 | | 0 | | 0 | | 0 | | 0 | | 0.00 | | 0.00 | | 0 | | | LV3346 | 0 | | 0 | | 0 | | 0 | | 0 | | 0 | | 0 | | | KD4150 | 0 | | 0 | | 0 | | 0 | | 0 | | 0 | | 0 | |  |  |
| 587 | F | | 370 | | 675 | | SP2565 | | 0 | | 0 | | 0 | | 1 | | 0.00 | | 36.475 | | 2.383119392 | | | LV3365 | 0 | | 0 | | 0 | | 0 | | 0 | | 0 | | 0 | | | KD4169 | 0 | | 0 | | 0 | | 0 | | 0 | | 0 | | 0 | |  |  |
| 588 | M | | 210 | | 88 | | SP2547 | | 0 | | 0 | | 0 | | 0 | | 0.00 | | 0.00 | | 0 | | | LV3347 | 0 | | 0 | | 0 | | 0 | | 0 | | 0 | | 0 | | | KD4151 | 0 | | 0 | | 0 | | 0 | | 0 | | 0 | | 0 | |  |  |
| 589 | F | | 345 | | 519 | | SP2558 | | 0 | | 0 | | 0 | | 0 | | 0.00 | | 0.00 | | 0 | | | LV3358 | 0 | | 0 | | 0 | | 0 | | 0 | | 0 | | 0 | | | KD4162 | 0 | | 0 | | 0 | | 0 | | 0 | | 0 | | 0 | |  |  |
| 590 | M | | 270 | | 198 | | SP2567 | | 0 | | 0 | | 1 | | 0 | | 37.00 | | 0.00 | | 1.663271205 | | | LV3367 | 0 | | 0 | | 0 | | 0 | | 37.927 | | 0 | | 0 | | | KD4171 | 0 | | 0 | | 0 | | 0 | | 0 | | 0 | | 0 | |  |  |
| 591 | F | | 360 | | 644 | | SP2568 | | 0 | | 0 | | 0 | | 0 | | 0.00 | | 38.033 | | 0 | | | LV3368 | 0 | | 0 | | 0 | | 0 | | 0 | | 38.549 | | 0 | | | KD4172 | 0 | | 0 | | 0 | | 0 | | 0 | | 0 | | 0 | |  |  |
| 592 | M | | 249 | | 142 | | SP2549 | | 0 | | 0 | | 0 | | 0 | | 0.00 | | 0.00 | | 0 | | | LV3349 | 0 | | 0 | | 0 | | 0 | | 0 | | 0 | | 0 | | | KD4153 | 0 | | 0 | | 0 | | 0 | | 0 | | 0 | | 0 | |  |  |
| 593 | F | | 400 | | 785 | | SP2571 | | 0 | | 0 | | 0 | | 0 | | 0.00 | | 0.00 | | 0 | | | LV3371 | 0 | | 0 | | 0 | | 0 | | 0 | | 0 | | 0 | | | KD4175 | 0 | | 0 | | 0 | | 0 | | 0 | | 0 | | 0 | |  |  |
| 594 | M | | 230 | | 117 | | SP2550 | | 0 | | 0 | | 0 | | 0 | | 0.00 | | 0.00 | | 0 | | | LV3350 | 0 | | 0 | | 0 | | 0 | | 0 | | 0 | | 0 | | | KD4154 | 0 | | 0 | | 0 | | 0 | | 0 | | 0 | | 0 | |  |  |
| 595 | F | | 370 | | 655 | | SP2561 | | 0 | | 0 | | 0 | | 0 | | 0.00 | | 0.00 | | 0 | | | LV3361 | 0 | | 0 | | 0 | | 0 | | 39.774 | | 0 | | 0 | | | KD4165 | 0 | | 0 | | 0 | | 0 | | 0 | | 0 | | 0 | |  |  |
| 596 | M | | 270 | | 187 | | SP2566 | | 0 | | 0 | | 0 | | 0 | | 0.00 | | 0.00 | | 0 | | | LV3366 | 0 | | 0 | |  | |  | |  | |  | |  | | | KD4170 | 0 | | 0 | | 0 | | 0 | | 0 | | 0 | | 0 | |  |  |
| 597 | F | | 375 | | 652 | | SP2563 | | 0 | | 0 | | 0 | | 0 | | 0.00 | | 0.00 | | 0 | | | LV3363 | 0 | | 1 | | 0 | | 0 | | 0 | | 0 | | 0 | | | KD4167 | 0 | | 0 | | 0 | | 0 | | 0 | | 0 | | 0 | |  |  |
| 598 | M | | 256 | | 161 | | SP2555 | | 0 | | 0 | | 0 | | 0 | | 0.00 | | 0.00 | | 0 | | | LV3355 | 0 | | 0 | | 0 | | 0 | | 0 | | 0 | | 0 | | | KD4159 | 0 | | 0 | | 0 | | 0 | | 0 | | 0 | | 0 | |  |  |
| 599 | F | | 335 | | 525 | | SP2573 | | 0 | | 0 | | 0 | | 1 | | 0.00 | | 37.592 | | 1.113618659 | | | LV3373 | 0 | | 0 | |  | | 0 | |  | | 0 | | 0 | | | KD4177 | 0 | | 0 | | 0 | | 0 | | 0 | | 0 | | 0 | |  |  |
| 600 | M | | 245 | | 163 | | SP2554 | | 0 | | 0 | | 0 | | 0 | | 0.00 | | 0.00 | | 0 | | | LV3354 | 0 | | 0 | | 0 | | 0 | | 0 | | 0 | | 0 | | | KD4158 | 0 | | 0 | | 0 | | 0 | | 0 | | 0 | | 0 | |  |  |
| 601 | F | | 320 | | 383 | | SP2579 | | 0 | | 0 | | 0 | | 0 | | 0.00 | | 0.00 | | 0 | | | LV3379 | 0 | | 0 | | 0 | | 0 | | 37.787 | | 0 | | 0 | | | KD4183 | 0 | | 0 | | 0 | | 0 | | 0 | | 0 | | 0 | |  |  |
| 602 | M | | 236 | | 128 | | SP2557 | | 0 | | 0 | | 0 | | 0 | | 0.00 | | 0.00 | | 0 | | | LV3357 | 0 | | 1 | | 0 | | 0 | | 0 | | 0 | | 0 | | | KD4160 | 0 | | 0 | | 0 | | 0 | | 0 | | 0 | | 0 | |  |  |
| 603 | F | | 260 | | 375 | | SP2576 | | 0 | | 0 | | 0 | | 0 | | 0.00 | | 0.00 | | 0 | | | LV3376 | 0 | | 0 | | 0 | | 0 | | 0 | | 0 | | 0 | | | KD4180 | 0 | | 0 | | 0 | | 0 | | 0 | | 0 | | 0 | |  |  |
| 604 | M | | 263 | | 158 | |  | |  | |  | |  | |  | |  | |  | |  | | | LV3375 | 0 | | 0 | | 0 | | 0 | | 0 | | 0 | | 0 | | | KD4179 | 0 | | 0 | | 0 | | 0 | | 0 | | 0 | | 0 | |  |  |
| 605 | F | | 348 | | 527 | | SP2581 | | 0 | | 0 | | 0 | | 0 | | 38.35 | | 0.00 | | 0 | | | LV3381 | 0 | | 0 | | 0 | | 0 | | 0 | | 0 | | 0 | | | KD4185 | 0 | | 0 | | 0 | | 0 | | 0 | | 0 | | 0 | |  |  |
| 606 | M | | 240 | | 134 | | SP2560 | | 0 | | 0 | | 0 | | 0 | | 0.00 | | 0.00 | | 0 | | | LV3360 | 0 | | 0 | | 0 | | 0 | | 0 | | 0 | | 0 | | | KD4164 | 0 | | 0 | | 0 | | 0 | | 0 | | 0 | | 0 | |  |  |
| 607 | F | | 324 | | 415 | | SP2584 | | 0 | | 0 | |  | |  | |  | |  | |  | | |  |  | |  | |  | |  | |  | |  | |  | | | KD4188 | 0 | | 0 | | 0 | | 0 | | 0 | | 0 | | 0 | |  |  |
| 608 | M | | 220 | | 106 | | SP2572 | | 0 | | 0 | | 0 | | 0 | | 0.00 | | 0.00 | | 0 | | | LV3372 | 0 | | 0 | | 0 | | 0 | | 0 | | 0 | | 0 | | | KD4176 | 0 | | 0 | | 0 | | 0 | | 0 | | 0 | | 0 | |  |  |
| 609 | F | | 246 | | 516 | | SP2585 | | 0 | | 0 | | 0 | | 0 | | 0.00 | | 0.00 | | 0 | | | LV3384 | 0 | | 1 | | 1 | | 0 | | 34.964 | | 0 | | 6.669611685 | | | KD4189 | 0 | | 0 | | 0 | | 0 | | 0 | | 0 | | 0 | |  |  |
| 610 | M | | 260 | | 179 | | SP2562 | | 0 | | 0 | | 0 | | 0 | | 0.00 | | 0.00 | | 0 | | | LV3362 | 1 | | 0 | | 0 | | 0 | | 0 | | 0 | | 0 | | | KD4166 | 0 | | 0 | | 0 | | 0 | | 0 | | 0 | | 0 | |  |  |
| 611 | F | | 355 | | 570 | | SP2586 | | 0 | | 0 | | 0 | | 0 | | 0.00 | | 0.00 | | 0 | | | LV3385 | 0 | | 1 | | 0 | | 0 | | 0 | | 0 | | 0 | | | KD4190 | 0 | | 0 | | 0 | | 0 | | 0 | | 0 | | 0 | |  |  |
| 612 | M | | 235 | | 113 | | SP2577 | | 0 | | 0 | | 0 | | 0 | | 0.00 | | 0.00 | | 0 | | | LV3377 | 0 | | 0 | | 0 | | 0 | | 0 | | 0 | | 0 | | | KD4181 | 0 | | 0 | | 0 | | 0 | | 0 | | 0 | | 0 | |  |  |
| 613 | F | | 340 | | 502 | | SP2582 | | 0 | | 0 | | 0 | | 0 | | 0.00 | | 0.00 | | 0 | | | LV3380 | 1 | | 0 | | 0 | | 0 | | 0 | | 0 | | 0 | | | KD4186 | 0 | | 0 | | 0 | | 0 | | 0 | | 0 | | 0 | |  |  |
| 614 | M | | 225 | | 111 | | SP2569 | | 0 | | 0 | | 0 | | 1 | | 38.00 | | 37.272 | | 1.384818001 | | | LV3369 | 0 | | 0 | | 0 | | 0 | | 0 | | 0 | | 0 | | | KD4173 | 0 | | 0 | | 0 | | 0 | | 0 | | 0 | | 0 | |  |  |
| 615 | F | | 293 | | 290 | | SP2580 | | 0 | | 0 | | 0 | | 0 | | 0.00 | | 0.00 | | 0 | | | LV3378 | 0 | | 0 | | 0 | | 0 | | 0 | | 0 | | 0 | | | KD4184 | 0 | | 0 | | 0 | | 0 | | 0 | | 0 | | 0 | |  |  |
| 616 | M | | 240 | | 139 | | SP2574 | | 0 | | 0 | | 0 | | 0 | | 0.00 | | 0.00 | | 0 | | | LV3374 | 0 | | 0 | | 0 | | 1 | | 39.252 | | 36.991 | | 1.676921283 | | | KD4178 | 0 | | 0 | | 0 | | 0 | | 0 | | 0 | | 0 | |  |  |
| 617 | F | | 340 | | 546 | | SP2583 | | 0 | | 0 | | 0 | | 0 | | 0.00 | | 0.00 | | 0 | | | LV3382 | 1 | | 0 | | 0 | | 0 | | 38.301 | | 38.647 | | 0 | | | KD4187 | 0 | | 0 | | 0 | | 0 | | 0 | | 0 | | 0 | |  |  |
| 618 | M | | 255 | | 155 | | SP2578 | | 0 | | 0 | | 0 | | 0 | | 38.82 | | 0.00 | | 0 | | |  |  | |  | |  | |  | |  | |  | |  | | | KD4182 | 0 | | 0 | | 0 | | 0 | | 0 | | 0 | | 0 | |  |  |
| 619 | F | | 280 | | 291 | | SP2587 | | 0 | | 0 | | 0 | | 0 | | 0.00 | | 0.00 | | 0 | | | LV3386 | 0 | | 0 | | 0 | | 0 | | 0 | | 0 | | 0 | | | KD4191 | 0 | | 0 | | 0 | | 0 | | 38.73 | | 0 | | 0 | |  |  |
| 620 | M | | 248 | | 140 | | SP2570 | | 0 | | 0 | | 0 | | 0 | | 0.00 | | 0.00 | | 0 | | | LV3370 | 1 | | 0 | | 0 | | 0 | | 0 | | 0 | | 0 | | | KD4174 | 0 | | 0 | | 0 | | 0 | | 0 | | 0 | | 0 | |  |  |
| 621 | F | | 357 | | 569 | | SP2489 | | 0 | | 0 | | 0 | | 0 | | 0.00 | | 0.00 | | 0 | | | LV3289 | 1 | | 1 | | 0 | | 0 | | 0 | | 0 | | 0 | | | KD4092 | 0 | | 0 | | 0 | | 0 | | 0 | | 0 | | 0 | |  |  |
| 622 | M | | 246 | | 137 | | SP2588 | | 0 | | 0 | | 0 | | 0 | | 0.00 | | 0.00 | | 0 | | | LV3387 | 0 | | 0 | | 0 | | 0 | | 0 | | 0 | | 0 | | | KD4192 | 0 | | 0 | | 0 | | 0 | | 39.277 | | 39.452 | | 0 | |  |  |
| 623 | F | | 373 | | 614 | | SP2590 | | 0 | | 0 | | 0 | | 0 | | 0.00 | | 0.00 | | 0 | | | LV3389 | 0 | | 0 | | 0 | | 0 | | 0 | | 0 | | 0 | | | KD4195 | 0 | | 0 | | 0 | | 0 | | 0 | | 39.37 | | 0 | |  |  |
| 624 | M | | 215 | | 99 | | SP2589 | | 0 | | 0 | | 0 | | 0 | | 0.00 | | 0.00 | | 0 | | | LV3388 | 0 | | 0 | | 0 | | 0 | | 39.094 | | 0 | | 0 | | | KD4193 | 0 | | 0 | | 0 | | 0 | | 0 | | 0 | | 0 | |  |  |
| 625 | F | | 335 | | 527 | | SP2593 | | 0 | | 0 | | 0 | | 0 | | 0.00 | | 0.00 | | 0 | | | LV3392 | 0 | | 0 | | 0 | | 0 | | 0 | | 0 | | 0 | | | KD4197 | 0 | | 0 | | 0 | | 0 | | 0 | | 0 | | 0 | |  |  |
| 626 | M | | 236 | | 136 | | SP2591 | | 0 | | 0 | | 0 | | 0 | | 0.00 | | 0.00 | | 0 | | | LV3390 | 0 | | 0 | | 0 | | 0 | | 38.923 | | 0 | | 0 | | | KD4194 | 0 | | 0 | | 0 | | 0 | | 0 | | 0 | | 0 | |  |  |
| 627 | F | | 333 | | 402 | | SP2594 | | 0 | | 0 | | 0 | | 0 | | 0.00 | | 0.00 | | 0 | | | LV3393 | 0 | | 0 | | 0 | | 0 | | 0 | | 0 | | 0 | | | KD4198 | 0 | | 0 | | 0 | | 0 | | 0 | | 0 | | 0 | |  |  |
| 628 | M | | 193 | | 83 | | SP2599 | | 0 | | 0 | | 0 | | 0 | | 0.00 | | 0.00 | | 0 | | | LV3398 | 0 | | 0 | | 0 | | 0 | | 0 | | 0 | | 0 | | | KD4203 | 0 | | 0 | | 1 | | 1 | | 35.627 | | 36.966 | | 2.97588309 | |  |  |
| 629 | F | | 346 | | 516 | | SP2597 | | 0 | | 0 | | 0 | | 0 | | 0.00 | | 0.00 | | 0 | | | LV3396 | 0 | | 0 | | 0 | | 0 | | 0 | | 0 | | 0 | | | KD4201 | 0 | | 0 | | 1 | | 0 | | 36.658 | | 0 | | 2.103847643 | |  |  |
| 630 | M | | 285 | | 227 | | SP2592 | | 0 | | 0 | | 0 | | 0 | | 0.00 | | 0.00 | | 0 | | | LV3391 | 0 | | 0 | | 0 | | 0 | | 0 | | 0 | | 0 | | | KD4196 | 0 | | 0 | | 0 | | 0 | | 0 | | 0 | | 0 | |  |  |
| 631 | F | | 353 | | 565 | | SP2601 | | 0 | | 0 | | 0 | | 0 | | 0.00 | | 0.00 | | 0 | | | LV3400 | 0 | | 0 | | 0 | | 0 | | 38 | | 0 | | 0 | | | KD4205 | 0 | | 0 | | 1 | | 0 | | 36.442 | | 0 | | 2.437290082 | |  |  |
| 632 | M | | 217 | | 102 | | SP2595 | | 0 | | 0 | | 0 | | 0 | | 0.00 | | 0.00 | | 0 | | | LV3394 | 0 | | 0 | | 0 | | 0 | | 0 | | 0 | | 0 | | | KD4199 | 0 | | 0 | | 0 | | 0 | | 0 | | 0 | | 0 | |  |  |
| 633 | F | | 330 | | 412 | | SP2602 | | 0 | | 0 | | 0 | | 0 | | 0.00 | | 0.00 | | 0 | | | LV3402 | 0 | | 1 | | 0 | | 0 | | 0 | | 0 | | 0 | | | KD4207 | 0 | | 0 | | 0 | | 0 | | 0 | | 0 | | 0 | |  |  |
| 634 | M | | 270 | | 205 | | SP2607 | | 0 | | 0 | | 0 | | 0 | | 0.00 | | 0.00 | | 0 | | | LV3406 | 0 | | 0 | | 0 | | 0 | | 0 | | 0 | | 0 | | | KD4211 | 0 | | 0 | | 0 | | 0 | | 0 | | 0 | | 0 | |  |  |
| 635 | F | | 310 | | 354 | | SP2606 | | 0 | | 0 | | 0 | | 0 | | 0.00 | | 0.00 | | 0 | | | LV3405 | 0 | | 0 | | 0 | | 0 | | 0 | | 0 | | 0 | | | KD4210 | 0 | | 0 | | 1 | | 0 | | 37.429 | | 38.707 | | 1.244377075 | |  |  |
| 636 | M | | 236 | | 131 | | SP2596 | | 0 | | 0 | | 0 | | 0 | | 0.00 | | 0.00 | | 0 | | | LV3395 | 1 | | 1 | | 0 | | 0 | | 0 | | 0 | | 0 | | | KD4200 | 0 | | 0 | | 0 | | 0 | | 0 | | 0 | | 0 | |  |  |
| 637 | F | | 343 | | 467 | | SP2609 | | 0 | | 0 | | 0 | | 0 | | 0.00 | | 0.00 | | 0 | | | LV3408 | 0 | | 0 | | 0 | | 0 | | 0 | | 0 | | 0 | | | KD4213 | 0 | | 0 | | 0 | | 0 | | 0 | | 0 | | 0 | |  |  |
| 638 | M | | 230 | | 133 | | SP2598 | | 0 | | 0 | | 0 | | 0 | | 0.00 | | 0.00 | | 0 | | | LV3397 | 0 | | 0 | | 0 | | 0 | | 0 | | 0 | | 0 | | | KD4202 | 0 | | 0 | | 0 | | 1 | | 0 | | 35.018 | | 6.428761518 | |  |  |
| 639 | F | | 315 | | 331 | | SP2611 | | 0 | | 0 | | 0 | | 0 | | 0.00 | | 0.00 | | 0 | | | LV3410 | 0 | | 0 | | 0 | | 0 | | 38.761 | | 0 | | 0 | | | KD4215 | 0 | | 0 | | 0 | | 0 | | 0 | | 0 | | 0 | |  |  |
| 640 | M | | 295 | | 258 | | SP2603 | | 0 | | 0 | | 0 | | 0 | | 0.00 | | 0.00 | | 0 | | | LV3401 | 0 | | 0 | | 0 | | 0 | | 0 | | 0 | | 0 | | | KD4206 | 0 | | 0 | | 0 | | 0 | | 0 | | 38.353 | | 0 | |  |  |
| 641 | F | | 339 | | 495 | | SP2613 | | 0 | | 0 | | 0 | | 0 | | 0.00 | | 0.00 | | 0 | | | LV3412 | 0 | | 0 | | 0 | | 0 | | 0 | | 38.051 | | 0 | | | KD4217 | 0 | | 0 | | 0 | | 1 | | 0 | | 36.977 | | 1.692988022 | |  |  |
| 642 | M | | 221 | | 113 | | SP2617 | | 0 | | 0 | | 0 | | 0 | | 0.00 | | 0.00 | | 0 | | | LV3416 | 0 | | 0 | | 0 | | 0 | | 0 | | 0 | | 0 | | | KD4221 | 0 | | 0 | | 0 | | 0 | | 0 | | 0 | | 0 | |  |  |
| 643 | F | | 390 | | 740 | | SP2615 | | 0 | | 0 | | 0 | | 0 | | 0.00 | | 0.00 | | 0 | | | LV3414 | 0 | | 0 | | 0 | | 0 | | 0 | | 0 | | 0 | | | KD4219 | 0 | | 0 | | 0 | | 0 | | 0 | | 0 | | 0 | |  |  |
| 644 | M | | 225 | | 108 | | SP2600 | | 0 | | 0 | | 0 | | 0 | | 0.00 | | 0.00 | | 0 | | | LV3399 | 0 | | 0 | | 0 | | 0 | | 39.125 | | 39.231 | | 0 | | | KD4204 | 0 | | 0 | | 1 | | 1 | | 34.784 | | 34.507 | | 8.322283954 | |  |  |
| 645 | F | | 350 | | 482 | | SP2614 | | 0 | | 0 | | 0 | | 0 | | 0.00 | | 0.00 | | 0 | | | LV3413 | 0 | | 0 | | 0 | | 0 | | 0 | | 0 | | 0 | | | KD4218 | 0 | | 0 | | 0 | | 0 | | 0 | | 0 | | 0 | |  |  |
| 646 | M | | 216 | | 114 | | SP2604 | | 0 | | 0 | | 0 | | 0 | | 0.00 | | 0.00 | | 0 | | | LV3403 | 0 | | 0 | | 0 | | 0 | | 0 | | 0 | | 0 | | | KD4208 | 0 | | 0 | | 1 | | 1 | | 36.407 | | 35.055 | | 4.38243258 | |  |  |
| 647 | F | | 357 | | 528 | | SP2618 | | 0 | | 0 | | 0 | | 0 | | 0.00 | | 0.00 | | 0 | | | LV3417 | 0 | | 0 | | 0 | | 0 | | 0 | | 38.915 | | 0 | | | KD4222 | 0 | | 0 | | 0 | | 0 | | 0 | | 0 | | 0 | |  |  |
| 648 | M | | 232 | | 130 | | SP2612 | | 0 | | 0 | | 0 | | 0 | | 0.00 | | 0.00 | | 0 | | | LV3411 | 0 | | 0 | | 0 | | 0 | | 0 | | 0 | | 0 | | | KD4216 | 0 | | 0 | | 0 | | 0 | | 0 | | 0 | | 0 | |  |  |
| 649 | F | | 335 | | 425 | | SP2619 | | 0 | | 0 | | 0 | | 0 | | 0.00 | | 0.00 | | 0 | | | LV3418 | 0 | | 0 | | 0 | | 0 | | 0 | | 0 | | 0 | | | KD4223 | 0 | | 0 | | 0 | | 0 | | 0 | | 0 | | 0 | |  |  |
| 650 | M | | 235 | | 133 | | SP2605 | | 0 | | 0 | | 0 | | 0 | | 0.00 | | 0.00 | | 0 | | | LV3404 | 0 | | 1 | | 0 | | 0 | | 0 | | 0 | | 0 | | | KD4209 | 0 | | 0 | | 0 | | 1 | | 38 | | 37.039 | | 1.62298403 | |  |  |
| 651 | F | | 371 | | 660 | | SP2616 | | 0 | | 0 | | 0 | | 0 | | 0.00 | | 0.00 | | 0 | | | LV3415 | 0 | | 0 | | 0 | | 0 | | 38 | | 37.972 | | 0 | | | KD4220 | 0 | | 0 | | 0 | | 0 | | 38.697 | | 0 | | 0 | |  |  |
| 652 | M | | 274 | | 190 | | SP2608 | | 0 | | 0 | | 0 | | 0 | | 0.00 | | 0.00 | | 0 | | | LV3407 | 0 | | 0 | | 0 | | 0 | | 0 | | 0 | | 0 | | | KD4212 | 0 | | 0 | | 0 | | 0 | | 0 | | 0 | | 0 | |  |  |
| 653 | F | | 210 | | 157 | | SP2620 | | 0 | | 0 | | 0 | | 0 | | 0.00 | | 0.00 | | 0 | | | LV3419 | 1 | | 0 | | 0 | | 0 | | 0 | | 39.112 | | 0 | | | KD4224 | 0 | | 0 | | 0 | | 0 | | 0 | | 0 | | 0 | |  |  |
| 654 | M | | 236 | | 136 | | SP2610 | | 0 | | 0 | | 0 | | 0 | | 0.00 | | 0.00 | | 0 | | | LV3409 | 0 | | 0 | | 0 | | 0 | | 0 | | 0 | | 0 | | | KD4214 | 0 | | 0 | | 0 | | 0 | | 0 | | 38.37 | | 0 | |  |  |
| 655 | F | | 340 | | 443 | | SP2621 | | 0 | | 0 | | 0 | | 0 | | 0.00 | | 0.00 | | 0 | | | LV3420 | 0 | | 0 | | 0 | | 1 | | 0 | | 36.279 | | 2.723470802 | | | KD4225 | 0 | | 0 | | 1 | | 1 | | 35.991 | | 34.793 | | 5.403580992 | |  |  |
| 656 | M | | 230 | | 139 | | SP2624 | | 0 | | 0 | | 0 | | 0 | | 0.00 | | 0.00 | | 0 | | | LV3423 | 0 | | 0 | | 1 | | 1 | | 36.709 | | 32.75 | | 16.08115014 | | | KD4228 | 0 | | 0 | | 1 | | 1 | | 35.851 | | 36.17 | | 3.289295655 | |  |  |
| 657 | F | | 315 | | 416 | | SP2623 | | 0 | | 0 | | 0 | | 0 | | 0.00 | | 0.00 | | 0 | | | LV3422 | 0 | | 0 | | 1 | | 0 | | 35.904 | | 0 | | 3.515992655 | | | KD4227 | 0 | | 0 | | 1 | | 1 | | 33.944 | | 33.985 | | 13.17636479 | |  |  |
| 658 | M | | 240 | | 137 | | SP2622 | | 0 | | 0 | | 0 | | 0 | | 0.00 | | 39.060 | | 0 | | | LV3421 | 0 | | 0 | | 1 | | 0 | | 37.465 | | 37.812 | | 1.214236207 | | | KD4226 | 0 | | 0 | | 1 | | 1 | | 33.875 | | 35.399 | | 9.481291653 | |  |  |
| 659 | F | | 355 | | 445 | | SP2626 | | 0 | | 0 | | 0 | | 0 | | 0.00 | | 0.00 | | 0 | | | LV3424 | 0 | | 0 | | 1 | | 1 | | 37.633 | | 36.908 | | 1.428701074 | | | KD4229 | 0 | | 0 | | 1 | | 1 | | 35.734 | | 34.853 | | 5.570505933 | |  |  |
| 660 | M | | 195 | | 78 | | SP2625 | | 0 | | 0 | | 0 | | 0 | | 0.00 | | 0.00 | | 0 | | | LV3425 | 0 | | 0 | | 1 | | 1 | | 35.838 | | 35.489 | | 4.171077373 | | | KD4230 | 0 | | 0 | | 1 | | 1 | | 34.758 | | 35.498 | | 6.155118693 | |  |  |
| 661 | F | | 316 | | 375 | | SP2628 | | 0 | | 0 | | 1 | | 1 | | 37.56 | | 37.012 | | 1.394857058 | | | LV3427 | 0 | | 0 | | 0 | | 0 | | 0 | | 37.889 | | 0 | | | KD4232 | 0 | | 0 | | 1 | | 1 | | 36.914 | | 34.67 | | 4.957747273 | |  |  |
| 662 | M | | 204 | | 92 | | SP2632 | | 0 | | 0 | | 0 | | 0 | | 0.00 | | 0.00 | | 0 | | | LV3432 | 1 | | 1 | | 1 | | 1 | | 36.52 | | 34.861 | | 4.732749804 | | | KD4237 | 0 | | 0 | | 1 | | 1 | | 35.208 | | 33.581 | | 11.37807186 | |  |  |
| 663 | F | | 320 | | 446 | | SP2630 | | 1 | | 1 | | 1 | | 1 | | 18.42 | | 20.648 | | 318162.726 | | | LV3429 | 1 | | 1 | | 1 | | 0 | | 18.986 | | 0 | | 355142.7736 | | | KD4234 | 1 | | 1 | | 1 | | 1 | | 19.163 | | 19.673 | | 268617.7995 | |  |  |
| 664 | M | | 255 | | 164 | | SP2627 | | 0 | | 0 | | 0 | | 0 | | 39.76 | | 38.501 | | 0 | | | LV3426 | 0 | | 0 | | 1 | | 1 | | 37.273 | | 37.138 | | 1.450515112 | | | KD4231 | 0 | | 0 | | 1 | | 1 | | 35.976 | | 34.289 | | 6.95510547 | |  |  |
| 665 | F | | 359 | | 552 | | SP2633 | | 0 | | 0 | | 0 | | 0 | | 0.00 | | 0.00 | | 0 | | | LV3431 | 1 | | 1 | | 1 | | 1 | | 33.384 | | 34.823 | | 13.45312743 | | | KD4236 | 1 | | 1 | | 1 | | 1 | | 33.806 | | 34.267 | | 12.69947911 | |  |  |
| 666 | M | | 224 | | 116 | | SP2644 | | 0 | | 0 | | 0 | | 0 | | 0.00 | | 0.00 | | 0 | | | LV3444 | 0 | | 0 | | 1 | | 0 | | 37.666 | | 0 | | 1.058881237 | | | KD4249 | 0 | | 0 | | 1 | | 1 | | 35.03 | | 33.067 | | 15.32781492 | |  |  |
| 667 | F | | 278 | | 222 | | SP2640 | | 0 | | 0 | | 0 | | 0 | | 0.00 | | 0.00 | | 0 | | | LV3439 | 0 | | 1 | | 1 | | 1 | | 35.246 | | 36.191 | | 4.197890033 | | | KD4244 | 0 | | 0 | | 1 | | 1 | | 35.399 | | 31.642 | | 34.52173538 | |  |  |
| 668 | M | | 225 | | 115 | | SP2629 | | 0 | | 0 | | 0 | | 0 | | 0.00 | | 0.00 | | 0 | | | LV3428 | 0 | | 0 | | 0 | | 1 | | 39.456 | | 36.918 | | 1.762406648 | | | KD4233 | 0 | | 0 | | 1 | | 1 | | 33.289 | | 32.817 | | 24.82916619 | |  |  |
| 669 | F | | 331 | | 382 | | SP2635 | | 0 | | 0 | | 0 | | 0 | | 0.00 | | 0.00 | | 0 | | | LV3434 | 0 | | 0 | | 1 | | 1 | | 36.262 | | 33.424 | | 10.89686158 | | | KD4239 | 0 | | 0 | | 1 | | 1 | | 32.948 | | 33.074 | | 25.24636597 | |  |  |
| 670 | M | | 215 | | 93 | | SP2641 | | 0 | | 0 | | 0 | | 0 | | 0.00 | | 0.00 | | 0 | | | LV3440 | 1 | | 1 | | 1 | | 0 | | 36.088 | | 37.813 | | 3.101848938 | | | KD4245 |  | |  | |  | |  | |  | |  | |  | |  |  |
| 671 | F | | 348 | | 475 | | SP2638 | | 0 | | 0 | | 0 | | 0 | | 0.00 | | 0.00 | | 0 | | | LV3437 | 0 | | 0 | | 1 | | 1 | | 33.868 | | 36.528 | | 8.184376964 | | | KD4242 | 0 | | 0 | | 1 | | 1 | | 35.245 | | 35.535 | | 5.014227313 | |  |  |
| 672 | M | | 210 | | 97 | | SP2631 | | 0 | | 0 | | 0 | | 0 | | 0.00 | | 39.091 | | 0 | | | LV3430 | 1 | | 1 | | 1 | | 1 | | 36.426 | | 33.669 | | 9.28823756 | | | KD4235 | 1 | | 1 | | 1 | | 1 | | 36.567 | | 35.43 | | 3.547067048 | |  |  |
| 673 | F | | 326 | | 414 | | SP2642 | | 0 | | 0 | | 0 | | 0 | | 0.00 | | 0.00 | | 0 | | | LV3441 | 0 | | 1 | | 0 | | 0 | | 0 | | 37.927 | | 0 | | | KD4246 | 0 | | 0 | | 1 | | 1 | | 33.596 | | 33.271 | | 19.03172055 | |  |  |
| 674 | M | | 245 | | 148 | | SP2637 | | 0 | | 0 | | 0 | | 0 | | 0.00 | | 0.00 | | 0 | | | LV3436 | 0 | | 0 | | 1 | | 1 | | 37.29 | | 36.829 | | 1.620244701 | | | KD4241 | 0 | | 0 | | 1 | | 1 | | 33.478 | | 33.902 | | 16.04953202 | |  |  |
| 675 | F | | 322 | | 394 | | SP2645 | | 0 | | 0 | | 0 | | 0 | | 0.00 | | 0.00 | | 0 | | | LV3443 | 1 | | 0 | | 0 | | 0 | | 0 | | 0 | | 0 | | | KD4248 | 0 | | 0 | | 1 | | 1 | | 34.067 | | 34.136 | | 12.00462175 | |  |  |
| 676 | M | | 256 | | 150 | | SP2634 | | 0 | | 0 | | 0 | | 0 | | 0.00 | | 0.00 | | 0 | | | LV3433 | 0 | | 0 | | 0 | | 1 | | 0 | | 35.229 | | 5.568181499 | | | KD4238 | 0 | | 0 | | 1 | | 1 | | 34.034 | | 34.189 | | 11.93645454 | |  |  |
| 677 | F | | 277 | | 225 | | SP2650 | | 0 | | 0 | | 0 | | 0 | | 0.00 | | 0.00 | | 0 | | | LV3449 | 0 | | 0 | | 0 | | 0 | | 0 | | 37.877 | | 0 | | | KD4254 | 0 | | 0 | | 1 | | 1 | | 33.826 | | 33.238 | | 18.04416016 | |  |  |
| 678 | M | | 277 | | 210 | | SP2636 | | 0 | | 0 | | 0 | | 0 | | 0.00 | | 0.00 | | 0 | | | LV3435 | 0 | | 0 | | 0 | | 1 | | 38.187 | | 36.478 | | 2.378254892 | | | KD4240 | 0 | | 0 | | 1 | | 1 | | 35.213 | | 32.225 | | 24.35572645 | |  |  |
| 679 | F | | 287 | | 272 | | SP2647 | | 0 | | 0 | | 0 | | 0 | | 0.00 | | 0.00 | | 0 | | | LV3446 | 0 | | 0 | | 1 | | 1 | | 35.887 | | 36.326 | | 3.097304014 | | | KD4251 | 0 | | 0 | | 1 | | 1 | | 32.863 | | 34.678 | | 18.00114861 | |  |  |
| 680 | M | | 259 | | 120 | | SP2639 | | 0 | | 0 | | 0 | | 0 | | 0.00 | | 0.00 | | 0 | | | LV3438 | 0 | | 0 | | 1 | | 1 | | 34.969 | | 37.239 | | 4.031616526 | | | KD4243 | 0 | | 0 | | 1 | | 1 | | 35.194 | | 35.59 | | 5.028462483 | |  |  |
| 681 | F | | 372 | | 627 | | SP2655 | | 0 | | 0 | | 0 | | 0 | | 0.00 | | 0.00 | | 0 | | | LV3454 | 0 | | 0 | | 1 | | 0 | | 36.757 | | 0 | | 1.966663346 | | | KD4259 | 0 | | 0 | | 1 | | 1 | | 32.957 | | 33.113 | | 24.84920788 | |  |  |
| 682 | M | | 247 | | 166 | | SP2643 | | 0 | | 0 | | 0 | | 0 | | 0.00 | | 0.00 | | 0 | | | LV3442 | 0 | | 1 | | 1 | | 0 | | 36.877 | | 39.725 | | 1.812316098 | | | KD4247 | 0 | | 0 | | 1 | | 1 | | 33.526 | | 33.663 | | 16.96961602 | |  |  |
| 683 | F | | 328 | | 461 | |  | |  | |  | |  | |  | |  | |  | |  | | | LV3456 | 0 | | 0 | | 0 | | 1 | | 0 | | 37.263 | | 1.393332944 | | | KD4261 | 0 | | 0 | | 1 | | 1 | | 34.373 | | 33.628 | | 13.27195025 | |  |  |
| 684 | M | | 270 | | 178 | | SP2654 | | 0 | | 0 | | 0 | | 0 | | 0.00 | | 0.00 | | 0 | | | LV3453 | 0 | | 0 | | 1 | | 1 | | 37.552 | | 36.679 | | 1.60917272 | | | KD4258 | 0 | | 0 | | 1 | | 1 | | 34.717 | | 35.804 | | 5.827690443 | |  |  |
| 685 | F | | 276 | | 302 | | SP2659 | | 0 | | 0 | | 0 | | 0 | | 0.00 | | 0.00 | | 0 | | | LV3458 | 0 | | 0 | | 0 | | 1 | | 0 | | 35.978 | | 3.343171937 | | | KD4263 | 0 | | 0 | | 1 | | 1 | | 33.046 | | 32.326 | | 32.42358118 | |  |  |
| 686 | M | | 253 | | 131 | | SP2656 | | 0 | | 0 | | 0 | | 0 | | 0.00 | | 0.00 | | 0 | | | LV3455 | 0 | | 0 | | 1 | | 1 | | 36.479 | | 36.349 | | 2.486652558 | | | KD4260 | 0 | | 0 | | 1 | | 1 | | 33.906 | | 33.805 | | 14.19882466 | |  |  |
| 687 | F | | 332 | | 399 | | SP2661 | | 0 | | 0 | | 0 | | 0 | | 0.00 | | 0.00 | | 0 | | | LV3460 | 0 | | 0 | | 0 | | 1 | | 0 | | 36.113 | | 3.049478866 | | | KD4265 | 0 | | 0 | | 1 | | 1 | | 32.69 | | 32.201 | | 37.58969592 | |  |  |
| 688 | M | | 262 | | 172 | | SP2646 | | 0 | | 0 | | 0 | | 0 | | 0.00 | | 0.00 | | 0 | | | LV3445 | 0 | | 0 | | 0 | | 1 | | 0 | | 35.61 | | 4.295496245 | | | KD4250 | 0 | | 0 | | 1 | | 1 | | 34.416 | | 35.125 | | 7.832065989 | |  |  |
| 689 | F | | 354 | | 506 | | SP2662 | | 0 | | 0 | | 0 | | 0 | | 0.00 | | 0.00 | | 0 | | | LV3461 | 0 | | 0 | | 1 | | 0 | | 36.627 | | 37.914 | | 2.148741171 | | | KD4266 | 0 | | 0 | | 1 | | 1 | | 32.096 | | 34.113 | | 29.47326424 | |  |  |
| 690 | M | | 243 | | 125 | | SP2649 | | 0 | | 0 | | 0 | | 0 | | 0.00 | | 0.00 | | 0 | | | LV3448 | 0 | | 0 | | 0 | | 1 | | 39.023 | | 37.717 | | 1.022731002 | | | KD4253 | 0 | | 0 | | 1 | | 1 | | 33.414 | | 34.093 | | 15.61981997 | |  |  |
| 691 | F | | 324 | | 376 | | SP2653 | | 0 | | 0 | | 0 | | 0 | | 0.00 | | 0.00 | | 0 | | | LV3452 | 0 | | 0 | | 0 | | 0 | | 37.821 | | 0 | | 0 | | | KD4257 | 0 | | 0 | | 1 | | 1 | | 33.914 | | 34.378 | | 11.78867656 | |  |  |
| 692 | M | | 262 | | 173 | | SP2652 | | 0 | | 0 | | 0 | | 0 | | 0.00 | | 0.00 | | 0 | | | LV3451 | 0 | | 0 | | 0 | | 0 | | 0 | | 0 | | 0 | | | KD4256 | 0 | | 0 | | 1 | | 1 | | 34.803 | | 33.931 | | 10.46087447 | |  |  |
| 693 | F | | 350 | | 518 | | SP2660 | | 0 | | 0 | | 0 | | 0 | | 0.00 | | 0.00 | | 0 | | | LV3459 | 0 | | 0 | | 1 | | 1 | | 37.463 | | 36.777 | | 1.577973121 | | | KD4264 | 0 | | 0 | | 1 | | 1 | | 34.652 | | 34.444 | | 8.876510255 | |  |  |
| 694 | M | | 239 | | 120 | | SP2651 | | 0 | | 0 | | 0 | | 0 | | 0.00 | | 0.00 | | 0 | | | LV3450 | 0 | | 0 | | 1 | | 1 | | 36.997 | | 37.561 | | 1.403732107 | | | KD4255 | 0 | | 0 | | 1 | | 1 | | 33.714 | | 34.744 | | 11.68694793 | |  |  |
| 695 | F | | 376 | | 612 | | SP2657 | | 0 | | 0 | | 0 | | 0 | | 0.00 | | 0.00 | | 0 | | | LV3457 | 0 | | 0 | | 0 | | 1 | | 0 | | 37.324 | | 1.336629579 | | | KD4262 | 0 | | 0 | | 1 | | 1 | | 33.388 | | 33.467 | | 19.00007732 | |  |  |
| 696 | M | | 250 | | 131 | | SP2648 | | 0 | | 0 | | 0 | | 0 | | 0.00 | | 0.00 | | 0 | | | LV3447 | 0 | | 0 | | 0 | | 1 | | 38.034 | | 37.35 | | 1.313167879 | | | KD4252 | 0 | | 0 | | 1 | | 1 | | 33.822 | | 31.576 | | 40.77426549 | |  |  |
| 697 | F | | 324 | | 402 | | SP2663 | | 0 | | 0 | | 0 | | 0 | | 0.00 | | 0.00 | | 0 | | | LV3462 | 0 | | 0 | | 0 | | 0 | | 0 | | 38.172 | | 0 | | | KD4267 | 0 | | 0 | | 1 | |  | | 36.846 | |  | | 1.850988701 | |  |  |
| 698 | M | | 240 | | 140 | | SP2666 | | 0 | | 0 | | 0 | | 0 | | 0.00 | | 0.00 | | 0 | | | LV3465 | 0 | | 0 | | 1 | | 0 | | 37.283 | | 38.012 | | 1.374481482 | | | KD4270 | 0 | | 0 | | 1 | |  | | 37.386 | |  | | 1.281360785 | |  |  |
| 699 | F | | 333 | | 412 | | SP2665 | | 0 | | 0 | | 0 | | 0 | | 0.00 | | 0.00 | | 0 | | | LV3464 | 0 | | 0 | | 1 | | 1 | | 36.01 | | 36.397 | | 2.89212179 | | | KD4269 | 0 | | 0 | | 1 | |  | | 33.873 | |  | | 14.0222929 | |  |  |
| 700 | M | | 255 | | 174 | | SP2667 | | 0 | | 0 | | 0 | | 0 | | 0.00 | | 0.00 | | 0 | | | LV3466 | 0 | | 0 | | 1 | | 0 | | 36.393 | | 0 | | 2.520005288 | | | KD4271 | 0 | | 0 | | 0 | |  | | 38 | |  | | 0 | |  |  |
| 701 | F | | 315 | | 376 | | SP2668 | | 0 | | 0 | | 0 | | 0 | | 0.00 | | 0.00 | | 0 | | | LV3467 | 0 | | 0 | | 1 | | 1 | | 36.587 | | 37.704 | | 1.619956801 | | | KD4272 | 0 | | 0 | | 1 | |  | | 37.491 | |  | | 1.192922864 | |  |  |
| 702 | M | | 219 | | 108 | | SP2664 | | 0 | | 0 | | 0 | | 0 | | 0.00 | | 0.00 | | 0 | | | LV3463 | 0 | | 0 | | 1 | | 1 | | 37.175 | | 37.241 | | 1.446883728 | | | KD4268 | 0 | | 0 | | 1 | |  | | 34.767 | |  | | 7.627342932 | |  |  |
| 703 | F | | 272 | | 258 | | SP2671 | | 0 | | 0 | | 0 | | 0 | | 0.00 | | 0.00 | | 0 | | | LV3470 | 0 | | 0 | | 0 | | 1 | | 0 | | 36.691 | | 2.057087973 | | | KD4274 | 0 | | 0 | | 1 | |  | | 35.969 | |  | | 3.363728372 | |  |  |
| 704 | M | | 232 | | 113 | | SP2674 | | 0 | | 0 | | 0 | | 0 | | 0.00 | | 0.00 | | 0 | | | LV3473 | 0 | | 0 | | 0 | | 1 | | 0 | | 35.522 | | 4.5608291 | | | KD4353 | 0 | | 0 | | 0 | | 0 | | 0 | | 0 | | 0 | |  |  |
| 705 | F | | 341 | | 465 | | SP2673 | | 0 | | 0 | | 0 | | 0 | | 0.00 | | 0.00 | | 0 | | | LV3472 | 0 | | 0 | | 0 | | 1 | | 0 | | 36.608 | | 2.176728768 | | | KD4351 | 0 | | 0 | | 1 | | 1 | | 35.539 | | 36.299 | | 3.597473801 | |  |  |
| 706 | M | | 276 | | 191 | | SP2670 | | 0 | | 0 | | 0 | | 0 | | 0.00 | | 0.00 | | 0 | | | LV3469 | 0 | | 0 | | 1 | | 1 | | 36.782 | | 37.258 | | 1.665772626 | | | KD4275 | 0 | | 0 | | 0 | |  | | 38.802 | |  | | 0 | |  |  |
| 707 | F | | 359 | | 506 | | SP2676 | | 0 | | 0 | | 0 | | 0 | | 0.00 | | 0.00 | | 0 | | | LV3475 | 0 | | 0 | | 0 | | 1 | | 0 | | 34.297 | | 10.50508494 | | | KD4354 | 0 | | 0 | | 1 | | 1 | | 36.957 | | 37.112 | | 1.630234625 | |  |  |
| 708 | M | | 278 | | 206 | | SP2669 | | 0 | | 0 | | 0 | | 0 | | 0.00 | | 0.00 | | 0 | | | LV3468 | 0 | | 0 | | 0 | | 0 | | 37.954 | | 0 | | 0 | | | KD4273 | 0 | | 0 | | 0 | |  | | 38.699 | |  | | 0 | |  |  |
| 709 | F | | 322 | | 305 | | SP2679 | | 0 | | 0 | | 0 | | 0 | | 0.00 | | 0.00 | | 0 | | | LV3478 | 0 | | 0 | | 1 | | 0 | | 37.321 | | 0 | | 1.339363531 | | | KD4358 | 0 | | 0 | | 1 | | 0 | | 36.532 | | 0 | | 2.292372368 | |  |  |
| 710 | M | | 236 | | 215 | | SP2672 | | 0 | | 0 | | 0 | | 0 | | 0.00 | | 0.00 | | 0 | | | LV3471 | 0 | | 0 | | 0 | | 0 | | 0 | | 0 | | 0 | | | KD4352 | 1 | | 0 | | 1 | | 0 | | 37.453 | | 38.515 | | 1.224201159 | |  |  |
| 711 | F | | 306 | | 394 | | SP2683 | | 0 | | 0 | | 0 | | 0 | | 0.00 | | 0.00 | | 0 | | | LV3482 | 0 | | 0 | | 1 | | 0 | | 36.766 | | 0 | | 1.954644662 | | | KD4362 | 0 | | 0 | | 1 | | 1 | | 36.467 | | 35.946 | | 2.906488827 | |  |  |
| 712 | M | | 260 | | 180 | | SP2675 | | 0 | | 0 | | 0 | | 0 | | 0.00 | | 0.00 | | 0 | | | LV3474 | 0 | | 0 | | 0 | | 0 | | 0 | | 0 | | 0 | | | KD4355 | 0 | | 0 | | 1 | | 0 | | 35.536 | | 0 | | 4.51754607 | |  |  |
| 713 | F | | 394 | | 678 | | SP2681 | | 0 | | 0 | | 0 | | 0 | | 0.00 | | 0.00 | | 0 | | | LV3480 | 0 | | 0 | | 0 | | 0 | | 38.815 | | 0 | | 0 | | | KD4359 | 0 | | 0 | | 0 | | 1 | | 0 | | 37.17 | | 1.484445865 | |  |  |
| 714 | M | | 246 | | 148 | | SP2677 | | 0 | | 0 | | 0 | | 0 | | 0.00 | | 0.00 | | 0 | | | LV3476 | 0 | | 1 | | 0 | | 0 | | 0 | | 0 | | 0 | | | KD4356 | 0 | | 0 | | 1 | | 1 | | 36.328 | | 36.12 | | 2.834525751 | |  |  |
| 715 | F | | 307 | | 313 | | SP2658 | | 0 | | 0 | | 0 | | 0 | | 0.00 | | 0.00 | | 0 | | | LV3484 | 0 | | 0 | | 1 | | 1 | | 36.716 | | 36.476 | | 2.201926956 | | | KD4364 | 0 | | 0 | | 1 | | 1 | | 36.293 | | 37.232 | | 2.060344785 | |  |  |
| 716 | M | | 225 | | 103 | | SP2678 | | 0 | | 1 | | 0 | | 0 | | 0.00 | | 0.00 | | 0 | | | LV3477 | 0 | | 0 | | 0 | | 0 | | 0 | | 37.846 | | 0 | | | KD4357 | 0 | | 0 | | 0 | | 1 | | 0 | | 36.812 | | 1.894353344 | |  |  |
| 717 | F | | 345 | | 349 | | SP2687 | | 0 | | 0 | | 0 | | 0 | | 0.00 | | 0.00 | | 0 | | | LV3486 | 0 | | 0 | | 1 | | 0 | | 37.192 | | 0 | | 1.462368253 | | | KD4366 | 0 | | 0 | | 1 | | 1 | | 37.01 | | 36.074 | | 2.393464036 | |  |  |
| 718 | M | | 251 | | 137 | | SP2686 | | 0 | | 0 | | 0 | | 0 | | 0.00 | | 0.00 | | 0 | | | LV3485 | 0 | | 0 | | 1 | | 0 | | 37.652 | | 0 | | 1.069026477 | | | KD4350 |  | |  | |  | |  | |  | |  | |  | |  |  |
| 719 | F | | 372 | | 656 | | SP2690 | | 0 | | 0 | | 0 | | 0 | | 0.00 | | 0.00 | | 0 | | | LV3489 | 0 | | 0 | | 0 | | 0 | | 0 | | 38 | | 0 | | | KD4368 | 0 | | 0 | | 1 | | 1 | | 35.611 | | 37.366 | | 2.795753309 | |  |  |
| 720 | M | | 246 | | 135 | | SP2680 | | 0 | | 0 | | 0 | | 0 | | 0.00 | | 0.00 | | 0 | | | LV3479 | 0 | | 0 | | 0 | | 1 | | 37.997 | | 37.169 | | 1.485457274 | | | KD4360 | 0 | | 0 | | 0 | | 1 | | 37.979 | | 35.671 | | 4.120685841 | |  |  |
| 721 | F | | 352 | | 471 | | SP2692 | | 0 | | 0 | | 0 | | 0 | | 0.00 | | 0.00 | | 0 | | | LV3491 | 0 | | 0 | | 0 | | 0 | | 0 | | 0 | | 0 | | | KD4371 | 0 | | 0 | | 1 | | 1 | | 36.547 | | 36.185 | | 2.586306249 | |  |  |
| 722 | M | | 228 | | 121 | | SP2684 | | 0 | | 0 | | 0 | | 0 | | 0.00 | | 0.00 | | 0 | | | LV3483 | 0 | | 0 | | 0 | | 1 | | 0 | | 37.387 | | 1.280488339 | | | KD4363 | 0 | | 0 | | 0 | | 1 | | 0 | | 36.286 | | 2.710516899 | |  |  |
| 723 | F | | 309 | | 352 | | SP2695 | | 0 | | 0 | | 0 | | 0 | | 0.00 | | 0.00 | | 0 | | | LV3494 | 0 | | 0 | | 1 | | 0 | | 30.556 | | 0 | | 134.2733169 | | | KD4374 | 0 | | 0 | | 0 | | 0 | | 0 | | 38.396 | | 0 | |  |  |
| 724 | M | | 273 | | 190 | | SP2688 | | 0 | | 0 | | 0 | | 0 | | 0.00 | | 0.00 | | 0 | | | LV3487 | 0 | | 0 | | 0 | | 0 | | 39.422 | | 0 | | 0 | | | KD4367 |  | |  | |  | |  | |  | |  | |  | |  |  |
| 725 | F | | 342 | | 491 | | SP2697 | | 0 | | 0 | | 0 | | 0 | | 0.00 | | 0.00 | | 0 | | | LV3496 | 0 | | 0 | | 0 | | 0 | | 0 | | 0 | | 0 | | | KD4376 | 0 | | 0 | | 0 | | 0 | | 38.786 | | 38.176 | | 0 | |  |  |
| 726 | M | | 226 | | 116 | | SP2682 | | 0 | | 0 | | 0 | | 0 | | 0.00 | | 0.00 | | 0 | | | LV3481 | 0 | | 0 | | 0 | | 1 | | 37.896 | | 37.645 | | 1.074135489 | | | KD4361 | 0 | | 0 | | 1 | | 0 | | 37.183 | | 37.88 | | 1.471360037 | |  |  |
| 727 | F | | 348 | | 466 | | SP2699 | | 0 | | 1 | | 0 | | 0 | | 0.00 | | 0.00 | | 0 | | | LV3498 | 0 | | 1 | | 1 | | 0 | | 36.275 | | 0 | | 2.730900809 | | | KD4378 | 0 | | 0 | | 1 | | 1 | | 35.051 | | 34.179 | | 8.835058609 | |  |  |
| 728 | M | | 222 | | 119 | | SP2701 | | 0 | | 0 | | 0 | | 0 | | 0.00 | | 0.00 | | 0 | | | LV3500 | 0 | | 0 | | 0 | | 0 | | 0 | | 0 | | 0 | | | KD4380 | 0 | | 0 | | 0 | | 1 | | 37.854 | | 36.539 | | 2.281468939 | |  |  |
| 729 | F | | 341 | | 534 | | SP2702 | | 0 | | 0 | | 0 | | 0 | | 0.00 | | 0.00 | | 0 | | | LV3501 | 0 | | 0 | | 0 | | 0 | | 0 | | 0 | | 0 | | | KD4381 | 0 | | 0 | | 1 | | 1 | | 35.629 | | 37.009 | | 2.948377093 | |  |  |
| 730 | M | | 263 | | 193 | | SP2689 | | 0 | | 0 | | 0 | | 0 | | 0.00 | | 0.00 | | 0 | | | LV3488 | 0 | | 0 | | 0 | | 0 | | 0 | | 0 | | 0 | | | KD4369 | 0 | | 0 | | 1 | | 1 | | 37.698 | | 37.017 | | 1.341769336 | |  |  |
| 731 | F | | 333 | | 500 | | SP2704 | | 0 | | 0 | | 0 | | 0 | | 0.00 | | 0.00 | | 0 | | | LV3503 | 0 | | 0 | | 1 | | 0 | | 34.568 | | 0 | | 8.734490927 | | | KD4383 | 0 | | 0 | | 1 | | 1 | | 35.379 | | 36.053 | | 4.10203984 | |  |  |
| 732 | M | | 261 | | 182 | | SP2693 | | 0 | | 0 | | 0 | | 0 | | 0.00 | | 0.00 | | 0 | | | LV3492 | 0 | | 0 | | 1 | | 0 | | 36.883 | | 0 | | 1.804924934 | | | KD4372 | 0 | | 0 | | 1 | | 1 | | 36.896 | | 37.331 | | 1.559643014 | |  |  |
| 733 | F | | 310 | | 312 | | SP2707 | | 0 | | 0 | | 0 | | 0 | | 0.00 | | 0.00 | | 0 | | | LV3506 | 0 | | 0 | | 0 | | 0 | | 0 | | 0 | | 0 | | | KD4385 | 0 | | 0 | | 1 | | 1 | | 36.385 | | 36.604 | | 2.35822054 | |  |  |
| 734 | M | | 250 | | 146 | | SP2698 | | 0 | | 0 | | 0 | | 0 | | 0.00 | | 0.00 | | 0 | | | LV3497 | 0 | | 0 | | 0 | | 0 | | 0 | | 0 | | 0 | | | KD4377 | 0 | | 0 | | 1 | | 1 | | 33.507 | | 34.925 | | 12.42063007 | |  |  |
| 735 | F | | 358 | | 513 | | SP2706 | | 0 | | 0 | | 0 | | 0 | | 0.00 | | 0.00 | | 0 | | | LV3505 | 0 | | 0 | | 0 | | 0 | | 0 | | 0 | | 0 | | | KD4386 | 0 | | 0 | | 1 | | 1 | | 36.829 | | 35.547 | | 3.178185915 | |  |  |
| 736 | M | | 228 | | 113 | | SP2703 | | 0 | | 0 | | 0 | | 0 | | 0.00 | | 0.00 | | 0 | | | LV3502 | 0 | | 0 | | 0 | | 1 | | 0 | | 35.358 | | 5.099822985 | | | KD4382 | 0 | | 0 | | 0 | | 1 | | 38.227 | | 35.982 | | 3.334076114 | |  |  |
| 737 | F | | 371 | | 594 | | SP2708 | | 1 | | 1 | | 0 | | 0 | | 0.00 | | 0.00 | | 0 | | | LV3507 | 0 | | 0 | | 0 | | 0 | | 0 | | 0 | | 0 | | | KD4387 | 0 | | 0 | | 1 | | 1 | | 37.379 | | 37.246 | | 1.348522203 | |  |  |
| 738 | M | | 254 | | 155 | | SP2691 | | 0 | | 0 | | 0 | | 0 | | 0.00 | | 0.00 | | 0 | | | LV3490 | 0 | | 0 | | 0 | | 0 | | 0 | | 0 | | 0 | | | KD4370 | 0 | | 0 | | 1 | | 1 | | 37.731 | | 37.129 | | 1.26975444 | |  |  |
| 739 | F | | 355 | | 565 | | SP2705 | | 0 | | 0 | | 0 | | 0 | | 0.00 | | 0.00 | | 0 | | | LV3504 | 0 | | 0 | | 0 | | 0 | | 0 | | 0 | | 0 | | | KD4384 | 0 | | 0 | | 1 | | 0 | | 37.323 | | 0 | | 1.337540275 | |  |  |
| 740 | M | | 250 | | 163 | | SP2696 | | 0 | | 0 | | 0 | | 0 | | 0.00 | | 0.00 | | 0 | | | LV3495 | 0 | | 0 | | 1 | | 0 | | 37.69 | | 0 | | 1.041712889 | | | KD4375 | 0 | | 0 | | 0 | | 0 | | 0 | | 0 | | 0 | |  |  |
| 741 | F | | 329 | | 729 | | SP2709 | | 0 | | 0 | | 0 | | 0 | | 0.00 | | 0.00 | | 0 | | | LV3508 | 0 | | 0 | | 0 | | 0 | | 0 | | 0 | | 0 | | | KD4388 | 0 | | 0 | | 1 | | 1 | | 35.409 | | 35.857 | | 4.278041184 | |  |  |
| 742 | M | | 255 | | 153 | | SP2700 | | 0 | | 0 | | 0 | | 0 | | 0.00 | | 0.00 | | 0 | | | LV3499 | 0 | | 0 | | 1 | | 0 | | 37.23 | | 37.945 | | 1.425004798 | | | KD4379 | 0 | | 0 | | 0 | | 1 | | 39.069 | | 36.352 | | 2.591369112 | |  |  |
| 743 | F | | 338 | | 519 | | SP2710 | | 0 | | 0 | | 0 | | 0 | | 0.00 | | 0.00 | | 0 | | | LV3509 | 0 | | 0 | | 0 | | 0 | | 0 | | 0 | | 0 | | | KD4389 | 0 | | 0 | | 0 | | 1 | | 0 | | 34.332 | | 10.25761858 | |  |  |
| 744 | M | | 229 | | 122 | | SP2694 | | 0 | | 0 | | 0 | | 0 | | 0.00 | | 0.00 | | 0 | | | LV3493 | 0 | | 0 | | 0 | | 1 | | 0 | | 37.08 | | 1.578288604 | | | KD4373 | 0 | | 0 | | 1 | | 0 | | 35.943 | | 37.958 | | 3.423826387 | |  |  |
| 745 | F | | 348 | | 451 | | SP2711 | | 0 | | 0 | | 0 | | 0 | | 0.00 | | 0.00 | | 0 | | | LV3510 | 0 | | 0 | | 0 | | 0 | | 0 | | 0 | | 0 | | | KD4390 | 0 | | 0 | | 0 | | 0 | | 0 | | 0 | | 0 | |  |  |
| 746 | M | | 238 | | 136 | | SP2714 | | 0 | | 1 | | 0 | | 0 | | 0.00 | | 0.00 | | 0 | | | LV3513 | 0 | | 0 | | 0 | | 0 | | 0 | | 0 | | 0 | | | KD4393 | 0 | | 0 | | 1 | | 1 | | 35.374 | | 36.698 | | 3.545926017 | |  |  |
| 747 | F | | 308 | | 324 | | SP2716 | | 0 | | 0 | | 0 | | 0 | | 0.00 | | 0.00 | | 0 | | | LV3515 | 0 | | 0 | | 0 | | 0 | | 0 | | 0 | | 0 | | | KD4394 | 0 | | 0 | | 1 | | 1 | | 36.432 | | 33.621 | | 9.550949209 | |  |  |
| 748 | M | | 235 | | 126 | | SP2715 | | 0 | | 0 | | 0 | | 0 | | 0.00 | | 0.00 | | 0 | | | LV3514 | 0 | | 0 | | 0 | | 0 | | 0 | | 0 | | 0 | | | KD4395 | 0 | | 0 | | 1 | | 1 | | 34.531 | | 34.789 | | 8.235654756 | |  |  |
| 749 | F | | 370 | | 587 | | SP2713 | | 0 | | 0 | | 0 | | 0 | | 0.00 | | 0.00 | | 0 | | | LV3512 | 0 | | 0 | | 0 | | 0 | | 0 | | 0 | | 0 | | | KD4392 | 0 | | 0 | | 1 | | 1 | | 36.971 | | 36.907 | | 1.737790643 | |  |  |
| 750 | M | | 272 | | 194 | | SP2712 | | 0 | | 0 | | 0 | | 0 | | 0.00 | | 0.00 | | 0 | | | LV3511 | 0 | | 0 | | 0 | | 0 | | 0 | | 0 | | 0 | | | KD4391 | 0 | | 0 | | 0 | | 1 | | 0 | | 36.433 | | 2.452276447 | |  |  |
| 751 | F | | 312 | | 324 | | SP2719 | | 0 | | 0 | | 0 | | 0 | | 0.00 | | 0.00 | | 0 | | | LV3518 | 0 | | 0 | | 0 | | 0 | | 0 | | 0 | | 0 | | | KD4397 | 0 | | 0 | | 1 | | 0 | | 36.789 | | 0 | | 1.924262886 | |  |  |
| 752 | M | | 226 | | 118 | | SP2717 | | 0 | | 0 | | 0 | | 0 | | 0.00 | | 0.00 | | 0 | | | LV3516 | 0 | | 0 | | 0 | | 0 | | 0 | | 0 | | 0 | | | KD4396 | 0 | | 0 | | 1 | | 1 | | 37.077 | | 37.347 | | 1.448685345 | |  |  |
| 753 | F | | 354 | | 572 | | SP2721 | | 0 | | 0 | | 0 | | 0 | | 0.00 | | 0.00 | | 0 | | | LV3520 | 0 | | 0 | | 0 | | 0 | | 0 | | 0 | | 0 | | | KD4400 | 0 | | 0 | | 0 | | 0 | | 38 | | 37.904 | | 0 | |  |  |
| 754 | M | | 244 | | 153 | | SP2718 | | 0 | | 0 | | 0 | | 0 | | 0.00 | | 0.00 | | 0 | | | LV3517 | 0 | | 0 | | 0 | | 0 | | 0 | | 0 | | 0 | | | KD4398 | 0 | | 0 | | 0 | | 0 | | 0 | | 37.984 | | 0 | |  |  |
| 755 | F | | 366 | | 594 | | SP2724 | | 0 | | 0 | | 0 | | 0 | | 0.00 | | 0.00 | | 0 | | | LV3523 | 0 | | 0 | | 0 | | 0 | | 0 | | 39.624 | | 0 | | | KD4277 | 0 | | 0 | | 1 | |  | | 36.753 | |  | | 1.972028677 | |  |  |
| 756 | M | | 202 | | 88 | | SP2720 | | 0 | | 0 | | 0 | | 0 | | 0.00 | | 0.00 | | 0 | | | LV3519 | 0 | | 0 | | 0 | | 0 | | 0 | | 0 | | 0 | | | KD4399 | 0 | | 0 | | 1 | | 1 | | 36.857 | | 36.051 | | 2.509092154 | |  |  |
| 757 | F | | 286 | | 269 | | SP2726 | | 0 | | 0 | | 1 | | 1 | | 37.58 | | 35.687 | | 2.599008531 | | | LV3525 | 0 | | 0 | | 0 | | 0 | | 38.918 | | 0 | | 0 | | | KD4280 | 0 | | 0 | | 1 | |  | | 36.225 | |  | | 2.825504202 | |  |  |
| 758 | M | | 235 | | 139 | | SP2722 | | 0 | | 0 | | 0 | | 0 | | 0.00 | | 0.00 | | 0 | | | LV3521 | 0 | | 0 | | 0 | | 0 | | 0 | | 0 | | 0 | | | KD4276 | 0 | | 0 | | 0 | |  | | 38.22 | |  | | 0 | |  |  |
| 759 | F | | 360 | | 502 | | SP2727 | | 0 | | 0 | | 0 | | 1 | | 0.00 | | 36.061 | | 3.159419254 | | | LV3526 | 0 | | 0 | | 0 | | 0 | | 0 | | 0 | | 0 | | | KD4281 | 0 | | 0 | | 1 | |  | | 34.794 | |  | | 7.488358989 | |  |  |
| 760 | M | | 250 | | 147 | | SP2743 | | 0 | | 0 | | 1 | | 1 | | 35.83 | | 36.138 | | 3.347870044 | | | LV3542 | 0 | | 0 | | 0 | | 0 | | 0 | | 0 | | 0 | | | KD4297 | 0 | | 0 | | 0 | | 1 | | 37.842 | | 37.176 | | 1.478391852 | |  |  |
| 761 | F | | 340 | | 450 | | SP2729 | | 0 | | 0 | | 0 | | 0 | | 38.20 | | 38.631 | | 0 | | | LV3528 | 0 | | 0 | | 0 | | 0 | | 0 | | 38.603 | | 0 | | | KD4283 | 0 | | 0 | | 1 | |  | | 36.024 | |  | | 3.24005128 | |  |  |
| 762 | M | | 201 | | 82 | | SP2723 | | 0 | | 0 | | 0 | | 0 | | 0.00 | | 0.00 | | 0 | | | LV3522 | 0 | | 0 | | 0 | | 0 | | 0 | | 0 | | 0 | | | KD4278 | 0 | | 0 | | 0 | |  | | 38.081 | |  | | 0 | |  |  |
| 763 | F | | 296 | | 317 | | SP2731 | | 0 | | 0 | | 0 | | 1 | | 0.00 | | 37.138 | | 1.51715511 | | | LV3530 | 0 | | 0 | | 0 | | 0 | | 0 | | 0 | | 0 | | | KD4285 | 0 | | 0 | | 1 | |  | | 35.01 | |  | | 6.463886477 | |  |  |
| 764 | M | | 258 | | 165 | | SP2728 | | 0 | | 0 | | 1 | | 1 | | 37.22 | | 36.844 | | 1.643151242 | | | LV3527 | 0 | | 0 | | 0 | | 0 | | 0 | | 0 | | 0 | | | KD4282 | 0 | | 0 | | 1 | |  | | 37.233 | |  | | 1.422096032 | |  |  |
| 765 | F | | 341 | | 444 | | SP2734 | | 0 | | 0 | | 0 | | 0 | | 0.00 | | 37.970 | | 0 | | | LV3532 | 0 | | 0 | | 0 | | 0 | | 0 | | 0 | | 0 | | | KD4287 | 0 | | 0 | | 0 | |  | | 38.242 | |  | | 0 | |  |  |
| 766 | M | | 224 | | 126 | | SP2730 | | 0 | | 0 | | 1 | | 1 | | 35.81 | | 36.999 | | 2.710690717 | | | LV3529 | 0 | | 0 | | 0 | | 0 | | 0 | | 0 | | 0 | | | KD4284 | 0 | | 0 | | 0 | |  | | 38.1 | |  | | 0 | |  |  |
| 767 | F | | 334 | | 492 | | SP2736 | | 0 | | 0 | | 0 | | 1 | | 38.15 | | 36.975 | | 1.695295802 | | | LV3535 | 0 | | 0 | | 1 | | 0 | | 37.686 | | 0 | | 1.044554827 | | | KD4289 | 0 | | 0 | |  | |  | |  | |  | |  | |  |  |
| 768 | M | | 226 | | 119 | | SP2725 | | 0 | | 0 | | 1 | | 1 | | 37.18 | | 35.664 | | 2.808882187 | | | LV3524 | 0 | | 0 | | 0 | | 0 | | 0 | | 0 | | 0 | | | KD4279 | 0 | | 0 | | 1 | |  | | 36.254 | |  | | 2.770242191 | |  |  |
| 769 | F | | 316 | | 379 | | SP2742 | | 0 | | 0 | | 0 | | 0 | | 38.03 | | 0.00 | | 0 | | | LV3541 | 0 | | 0 | | 0 | | 0 | | 0 | | 0 | | 0 | | | KD4295 | 0 | | 0 | | 1 | | 1 | | 36.457 | | 36.674 | | 2.246780537 | |  |  |
| 770 | M | | 279 | | 219 | | SP2732 | | 0 | | 0 | | 0 | | 1 | | 0.00 | | 36.555 | | 2.256741162 | | | LV3531 | 0 | | 0 | | 0 | | 0 | | 0 | | 0 | | 0 | | | KD4286 | 0 | | 0 | | 1 | |  | | 35.363 | |  | | 5.082484921 | |  |  |
| 771 | F | | 354 | | 521 | | SP2738 | | 0 | | 0 | | 0 | | 1 | | 0.00 | | 35.588 | | 4.36034605 | | | LV3537 | 0 | | 0 | | 0 | | 0 | | 0 | | 0 | | 0 | | | KD4292 | 0 | | 0 | | 1 | | 0 | | 37.49 | | 0 | | 1.193735648 | |  |  |
| 772 | M | | 230 | | 123 | | SP2741 | | 0 | | 0 | | 0 | | 0 | | 0.00 | | 37.974 | | 0 | | | LV3540 | 0 | | 0 | | 0 | | 0 | | 0 | | 0 | | 0 | | | KD4296 | 0 | | 0 | | 0 | | 0 | | 0 | | 0 | | 0 | |  |  |
| 773 | F | | 350 | | 481 | | SP2740 | | 0 | | 0 | | 0 | | 1 | | 37.91 | | 36.513 | | 2.322230778 | | | LV3539 | 0 | | 0 | | 0 | | 0 | | 0 | | 0 | | 0 | | | KD4294 | 0 | | 0 | | 1 | | 0 | | 36.953 | | 0 | | 1.720889958 | |  |  |
| 774 | M | | 293 | | 218 | | SP2735 | | 0 | | 0 | | 1 | | 0 | | 36.87 | | 38.200 | | 1.824702027 | | | LV3534 | 0 | | 0 | | 0 | | 0 | | 0 | | 0 | | 0 | | | KD4290 | 0 | | 0 | | 1 | | 1 | | 35.08 | | 37.021 | | 3.902970203 | |  |  |
| 775 | F | | 346 | | 455 | | SP2746 | | 0 | | 0 | | 1 | | 0 | | 37.16 | | 37.957 | | 1.495609348 | | | LV3545 | 0 | | 0 | | 0 | | 0 | | 0 | | 0 | | 0 | | | KD4300 | 0 | | 0 | | 1 | | 1 | | 37.21 | | 35.813 | | 2.592680316 | |  |  |
| 776 | M | | 239 | | 140 | | SP2749 | | 0 | | 0 | | 0 | | 1 | | 38.32 | | 36.151 | | 2.971564792 | | | LV3548 | 0 | | 0 | | 0 | | 0 | | 0 | | 0 | | 0 | | | KD4303 | 0 | | 0 | | 1 | | 1 | | 34.863 | | 37.071 | | 4.366284187 | |  |  |
| 777 | F | | 375 | | 655 | | SP2748 | | 0 | | 0 | | 1 | | 1 | | 37.22 | | 36.983 | | 1.560413617 | | | LV3547 | 0 | | 0 | | 0 | | 0 | | 0 | | 0 | | 0 | | | KD4302 | 0 | | 0 | | 1 | | 1 | | 36.501 | | 36.665 | | 2.217564847 | |  |  |
| 778 | M | | 250 | | 154 | | SP2737 | | 0 | | 0 | | 0 | | 1 | | 0.00 | | 37.321 | | 1.339363531 | | | LV3536 | 0 | | 0 | | 0 | | 0 | | 0 | | 37.888 | | 0 | | | KD4291 | 0 | | 0 | | 1 | | 1 | | 34.899 | | 37.148 | | 4.239189296 | |  |  |
| 779 | F | | 365 | | 687 | | SP2751 | | 0 | | 0 | | 1 | | 1 | | 36.88 | | 36.750 | | 1.892339509 | | | LV3549 | 0 | | 0 | | 0 | | 0 | | 0 | | 0 | | 0 | | | KD4304 | 0 | | 0 | | 1 | | 0 | | 36.658 | | 0 | | 2.103847643 | |  |  |
| 780 | M | | 254 | | 158 | | SP2733 | | 0 | | 0 | | 0 | | 0 | | 38.09 | | 0.00 | | 0 | | | LV3533 | 0 | | 0 | | 0 | | 0 | | 0 | | 0 | | 0 | | | KD4288 | 0 | | 0 | | 1 | | 0 | | 35.06 | | 37.775 | | 6.247462947 | |  |  |
| 781 | F | | 314 | | 354 | | SP2754 | | 0 | | 0 | | 1 | | 1 | | 37.04 | | 37.135 | | 1.572174071 | | | LV3553 | 0 | | 0 | | 0 | | 0 | | 0 | | 0 | | 0 | | | KD4308 | 0 | | 0 | | 0 | | 1 | | 37.933 | | 36.868 | | 1.823459634 | |  |  |
| 782 | M | | 238 | | 143 | | SP2744 | | 1 | | 1 | | 0 | | 1 | | 38.19 | | 35.320 | | 5.233539735 | | | LV3543 | 0 | | 0 | | 0 | | 0 | | 0 | | 0 | | 0 | | | KD4298 | 0 | | 0 | | 0 | | 1 | | 0 | | 36.752 | | 1.973372295 | |  |  |
| 783 | F | | 330 | | 611 | | SP2756 | | 0 | | 0 | | 1 | | 1 | | 36.41 | | 37.550 | | 1.821012695 | | | LV3555 | 0 | | 0 | | 0 | | 0 | | 0 | | 38.774 | | 0 | | | KD4310 | 0 | | 0 | | 0 | | 1 | | 0 | | 37.741 | | 1.006148782 | |  |  |
| 784 | M | | 255 | | 163 | | SP2745 | | 0 | | 0 | | 1 | | 1 | | 36.84 | | 37.167 | | 1.674926094 | | | LV3544 | 0 | | 0 | | 0 | | 0 | | 0 | | 0 | | 0 | | | KD4299 | 0 | | 0 | | 1 | | 1 | | 34.215 | | 36.781 | | 6.521635707 | |  |  |
| 785 | F | | 351 | | 524 | | SP2759 | | 0 | | 0 | | 1 | | 1 | | 35.45 | | 36.417 | | 3.642776349 | | | LV3558 | 0 | | 0 | | 0 | | 0 | | 0 | | 0 | | 0 | | | KD4313 | 0 | | 0 | | 0 | | 1 | | 0 | | 35.227 | | 5.575771712 | |  |  |
| 786 | M | | 237 | | 127 | | SP2755 | | 0 | | 0 | | 1 | | 1 | | 36.39 | | 37.747 | | 1.762742908 | | | LV3554 | 0 | | 0 | | 0 | | 0 | | 0 | | 0 | | 0 | | | KD4309 | 0 | | 0 | | 1 | | 0 | | 36.524 | | 38.35 | | 2.30489725 | |  |  |
| 787 | F | | 318 | | 414 | | SP2757 | | 0 | | 0 | | 1 | | 1 | | 36.86 | | 36.796 | | 1.87426642 | | | LV3556 | 0 | | 0 | | 0 | | 0 | | 0 | | 0 | | 0 | | | KD4311 | 0 | | 0 | | 1 | | 1 | | 37.45 | | 36.952 | | 1.474383808 | |  |  |
| 788 | M | | 242 | | 142 | | SP2739 | | 0 | | 0 | | 1 | | 1 | | 37.12 | | 36.506 | | 1.933031864 | | | LV3538 | 0 | | 0 | | 0 | | 0 | | 38.445 | | 0 | | 0 | | | KD4293 | 0 | | 0 | | 1 | | 1 | | 36.995 | | 36.844 | | 1.762935357 | |  |  |
| 789 | F | | 351 | | 479 | | SP2760 | | 0 | | 0 | | 0 | | 1 | | 0.00 | | 37.421 | | 1.251176004 | | | LV3559 | 0 | | 0 | | 0 | | 0 | | 0 | | 0 | | 0 | | | KD4314 | 0 | | 0 | | 1 | | 1 | | 36.11 | | 37.003 | | 2.359493749 | |  |  |
| 790 | M | | 226 | | 137 | | SP2750 | | 0 | | 0 | | 1 | | 1 | | 35.85 | | 36.897 | | 2.721487216 | | | LV3550 | 0 | | 0 | | 0 | | 0 | | 0 | | 0 | | 0 | | | KD4305 | 0 | | 0 | | 1 | | 0 | | 36.115 | | 0 | | 3.045327657 | |  |  |
| 791 | F | | 281 | | 289 | | SP2761 | | 0 | | 0 | | 1 | | 0 | | 36.57 | | 38.071 | | 2.232281398 | | | LV3560 |  | |  | | 0 | | 0 | | 0 | | 0 | | 0 | | | KD4315 | 0 | | 0 | | 1 | | 1 | | 35.182 | | 36.566 | | 3.994605125 | |  |  |
| 792 | M | | 244 | | 150 | | SP2752 | | 0 | | 0 | | 1 | | 1 | | 35.69 | | 36.452 | | 3.242839951 | | | LV3551 | 0 | | 0 | | 0 | | 0 | | 0 | | 0 | | 0 | | | KD4306 | 0 | | 0 | | 0 | | 1 | | 0 | | 37.309 | | 1.350355373 | |  |  |
| 793 | F | | 236 | | 373 | | SP2763 | | 0 | | 0 | | 1 | | 1 | | 37.23 | | 26.630 | | 974.0723344 | | | LV3562 | 0 | | 0 | | 0 | | 0 | | 0 | | 0 | | 0 | | | KD4317 | 0 | | 0 | | 0 | | 1 | | 0 | | 36.735 | | 1.996354382 | |  |  |
| 794 | M | | 263 | | 198 | | SP2753 | | 0 | | 0 | | 1 | | 1 | | 36.84 | | 37.421 | | 1.552343931 | | | LV3552 | 0 | | 0 | | 0 | | 0 | | 0 | | 0 | | 0 | | | KD4307 | 0 | | 0 | | 0 | | 0 | | 0 | | 37.884 | | 0 | |  |  |
| 795 | F | | 335 | | 432 | | SP2758 | | 0 | | 0 | | 1 | | 1 | | 36.35 | | 36.622 | | 2.37460298 | | | LV3557 | 0 | | 0 | | 0 | | 0 | | 38.802 | | 0 | | 0 | | | KD4312 | 0 | | 0 | | 0 | | 0 | | 38.149 | | 0 | | 0 | |  |  |
| 796 | M | | 226 | | 139 | | SP2747 | | 0 | | 0 | | 1 | | 0 | | 35.82 | | 37.874 | | 3.717950562 | | | LV3546 | 0 | | 0 | | 0 | | 0 | | 38 | | 0 | | 0 | | | KD4301 | 0 | | 0 | | 1 | | 1 | | 37.306 | | 36.44 | | 1.896864926 | |  |  |
| 797 | F | | 319 | | 335 | | SP2764 | | 0 | | 0 | | 1 | | 1 | | 36.42 | | 37.165 | | 1.978432384 | | | LV3563 | 0 | | 0 | | 0 | | 0 | | 0 | | 0 | | 0 | | | KD4318 | 0 | | 0 | | 0 | | 0 | | 37.817 | | 0 | | 0 | |  |  |
| 798 | M | | 232 | | 117 | | SP2768 | | 0 | | 0 | | 1 | | 1 | | 36.78 | | 36.552 | | 2.096749928 | | | LV3567 | 0 | | 0 | | 0 | | 0 | | 0 | | 0 | | 0 | | | KD4322 | 0 | | 0 | | 1 | | 1 | | 37.321 | | 37.059 | | 1.470194487 | |  |  |
| 799 | F | | 356 | | 565 | | SP2766 | | 0 | | 0 | | 1 | | 0 | | 36.25 | | 39.800 | | 2.787275806 | | | LV3565 | 0 | | 0 | | 0 | | 0 | | 0 | | 0 | | 0 | | | KD4319 | 0 | | 0 | | 1 | | 1 | | 35.694 | | 36.802 | | 2.981968207 | |  |  |
| 800 | M | | 261 | | 209 | | SP2765 | | 0 | | 0 | | 1 | | 0 | | 37.26 | | 38.239 | | 1.395232253 | | | LV3564 | 0 | | 0 | | 0 | | 0 | | 0 | | 0 | | 0 | | | KD4320 | 0 | | 0 | | 1 | | 1 | | 35.744 | | 35.458 | | 4.34242421 | |  |  |
| 801 | F | | 343 | | 480 | | SP2762 | | 0 | | 0 | | 1 | | 1 | | 35.97 | | 36.814 | | 2.624318405 | | | LV3561 |  | |  | | 0 | | 0 | | 0 | | 0 | | 0 | | | KD4316 | 0 | | 0 | | 1 | | 0 | | 35.572 | | 0 | | 4.408123644 | |  |  |
| 802 | M | | 265 | | 181 | | SP2767 | | 0 | | 0 | | 0 | | 0 | | 0.00 | | 0.00 | | 0 | | | LV3566 | 0 | | 0 | | 0 | | 0 | | 0 | | 0 | | 0 | | | KD4321 | 0 | | 0 | | 0 | | 1 | | 0 | | 36.696 | | 2.050094412 | |  |  |
| 803 | F | | 310 | | 362 | | SP2776 | | 0 | | 0 | | 1 | | 0 | | 37.57 | | 38.238 | | 1.131972078 | | | LV3575 | 0 | | 0 | | 0 | | 0 | | 0 | | 38.462 | | 0 | | | KD4330 | 0 | | 0 | | 1 | | 1 | | 35.94 | | 36.259 | | 3.0958268 | |  |  |
| 804 | M | | 245 | | 147 | | SP2769 | | 0 | | 0 | | 1 | | 1 | | 37.53 | | 37.579 | | 1.142983287 | | | LV3568 | 0 | | 0 | | 0 | | 0 | | 0 | | 0 | | 0 | | | KD4323 | 0 | | 0 | | 1 | | 0 | | 36.891 | | 38 | | 1.795116916 | |  |  |
| 805 | F | | 287 | | 279 | | SP2775 | | 0 | | 0 | | 1 | | 0 | | 36.78 | | 38.477 | | 1.934776521 | | | LV3574 | 0 | | 0 | | 0 | | 0 | | 0 | | 0 | | 0 | | | KD4329 | 0 | | 0 | | 1 | | 0 | | 37.491 | | 0 | | 1.192922864 | |  |  |
| 806 | M | | 260 | | 176 | | SP2771 | | 0 | | 0 | | 1 | | 0 | | 35.87 | | 38.441 | | 3.593466324 | | | LV3570 | 0 | | 0 | | 0 | | 0 | | 0 | | 0 | | 0 | | | KD4325 | 0 | | 0 | | 1 | | 1 | | 33.902 | | 36.678 | | 7.911712005 | |  |  |
| 807 | F | | 304 | | 357 | | SP2770 | | 0 | | 0 | | 1 | | 0 | | 37.49 | | 38.100 | | 1.195362878 | | | LV3569 | 0 | | 0 | | 0 | | 0 | | 37.896 | | 0 | | 0 | | | KD4324 | 0 | | 0 | | 1 | | 1 | | 36.967 | | 36.251 | | 2.240233445 | |  |  |
| 808 | M | | 239 | | 149 | | SP2785 | | 0 | | 0 | | 1 | | 1 | | 36.77 | | 37.444 | | 1.593852471 | | | LV3584 | 0 | | 0 | | 0 | | 0 | | 0 | | 0 | | 0 | | | KD4339 | 0 | | 0 | | 0 | | 1 | | 0 | | 32.486 | | 36.06571158 | |  |  |
| 809 | F | | 330 | | 475 | | SP2783 | | 0 | | 0 | | 1 | | 1 | | 36.24 | | 37.397 | | 2.032386704 | | | LV3581 | 0 | | 0 | | 0 | | 0 | | 0 | | 0 | | 0 | | | KD4336 | 0 | | 0 | | 1 | | 0 | | 35.313 | | 0 | | 5.258551484 | |  |  |
| 810 | M | | 260 | | 182 | | SP2781 | | 0 | | 0 | | 1 | | 1 | | 36.40 | | 36.497 | | 2.430412606 | | | LV3580 | 0 | | 0 | | 0 | | 0 | | 0 | | 38.282 | | 0 | | | KD4335 | 0 | | 0 | | 0 | | 0 | | 0 | | 0 | | 0 | |  |  |
| 811 | F | | 323 | | 369 | | SP2778 | | 0 | | 0 | | 1 | | 1 | | 36.66 | | 36.971 | | 1.899737003 | | | LV3576 | 0 | | 0 | | 0 | | 0 | | 0 | | 39.066 | | 0 | | | KD4331 | 0 | | 0 | | 1 | | 1 | | 36.955 | | 35.652 | | 2.94645279 | |  |  |
| 812 | M | | 247 | | 165 | | SP2772 | | 0 | | 0 | | 1 | | 1 | | 36.26 | | 36.915 | | 2.264358318 | | | LV3571 | 0 | | 0 | | 0 | | 0 | | 0 | | 0 | | 0 | | | KD4326 | 0 | | 0 | | 0 | | 0 | | 37.753 | | 37.873 | | 0 | |  |  |
| 813 | F | | 376 | | 574 | | SP2780 | | 0 | | 0 | | 1 | | 1 | | 37.48 | | 36.903 | | 1.490790199 | | | LV3579 | 0 | | 0 | | 1 | | 0 | | 36.846 | | 0 | | 1.850988701 | | | KD4334 | 0 | | 0 | | 1 | | 1 | | 36.919 | | 36.906 | | 1.769038489 | |  |  |
| 814 | M | | 238 | | 138 | | SP2774 | | 0 | | 0 | | 1 | | 0 | | 36.96 | | 0.00 | | 1.713871657 | | | LV3573 | 0 | | 0 | | 0 | | 0 | | 0 | | 0 | | 0 | | | KD4328 | 0 | | 0 | | 1 | | 1 | | 36.264 | | 35.099 | | 4.417566728 | |  |  |
| 815 | F | | 358 | | 518 | | SP2773 | | 0 | | 0 | | 1 | | 1 | | 36.69 | | 36.615 | | 2.111731679 | | | LV3572 | 0 | | 0 | | 0 | | 0 | | 0 | | 0 | | 0 | | | KD4327 | 0 | | 0 | | 1 | | 1 | | 35.657 | | 36.76 | | 3.061407723 | |  |  |
| 816 | M | | 213 | | 107 | | SP2786 | | 0 | | 0 | | 1 | | 0 | | 37.24 | | 0.00 | | 1.411481264 | | | LV3585 | 0 | | 0 | | 0 | | 0 | | 0 | | 0 | | 0 | | | KD4340 | 0 | | 0 | | 0 | | 1 | | 0 | | 35.742 | | 3.926156987 | |  |  |
| 817 | F | | 328 | | 426 | | SP2779 | | 0 | | 0 | | 1 | | 1 | | 36.57 | | 36.106 | | 2.648927524 | | | LV3578 | 0 | | 0 | | 0 | | 0 | | 0 | | 0 | | 0 | | | KD4333 | 0 | | 0 | | 0 | | 1 | | 0 | | 37.54 | | 1.15376705 | |  |  |
| 818 | M | | 268 | | 192 | | SP2784 | | 0 | | 0 | | 1 | | 1 | | 36.73 | | 37.236 | | 1.709815472 | | | LV3583 | 0 | | 0 | | 0 | | 0 | | 0 | | 38.044 | | 0 | | | KD4338 | 0 | | 0 | | 0 | | 1 | | 37.973 | | 36.854 | | 1.840930371 | |  |  |
| 6/3/2019 | | 819 | | F | | 334 | | 467 | | SP2777 | | 0 | | 0 | | 1 | | 1 | | 37.70 | | 36.513 | 1.679141483 | | | LV3577 | | 0 | | 0 | | 0 | | 0 | | 0 | | 0 | 0 | | | KD4332 | | 0 | | 0 | | 0 | | 1 | | 37.931 | | 37.354 | | 1.309595122 |
| 6/3/2019 | | 820 | | M | | 209 | | 93 | | SP2782 | | 0 | | 0 | | 1 | | 1 | | 37.72 | | 37.405 | 1.142416974 | | | LV3582 | | 0 | | 0 | | 0 | | 0 | | 0 | | 38.117 | 0 | | | KD4337 | | 0 | | 0 | | 0 | | 1 | | 0 | | 37.092 | | 1.565441393 |
